# Roads to pentazolate anion: a theoretical insight

**DOI:** 10.1098/rsos.172269

**Published:** 2018-05-23

**Authors:** Tao Yu, Yi-Ding Ma, Wei-Peng Lai, Ying-Zhe Liu, Zhong-Xue Ge, Gan Ren

**Affiliations:** State Key Laboratory of Fluorine and Nitrogen Chemicals, Xi'an Modern Chemistry Research Institute, Xi'an, People's Republic of China

**Keywords:** potential energy surface, reaction mechanism, polynitrogen, pentazole

## Abstract

The formation mechanism of pentazolate anion (PZA) is not yet clear. In order to present the possible formation pathways of PZA, the potential energy surfaces of phenylpentazole (PPZ), phenylpentazole radical (PPZ-R), phenylpentazole radical anion (PPZ-RA), PPZ and *m*-chloroperbenzoic acid (*m*-CPBA), *p*-pentazolylphenolate anion (*p*-PZPolA) and *m*-CPBA, and *p*-pentazolylphenol (*p*-PZPol) and *m*-CPBA were calculated by the computational electronic structure methods including the hybrid density functional, the double hybrid density functional and the coupled-cluster theories. At the thermodynamic point of view, the cleavages of C–N bonds of PPZ and PPZ-R need to absorb large amounts of heat. Thus, they are not feasible entrance for PZA formation at ambient condition. But excitation of PPZ and deprotonation of PPZ-RA probably happen before cleavage of C–N bond of PPZ at high-energy condition. As to the radical anion mechanism, the high accuracy calculations surveyed that the barrier of PZA formation is probably lower than that of dinitrogen evolution, but the small ionization potential of PPZ-RA gives rise to the unstable ionic pair of sodium PPZ at high temperature. In respect of oxidation mechanism, except for PPZ, the reactions of *p*-PZPolA and *p*-PZPol with *m*-CPBA can form PZA and quinone. The PZA formations have the barriers of about 20 kcal mol^−1^ which compete with the dinitrogen evolutions. The stabilities of PZA in both solid and gas phases were also studied herein. The proton prefers to transfer to pentazolyl group in the (N_5_)_6_(H_3_O)_3_(NH_4_)_4_Cl system which leads to the dissociation of pentazole ring. The ground states of M(N_5_)_2_(H_2_O)_4_ (M = Co, Fe and Mn) are high-spin states. The pentazolyl groups confined by the crystal waters in the coordinate compounds can improve the kinetic stability. As to the reactivity of PZA, it can be persistently oxidized by *m*-CPBA to oxo-PZA and 1,3-oxo-PZA with the barriers of about 20 kcal mol^−1^.

## 1. Introduction

Nitrogen is located at the second period and group 15 of the element table. The standard state manifests as a diatomic gas with a formula of N_2_. Owing to the highly strong triple bond, it imparts a low chemical reactivity. By contrast to formation, the combustion or decomposition of a nitrogenous compound ultimately releases dinitrogen following the thermodynamics. Consequently, the homopolyatomic states of nitrogen are metastable with high energy. Although they are difficult to synthesize, the innovation of the element chemistry and the exploration of the stable margin have been attractive to the science community, and the promising applications on the space, defence and oil drilling technologies have been interesting to the engineering community.

Since azide anion [1] (N_3_^−^) and pentazenium cation [2] (N_5_^+^) were discovered, pentazolate anion (N_5_^−^) had been expected to be the third homopolynitrogen species in bulk based on the successful synthesis of arylpentazoles [3]. Some high-level calculations indicated that the dissociation barrier towards N_3_^−^ and N_2_ exceeds 25 kcal mol^−1^, e.g. 26 kcal mol^−1^ at CASPT2/ANO(4s3p2d) level by Curtiss *et al.* [4], 27.7 kcal mol^−1^ at CCSD(T)/aug-cc-pVTZ level by Nguyen & Ha [5], and 27.2 kcal mol^−1^ at CCSD(T)/CBS level by Dixon *et al.* [6]*.* The sufficient barrier makes pentazolate anion (PZA) possible to be held at ambient condition. Over time, PZA was only detected in spectrum as to the experimental attempts. Vij *et al.* [7] in 2002 observed the *m/z* 70 (71 for a pentazole ring containing one ^15^N) peak of mass spectrometry using electrospray ionization (ESI) at high collision voltages for *p*-pentazolylphenolate anion (*p*-PZPolA). The removal of aryl group of *p*-PZPolA undergoes an intersystem crossing between singlet and triplet states with a barrier of 63.8 kcal mol^−1^ at the CASSCF(12,11)/DZV level [8]. Östmark *et al.* [9] in 2003 also discovered the peak of mass spectrometry using a relatively mild ionization, i.e. UV-laser desorption ionization (LDI), for *p*-dimethylaminophenylpentazole. Although the starting material also contains the arylpentazolyl structure, *p*-dimethylaminophenylpentazole forms PZA in a different pathway. The cleavage of the C–N bond has a radical anion mechanism with a barrier of 21.1 kcal mol^−1^ at the B3LYP/6-311+(2df,p) level [9]. However, ESI and UV-LDI methods cannot keep PZA a long life. Bazanov *et al.* [10] in 2016 treated phenylpentazole (PPZ) with sodium in tetrahydrofuran (THF), and the mass spectrometry of PZA was detected with an elution time of 2.1 min after injection into the HPLC column. The electron transfer from sodium to PPZ also follows the radical anion mechanism [11]. The cleavage of the C–N bond competes with the evolution of dinitrogen. Their ^15^N labelled experiment further substantiated the discovery of PZA in solution in 2017 [12]. The detection of PZA in an extreme condition reported by Steele *et al.* [13] in 2017 is the phase change for mixture of caesium azide and liquid dinitrogen in a diamond anvil cell (DAC) at a super high pressure near 60 GPa. The existence of PZA was supported by XRD, Raman and computational studies, but the PZA disappeared when the external pressure was relieved [13]. Laniel *et al.* [14] in 2018 observed PZA in DAC around 45 GPa using lithium embedded in dinitrogen. The Raman modes and *m/z* 70 peak of mass spectrometry for PZA could still be measured at ambient condition which means LiN_5_ is metastable [14]. As to the chemical synthesis ways, the redox reactions were designed for the cleavage of C–N bond of *p*-pentazolylphenol (*p*-PZPol) derivatives. Butler *et al*. [15] in 2003 claimed that PZA was identified by NMR spectroscopy using the oxidizer of cerium ammonium nitrate. The discovery was argued by Schroer *et al*. [16] in 2005. The reinvestigation of Butler *et al*. [17] in 2008 still did not find the direct evidence on PZA. Although the computational result of Perera *et al*. [18] in 2009 for the spin–spin constants of ^15^N–^15^N supported the observation of Butler in 2003. Most recently, there has been breakthrough in 2017 for the synthesis of PZA in bulk. Zhang *et al.* [19] managed to cleave PZA out of 3,5-dimethyl-4-hydroxyphenylpentazole through treatment with *m*-chloroperbenzoic acid (*m*-CPBA) and ferrous bisglycinate and successfully isolated a stable salt of (N_5_)_6_(H_3_O)_3_(NH_4_)_4_Cl in the crystalline state with a decomposition onset temperature of 117°C. Afterwards they obtained the crystal structure of Co(N_5_)_2_(H_2_O)_4_·4H_2_O [20]. Likewise based on (N_5_)_6_(H_3_O)_3_(NH_4_)_4_Cl, the three-dimensional frameworks with the formulae of N_80_Na_15.33_O_5_ and [Ag(NH_3_)_2_]^+^[Ag_3_(N_5_)_4_]^−^ were synthesized by Zhang *et al.* [21] and Sun *et al.* [22] in 2018, respectively. Xu *et al.* [23] in 2017 used almost the same approach, but obtained a different crystal with a formula of Na(N_5_)(H_2_O)·2H_2_O. Based on the sodium pentazolate hydrate, a series of metal salts including M(N_5_)_2_(H_2_O)_4_·4H_2_O (M = Fe and Mn) and Mg(H_2_O)_6_(N_5_)_2_·4H_2_O were synthesized. Afterwards they synthesized three anhydrous and metal-free salts including (N_5_^−^)_2_DABTT^2+^ (DABTT = 3,9-diamino-6,7-dihydro-5*H*-bis([1,2,4]triazolo)[4,3-e:3′,4′-g][1,2,4,5] tetrazepine-2,10-diium), N_5_^−^GU^+^ (GU = *N*-carbamoylguanidinium) and N_5_^−^Oxahy^+^ (Oxahy = oxalohydrazinium) [24].

Since the successful synthesis of N_5_^+^ in 1999 [2], the formation mechanisms for N_2_F^+^ + HN_3_ [25,26] and ONF_2_^+^ + HN_3_ [26–28] had been thoroughly expounded by the aids of theoretical and computational chemistry. Although the great advances of PZA have been made, there are still questions regarding the mechanisms on its formation and stabilization. Thus, the computational tasks on the reaction pathways for the high-energy, radical anion and redox methods were put forward. Our investigations include the following contents: the potential energy surface (PES) scans around PPZ, PPZ-R (phenylpentazole radical), PPZ-RA (phenylpentazole radical anion), PPZ and *m*-CPBA, *p*-PZPolA and *m*-CPBA, *p*-PZPol and *m*-CPBA and M(N_5_)_2_(H_2_O)_4_ (M = Co, Fe and Mn), and the evaluations of PZA stabilities in both isolated and condensed states. The computational results are expected to be useful for deeply understanding the characteristics of PZA, and tutoring the design and synthesis of the high-energy compounds comprising PZA in the future works.

## 2. Computational methods

### 2.1. Quantum chemistry

The total electronic energies of the isolated states were calculated by the B3LYP [29,30] (a hybrid density functional), RI-B2KPLYP (a double hybrid density functional with the kinetic reparametrization [31] of B2PLYP [32] and RI approximation [33] used for the second-order many-body perturbation part) and CCSD(T) [34–38] (coupled-cluster singles and doubles with perturbative triples) methods. The basis set of 6-311++G** for CHNO elements [39] and effective core potential of LanL2 with valence basis set of LanLDZ for CoFeMn elements [40,41] were used to express the wave functions of the B3LYP method. The basis set of ma-def2-TZVP [42,43] (the auxiliary basis of RI approximation was automatically constructed) was used to combine with the RI-B2KPLYP method. Based on the geometries optimized at the B3LYP/6-311++G** level, the complete basis set (CBS) was used to combine with the frozen core CCSD(T) method. The basis set extrapolation [44] for CBS deduced by cc-pVDZ [45] and cc-pVTZ [45] was divided into the Hartree–Fock (HF) and correlated parts. Using the different converged approaches of two parts, the small basis sets can efficiently get to the complete limit. The specific equation on the total electronic energy calculated at the CCSD(T)/CBS level is presented as follows:

2.1 $$\begin{array}{rl} E_{\mathrm{CBS}}^{\mathrm{CCSD}(T)} & =\frac{3^{3.4}}{3^{3.4}-2^{3.4}}E_{\mathrm{cc}-\mathrm{pVTZ}}^{\mathrm{HF}}-\frac{2^{3.4}}{3^{3.4}-2^{3.4}}E_{\mathrm{cc}-\mathrm{pVDZ}}^{\mathrm{HF}}+\frac{3^{2.4}}{3^{2.4}-2^{2.4}}(E_{\mathrm{cc}-\mathrm{pVTZ}}^{\mathrm{CCSD}(T)}-E_{\mathrm{cc}-\mathrm{pVTZ}}^{\mathrm{HF}})\\ & \;-\frac{2^{2.4}}{3^{2.4}-2^{2.4}}(E_{\mathrm{cc}-\mathrm{pVDZ}}^{\mathrm{CCSD}(T)}-E_{\mathrm{cc}-\mathrm{pVDZ}}^{\mathrm{HF}}). \end{array}$$

The spin-unrestricted computational methods were applied to all the systems in the text. The test results of different methods on energy barriers are presented in electronic supplementary material, table S1. There is a little deviation between RI-B2KPLYP/ma-def2-TZVP and CCSD(T)/CBS calculations. Considering the computational cost, the accuracy at the B3LYP/6-311++G** level is also acceptable. The B3LYP, RI-B2KPLYP and CCSD(T) methods were realized by the Gaussian 09 [46] and ORCA 3.0 [47] program packages. The polarized continuum model [48] of integral equation form [49,50] (IEF) was used to evaluate the solvent effects. For ionic pairs with unknown packing modes, their stabilities were presented by Born–Haber cycles. The lattice potential energies were empirically estimated by the ionic pair volumes inside a contour of 0.001 electrons Bohr^−3^ at the HF/6-311++G**// B3LYP/6-311++G** level. It must be noted that these calculations only involve the isolated states. For 1 : 1 salts, the specific equation on the lattice potential energies is presented as follows [51]:

2.2 $$U_{\mathrm{POT}}=2\left(\frac{28.0}{\sqrt[{3}]{V}}+12.4\right)\text{kcal mo}{\text{l}}^{-1},$$

where *V* represents the ionic pair (formula unit) volume in nm^3^. The lattice enthalpy corrections for monatomic, linear and polyatomic ions are −0.5RT, 0.5RT and RT, respectively.

### 2.2. Solid physics

The PBE [52,53] method (a density functional with generalized gradient approximation) combined with on the fly generation ultrasoft [54] potential for Materials Studio 8.0 [55] (OTFG_80) and semi-empirical dispersion correction of Tkatchenko & Scheffler [56] was used for periodic boundary condition. Brillouin zone sampling on a grid of spacing 2π × 0.07 Å^−1^ and a plane-wave basis set cut-off of 630 eV were set. This calculation was realized by the CASTEP [57] plane-wave code.

### 2.3. Potential energy surfaces

There are three kinds of optimized geometries on the PESs, i.e. the local minimum energy points, the first-order saddle points and the minimum energy crossing points (MECPs). The local minimum energy points involve the equilibrium geometries of reactants, intermediates (IMs) and products which locate at the lowest energy points within the 3*N *− 6 dimensional spaces of PESs (suppose a nonlinear molecule, *N* represents the atom number of molecule). The first-order saddle points involve the transition states (TSs) which locate at the minimum energy points except for only one direction (only valid for the selected cell of periodic boundary condition) of the 3*N *− 6 dimensions. The MECPs involve the crossings between the two spin states which locate at the minimum energy points within the 3*N *− 7 dimensional subspaces of PESs of the crossing hyperlines. They can be optimized along the first and second gradients of the square of the difference between the total energies of two states. The optimized *xyz* coordinates in this paper are presented in the electronic supplementary material. The dimensionality of PESs can be checked by the frequency or phonon calculations. All reactants, IMs, products and MECPs have no imaginary frequency, and TSs have only one imaginary frequency. The thermodynamic properties can also be deduced by frequency or phonon calculations. The activation free energy barriers were derived by the differences counted from TSs or MECPs to reactants or products. Unless otherwise specified, the barriers mainly stand for the activation free energy barriers at 298 K calculated at the B3LYP/6-311++G** level in the text.

## 3. Results and discussion

### 3.1. Reaction pathways for phenylpentazole

**Figure 1.**
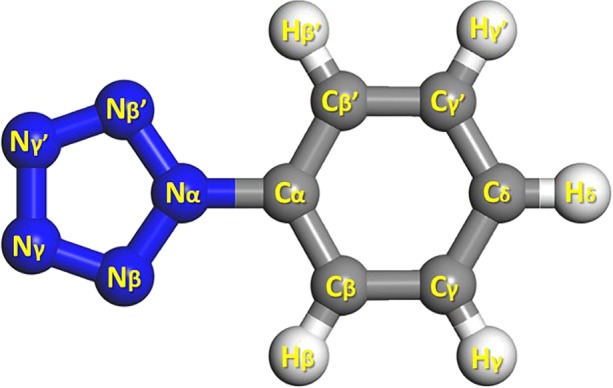
Atomic labels for PPZ, PPZ-R and PPZ-RA and their derivatives.

PPZ consists of a pentazolyl group bonded to a phenyl group. Beside PPZ (singlet state), PPZ-R (triplet state) and PPZ-RA (doublet state) have the same symmetry of *C*_2v_. For the sake of argument of PESs on the changes of atomic positions, the labels of PPZ and their derivatives are shown in figure 1.

**Figure 2.**
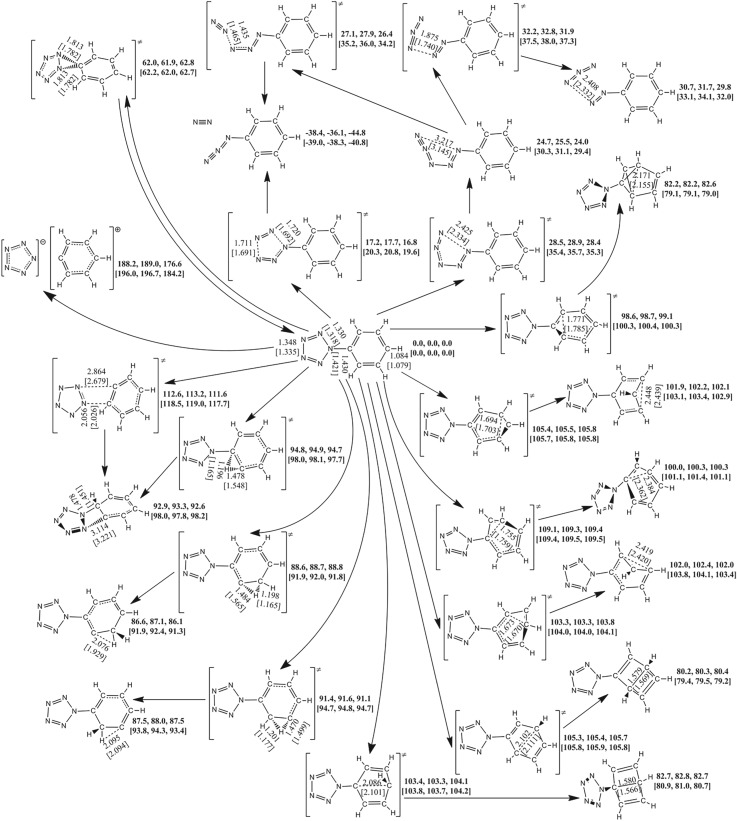
The PES scanning result for PPZ calculated at the B3LYP/6-311++G** and RI-B2KPLYP/ma-def2-TZVP (in brackets) levels. The energy (sum of total electronic energy and ZPC at front, enthalpy of 298 K at middle and free energy of 298 K at back) scales are offset to 0 kcal mol^−1^ for PPZ as the reference. The crucial bond lengths are labelled in Å.

**Figure 3.**
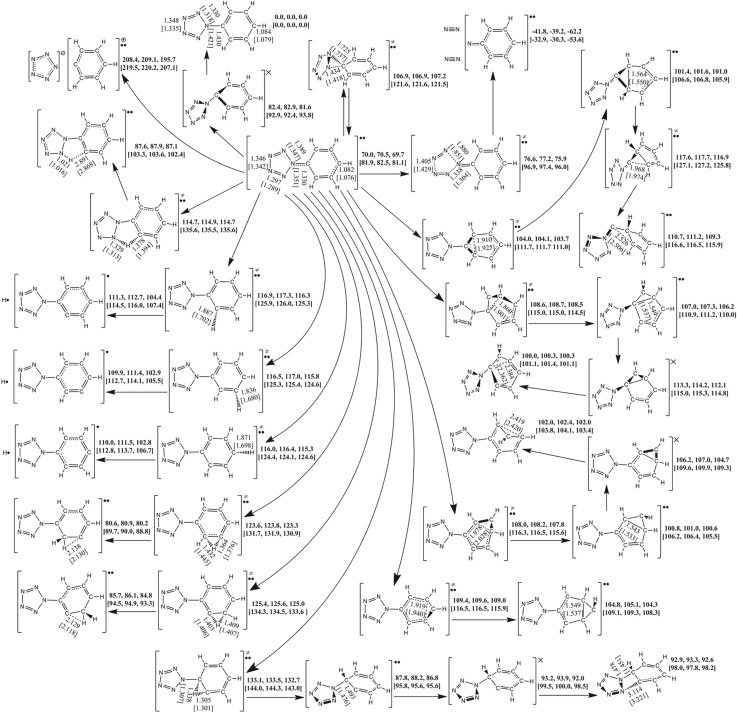
The PES scanning result for PPZ-R calculated at the B3LYP/6-311++G** and RI-B2KPLYP/ma-def2-TZVP (in brackets) levels. The energy (sum of total electronic energy and ZPC at front, enthalpy of 298 K at middle and free energy of 298 K at back) scales are offset to 0 kcal mol^−1^ for PPZ as the reference. The crucial bond lengths are labelled in Å.

**Figure 4.**
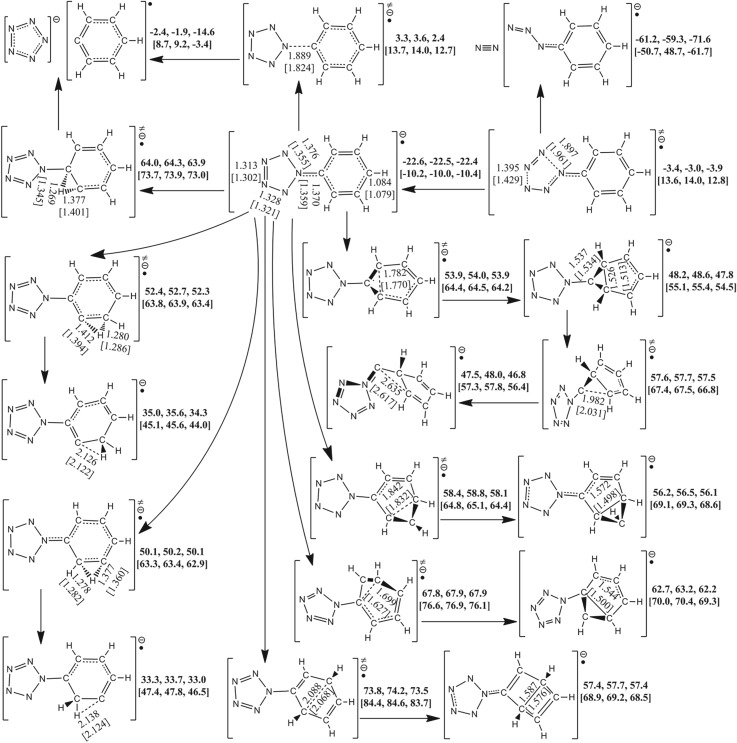
The PES scanning result for PPZ-RA calculated at the B3LYP/6-311++G** and RI-B2KPLYP/ma-def2-TZVP (in brackets) levels. The energy (sum of total electronic energy and ZPC at front, enthalpy of 298 K at middle and free energy of 298 K at back) scales are offset to 0 kcal mol^−1^ for PPZ as the reference. The crucial bond lengths are labelled in Å.

The reactions starting with a single molecule usually impart two kinds of modes, i.e. isomerization and dissociation. As to the same product, it can be achieved directly or indirectly. In this way, the PESs around PPZ and their derivatives are presented in figures 2–4. As shown in figure 2, there are concerted and stepwise mechanisms for dissociation of PPZ to azidobenzene with evolution of dinitrogen. The concerted pathway involving the breakages of N_α_–N_β_ and N_γ_–N_γ_ bonds has a barrier of 16.8 kcal mol^−1^. The stepwise pathway involves an IM, 1-azido-2-phenyldiazene, with a N_5_ chain. Because the free energy of TS of the first step is higher than that of the second step, the determinant barrier of 28.4 kcal mol^−1^ for dissociation only involves the breakage of N_α_–N_β_ bond in the first step. The IM can also dissociate into benzenediazonium cation and azide anion, and the effective barrier counted from PPZ is 31.9 kcal mol^−1^. There is only stepwise pathway for the formation of PPZ via reaction between benzenediazonium cation and azide anion, i.e. the reverse pathway for dissociation. The determinant IM, azidophenyldiazene, is located in a shallow potential well for dissociating to azidobenzene and cyclizing to PPZ. The dissociation and cyclization barriers are 2.4 and 4.4 kcal mol^−1^, respectively. The computational results are consistent with the previous study on the dissociation and formation of PPZ analogue [58,59].

The carbon–carbon coupling and proton transfer can produce isomers of PPZ. The coupling barriers for C_β_–C_β′_, C_γ_–C_γ′_, C_α_–C_γ_, C_β_–C_δ_, C_β_–C_γ′_ and C_α_–C_δ_ are 99.1, 105.8, 109.4, 103.8, 105.7 and 104.1 kcal mol^−1^, respectively. The proton transfers from C_β_ to C_α_, from C_β_ to C_γ_ and from C_γ_ to C_β_ have the barriers of 94.7, 88.8 and 91.1 kcal mol^−1^, respectively. These coupling and transfer reactions are endothermic. The processes impart above 75% contributions to barriers. Owing to the ultra-high barriers, they are difficult to initiate subsequent reactions at ambient condition.

The cleavages of C_α_–N_α_ bonds can proceed along two different pathways. One involves the breakage of C_α_–N_α_ and the coupling of C_α_–N_β_ with a barrier of 62.8 kcal mol^−1^, but the production is still PPZ. The other involves the breakage of C_α_–N_α_ and the coupling of C_β_–N_β_ with a barrier of 111.6 kcal mol^−1^. The proton transfer from C_β_ to C_α_ can also form the same isomer with a barrier of 94.7 kcal mol^−1^. At the thermodynamic point of view, the formation of benzenium cation and pentazolate anion is endothermic associated with a free energy of 176.6 kcal mol^−1^. The preferred pathway for PPZ is dinitrogen evolution at ambient condition. The barriers for C–C coupling and proton transfer are lower than 176.6 kcal mol^−1^. Thus, they are possible at high-energy condition.

### 3.2. Reaction pathways for phenylpentazole radical

As shown in figure 3, PPZ-R lies 69.7 kcal mol^−1^ (adiabatic excited energy) above PPZ. There are several entrances to the singlet states, such as carbon–carbon coupling, proton transfer and non-planarization. Each pathway for carbon–carbon coupling undergoes a triplet TS, a triplet IM and a MECP. The effective barriers, counted from PPZ to TS (or MECP) which has the highest potential energies along the pathway, for C_α_–C_γ_ and C_β_–C_δ_ couplings are 42.4 and 35.0 kcal mol^−1^, respectively. The proton transfer from C_β_ to C_α_ can also lead to the singlet state. The determinant pathway is the isomerization in the triplet system with a barrier of 63.0 kcal mol^−1^. The direct pathway from PPZ-R to PPZ undergoes a non-planarized MECP with a barrier of 11.9 kcal mol^−1^.

The C_β_–C_β′_ coupling has a barrier of 34.0 kcal mol^−1^ which is lower than that of C_γ_–C_γ′_ coupling by 5.3 kcal mol^−1^, and the further reaction can produce a structure with a carbon atom bridging a cyclopentyl group and a pentazolyl group. The free energy of the bridging structure is higher than that of the C_β_–C_β′_ coupling structure by 8.3 kcal mol^−1^.

The proton transfer from C_β_ to N_β_ has a barrier of 45.0 kcal mol^−1^, and the endothermic process is associated with a free energy of 17.4 kcal mol^−1^, but it cannot form pentazole successively. The proton transfers from C_β_ to C_γ_ and from C_γ_ to C_β_ have the barriers of 53.6 and 55.3 kcal mol^−1^, respectively. The deprotonations of H_α_, H_γ_ and H_δ_ have the barriers of 46.6, 46.1 and 45.6 kcal mol^−1^, respectively. They are endothermic, and impart about 28% contributions to barriers.

The cleavage of C_α_–N_α_ accompanied by the coupling of C_α_–N_β_ gives rise to no chemical change for PPZ-R. At the thermodynamic point of view, the dissociation energy of the formation of benzenium radical cation and pentazolate anion is 126.0 kcal mol^−1^. Although the barrier of dinitrogen evolution is only 6.6 kcal mol^−1^, the C–C couplings, proton transfers and deprotonations still have the lower energies than 176.6 kcal mol^−1^ for C–N cleavage of PPZ. Thus, the possible pathway of the formation of PZA at high-energy condition is that PPZ is excited to PPZ-R at the first step, the proton is abstracted at the second step and PZA is formed at the subsequent step.

### 3.3. Reaction pathways for phenylpentazole radical anion

As shown in figure 4, the free energy of PPZ-RA is higher than that of PPZ by 22.4 kcal mol^−1^ (adiabatic ionization potential (IP) for PPZ-RA). Similar to PPZ and PPZ-R, the pathways of carbon–carbon coupling and proton transfer have the very high barriers, and need external heat. The coupling barriers for C_β_–C_β′_, C_β_–C_δ_, C_α_–C_γ_ and C_β_–C_γ′_ are 76.3, 80.5, 90.3 and 95.9 kcal mol^−1^, respectively. The C_β_–C_β′_ coupling product can isomerize to a structure with a carbon atom bridging two five-membered ring groups with an effective barrier counted from PPZ-RA of 79.9 kcal mol^−1^. The proton transfers from C_β_ to C_γ_ and from C_γ_ to C_β_ have the barriers of 74.7 and 72.5 kcal mol^−1^, respectively. The formation of pentazolate anion can proceed along two different pathways, and absorb 7.8 kcal mol^−1^ heat. One is the proton transfer from C_β_ to C_α_ leading to the cleavage of C_α_–N_α_ bond with a barrier of 86.3 kcal mol^−1^. The other is the direct cleavage of C_α_–N_α_ bond which competes against the dinitrogen evolution. The barriers for the pentazolate anion formation and the dinitrogen evolution are 24.8 and 18.5 kcal mol^−1^ at the B3LYP/6-311++G** level, respectively. In contrast to the calculated result at the RI-B2KPLYP/ma-def2-TZVP level, the difference between the two barriers is smaller and the high–low relationship is still kept, i.e. 23.1 kcal mol^−1^ for the pentazolate anion formation and 23.2 kcal mol^−1^ for the dinitrogen evolution. The same trend was reported using the average of three high accuracy methods [11]. As shown in electronic supplementary material, table S2, the solvent effect of THF has little impact on the correlative barriers.

It must be prudent to re-compute the two critical barriers using the theoretical method as accurately as possible. The re-calculated result survey that the high–low relationship between the two barriers at the CCSD(T)/CBS level is opposite to those at the B3LYP/6-311++G** and RI-B2KPLYP/ma-def2-TZVP levels, i.e. 25.5 kcal mol^−1^ for the pentazolate anion formation and 26.7 kcal mol^−1^ for the dinitrogen evolution at the CCSD(T)/CBS level.

**Figure 5.**
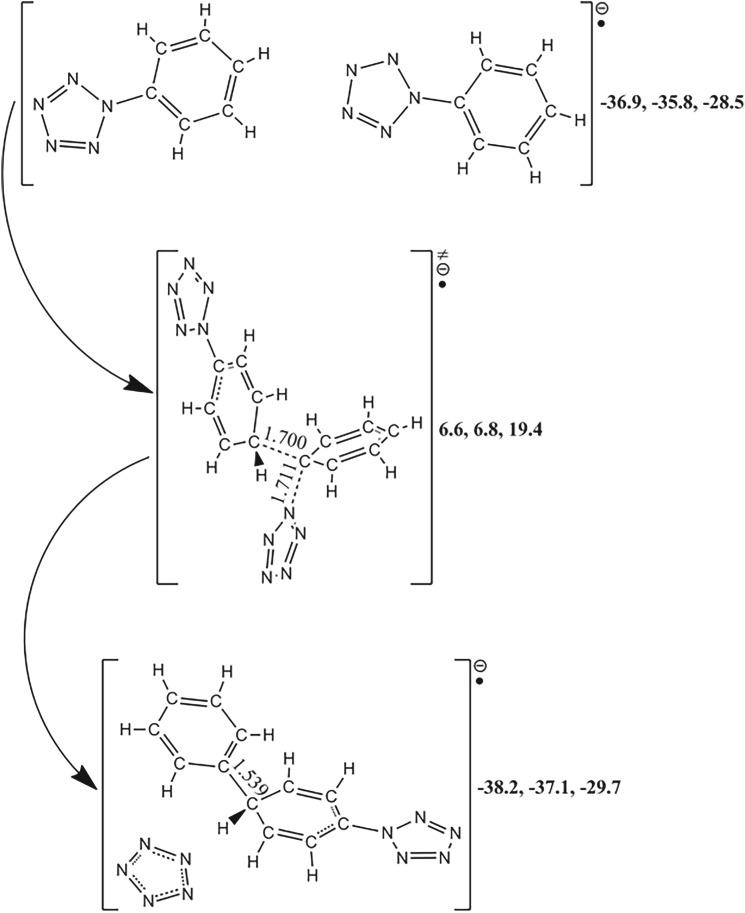
The PZA formation pathway starting with PPZ-RA and PPZ calculated at the B3LYP/6-311++G** level. The energy (sum of total electronic energy and ZPC at front, enthalpy of 298 K at middle and free energy of 298 K at back) scales are offset to 0 kcal mol^−1^ for two PPZs as the reference. The crucial bond lengths are labelled in Å.

The radical production is another important issue. As shown in figure 5, the reaction between PPZ and PP-RA can produce pentazolate anion and biphenyl pentazole with a barrier of 47.9 kcal mol^−1^. Although the long-range interaction of the reaction complex causes the deeper potential well by 14.3 kcal mol^−1^, the formation barrier of pentazolate anion in the presence of PPZ is higher than that in the absence of PPZ indeed. The possible pathway may be the formation of phenyl radical at the first step, and spontaneous formation of polyphenyl at the second step. It is the possible origin of gel [10].

### 3.4. Electron transfer in sodium phenylpentazole

**Figure 6.**
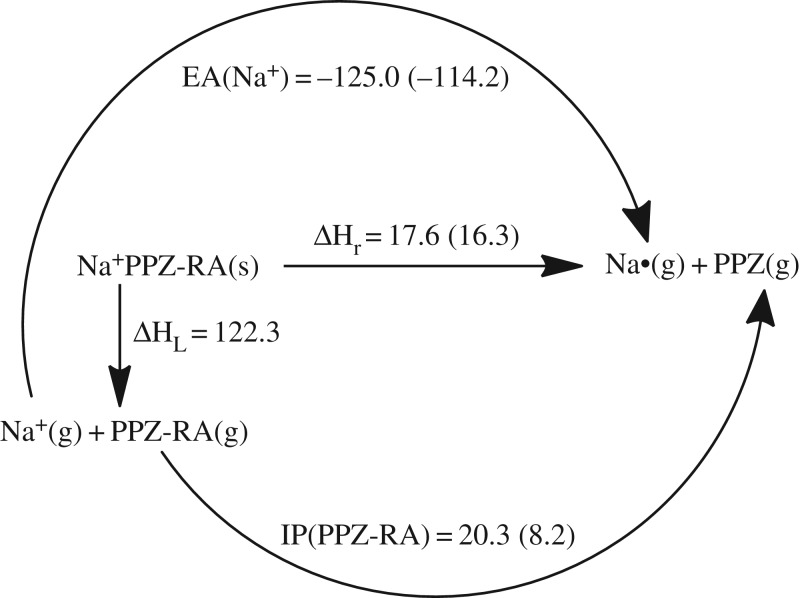
Born–Haber cycle in kcal mol^−1^ for the dissociative reaction of solid Na^+^PPZ-RA. The IP and EA are calculated at the B3LYP/6-311++G** and the CCSD(T)/CBS (in parentheses) levels, and the lattice enthalpy is estimated by an ionic pair volume of 0.192 nm^3^.

Besides self-dissociation, the stability of PPZ-RA also depends on the electron transfer from it to sodium cation. The process generally undergoes the formation of the neutral counterparts of ions without a barrier. If the electron transfer free energy has a negative value, the determinant factor on stability will be the dissociation barrier of PPZ. Otherwise, the positive energy will provide an extra barrier to PPZ. The Born–Haber cycle shown in figure 6 indicates that the IP value of PPZ-RA is not large enough to cover the electron affinity (EA) value of sodium cation, but when the lattice enthalpy is taken into account, the electron transfer enthalpy (17.6 kcal mol^−1^ at 298 K) becomes positive. The reactivity depends on the free energy change. Consequently, the contribution of entropy cannot be neglected. These kinds of reactions always go through the entropy increasing process which can reduce the enthalpy. The dinitrogen evolution barrier for PPZ is lower than the PZA formation barrier from PPZ-RA by 8.0 kcal mol^−1^ at 298 K. Thus, the entropy correction (*T*Δ*S*) at 298 K must be less than 9.6 kcal mol^−1^ for the dominant formation of pentazolate anion. Given the calculated entropies of PPZ and Na radical of 90.7 and 36.7 cal mol^−1^ K^−1^, respectively, the entropy of sodium PPZ of more than 94.6 cal mol^−1^ K^−1^ can meet the requirement. Some known sodium salts can be used as references, e.g. the entropies of sodium azide, sodium cyanide, sodium cyanate, sodium nitrate, sodium formate, sodium hydrogen carbonate and sodium acetate of 23.2, 16.8, 20.7, 22.2, 19.8, 20.9 and 19.1 cal mol^−1^ K^−1^, respectively [60]. As such, the goal of 94.6 cal mol^−1^ K^−1^ is hard to achieve. If the entropy of sodium azide is used to estimate the electron transfer reaction free energy at 298 K, it is equal to −13.5 kcal mol^−1^. The lattice energy for the crystalline state almost represents the limitation of binding ability. The ionic pair can be formed at a very low temperature. In consideration of the barrier of radical anion mechanism of 25.5 kcal mol^−1^ at the CCSD(T)/CBS level, the pentazolate anion formation should be handled at a relative high temperature which can also lead to the dissociation of PZA. The computational result is consistent with the experimental evidence which indicated that PPZ treated with clean Na leads to dissociation, but PZA can be formed by PPZ treated with passivated Na [12].

### 3.5. Reaction pathways for phenylpentazole and *m*-chloroperbenzoic acid

**Figure 7.**
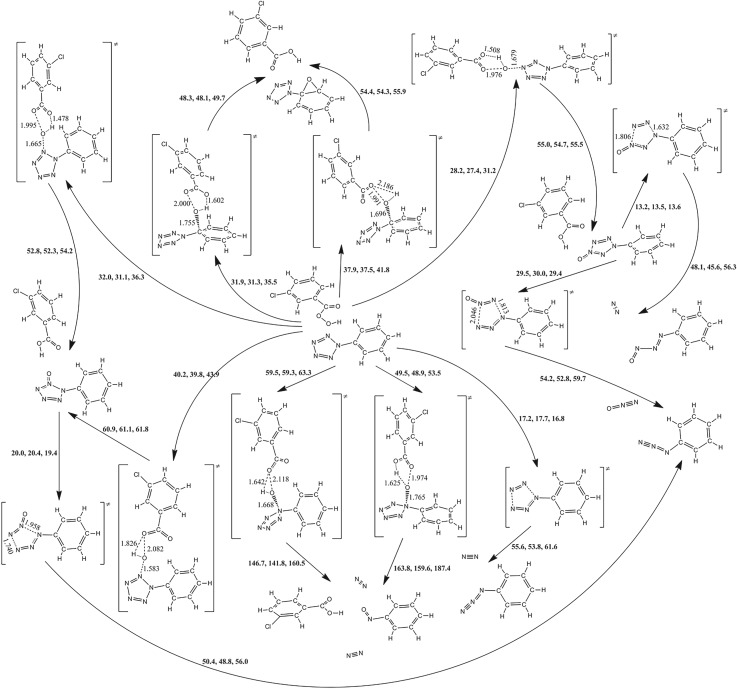
The PES scanning result for PPZ and *m*-CPBA calculated at the B3LYP/6-311++G** level. The activation energy barriers (sum of total electronic energy and ZPC at front, enthalpy of 298 K at middle and free energy of 298 K at back) are in kcal mol^−1^. The crucial bond lengths are labelled in Å.

**Figure 8.**
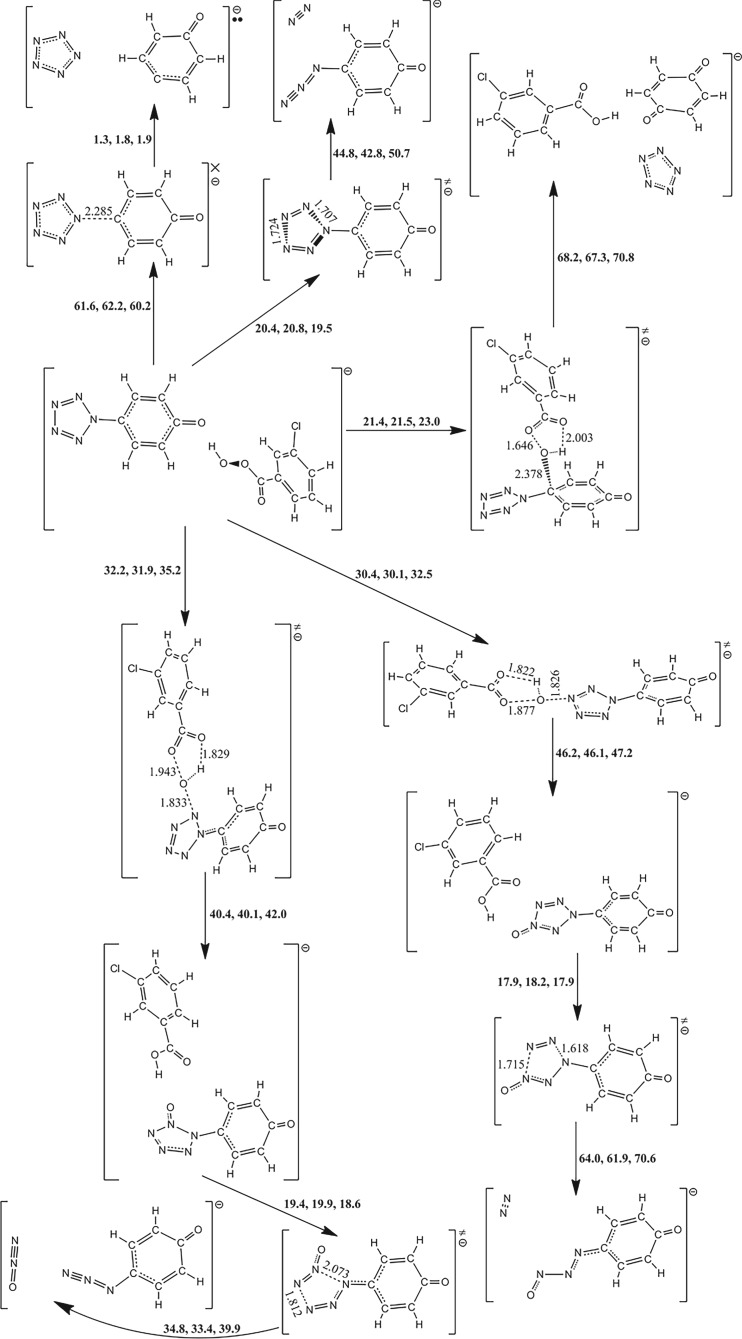
The PES scanning result for PZPolA and *m*-CPBA calculated at the B3LYP/6-311++G** level. The activation energy barriers (sum of total electronic energy and ZPC at front, enthalpy of 298 K at middle and free energy of 298 K at back) are in kcal mol^−1^. The crucial bond lengths are labelled in Å.

**Figure 9.**
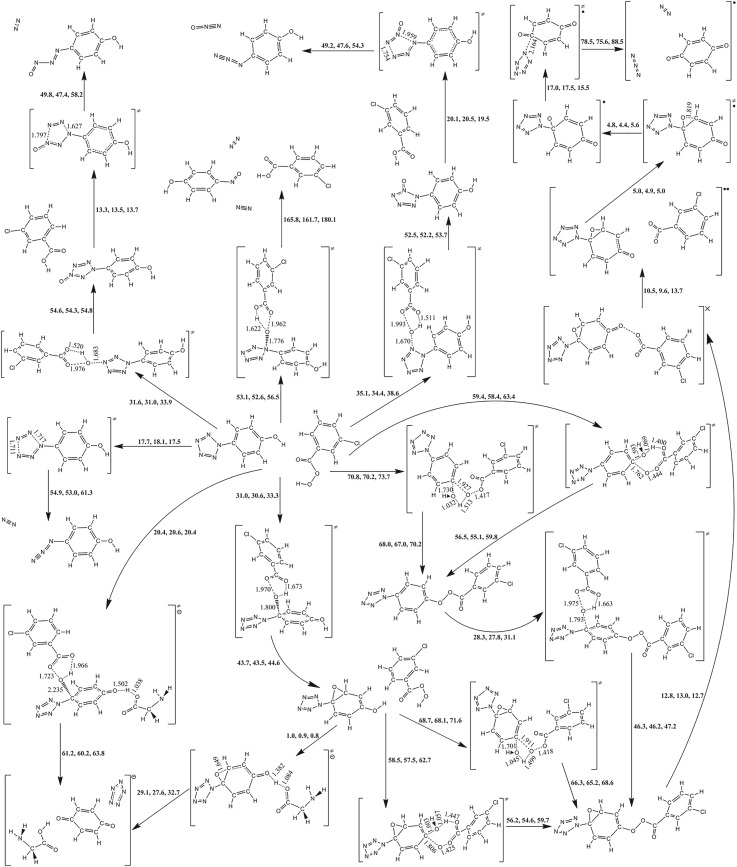
The PES scanning result for PZPol and *m*-CPBA calculated at the B3LYP/6-311++G** level. The activation energy barriers (sum of total electronic energy and ZPC at front, enthalpy of 298 K at middle and free energy of 298 K at back) are in kcal mol^−1^. The crucial bond lengths are labelled in Å.

Owing to the multiple reactive sites for multi-molecule, the oxidation mechanisms of *m*-CPBA are more complex. Figures 7–9 present the information on the reactions of PPZ, PZPolA and PZPol with *m*-CPBA as much as possible. There are two isomers of *m*-CPBA, i.e. the chorophenyl points either to the same direction as the carbonyl or to the opposite direction. The two isomers have almost the same potential energy with a conversion barrier of 5.9 kcal mol^−1^. The detailed pathways for *m*-CPBA are presented in electronic supplementary material, figure S1. Thus, the computational result will not be affected by the selection of isomers.

The reactions between PPZ and *m*-CPBA can proceed along two kinds of pathways, i.e. the oxidizations of pentazolyl and phenyl groups. The transition states on *m*-CPBA concern the breakages of peroxyl bond and the transfers of proton. As shown in figure 7, the barriers of the proton transfers to the original carbonyl groups are usually higher than those of the proton transfers to the original peroxyl groups. Thus, the latter oxidations were not involved in further consideration.

The cleavage of C_α_–N_α_ is not induced by the attack of the oxygen of *m*-CPBA on the C_α_ of PPZ, but it forms a benzene oxide structure with a barrier of 35.5 kcal mol^−1^. The oxidizations of N_α_, N_β_ and N_γ_ of PPZ have the barriers of 53.5, 36.3 and 31.2 kcal mol^−1^, respectively, and they involve the dinitrogen evolution, the N_β_-oxo-PPZ formation and the N_γ_-oxo-PPZ formation, respectively. The N_β_-oxo-PPZ dissociates to azidobenzene with a barrier of 19.4 kcal mol^−1^. The N_γ_-oxo-PPZ dissociates to azidobenzene and nitrosophenyldiazene with the barriers of 29.4 and 13.6 kcal mol^−1^, respectively.

According to the potential energy scanning result, the barriers of the reactions between PPZ and *m*-CPBA are higher than that of PPZ self-dissociation, and there is no pathway concerning the formation of PZA.

### 3.6. Reaction pathways for pentazolylphenolate anion and *m*-chloroperbenzoic acid

As shown in figure 8, the cleavage of C_α_–N_α_ bond of PZPolA undergoes a MECP, and forms phenyl radical and PZA. It has a very high barrier of 60.2 kcal mol^−1^ which agrees with the previous computational investigation [8]. Comparatively, the dinitrogen evolution barrier of PZPolA is only 19.5 kcal mol^−1^. Although it is one of the most kinetic stable PPZ derivatives [61].

The pentazolyl oxidization barriers in the PZPolA and *m*-CPBA system are approximate to those in the PPZ and *m*-CPBA system. The oxidizations of N_β_ and N_γ_ of PZPolA have the barriers of 35.2 and 32.5 kcal mol^−1^, respectively. The oxidation of the C_α_ of PZPolA prefers to form benzoquinone and transfer the electron to pentazole. The barrier of 23.0 kcal mol^−1^ for formation of PZA means that it should compete against the dissociation.

### 3.7. Reaction pathways for pentazolylphenol and *m*-chloroperbenzoic acid

As shown in figure 9, the pentazolyl oxidizations in PZPol and *m*-CPBA systems are similar to PPZ and *m*-CPBA. The oxidizations of N_α_, N_β_ and N_γ_ of PZPol have the barriers of 56.5, 38.6 and 33.9 kcal mol^−1^, respectively. The dissociation abilities of oxide products for PZPol and *m*-CPBA are the same as those for PPZ and *m*-CPBA. These reactions are not easy to realize.

**Figure 10.**
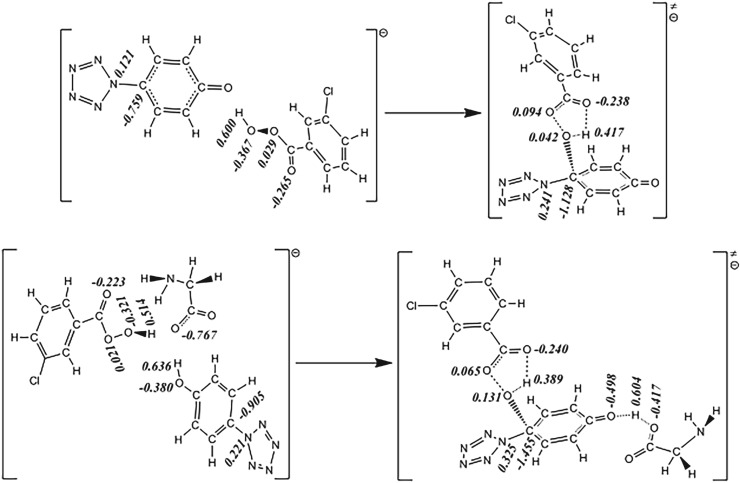
Mulliken charges of active atoms for formations of PZA from reactants to TSs.

The experimental attempt used phenol substance as the starting material [19]. The formation of quinone via the hydroxyl group deprotonation is the possible way to the goal of formation of PZA. The first strategy is the oxygen of *m*-CPBA attacking the C_α_ of PZPol, and then deprotonation. The C_α_ oxidation involves the production of a phenol oxide structure with a barrier of 33.3 kcal mol^−1^. The deprotonations of PZPol oxide can proceed along two different pathways: (1) a stepwise pathway to benzoquinone, dinitrogen and N_3_ radical undergoes the dehydration with *m*-CPBA, the cleavage of peroxyl bond via a MECP, the cleavage of C_α_–N_α_ bond, etc., with an effective barrier of 62.7 kcal mol^−1^ counted to the seven-membered ring TS of dehydration; (2) the glycinate anion absorbs the proton of PZPol oxide and the cleavage of C_α_–N_α_ bond produce benzoquinone and PZA with a barrier of 0.8 kcal mol^−1^. Mulliken analyses indicate that the electrons transfer to C_α_ in both TSs which makes the C_α_–N_α_ bond weak, figure 10. The anionic systems provide the environments for the electrons transferring to pentazole fragments, just as the radical anion mechanism. According to the TS geometries on the active sites, the dehydrations are divided into two mechanisms, i.e. four-membered and seven-membered rings, and the dehydration barrier of the former mechanism is higher than that of the latter mechanism. The second strategy is dehydration in advance, and then C_α_ oxidation, cleavage of peroxyl bond, the cleavage of C_α_–N_α_ bond, etc. The terminal productions are the same as the first strategy. The determinant step is also the seven-membered ring dehydration with a barrier of 63.4 kcal mol^−1^. The third strategy is the concerted mechanism, i.e. the deprotonation is simultaneous with the cleavage of C_α_–N_α_ bond. The tri-molecular reaction among PZPol, *m*-CPBA and glycinate anion produces benzoquinone, glycine and PZA with a barrier of 20.4 kcal mol^−1^ which is higher than the barrier of dinitrogen evolution of 17.5 kcal mol^−1^. Based on the solvent effects shown in table 1, the reaction barriers of PZPolA and *m*-CPBA are decreased by 2.6 kcal mol^−1^ for water, 2.5 kcal mol^−1^ for methanol and 2.6 kcal mol^−1^ for acetonitrile. There are no significant impacts on the competitive reactions. Thus, PZPolA is more feasible than PZPol on PZA formation.

**Table 1.** The crucial activation energy barriers for formations of PZA and dissociations of arylpentazoles in kcal mol^−1^ excluding ZPCs with and without the solvent effects.

	Δ*E*^≠^(B3LYP^a^)				Δ*E*^≠^(RI-B2KPLYP^b^)
transition states	gas	water	methanol	acetonitrile	gas
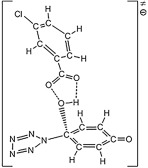	22.2	19.6	19.7	19.6	25.3
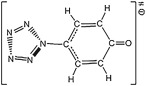	23.1	23.0	23.0	23.0	26.3
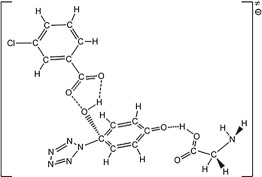	22.3	22.0	22.0	22.0	27.4
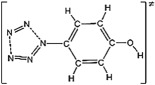	20.2	22.0	21.9	22.0	23.4

^a^Using the 6-311++G** basis set.

^b^At the RI-B2KPLYP/ma-def2-TZVP//B3LYP/6-311++G** level.

### 3.8. Electron and proton transfers in (N_5_)_6_(H_3_O)_3_(NH_4_)_4_Cl

**Figure 11.**
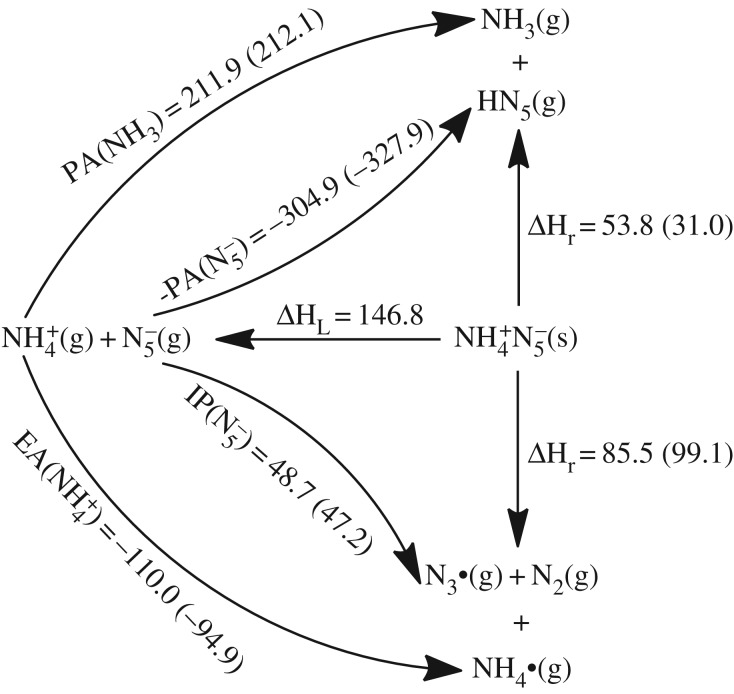
Born–Haber cycles in kcal mol^−1^ for the dissociative reaction of solid NH_4_^+^N_5_^−^. The IP, EA and PA are calculated at the B3LYP/6-311++G** and the CCSD(T)/CBS (in parentheses) levels, and the lattice enthalpy is estimated by an ionic pair volume of 0.104 nm^3^.

Primarily the stability of (N_5_)_6_(H_3_O)_3_(NH_4_)_4_Cl is simplified as the dissociations of two ionic pairs, i.e. NH_4_^+^N_5_^−^ and H_3_O^+^N_5_^−^. The complete contact between H_3_O^+^ and N_5_^−^ partly supports the reasonability for the assumption. Because the *cyclo*-N_5_ radical (removal of one electron of PZA) does not locate at the local minimum point of PES, the dissociation products of N_3_ radical and N_2_ are used for the adiabatic IP calculation. The Born–Haber cycles on NH_4_^+^N_5_^−^ shown in figure 11 indicate the reaction enthalpy of electron transfer of 85.5 kcal mol^−1^ is higher than that of proton transfer of 53.8 kcal mol^−1^, i.e. the proton transfer is dominant. These enthalpies need to be corrected by the entropies. The calculated entropies of NH_3_ and HN_5_ are 46.0 and 63.8 cal mol^−1^ K^−1^, respectively, and the referenced solid entropies of ammonium azide, ammonium nitrate and ammonium hydrogen carbonate are 26.9, 36.1 and 28.9 cal mol^−1^ K^−1^ [60], respectively. If the entropy of ammonium azide is used to estimate the proton transfer reaction free energy of NH_4_^+^N_5_^−^ at 298 K, it is equal to 29.1 kcal mol^−1^. It is thermodynamically stable enough to prevent the proton transfer. Thus, the stability of solid NH_4_^+^N_5_^−^ is decided by the dissociation barrier of PZA.

**Figure 12.**
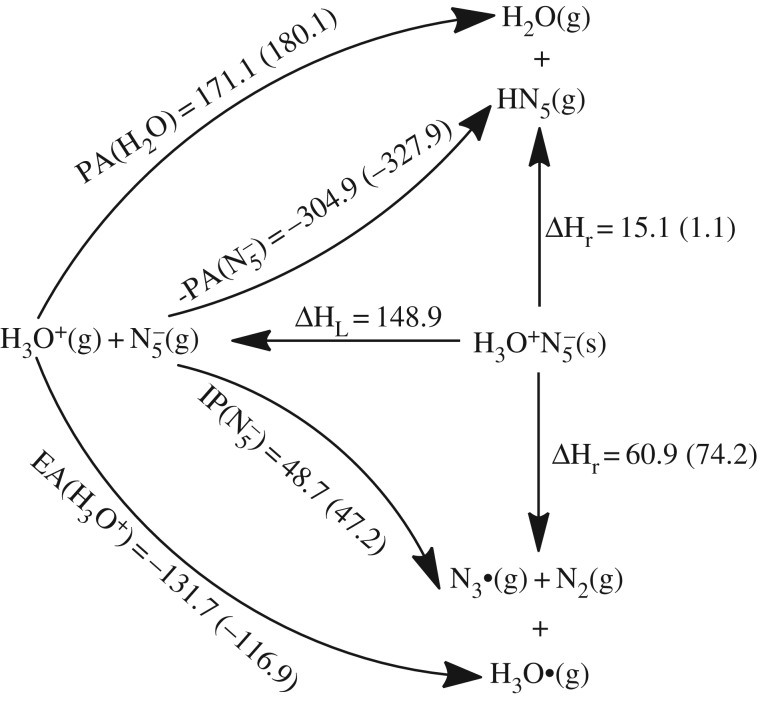
Born–Haber cycles in kcal mol^−1^ for the dissociative reaction of solid H_3_O^+^N_5_^−^. The IP, EA and PA are calculated at the B3LYP/6-311++G** and the CCSD(T)/CBS (in parentheses) levels, and the lattice enthalpy is estimated by an ionic pair volume of 0.110 nm^3^.

The Born–Haber cycles on H_3_O^+^N_5_^−^ shown in figure 12 indicate that the proton transfer is more dominant than the electron transfer thermodynamically. Although the proton transfer enthalpy of 15.1 kcal mol^−1^ at the B3LYP/6-311++G** level is sufficient to overcome the entropy correction, a more accurate value of 1.1 kcal mol^−1^ at the CCSD(T)/CBS level cannot assure an extra barrier for the enhancement of stability of HN_5_. However, in the absence of the entropies of hydronium salts, the calculation of (N_5_)_6_(H_3_O)_3_(NH_4_)_4_Cl in the crystalline state was put forward. Except for the removal of three hydrogen atoms conjugated to each oxygen atom, the initial crystal structure from calculation was analogous to that from experiment [19]. The geometry optimization task was performed without the symmetry constrain and with the constant cell. Then the phonon calculation result deduced the thermodynamic properties. The entropy per (N_5_)_6/7_(H_3_O)_3/7_(NH_4_)_4/7_Cl_1/7_ at 298 K of 33.6 cal mol^−1^ K^−1^ approximately represents that of H_3_O^+^N_5_^−^. Combined with the calculated entropies of H_2_O and HN_5_ of 45.1 and 63.8 cal mol^−1^ K^−1^, respectively, the proton transfer reaction free energies of H_3_O^+^N_5_^−^ are −7.3 and −21.3 kcal mol^−1^, if the reaction enthalpies are calculated at the B3LYP/6-311++G** and the CCSD(T)/CBS levels, respectively. It surveys that the proton transfer in H_3_O^+^N_5_^−^ produces H_2_O and HN_5_ spontaneously. The subsequent reaction for HN_5_ dissociation has the barriers of 19.1 and 21.2 kcal mol^−1^ at the B3LYP/6-311++G** and the CCSD(T)/CBS levels, respectively.

**Figure 13.**
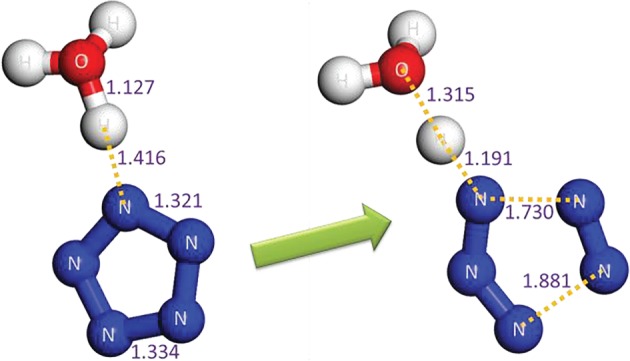
The crucial fragments of the optimized crystalline state of (N_5_)_6_(H_3_O)_3_(NH_4_)_4_Cl for the dissociation from reactant to TS. The distances calculated at the PBE/OTFG_80 level are labelled in Å. The activation energy barrier including ZPC, activation enthalpy barrier at 298 K and activation free energy barrier at 298 K calculated at the PBE/OTFG_80 level are 25.9, 27.5 and 24.7 kcal mol^−1^, respectively. The cell parameters and atomic positions of TS are presented in the electronic supplementary material.

The concerted effect for the proton transfer and dinitrogen evolution in the solid (N_5_)_6_(H_3_O)_3_(NH_4_)_4_Cl is shown in figure 13. The closest non-bond H–N distance in the equilibrium structure is only 1.416 Å. It tends to form H–N covalence bond together with the breakage of PZA, and the dissociation process releases 7.4 kcal mol^−1^ heat. The dissociation barrier calculated at the PBE/OTFG_80 level is 24.7 kcal mol^−1^. To test the reliability of the computational method, a comparative study on the dinitrogen evolution barriers of PPZ excluding ZPCs indicates that the PBE/OTFG_80 method with a barrier of 17.2 kcal mol^−1^ underestimates the barriers by 2.6 and 4.9 kcal mol^−1^ at the B3LYP/6-311++G** and the CCSD(T)/CBS levels, respectively. When the thermal correction to Gibbs free energy at the B3LYP/6-311++G** level is used for the dissociation barrier of PPZ, it is down to 14.2 kcal mol^−1^. Thus, the kinetic stability of the solid (N_5_)_6_(H_3_O)_3_(NH_4_)_4_Cl is better than that of the isolated PPZ.

### 3.9. Stability of M(N_5_)_2_(H_2_O)_4_ (M = Co, Fe and Mn)

**Figure 14.**
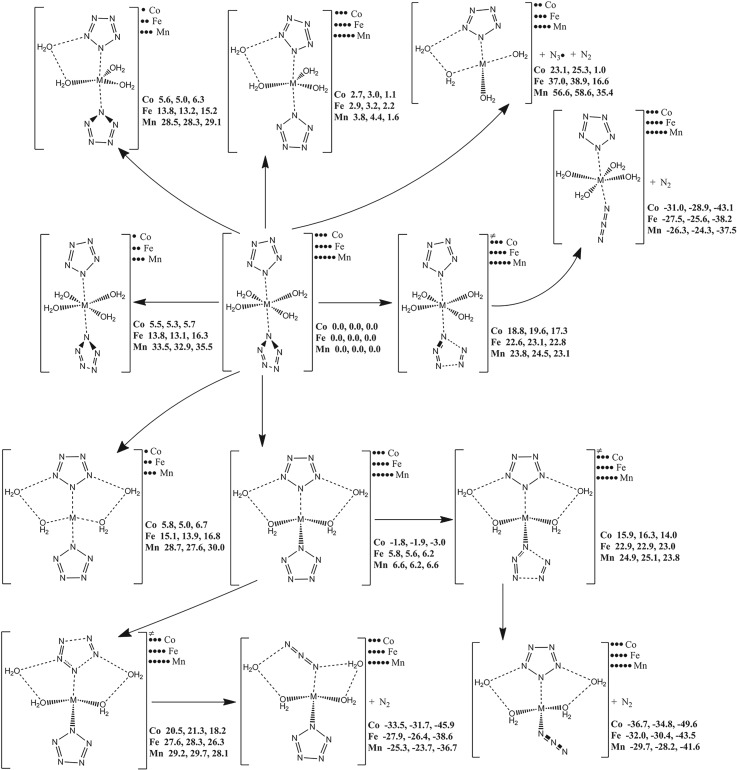
The PES scanning result for M(N_5_)_2_(H_2_O)_4_ (M = Co, Fe and Mn) calculated at the B3LYP/6-311++G**(HNO) + LanL2DZ(CoFeMn) level. The energy (sum of total electronic energy and ZPC at front, enthalpy of 298 K at middle and free energy of 298 K at back) scales are offset to 0 kcal mol^−1^ for M(N_5_)_2_(H_2_O)_4_ as the reference. The crucial bond lengths are labelled in Å.

According to the structure of M(N_5_)_2_(H_2_O)_4_·4H_2_O in the crystalline state [20,23], each metal atom coordinately bonds two pentazolyl groups and four water molecules, and there exists the long-range interaction between the four crystal water molecules in the primitive cell and the coordination compound. Therefore, the stability of isolated state Mn(N_5_)_2_(H_2_O)_4_ can stand for that of crystalline state in a great part. The spin state and coordination must be revealed in the first place. The potential energy scanning result is shown in figure 14. The quartet Co(N_5_)_2_(H_2_O)_4_ imparts the quasi-octahedral structure. Across a Co atom, the two pentazolyl groups are perpendicular to each other. As to the doublet states, the four-coordinated Co(N_5_)_2_(H_2_O)_2_·2H_2_O and the five-coordinated Co(N_5_)_2_(H_2_O)_2_·H_2_O have the same potential energy level as six-coordinated Co(N_5_)_2_(H_2_O)_4_ which lies 5.7 kcal mol^−1^ above its quartet state. It demonstrates that the pentazolyl group is a weak ligand for Co, and the coordination compound imparts the high-spin state. The prediction can be verified by the measurement of magnetic moment. As to the quartet states, the potential energy of the six-coordinated Co(N_5_)_2_(H_2_O)_4_ is lower than that of the five-coordinated Co(N_5_)_2_(H_2_O)_2_·H_2_O by 1.1 kcal mol^−1^, but higher than that of the four-coordinated Co(N_5_)_2_(H_2_O)_2_·2H_2_O by 3.0 kcal mol^−1^. It demonstrates that the binding ability of the coordinate bond of water molecule is inferior to that of the crystal waters at the double sides of the pentazolyl group which is consistent with the space occupancy of the crystal waters in crystalline state. The electron transfer in Co(N_5_)_2_(H_2_O)_4_ produces triplet Co(N_5_)(H_2_O)_3_·H_2_O, N_3_ radical and N_2_ with a free energy change of 1.0 kcal mol^−1^ at 298 K. The slight positive energy is difficult to prevent the subsequent reaction. The dinitrogen evolution of Co(N_5_)_2_(H_2_O)_4_ has a barrier of 17.3 kcal mol^−1^ which is at the same level of PPZ. The dissociation of the exposed pentazolyl group in Co(N_5_)_2_(H_2_O)_2_·2H_2_O has a barrier of 14.0 kcal mol^−1^. But the dissociation of the pentazolyl group confined by the crystal waters has a higher barrier of 18.2 kcal mol^−1^. Thus, the confinement effect of the crystal waters is beneficial to the kinetic stability of Co(N_5_)_2_(H_2_O)_4_·4H_2_O in the crystalline state. The most stable Fe and Mn coordination compounds are quintet Fe(N_5_)_2_(H_2_O)_4_ and sextet Mn(N_5_)_2_(H_2_O)_4_. Their dissociation barriers are 22.8 and 23.1 kcal mol^−1^, respectively. The dissociation barriers of the pentazolyl groups confined by the crystal waters for quintet Fe(N_5_)_2_(H_2_O)_2_·2H_2_O and sextet Mn(N_5_)_2_(H_2_O)_2_·2H_2_O are 26.3 and 28.1 kcal mol^−1^, respectively. Consequently, Fe and Mn coordination compounds are more stable than Co coordination compound.

### 3.10. Stability of isolated pentazolate anion

**Figure 15.**
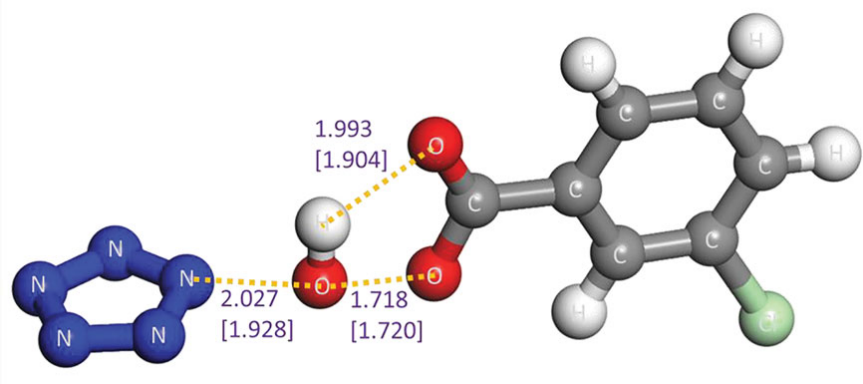
The optimized TS of reaction between PZA and *m*-CPBA. The system possesses one negative charge. The distances calculated at the B3LYP/6-311++G** and RI-B2KPLYP/ma-def2-TZVP (in parenthesis) levels are labelled in Å. The activation energy barrier including ZPC, activation enthalpy barrier at 298 K and activation free energy barrier at 298 K calculated from complex of PZA and *m*-CPBA to TS are 17.1, 16.9 and 19.0 kcal mol^−1^ at the B3LYP/6-311++G** level, respectively, and 22.6, 23.3 and 21.5 kcal mol^−1^ at the RI-B2KPLYP/ma-def2-TZVP level, respectively.

**Figure 16.**
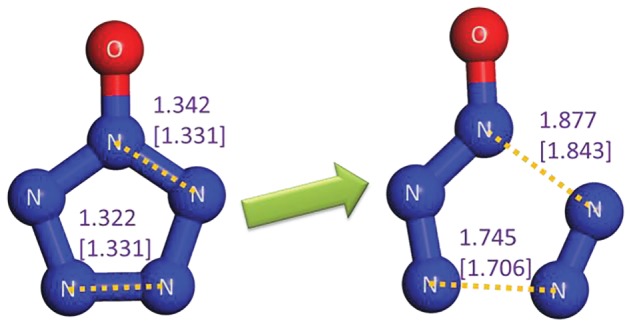
The determinant step for the dissociation of oxo-PZA. The system possesses one negative charge. The distances calculated at the B3LYP/6-311++G** and RI-B2KPLYP/ma-def2-TZVP (in parenthesis) levels are labelled in Å. The activation energy barrier including ZPC, activation enthalpy barrier at 298 K and activation free energy barrier at 298 K from complex of PZA and *m*-CPBA to TS are 25.0, 25.6 and 24.2 kcal mol^−1^ at the B3LYP/6-311++G** level, respectively, and 28.0, 28.5 and 27.4 kcal mol^−1^ at the RI-B2KPLYP/ma-def2-TZVP level, respectively. The activation energy barrier excluding ZPC at the CCSD(T)/CBS level is 33.3 kcal mol^−1^.

**Figure 17.**
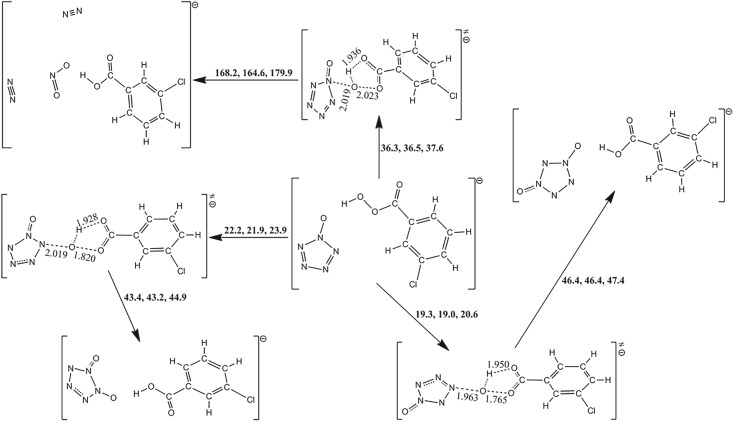
The PES scanning result for oxo-PZA and *m*-CPBA calculated at the B3LYP/6-311++G** level. The activation energy barriers (sum of total electronic energy and ZPC at front, enthalpy of 298 K at middle and free energy of 298 K at back) are in kcal mol^−1^. The crucial bond lengths are labelled in Å.

**Figure 18.**
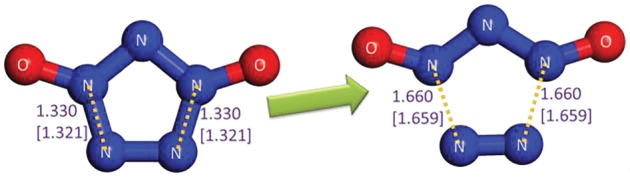
The process for dinitrogen evolution from 1,3-oxo-PZA to TS. The system possesses one negative charge. The distances calculated at the B3LYP/6-311++G** and RI-B2KPLYP/ma-def2-TZVP (in parenthesis) levels are labelled in Å. The activation energy barrier including ZPC, activation enthalpy barrier at 298 K and activation free energy barrier at 298 K from complex of PZA and *m*-CPBA to TS are 23.1, 23.5 and 22.7 kcal mol^−1^ at the B3LYP/6-311++G** level, respectively, and 28.0, 28.4 and 27.6 kcal mol^−1^ at the RI-B2KPLYP/ma-def2-TZVP level, respectively. The activation energy barrier excluding ZPC at the CCSD(T)/CBS level is 32.7 kcal mol^−1^.

The stability of a substance generally hinges on the two aspects, i.e. the decomposability of itself and the reactivity with other substances in the system. Without a doubt, PZA has a sufficient energy barrier without ZPC of above 25 kcal mol^−1^ for the dinitrogen evolution according to our (26.3, 28.6 and 28.7 kcal mol^−1^ at the B3LYP/6-311++G**, the RI-B2KPLYP/ma-def2-TZVP and the CCSD(T)/CBS levels, respectively) and other calculations [4–6]. As to the reactivity of PZA, a similar TS as the reaction between oxo-PPZ and *m*-CPBA is found. As shown in figure 15, the TS involves the breakage of peroxyl bond, proton transfer to original carbonyl group and oxygen atom approach to pentazolyl group that indicates the formation of oxo-PZA. The pathway has a low barrier of 19.0 kcal mol^−1^. Based on the PES scan around oxo-PZA, the optimum dissociation pathway involves the production of N_3_O^−^ and N_2_ in the first step, and the production of triplet NO^−^ in the second step. The re-calculation of published result [62] at the B3LYP/6-311++G** level is presented in electronic supplementary material, figure S2, and the dinitrogen evolution pathway of oxo-PZA is presented in figure 16. The determinant barrier concerned with the stability of oxo-PZA is 24.2 kcal mol^−1^, which is comparable to that of PZA. The reactivity of oxo-PZA should be considered for further calculation. The reaction pathways for oxo-PZA and *m*-CPBA shown in figure 17 indicate that the oxygen atom of *m*-CPBA can attack the three different sites of oxo-PZA. The barriers for the formations of nitrite, 1,2-oxo-PZA and 1,3-oxo-PZA are 37.6, 23.9 and 20.6 kcal mol^−1^, respectively. Therefore 1,3-oxo-PZA is the dominant production. The formation barrier of 1,3-oxo-PZA is also re-calculated at the RI-B2KPLYP/ma-def2-TZVP level, and the barrier is 25.8 kcal mol^−1^. The dissociation of 1,3-oxo-PZA shown in figure 18 has a barrier of 22.7 kcal mol^−1^. Compared with the computational results at the RI-B2KPLYP/ma-def2-TZVP and CCSD(T)/CBS levels, the calculation at the B3LYP/6-311++G** level underestimates the barrier to a certain degree. Consequently, the stabilities of oxo-PZA and 1,3-oxo-PZA are perhaps better. The synthesizabilities should be validated by further experimental studies.

## 4. Conclusion

The synthetic and stable mechanisms for PZA have been credibly revealed by the quantum chemistry calculations on PESs. Although the mechanisms are not so perfect within the constraints of theoretical methods and computing resources, e.g. there is no interpretation of the source of ammonium, the results presented in this paper have a valuable reference for illuminating the roads of theoretical and experimental studies on PZA. With the investigation above, we can draw conclusions as follows:

(1) Both singlet and triplet PPZ cannot produce PZA solely at ambient condition, because the processes are subject to the reaction thermodynamics. At high-energy condition, excitation of PPZ and deprotonation of PPZ-RA probably occur before the cleavage of C–N bond of PPZ.
(2) A new proton transfer pathway for the radical anion mechanism of PZA formation was found, but the pathway for the direct cleavage of C–N bond of PPZ-RA is still kinetically dominant. According to the verification of various methods, the barrier (about 25 kcal mol^−1^) of PZA formation is close to that of dinitrogen evolution. Typically, the former is slightly lower than the latter at the CCSD(T)/CBS level. Even so, the ionic pair of sodium PPZ should be formed at a very low temperature, which is not beneficial in the reactivity. The endothermic process is another possible difficulty. The selection of other arylpentazole radical anions with large IPs should be focused in the subsequent experimental work. Moreover, due to the same level barriers for PZA formation and dissociation, the vigorous reaction condition is likely to cause the dinitrogen evolution.
(3) The direct cleavage of C–N bond of singlet PZPolA for PZA formation undergoes a MECP with an ultra-high effective barrier. The cleavage reaction between PZPolA and *m*-CPBA has a moderate barrier of about 20 kcal mol^−1^ which competes with the dinitrogen evolution of PZPolA. Although the reaction between PPZ and *m*-CPBA cannot form PZA, the reaction between PZPol and *m*-CPBA assisted by the proton absorption of glycinate anion can produce PZA with a barrier of about 20 kcal mol^−1^. If the solvent effects are considered, the PZA formation starting with PZPolA is more feasible than that starting with PZPol.
(4) The ionic pair of H_3_O^+^N_5_^−^ is unstable. The proton can transfer to gaseous PZA without a barrier at high temperature, and then the hydrogenated pentazole exhausts dinitrogen, which is easier than PZA dissociation. The proton transfer in solid (N_5_)_6_(H_3_O)_3_(NH_4_)_4_Cl can lead to concerted dissociation. The stability of solid (N_5_)_6_(H_3_O)_3_(NH_4_)_4_Cl is better than that of gaseous PPZ.
(5) The quartet Co(N_5_)_2_(H_2_O)_4_, quintet Fe(N_5_)_2_(H_2_O)_4_ and sextet Mn(N_5_)_2_(H_2_O)_4_ are more thermodynamically stable than their low-spin states, which needs to be validated by the magnetic measurement. The Co coordination compound can spontaneously convert to the doublet Co(N_5_)(H_2_O)_3_·H_2_O at the high temperature. The quartet Co(N_5_)_2_(H_2_O)_2_·2H_2_O is the most thermodynamically stable among Co(N_5_)_2_(H_2_O)_n_· (4-n)H_2_O. The exposed pentazolyl group in Co(N_5_)_2_(H_2_O)_2_·2H_2_O is less kinetically stable than that confined by the crystal waters. If the confined effect is taken into account, the coordinate compound with the pentazolyl groups is still less kinetically stable than gaseous PZA. According to the computational result of similar reactions, Fe(N_5_)_2_(H_2_O)_4_ and Mn(N_5_)_2_(H_2_O)_4_ are more stable than Co(N_5_)_2_(H_2_O)_4_.
(6) PZA can be persistently oxidized by *m*-CPBA to oxo-PZA and 1,3-oxo-PZA. The reactivity of *m*-CPBA with PZA is almost the same as those with PZPolA and PZPol. The selectivity of *m*-CPBA and synthetic method of oxo-PZA must be explained by both experimental and theoretical efforts in the future.

## Supplementary Material

Geometries

## Supplementary Material

Computational test

## Supplementary Material

Supplementary solvent effect

## Supplementary Material

Supplementary potential energy surfaces

## Supplementary Material

Crystallographic information file

## Data accessibility

Electronic supplementary material is available on Figshare. The *xyz* coordinates for the optimized geometries and their total energies, the computational test of different quantum chemistry methods on dissociation barriers, the solvent effect for radical anion mechanism, the PES scanning result for *m*-chloroperbenzoic acid (*m*-CPBA), the PES scanning result for oxo-phenylpentazole (oxo-PPZ) and the crystallographic information file for the TS of the solid (N_5_)_6_(H_3_O)_3_(NH_4_)_4_Cl dissociation.
