# Supplementary material for: Roads to pentazolate anion: a theoretical insight
Source: R Soc Open Sci. 2018 May 23;5(5):172269. doi: 10.1098/rsos.172269 (PMC5990749; doi:10.1098/rsos.172269)
Supplement: Geometries [file rsos172269supp1.docx]

**Electronic supplementary material of “Roads to pentazolate anion: A theoretical insight”**

The xyz coordinates of geometries in Fig. 2:

­­­___________________________________________________________


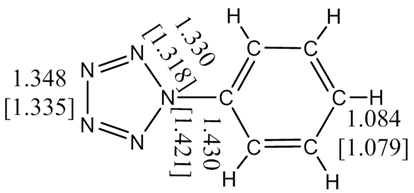


B3LYP/6-311++G**

Total electronic energy = -505.447772 Ha

Total electronic energy solvent (THF) = -505.455065 Ha

Total electronic energy CCSD(T)/cc-pVTZ = -504.471239 Ha

Total electronic energy HF/cc-pVTZ = -502.425614 Ha

Total electronic energy CCSD(T)/cc-pVDZ = -503.999025 Ha

Total electronic energy HF/cc-pVDZ =-502.302323 Ha

Total electronic energy CCSD(T)/CBS = -504.724721 Ha

Zero-point correction = 0.114726 Ha

Thermal correction to enthalpy = 0.123434 Ha

Thermal correction to free energy = 0.080359 Ha

C 2.455602 1.206565 0.000009

C 1.064239 1.217340 0.000008

C 0.385140 0.000001 -0.000005

C 1.064235 -1.217339 -0.000016

C 2.455599 -1.206569 -0.000015

C 3.153936 -0.000003 -0.000003

H 2.992792 2.147435 0.000020

H 0.511242 2.146696 0.000018

H 0.511235 -2.146694 -0.000025

H 2.992785 -2.147440 -0.000023

H 4.237495 -0.000005 -0.000001

N -1.044529 0.000003 -0.000002

N -1.795543 -1.097517 0.000070

N -3.019203 -0.673852 -0.000029

N -3.019195 0.673848 -0.000032

N -1.795537 1.097524 0.000014

RI-B2KPLYP/ma-def2-TZVP

Total electronic energy = -505.027318

Zero-point correction = 0.116524 Ha

Thermal correction to enthalpy = 0.125077 Ha

Thermal correction to free energy = 0.084033 Ha

C -2.443749 1.203484 -0.001969

C -1.058401 1.212824 -0.005563

C -0.387928 -0.000151 -0.000722

C -1.060902 -1.211797 0.007612

C -2.446333 -1.199387 0.010963

C -3.139001 0.002771 0.006222

H -2.978788 2.140477 -0.005640

H -0.503396 2.136345 -0.012245

H -0.508053 -2.136592 0.011496

H -2.983516 -2.135129 0.017703

H -4.218110 0.003804 0.008766

N 1.032798 -0.001908 -0.004559

N 1.771887 -1.093687 -0.002687

N 2.996264 -0.672260 -0.007302

N 2.997979 0.663210 -0.011986

N 1.774766 1.088005 -0.010094


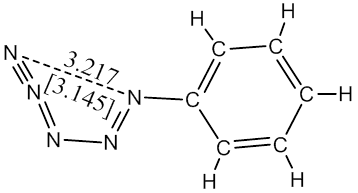


B3LYP/6-311++G**

Total electronic energy = -505.404920 Ha

Zero-point correction = 0.111273 Ha

Thermal correction to enthalpy = 0.121286 Ha

Thermal correction to free energy = 0.075684 Ha

C 2.641084 1.333032 -0.000003

C 1.250583 1.266554 0.000019

C 0.609313 0.023878 0.000034

C 1.364724 -1.158754 0.000025

C 2.750768 -1.081628 0.000003

C 3.393310 0.160429 -0.000011

H 3.135006 2.297749 -0.000014

H 0.643597 2.163993 0.000025

H 0.860348 -2.116039 0.000038

H 3.337700 -1.993164 -0.000002

H 4.476230 0.209409 -0.000028

N -0.807747 0.074273 0.000057

N -1.384803 -1.029474 0.000074

N -2.783060 -0.966047 0.000016

N -3.266880 0.200577 -0.000076

N -3.830592 1.174527 -0.000131

RI-B2KPLYP/ma-def2-TZVP

Total electronic energy = -504.975469 Ha

Zero-point correction = 0.112899 Ha

Thermal correction to enthalpy = 0.122714 Ha

Thermal correction to free energy = 0.07896141 Ha

C -2.626054 1.328125 0.000023

C -1.241146 1.262779 0.000113

C -0.605692 0.027482 0.000097

C -1.351836 -1.150586 -0.000006

C -2.732330 -1.074818 -0.000100

C -3.373075 0.160206 -0.000087

H -3.118993 2.288394 0.000037

H -0.635688 2.156370 0.000198

H -0.848096 -2.103608 -0.000015

H -3.316323 -1.982857 -0.000183

H -4.451573 0.207347 -0.000158

N 0.807722 0.076991 0.000193

N 1.369063 -1.030101 0.000191

N 2.760182 -0.979266 0.000169

N 3.223586 0.190418 -0.000114

N 3.752110 1.182073 -0.000385


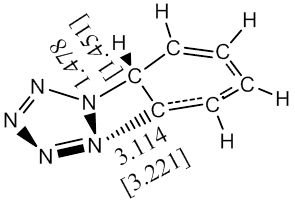


B3LYP/6-311++G**

Total electronic energy = -505.295726 Ha

Zero-point correction = 0.110779 Ha

Thermal correction to enthalpy = 0.120067 Ha

Thermal correction to free energy = 0.075882 Ha

C -1.073576 -1.317019 -0.112224

C -0.385946 -0.117247 -0.600371

C -1.073411 1.212318 -0.459334

C -2.307393 1.224675 0.087874

C -2.970055 0.001784 0.431271

C -2.451041 -1.238620 0.071169

H -3.894395 0.058194 1.000195

H -3.043482 -2.137446 0.214810

H -2.840821 2.159420 0.220266

H -0.627227 2.091923 -0.911216

N 1.028564 -0.054938 -0.177558

N 2.057044 -0.177282 -1.003410

N 3.107376 -0.072727 -0.251117

N 2.715096 0.114074 1.025329

N 1.418984 0.126325 1.072118

H -0.315006 -0.315593 -1.691909

RI-B2KPLYP/ma-def2-TZVP

Total electronic energy = -504.867314 Ha

Zero-point correction = 0.112630 Ha

Thermal correction to enthalpy = 0.121000 Ha

Thermal correction to free energy = 0.08048574 Ha

C -1.004413 -1.343473 -0.211984

C -0.377582 -0.033262 -0.399736

C -1.024038 1.241013 -0.024316

C -2.340660 1.213235 0.254676

C -3.032149 -0.034814 0.234261

C -2.411007 -1.223953 -0.054661

H -4.085983 -0.031540 0.482220

H -3.003898 -2.128089 -0.025027

H -2.875733 2.117979 0.496796

H -0.455209 2.159035 -0.052951

N 1.056221 -0.009797 -0.177724

N 1.959630 -0.136451 -1.122974

N 3.097325 -0.094366 -0.503061

N 2.875170 0.057961 0.804742

N 1.595680 0.110918 1.012675

H -0.490853 -0.095299 -1.512115


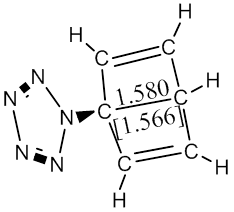


B3LYP/6-311++G**

Total electronic energy = -505.312849 Ha

Zero-point correction = 0.111579 Ha

Thermal correction to enthalpy = 0.120503 Ha

Thermal correction to free energy = 0.077186 Ha

C 1.293469 -1.321046 0.712835

C 2.187368 -1.306887 -0.280221

C 1.678026 -0.000036 -0.892437

C 2.187338 1.306887 -0.280352

C 1.293448 1.321117 0.712711

C 0.645424 0.000010 0.303133

H 1.099747 -1.971669 1.555066

H 3.029030 -1.939168 -0.529886

H 3.028979 1.939168 -0.530086

H 1.099713 1.971813 1.554883

N -0.788898 0.000000 0.110811

N -1.529328 -1.097768 0.001460

N -2.739425 -0.673856 -0.184406

N -2.739451 0.673827 -0.184301

N -1.529365 1.097752 0.001630

H 1.317359 -0.000094 -1.920339

RI-B2KPLYP/ma-def2-TZVP

Total electronic energy = -504.895722 Ha

Zero-point correction = 0.113898 Ha

Thermal correction to enthalpy = 0.122641 Ha

Thermal correction to free energy = 0.081094 Ha

C -1.288779 -1.310703 -0.715322

C -2.172543 -1.296439 0.284255

C -1.657699 -0.000035 0.886565

C -2.172519 1.296438 0.284384

C -1.288755 1.310785 -0.715193

C -0.645687 0.000015 -0.309116

H -1.104961 -1.957945 -1.557065

H -3.009965 -1.925301 0.538562

H -3.009929 1.925291 0.538753

H -1.104926 1.958104 -1.556875

N 0.776585 -0.000003 -0.119512

N 1.505508 -1.091319 -0.001752

N 2.714437 -0.667777 0.197252

N 2.714461 0.667732 0.197195

N 1.505545 1.091299 -0.001840

H -1.283793 -0.000090 1.905035


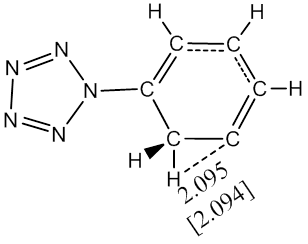


B3LYP/6-311++G**

Total electronic energy = -505.303774 Ha

Zero-point correction = 0.110177 Ha

Thermal correction to enthalpy = 0.119606 Ha

Thermal correction to free energy = 0.075740 Ha

C 2.496123 1.134770 -0.092897

C 1.088930 1.166726 -0.269917

C 0.378892 0.006843 -0.186718

C 1.097591 -1.297288 -0.189430

C 2.555525 -1.224446 0.115283

C 3.142579 -0.007931 0.400704

H 3.063257 2.031679 -0.328089

H 0.587758 2.118358 -0.393522

H 0.627538 -2.020868 0.492293

H 4.154366 0.054756 0.789240

N -1.023283 0.024569 -0.028601

N -1.788581 -1.069420 -0.077552

N -3.000564 -0.646334 0.061841

N -2.988080 0.702079 0.184502

N -1.769307 1.122984 0.137450

H 0.997943 -1.793113 -1.165568

RI-B2KPLYP/ma-def2-TZVP

Total electronic energy = -504.873602 Ha

Zero-point correction = 0.112311 Ha

Thermal correction to enthalpy = 0.121577 Ha

Thermal correction to free energy = 0.079193 Ha

C 2.482405 1.123688 -0.120816

C 1.089549 1.149549 -0.317255

C 0.377839 -0.004119 -0.229047

C 1.092850 -1.297752 -0.237599

C 2.518336 -1.160341 0.147888

C 3.099750 0.011969 0.491971

H 3.074139 1.966260 -0.455445

H 0.592941 2.094624 -0.475057

H 0.616470 -2.024872 0.428141

H 4.070375 0.079496 0.959978

N -1.011875 0.019906 -0.023145

N -1.783140 -1.047144 -0.149503

N -2.985126 -0.624093 0.068244

N -2.947903 0.692421 0.312824

N -1.722253 1.099336 0.266579

H 1.056332 -1.775565 -1.218746


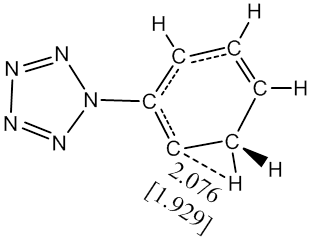


B3LYP/6-311++G**

Total electronic energy = - 505.304926 Ha

Zero-point correction = 0.109823 Ha

Thermal correction to enthalpy = 0.119452 Ha

Thermal correction to free energy = 0.074661 Ha

C 2.407782 1.240418 -0.152898

C 1.003040 1.103664 -0.350620

C 0.360227 -0.092539 -0.065043

C 1.022110 -1.313504 0.059894

C 2.496762 -1.186475 0.164698

C 3.146768 0.147765 0.164231

H 2.854194 2.223412 -0.242739

H 0.435942 1.956431 -0.712332

H 4.198408 0.236744 0.419712

N -1.080680 -0.060172 -0.015000

N -1.893859 -1.056226 -0.350304

N -3.088077 -0.597888 -0.149697

N -3.009154 0.671839 0.303583

N -1.763900 1.009467 0.389877

H 2.831546 -1.788566 1.027062

H 2.909475 -1.793130 -0.662488

RI-B2KPLYP/ma-def2-TZVP

Total electronic energy = -504.876483 Ha

Zero-point correction = 0.112146 Ha

Thermal correction to enthalpy = 0.121491 Ha

Thermal correction to free energy = 0.078725 Ha

C 2.412920 1.236324 -0.160571

C 1.001480 1.117732 -0.231146

C 0.375044 -0.079720 0.019275

C 1.011211 -1.283127 0.368969

C 2.473614 -1.156253 0.298977

C 3.149503 0.137155 0.114040

H 2.869793 2.198016 -0.332115

H 0.412429 1.991175 -0.476594

H 4.228080 0.189207 0.166527

N -1.057121 -0.060598 -0.011819

N -1.814021 -1.061495 -0.412446

N -3.028244 -0.625052 -0.306607

N -3.006842 0.631444 0.153972

N -1.777905 0.989451 0.337674

H 2.999982 -1.829854 0.974809

H 2.580659 -1.693166 -0.675009


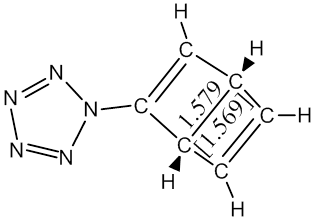


B3LYP/6-311++G**

Total electronic energy = -505.317265 Ha

Zero-point correction = 0.111949 Ha

Thermal correction to enthalpy = 0.120818 Ha

Thermal correction to free energy = 0.077997 Ha

C 0.323132 0.359374 0.267333

C 1.103323 1.429028 0.077201

C 2.366958 0.648042 0.440220

C 3.011360 -0.184518 -0.672697

C 2.211880 -1.241111 -0.492506

C 1.426621 -0.602048 0.654457

H 0.927128 2.436483 -0.272180

H 3.800848 0.047865 -1.376260

H 2.100995 -2.197145 -0.987613

N -1.046586 0.122445 0.081313

N -1.932873 1.027149 -0.337352

N -3.064172 0.402394 -0.362676

N -2.877347 -0.875785 0.036466

N -1.627337 -1.054751 0.314650

H 2.989622 1.022802 1.251795

H 1.359966 -1.112762 1.613398

RI-B2KPLYP/ma-def2-TZVP

Total electronic energy = -504.898602 Ha

Zero-point correction = 0.114282 Ha

Thermal correction to enthalpy = 0.122991 Ha

Thermal correction to free energy = 0.081574 Ha

C 0.329696 0.361768 0.273855

C 1.107883 1.427033 0.079493

C 2.359029 0.644446 0.441315

C 2.987645 -0.181455 -0.670373

C 2.186015 -1.232575 -0.485923

C 1.420775 -0.594624 0.659588

H 0.932906 2.428135 -0.275965

H 3.766937 0.047163 -1.379681

H 2.066668 -2.182792 -0.980530

N -1.032354 0.121990 0.083192

N -1.910228 1.019838 -0.326846

N -3.038103 0.388336 -0.364466

N -2.846405 -0.880318 0.018275

N -1.595823 -1.052865 0.300541

H 2.983868 1.013970 1.247148

H 1.355007 -1.100589 1.615924


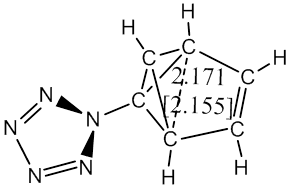


B3LYP/6-311++G**

Total electronic energy = -505.313933 Ha

Zero-point correction = 0.111958 Ha

Thermal correction to enthalpy = 0.120660 Ha

Thermal correction to free energy = 0.078085 Ha

C -1.529757 -0.234254 -1.085551

C -1.288600 -1.292196 -0.001745

C -0.447771 -0.132065 -0.000098

C -1.530037 -0.237374 1.084981

C -2.690016 0.624422 0.670115

C -2.689852 0.626266 -0.668603

H -1.250369 -0.444137 2.109949

H -3.352182 1.142982 1.347369

H -3.351758 1.146750 -1.344632

N 0.954005 0.016467 0.000154

N 1.829303 -0.995094 -0.000469

N 2.990692 -0.438763 -0.000088

N 2.838252 0.911409 0.000624

N 1.581177 1.194259 0.000761

H -1.249839 -0.438344 -2.110994

H -1.093662 -2.353997 -0.003160

RI-B2KPLYP/ma-def2-TZVP

Total electronic energy = -504.899119 Ha

Zero-point correction = 0.114329 Ha

Thermal correction to enthalpy = 0.122859 Ha

Thermal correction to free energy = 0.081684 Ha

C -1.522083 -0.231460 -1.077945

C -1.284668 -1.282889 -0.001710

C -0.451535 -0.124662 -0.000147

C -1.522212 -0.234293 1.077251

C -2.678429 0.619563 0.668297

C -2.678365 0.621308 -0.666891

H -1.237920 -0.441064 2.096566

H -3.340099 1.134076 1.342045

H -3.339964 1.137579 -1.339362

N 0.941965 0.018728 0.000104

N 1.801036 -0.990463 -0.000310

N 2.966113 -0.438766 -0.000915

N 2.821021 0.897337 0.001334

N 1.563520 1.185385 0.001145

H -1.237651 -0.435541 -2.097764

H -1.081142 -2.338507 -0.003085


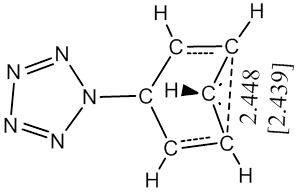


B3LYP/6-311++G**

Total electronic energy = -505.281035 Ha

Zero-point correction = 0.110325 Ha

Thermal correction to enthalpy = 0.119555 Ha

Thermal correction to free energy = 0.076302 Ha

C 0.290794 2.481072 1.224381

C 0.290794 1.095513 1.278743

C 0.053318 0.459297 0.000000

C 0.290794 1.095513 -1.278743

C 0.290794 2.481072 -1.224381

C -0.407578 2.730852 0.000000

H 0.960382 3.120446 1.786950

H 0.659694 0.503898 2.110000

H 0.659694 0.503898 -2.110000

H 0.960382 3.120446 -1.786950

H -1.368740 2.213699 0.000000

N -0.098176 -0.953874 0.000000

N -0.167263 -1.707913 -1.100292

N -0.264000 -2.923886 -0.676663

N -0.264000 -2.923886 0.676663

N -0.167263 -1.707913 1.100292

RI-B2KPLYP/ma-def2-TZVP

Total electronic energy = -504.858944 Ha

Zero-point correction = 0.112480 Ha

Thermal correction to enthalpy = 0.121551 Ha

Thermal correction to free energy = 0.079654 Ha

C 0.290563 2.470142 1.219502

C 0.304324 1.091067 1.275201

C 0.059964 0.465994 0.000004

C 0.304378 1.091075 -1.275189

C 0.290603 2.470147 -1.219500

C -0.413275 2.696889 -0.000005

H 0.943751 3.122444 1.777376

H 0.677498 0.498894 2.098755

H 0.677554 0.498897 -2.098738

H 0.943756 3.122463 -1.777394

H -1.357946 2.157732 -0.000017

N -0.100489 -0.939176 0.000002

N -0.173190 -1.681583 -1.093299

N -0.277484 -2.897567 -0.669954

N -0.277370 -2.897579 0.669954

N -0.173013 -1.681602 1.093302


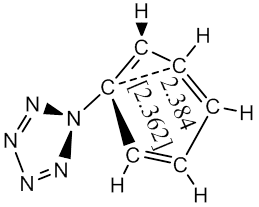


B3LYP/6-311++G**

Total electronic energy = -505.284793 Ha

Zero-point correction = 0.111110 Ha

Thermal correction to enthalpy = 0.120243 Ha

Thermal correction to free energy = 0.077158 Ha

C 1.630414 -1.418501 0.383338

C 2.095517 -0.337380 1.061848

C 2.034031 1.034363 0.482054

C 1.294637 1.350855 -0.617020

C 0.473274 0.155485 -0.984213

C 1.285564 -0.934129 -0.985017

H 1.695641 -2.438169 0.741421

H 2.549977 -0.450867 2.041436

H 1.169370 2.361986 -0.984736

H 2.185286 -0.772546 -1.583631

N -0.796855 0.069377 -0.347302

N -1.539892 -1.040906 -0.313993

N -2.637413 -0.695528 0.274355

N -2.583437 0.619606 0.583174

N -1.448235 1.100731 0.193037

H 2.559933 1.822471 1.014674

RI-B2KPLYP/ma-def2-TZVP

Total electronic energy = -504.862989 Ha

Zero-point correction = 0.113359 Ha

Thermal correction to enthalpy = 0.122286 Ha

Thermal correction to free energy = 0.080749 Ha

C 0.345610 2.761430 0.767458

C 0.884527 1.529865 0.595445

C 0.463852 0.641550 -0.513729

C -0.260880 1.072909 -1.577758

C -0.374741 2.549860 -1.472184

C -0.763522 2.860408 -0.211045

H 0.580814 3.415332 1.592035

H 1.622892 1.151322 1.289142

H -0.484719 0.459183 -2.436245

H -1.637276 2.293130 0.102519

N 0.711655 3.272897 -2.025864

N 0.953734 4.546628 -1.760111

N 1.988347 4.846596 -2.475961

N 2.360401 3.772583 -3.183988

N 1.564320 2.789120 -2.910898

H 0.818623 -0.380744 -0.492644


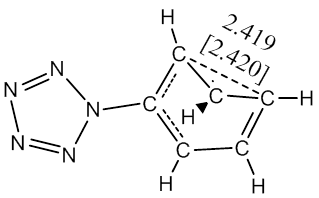


B3LYP/6-311++G**

Total electronic energy = -505.281130 Ha

Zero-point correction = 0.110648 Ha

Thermal correction to enthalpy = 0.119963 Ha

Thermal correction to free energy = 0.076305 Ha

C -3.199216 -0.079362 -0.323985

C -2.535280 1.096399 -0.073785

C -1.133433 1.037380 0.353005

C -0.363791 -0.121007 0.191430

C -1.098865 -1.277387 -0.239823

C -2.334355 -1.058580 0.343012

H -4.085237 -0.195256 -0.933527

H -2.959050 2.069510 -0.304424

H -2.234686 -0.801442 1.399345

N 1.051974 -0.026594 0.055853

N 1.868779 -1.075462 0.140891

N 3.058667 -0.593074 -0.015324

N 2.976561 0.744946 -0.182239

N 1.733777 1.101135 -0.146357

H -0.634083 1.967242 0.605374

H -0.925610 -1.681367 -1.235667

RI-B2KPLYP/ma-def2-TZVP

Total electronic energy = -504.858259 Ha

Zero-point correction = 0.112805 Ha

Thermal correction to enthalpy = 0.121936 Ha

Thermal correction to free energy = 0.079762 Ha

C -3.178229 -0.075799 -0.342591

C -2.522207 1.100534 -0.079970

C -1.143262 1.026919 0.376708

C -0.375395 -0.133504 0.212505

C -1.089300 -1.289488 -0.195726

C -2.334805 -1.027268 0.357166

H -4.014366 -0.206081 -1.009104

H -2.924652 2.070680 -0.338279

H -2.237801 -0.731059 1.398718

N 1.030346 -0.027667 0.059252

N 1.844594 -1.065492 0.109874

N 3.027608 -0.573092 -0.072582

N 2.932560 0.752621 -0.221428

N 1.687718 1.099097 -0.147227

H -0.640891 1.945951 0.646066

H -0.879942 -1.768935 -1.143608


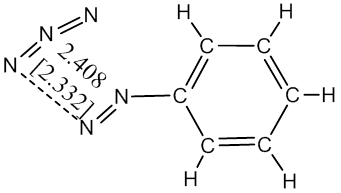


B3LYP/6-311++G**

Total electronic energy = -505.395273 Ha

Zero-point correction = 0.111093 Ha

Thermal correction to enthalpy = 0.121497 Ha

Thermal correction to free energy = 0.075283 Ha

C 2.276031 1.383725 -0.000067

C 0.911806 1.106032 -0.000091

C 0.513933 -0.225713 -0.000010

C 1.426093 -1.285580 0.000029

C 2.781946 -0.985690 0.000055

C 3.206154 0.345289 0.000014

H 2.610046 2.414084 -0.000112

H 0.158550 1.881036 -0.000152

H 1.071904 -2.308684 0.000062

H 3.507581 -1.790190 0.000112

H 4.266292 0.570615 0.000027

N -0.883181 -0.536358 -0.000036

N -1.563885 -1.453961 -0.000011

N -3.695185 -0.334036 -0.000144

N -2.989034 0.595485 0.000088

N -2.055879 1.329550 0.000173

RI-B2KPLYP/ma-def2-TZVP

Total electronic energy = -504.970791 Ha

Zero-point correction = 0.112666 Ha

Thermal correction to enthalpy = 0.122823 Ha

Thermal correction to free energy = 0.078466 Ha

C 2.265639 1.380194 -0.000046

C 0.905250 1.113759 -0.000055

C 0.506056 -0.209846 -0.000013

C 1.404625 -1.271036 0.000035

C 2.756328 -0.981542 0.000043

C 3.185541 0.341077 0.000002

H 2.604831 2.404274 -0.000078

H 0.159349 1.890123 -0.000089

H 1.041861 -2.287003 0.000066

H 3.474581 -1.786453 0.000081

H 4.242381 0.560303 0.000009

N -0.882383 -0.517228 -0.000025

N -1.556067 -1.439936 -0.000005

N -3.627331 -0.368198 -0.000032

N -2.937443 0.572771 0.000040

N -2.000045 1.304345 0.000001


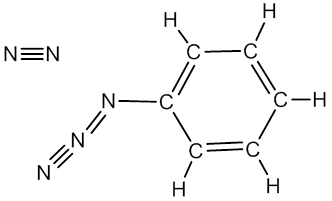


B3LYP/6-311++G**

Total electronic energy = -505.503169 Ha

Zero-point correction = 0.108910 Ha

Thermal correction to enthalpy = 0.121311 Ha

Thermal correction to free energy = 0.064323 Ha

C 2.984206 0.672213 0.002579

C 1.636237 1.022080 0.001368

C 0.662328 0.017492 -0.001067

C 1.042429 -1.327837 -0.002281

C 2.391689 -1.663190 -0.001054

C 3.368984 -0.667339 0.001384

H 3.735305 1.453950 0.004465

H 1.348759 2.067754 0.002322

H 0.273902 -2.091013 -0.004173

H 2.680822 -2.707960 -0.002004

H 4.419451 -0.932869 0.002336

N -0.735634 0.272251 -0.002470

N -4.478890 -1.627198 0.001942

N -4.195461 -0.568965 0.001761

N -1.606734 2.466391 -0.000909

N -1.122349 1.441754 -0.001542

RI-B2KPLYP/ma-def2-TZVP

Total electronic energy = -505.082666 Ha

Zero-point correction = 0.109755 Ha

Thermal correction to enthalpy = 0.119385 Ha

Thermal correction to free energy = 0.074353 Ha

C -2.919991 -0.506883 0.001183

C -1.610042 -0.962275 -0.003475

C -0.566489 -0.042472 -0.004425

C -0.831831 1.321958 -0.001354

C -2.143742 1.763345 0.003442

C -3.193090 0.853002 0.004822

H -3.728552 -1.222134 0.001878

H -1.405912 -2.023536 -0.006555

H -0.006163 2.016757 -0.002237

H -2.346988 2.823629 0.006420

H -4.214572 1.200850 0.008773

N 0.799839 -0.411296 -0.008864

N 3.757943 1.620391 -0.041483

N 3.994554 0.553063 0.034832

N 1.441258 -2.678521 0.013878

N 1.067444 -1.609024 0.003547


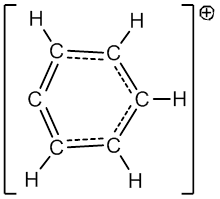


B3LYP/6-311++G**

Total electronic energy = -231.316276 Ha

Zero-point correction = 0.084508 Ha

Thermal correction to enthalpy = 0.090324 Ha

Thermal correction to free energy = 0.057546 Ha

C 0.000000 1.212372 0.613609

C 0.000000 1.268820 -0.819786

C 0.000000 0.000000 -1.189270

C 0.000000 -1.268820 -0.819786

C 0.000000 -1.212372 0.613609

C 0.000000 0.000000 1.296703

H 0.000000 2.172387 1.120996

H 0.000000 2.184090 -1.396083

H 0.000000 -2.184090 -1.396083

H 0.000000 -2.172387 1.120996

H 0.000000 0.000000 2.379695

RI-B2KPLYP/ma-def2-TZVP

Total electronic energy = -231.089556 Ha

Zero-point correction = 0.086165 Ha

Thermal correction to enthalpy = 0.091834 Ha

Thermal correction to free energy = 0.059682 Ha

C 0.000000 1.206963 0.610576

C -0.000001 1.266758 -0.816352

C -0.000000 -0.000000 -1.172715

C 0.000000 -1.266759 -0.816354

C -0.000001 -1.206961 0.610576

C -0.000000 0.000001 1.291246

H 0.000001 2.162177 1.117309

H -0.000001 2.176244 -1.393337

H 0.000001 -2.176245 -1.393336

H -0.000001 -2.162176 1.117308

H 0.000000 -0.000000 2.369682


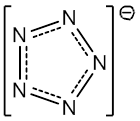


B3LYP/6-311++G**

Total electronic energy = -273.822812 Ha

Zero-point correction = 0.021452 Ha

Thermal correction to enthalpy = 0.025574 Ha

Thermal correction to free energy = -0.004430 Ha

N -0.707438 0.878379 0.000099

N 0.617063 0.943751 -0.000021

N 1.088874 -0.294868 -0.000065

N 0.055809 -1.126084 0.000126

N -1.054308 -0.401178 -0.000139

RI-B2KPLYP/ma-def2-TZVP

Total electronic energy = -273.616961 Ha

Zero-point correction = 0.021874 Ha

Thermal correction to enthalpy = 0.025968 Ha

Thermal correction to free energy = -0.002952 Ha

N -0.704254 0.874831 0.000006

N 0.614322 0.940043 -0.000003

N 1.084003 -0.293698 -0.000000

N 0.055502 -1.121569 0.000003

N -1.049572 -0.399607 -0.000006


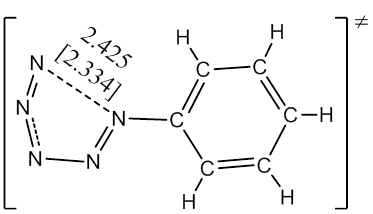


B3LYP/6-311++G**

Imaginary frequency = 239.3i cm^-1^

Total electronic energy = -505.398119 Ha

Zero-point correction = 0.110489 Ha

Thermal correction to enthalpy = 0.119767 Ha

Thermal correction to free energy = 0.075988 Ha

C 2.548939 1.289601 -0.000102

C 1.158441 1.229555 -0.000111

C 0.518339 -0.012242 0.000002

C 1.265842 -1.198149 0.000120

C 2.652587 -1.125498 0.000126

C 3.297710 0.114338 0.000016

H 3.045894 2.252615 -0.000189

H 0.554924 2.129019 -0.000203

H 0.756077 -2.152863 0.000205

H 3.235904 -2.039167 0.000217

H 4.380633 0.160585 0.000021

N -0.898433 0.021388 -0.000016

N -1.523647 -1.036895 0.000104

N -2.940722 -0.965828 0.000081

N -3.248846 0.294679 -0.000063

N -2.906147 1.381538 -0.000158

RI-B2KPLYP/ma-def2-TZVP

Imaginary frequency = 287.5 i cm^-1^

Total electronic energy = -504.966798 Ha

Zero-point correction = 0.112453 Ha

Thermal correction to enthalpy = 0.12146 Ha

Thermal correction to free energy = 0.079784 Ha

C 2.522625 1.301500 0.000620

C 1.138633 1.229086 -0.000896

C 0.516443 -0.011802 0.001517

C 1.265691 -1.186080 0.002875

C 2.646147 -1.101339 0.003256

C 3.277294 0.137866 0.002489

H 3.009400 2.264776 -0.000440

H 0.526257 2.117829 -0.003309

H 0.764893 -2.141055 0.002754

H 3.235786 -2.005553 0.003594

H 4.355319 0.192801 0.002740

N -0.896256 0.012053 -0.000824

N -1.501324 -1.052342 0.031904

N -2.915366 -0.994214 0.025687

N -3.195467 0.264719 -0.017995

N -2.817251 1.336436 -0.053824


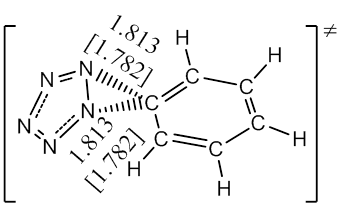


B3LYP/6-311++G**

Imaginary frequency = 595.3i cm^-1^

Total electronic energy = -505.345031 Ha

Zero-point correction = 0.110772 Ha

Thermal correction to enthalpy = 0.119356 Ha

Thermal correction to free energy = 0.077694 Ha

C 2.585270 -0.917877 -0.000001

C 1.245385 -1.295077 0.000003

C 0.310518 -0.276043 0.000004

C 0.607429 1.070642 0.000004

C 1.960226 1.417151 0.000000

C 2.944652 0.431878 -0.000003

H 3.348399 -1.687200 -0.000004

H 0.938540 -2.331520 0.000003

H -0.167633 1.826400 0.000010

H 2.232515 2.465843 0.000001

H 3.990771 0.712771 -0.000006

N -1.302134 -0.738576 -0.688479

N -1.302138 -0.738574 0.688481

N -2.193226 0.157440 1.082846

N -2.761206 0.652224 -0.000004

N -2.193220 0.157438 -1.082850

RI-B2KPLYP/ma-def2-TZVP

Imaginary frequency = 582.0i cm^-1^

Total electronic energy = -504.924683 Ha

Zero-point correction = 0.112970 Ha

Thermal correction to enthalpy = 0.121287 Ha

Thermal correction to free energy = 0.081370 Ha

C 2.567945 -0.906504 -0.000116

C 1.238051 -1.294994 -0.000314

C 0.296354 -0.289541 -0.000115

C 0.579517 1.054522 0.000241

C 1.922143 1.411960 0.000432

C 2.912148 0.440604 0.000261

H 3.336037 -1.664491 -0.000248

H 0.939834 -2.330098 -0.000601

H -0.202724 1.797985 0.000365

H 2.184351 2.458804 0.000715

H 3.951086 0.730974 0.000423

N -1.280723 -0.750152 -0.689922

N -1.280790 -0.750522 0.689272

N -2.140892 0.167502 1.076971

N -2.691264 0.680631 -0.000009

N -2.140787 0.168078 -1.077211


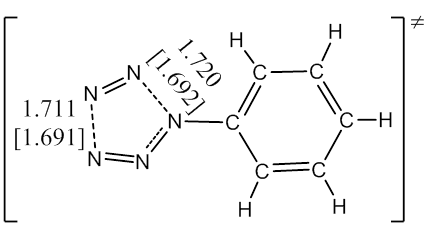


B3LYP/6-311++G**

Imaginary frequency = 559.2i cm^-1^

Total electronic energy = -505.416281 Ha

Total electronic energy solvent (THF) = -505.420955 Ha

Total electronic energy CCSD(T)/cc-pVTZ = -504.436407 Ha

Total electronic energy HF/cc-pVTZ = -502.362458 Ha

Total electronic energy CCSD(T)/cc-pVDZ = -503.966259 Ha

Total electronic energy HF/cc-pVDZ = -502.240137 Ha

Total electronic energy CCSD(T)/CBS = -504.688896 Ha

Zero-point correction = 0.110677 Ha

Thermal correction to enthalpy = 0.120071 Ha

Thermal correction to free energy = 0.075593 Ha

C -2.508355 -1.169436 0.227154

C -1.120784 -1.211887 0.140807

C -0.416641 -0.027735 -0.102426

C -1.090541 1.188257 -0.244502

C -2.476974 1.214553 -0.134825

C -3.191069 0.039511 0.095866

H -3.056311 -2.087205 0.406650

H -0.584001 -2.145520 0.251012

H -0.528288 2.093454 -0.434706

H -3.000394 2.157662 -0.239772

H -4.271604 0.064923 0.171576

N 0.990623 -0.010380 -0.192088

N 2.064856 1.217473 0.353313

N 3.116239 0.702925 0.329262

N 2.960948 -0.930574 -0.155586

N 1.762588 -1.019858 -0.341644

RI-B2KPLYP/ma-def2-TZVP

Imaginary frequency = 643.1i cm^-1^

Total electronic energy = -504.990635 Ha

Zero-point correction = 0.112246 Ha

Thermal correction to enthalpy = 0.121509 Ha

Thermal correction to free energy = 0.078652 Ha

C 2.509079 -1.167794 -0.200277

C 1.126725 -1.217830 -0.128746

C 0.420301 -0.038403 0.080834

C 1.077875 1.179108 0.205615

C 2.459441 1.211224 0.113625

C 3.179817 0.041700 -0.084355

H 3.062497 -2.081054 -0.357645

H 0.596841 -2.151869 -0.227624

H 0.508287 2.079758 0.371043

H 2.973882 2.155380 0.207304

H 4.256476 0.072097 -0.147599

N -0.980228 -0.030360 0.161624

N -2.035689 1.238710 -0.212420

N -3.093339 0.740082 -0.222497

N -2.953713 -0.916852 0.082692

N -1.753406 -1.036789 0.229155


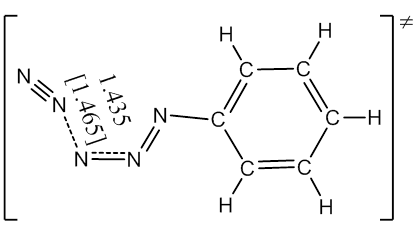


B3LYP/6-311++G**

Imaginary frequency = 684.1i cm^-1^

Total electronic energy = -505.399232 Ha

Zero-point correction = 0.109390 Ha

Thermal correction to enthalpy = 0.119303 Ha

Thermal correction to free energy = 0.073903 Ha

C -2.686319 1.326313 -0.000020

C -1.295258 1.279550 -0.000027

C -0.633937 0.046434 0.000004

C -1.377186 -1.143922 0.000043

C -2.764882 -1.085967 0.000050

C -3.425937 0.145348 0.000019

H -3.191660 2.285326 -0.000044

H -0.703653 2.187356 -0.000057

H -0.861790 -2.095803 0.000067

H -3.337457 -2.006796 0.000081

H -4.509259 0.180359 0.000025

N 0.778207 0.109252 -0.000007

N 1.396601 -0.983081 0.000024

N 2.683075 -1.003249 0.000015

N 3.252251 0.313952 -0.000035

N 4.133429 0.997842 -0.000068

RI-B2KPLYP/ma-def2-TZVP

Imaginary frequency = 842.0i cm^-1^

Total electronic energy = -504.965264 Ha

Zero-point correction = 0.110624 Ha

Thermal correction to enthalpy = 0.120456 Ha

Thermal correction to free energy = 0.076535 Ha

C 2.668670 1.322424 0.000026

C 1.283408 1.273543 0.000052

C 0.629360 0.046866 0.000015

C 1.367120 -1.136408 -0.000056

C 2.749359 -1.077296 -0.000082

C 3.406143 0.148053 -0.000042

H 3.170663 2.278247 0.000057

H 0.691755 2.176566 0.000105

H 0.854782 -2.085432 -0.000092

H 3.320635 -1.993653 -0.000140

H 4.484941 0.183775 -0.000065

N -0.778942 0.105191 0.000038

N -1.380666 -0.992922 0.000090

N -2.649667 -1.038013 0.000090

N -3.207182 0.316353 -0.000007

N -4.060428 1.028373 -0.000109


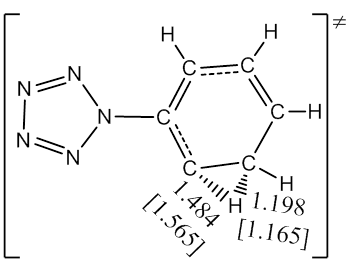


B3LYP/6-311++G**

Imaginary frequency = 729.1i cm^-1^

Total electronic energy = -505.300295 Ha

Zero-point correction = 0.108424 Ha

Thermal correction to enthalpy = 0.117350 Ha

Thermal correction to free energy = 0.074318 Ha

C 2.458306 -1.172306 -0.203453

C 1.016548 -1.293673 -0.309479

C 0.376601 -0.051938 -0.006394

C 1.009954 1.150541 0.203202

C 2.428991 1.217862 0.190528

C 3.159955 0.084481 -0.009138

H 2.911960 2.175172 0.345779

H 4.244007 0.110475 -0.027010

H 0.434332 2.052676 0.380108

N -1.062950 -0.038465 -0.013581

N -1.791882 1.004518 -0.409514

N -1.834283 -1.040734 0.395362

N -3.046817 -0.615055 0.250134

N -3.021190 0.642511 -0.246474

H 1.956068 -1.589845 0.800372

H 3.051345 -2.027702 -0.522325

RI-B2KPLYP/ma-def2-TZVP

Imaginary frequency = 451.7i cm^-1^

Total electronic energy = -504.875510 Ha

Zero-point correction = 0.111111 Ha

Thermal correction to enthalpy = 0.119801 Ha

Thermal correction to free energy = 0.078537 Ha

C 2.442214 -1.187082 -0.151887

C 0.998816 -1.311765 -0.256381

C 0.371123 -0.066007 0.001072

C 1.004353 1.135123 0.192854

C 2.414555 1.208780 0.155679

C 3.140052 0.075937 -0.028719

H 2.896074 2.166568 0.276326

H 4.218818 0.104977 -0.073854

H 0.426809 2.031362 0.368591

N -1.057557 -0.039075 -0.023617

N -1.758645 0.997481 -0.447685

N -1.836341 -1.015497 0.398888

N -3.041860 -0.576948 0.231612

N -2.994975 0.656256 -0.288745

H 2.040865 -1.504733 0.894858

H 3.028848 -2.042752 -0.463762


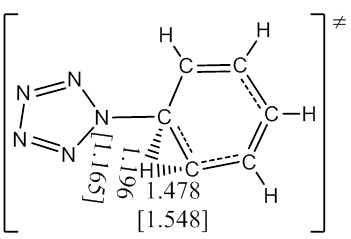


B3LYP/6-311++G**

Imaginary frequency = 687.9i cm^-1^

Total electronic energy = -505.290547 Ha

Zero-point correction = 0.108502 Ha

Thermal correction to enthalpy = 0.117445 Ha

Thermal correction to free energy = 0.074039 Ha

C 1.063477 -1.326400 0.316754

C 0.405143 -0.048717 0.167983

C 1.037522 1.198507 -0.226680

C 2.395064 1.202654 -0.328900

C 3.126785 0.001473 -0.100301

C 2.494467 -1.172426 0.234289

H 4.208194 0.028288 -0.198470

H 3.098637 -2.062468 0.384236

H 2.921116 2.111431 -0.596786

H 0.638974 -0.316991 1.309923

H 0.438759 2.084578 -0.402071

N -1.046954 -0.058172 0.072291

N -1.843447 0.698871 0.819755

N -3.039983 0.444437 0.401066

N -2.970787 -0.455290 -0.605667

N -1.733177 -0.769186 -0.812545

RI-B2KPLYP/ma-def2-TZVP

Imaginary frequency = 377.8i cm^-1^

Total electronic energy = -504.865842 Ha

Zero-point correction = 0.111198 Ha

Thermal correction to enthalpy = 0.119893 Ha

Thermal correction to free energy = 0.078352 Ha

C 1.056118 -1.328886 0.335941

C 0.406105 -0.045823 0.187515

C 1.033603 1.183038 -0.252173

C 2.386043 1.186456 -0.344984

C 3.107736 -0.006745 -0.089655

C 2.476912 -1.178803 0.239833

H 4.185696 0.016863 -0.175498

H 3.081075 -2.064755 0.377798

H 2.912517 2.083205 -0.632773

H 0.654706 -0.199801 1.315710

H 0.433736 2.056667 -0.458663

N -1.032804 -0.057583 0.087375

N -1.822796 0.686733 0.832722

N -3.019010 0.431422 0.408651

N -2.944518 -0.452063 -0.593510

N -1.704095 -0.760547 -0.798714


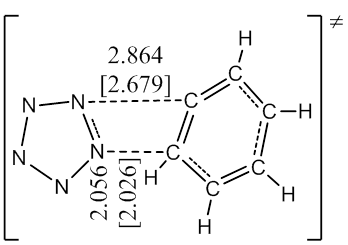


B3LYP/6-311++G**

Imaginary frequency = 192.7i cm^-1^

Total electronic energy = -505.261425 Ha

Zero-point correction = 0.107781 Ha

Thermal correction to enthalpy = 0.117432 Ha

Thermal correction to free energy = 0.071934 Ha

C -1.187706 0.859034 0.617435

C -0.690618 -0.368741 0.902111

C -1.535035 -1.354612 0.264548

C -2.596932 -0.948727 -0.526687

C -2.956089 0.395979 -0.652019

C -2.265486 1.417773 0.083666

H -3.636296 0.724967 -1.430806

H -2.458953 2.471317 -0.068021

H -3.194824 -1.697566 -1.033600

H -1.411462 -2.388897 0.565346

N 1.238389 -0.271242 0.198889

N 1.632054 0.968756 0.125751

N 2.883528 0.943821 -0.290267

N 3.233514 -0.329593 -0.460159

N 2.206726 -1.095749 -0.150336

H -0.266739 -0.626010 1.865609

RI-B2KPLYP/ma-def2-TZVP

Imaginary frequency = 343.9i cm^-1^

Total electronic energy = -504.832410 Ha

Zero-point correction = 0.110403 Ha

Thermal correction to enthalpy = 0.119788 Ha

Thermal correction to free energy = 0.076644 Ha

C -1.242709 -0.566813 -0.893328

C -0.635596 0.597192 -1.088498

C -1.728813 1.323936 -0.440073

C -2.633146 0.867687 0.522709

C -2.776842 -0.485210 0.763182

C -1.918705 -1.299150 -0.006918

H -3.375335 -0.898482 1.558475

H -1.675093 -2.325495 0.214533

H -3.247930 1.595934 1.028427

H -1.858513 2.313874 -0.856804

N 1.155194 0.332593 -0.178461

N 1.269787 -0.968814 -0.054660

N 2.457180 -1.201420 0.446965

N 3.063605 -0.037689 0.621671

N 2.255398 0.921088 0.227738

H -0.199500 1.028626 -1.968579


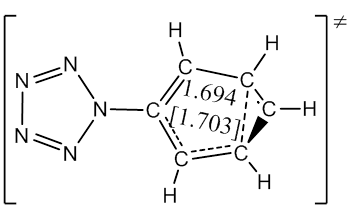


B3LYP/6-311++G**

Imaginary frequency = 377.4i cm^-1^

Total electronic energy = -505.285404 Ha

Zero-point correction = 0.109553 Ha

Thermal correction to enthalpy = 0.118373 Ha

Thermal correction to free energy = 0.075842 Ha

C -2.390355 -1.096335 -0.360614

C -1.106258 -0.885428 0.390951

C -0.303665 -0.045060 -0.365573

C -1.215931 0.880123 0.476013

C -2.580308 1.078329 0.315923

C -3.220809 -0.023803 -0.324371

H -0.636158 1.470978 1.175977

H -3.079757 1.967752 0.676716

H -4.189798 0.027734 -0.803149

N 1.084131 0.004391 -0.152868

N 1.819704 -1.012616 0.319478

N 3.042682 -0.605090 0.260241

N 3.067600 0.659078 -0.228913

N 1.863557 1.032730 -0.505299

H -2.516417 -1.990861 -0.957298

H -0.817623 -1.472017 1.265307

RI-B2KPLYP/ma-def2-TZVP

Imaginary frequency = 402.8i cm^-1^

Total electronic energy = -504.862807 Ha

Zero-point correction = 0.111853 Ha

Thermal correction to enthalpy = 0.120514 Ha

Thermal correction to free energy = 0.079322 Ha

C 2.386356 1.094132 -0.348835

C 1.100955 0.895472 0.383465

C 0.313972 0.060174 -0.376952

C 1.198918 -0.886165 0.438857

C 2.560677 -1.075259 0.315729

C 3.207248 0.022230 -0.313801

H 0.603735 -1.483314 1.114560

H 3.048991 -1.960257 0.689321

H 4.175470 -0.028017 -0.784089

N -1.069931 0.002897 -0.163822

N -1.794099 1.000008 0.328500

N -3.014602 0.579503 0.293552

N -3.035864 -0.666842 -0.204211

N -1.832054 -1.024383 -0.505616

H 2.528813 1.995434 -0.924131

H 0.799350 1.480766 1.249019


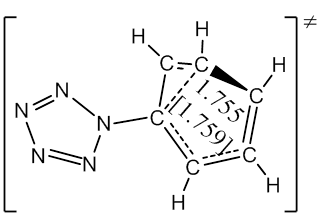


B3LYP/6-311++G**

Imaginary frequency = 455.3i cm^-1^

Total electronic energy = -505.267856 Ha

Zero-point correction = 0.108691 Ha

Thermal correction to enthalpy = 0.117619 Ha

Thermal correction to free energy = 0.074791 Ha

C -2.829429 -0.232443 0.301276

C -1.602120 -0.986786 0.730304

C -1.106794 -1.387647 -0.503631

C -0.534996 0.002013 -0.251368

C -1.219380 1.237542 -0.347196

C -2.590416 1.038242 -0.176301

H -0.710054 2.175286 -0.512519

H -3.356386 1.732651 -0.493527

H -3.773897 -0.761225 0.266096

H -1.401275 -1.344698 1.742345

N 0.885773 0.029668 -0.069760

H -0.470331 -2.243323 -0.698804

N 1.566126 1.096681 0.355042

N 2.807506 0.739102 0.328974

N 2.894282 -0.539132 -0.096378

N 1.704991 -0.981205 -0.349605

RI-B2KPLYP/ma-def2-TZVP

Imaginary frequency = 432.9i cm^-1^

Total electronic energy = -504.847740 Ha

Zero-point correction = 0.111334 Ha

Thermal correction to enthalpy = 0.119969 Ha

Thermal correction to free energy = 0.078971 Ha

C 2.825367 -0.227530 -0.296995

C 1.610871 -0.990603 -0.719742

C 1.088801 -1.384301 0.496266

C 0.536787 0.002864 0.257491

C 1.210342 1.232616 0.326400

C 2.582248 1.033479 0.172995

H 0.694999 2.166654 0.467130

H 3.341524 1.725597 0.496774

H 3.769264 -0.749964 -0.264150

H 1.424582 -1.358210 -1.725927

N -0.876245 0.028196 0.082439

H 0.434352 -2.224679 0.673501

N -1.543032 1.076712 -0.372556

N -2.786274 0.724905 -0.336162

N -2.876435 -0.527112 0.125834

N -1.688303 -0.965255 0.391410


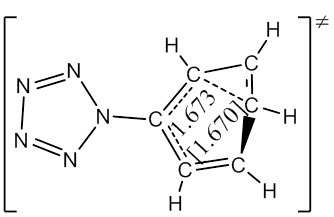


B3LYP/6-311++G**

Imaginary frequency = 273.0i cm^-1^

Total electronic energy = -505.277712 Ha

Zero-point correction = 0.109239 Ha

Thermal correction to enthalpy = 0.118020 Ha

Thermal correction to free energy = 0.075663 Ha

C -2.401067 1.133506 0.012455

C -2.672458 -0.234395 0.576037

C -2.639817 -1.062422 -0.550854

C -1.252514 -0.955009 0.063361

C -0.380908 0.155078 -0.047209

C -1.072950 1.367426 -0.224479

H -0.881580 -1.895452 0.455083

H -0.637779 2.272871 -0.621994

H -3.222343 1.779226 -0.268082

H -3.104857 -0.402469 1.563879

H -3.150113 -2.017715 -0.639699

N 1.011394 0.030491 -0.002936

N 1.855584 1.062509 0.089493

N 3.037275 0.534228 0.072111

N 2.925245 -0.807091 -0.013279

N 1.672638 -1.128932 -0.068967

RI-B2KPLYP/ma-def2-TZVP

Imaginary frequency = 261.3i cm^-1^

Total electronic energy = -504.856737 Ha

Zero-point correction = 0.111691 Ha

Thermal correction to enthalpy = 0.120300 Ha

Thermal correction to free energy = 0.079409 Ha

C -2.386586 1.143059 0.025490

C -2.664397 -0.220676 0.572551

C -2.643389 -1.069788 -0.525542

C -1.241140 -0.958187 0.006995

C -0.388140 0.151465 -0.071446

C -1.072525 1.368328 -0.233459

H -0.858397 -1.899247 0.375659

H -0.638948 2.267728 -0.635967

H -3.208074 1.793557 -0.228048

H -3.069722 -0.387755 1.567137

H -3.130393 -2.035119 -0.565157

N 0.999205 0.026791 -0.010468

N 1.826693 1.052173 0.104315

N 3.011272 0.526157 0.104238

N 2.903607 -0.800390 0.005249

N 1.650583 -1.121983 -0.074594


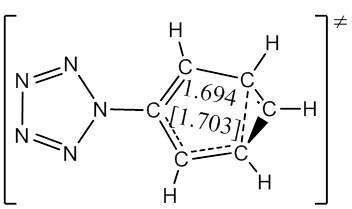


B3LYP/6-311++G**

Imaginary frequency = 351.6i cm^-1^

Total electronic energy = -505.274178 Ha

Zero-point correction = 0.109066 Ha

Thermal correction to enthalpy = 0.117924 Ha

Thermal correction to free energy = 0.075316 Ha

C -1.149717 -1.095688 -0.054788

C -2.577927 -0.763783 -0.350087

C -2.972662 -0.192081 0.858485

C -2.442122 0.901024 -0.066382

C -1.090205 1.177021 -0.361605

C -0.335959 0.006133 -0.211730

H -0.857095 -2.065711 0.319171

H -3.153239 -1.186688 -1.175297

H -3.987505 -0.173619 1.248383

H -3.216841 1.599665 -0.367229

H -0.709796 2.147504 -0.644175

N 1.068008 -0.006632 -0.052194

N 1.807683 1.074829 0.183353

N 3.028110 0.646096 0.230332

N 3.036101 -0.689057 0.034964

N 1.822390 -1.099079 -0.148485

RI-B2KPLYP/ma-def2-TZVP

Imaginary frequency = 290.1i cm^-1^

Total electronic energy = -504.853813 Ha

Zero-point correction = 0.111521 Ha

Thermal correction to enthalpy = 0.120169 Ha

Thermal correction to free energy = 0.079147 Ha

C -1.136336 -1.098578 -0.077101

C -2.560948 -0.764239 -0.346757

C -2.981019 -0.198949 0.847135

C -2.425828 0.902976 -0.028709

C -1.088331 1.170758 -0.349214

C -0.336116 -0.001051 -0.215643

H -0.841359 -2.072293 0.274829

H -3.129172 -1.164694 -1.182110

H -4.007190 -0.161753 1.189755

H -3.196015 1.607766 -0.312203

H -0.714224 2.138230 -0.635607

N 1.059683 -0.009110 -0.056195

N 1.787840 1.074129 0.136940

N 3.008354 0.647637 0.207859

N 3.016455 -0.681708 0.066183

N 1.802682 -1.097835 -0.105355


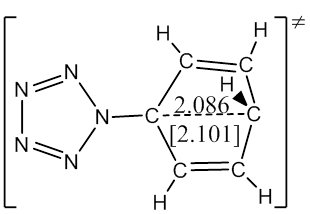


B3LYP/6-311++G**

Imaginary frequency = 250.6i cm^-1^

Total electronic energy = -505.277828 Ha

Zero-point correction = 0.109482 Ha

Thermal correction to enthalpy = 0.118081 Ha

Thermal correction to free energy = 0.076354 Ha

C 1.154463 1.300721 -0.431125

C 2.426586 1.246269 0.034572

C 2.348655 0.000000 0.793792

C 2.426587 -1.246268 0.034571

C 1.154463 -1.300720 -0.431126

C 0.505942 0.000000 -0.184790

H 0.661731 2.100188 -0.975560

H 3.297385 1.805920 -0.279494

H 3.297385 -1.805919 -0.279495

H 0.661731 -2.100188 -0.975561

N -0.902787 0.000000 -0.034662

N -1.657217 1.101287 0.031025

N -2.874121 0.676009 0.123531

N -2.874121 -0.676009 0.123532

N -1.657217 -1.101287 0.031026

H 1.739838 -0.000001 1.693578

RI-B2KPLYP/ma-def2-TZVP

Imaginary frequency = 183.3i cm^-1^

Total electronic energy = -504.857338 Ha

Zero-point correction = 0.111924 Ha

Thermal correction to enthalpy = 0.120297 Ha

Thermal correction to free energy = 0.080081 Ha

C -1.145878 -1.292337 -0.398574

C -2.430589 -1.234241 0.030100

C -2.376334 0.000025 0.786668

C -2.430580 1.234264 0.030051

C -1.145870 1.292328 -0.398629

C -0.503813 -0.000002 -0.165125

H -0.644943 -2.091337 -0.927217

H -3.286978 -1.789862 -0.314483

H -3.286964 1.789881 -0.314547

H -0.644928 2.091301 -0.927307

N 0.899951 -0.000000 -0.036549

N 1.643853 -1.094269 0.022082

N 2.861605 -0.670136 0.103371

N 2.861606 0.670140 0.103348

N 1.643853 1.094270 0.022044

H -1.722940 0.000040 1.651831


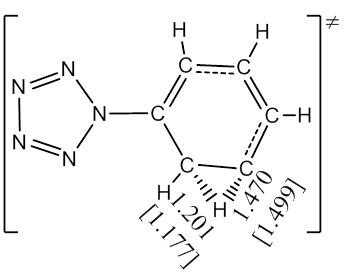


B3LYP/6-311++G**

Imaginary frequency = 819.4i cm^-1^

Total electronic energy = -505.295694 Ha

Zero-point correction = 0.108236 Ha

Thermal correction to enthalpy = 0.117275 Ha

Thermal correction to free energy = 0.073536 Ha

C 2.487617 1.168895 -0.002329

C 1.065946 1.208652 0.008382

C 0.381769 0.029938 -0.002861

C 1.103267 -1.234476 -0.013380

C 2.548700 -1.315294 -0.100536

C 3.178021 -0.017912 -0.010050

H 3.020041 2.115605 0.003892

H 0.539411 2.154030 0.022318

H 0.520042 -2.132064 -0.201131

H 4.263737 0.016807 -0.017350

N -1.038664 0.020659 -0.001557

N -1.781182 -1.087320 -0.008914

N -3.005620 -0.678086 -0.008072

N -3.020344 0.675644 -0.001327

N -1.806447 1.113003 0.003416

H 1.630639 -1.500504 1.032085

RI-B2KPLYP/ma-def2-TZVP

Imaginary frequency = 540.4i cm^-1^

Total electronic energy = -504.870890 Ha

Zero-point correction = 0.110987 Ha

Thermal correction to enthalpy = 0.119686 Ha

Thermal correction to free energy = 0.078468 Ha

C 2.475549 1.156813 0.016869

C 1.062413 1.199735 0.039495

C 0.384984 0.024614 0.010938

C 1.098007 -1.233358 0.010128

C 2.542175 -1.317735 -0.113656

C 3.163705 -0.025191 -0.050643

H 3.010110 2.096632 0.043062

H 0.534752 2.139569 0.064056

H 0.513577 -2.129547 -0.148784

H 4.242566 0.015845 -0.112429

N -1.026863 0.016397 -0.009462

N -1.759372 -1.083681 -0.026679

N -2.984008 -0.672765 -0.037544

N -2.996735 0.667112 -0.027265

N -1.781456 1.102724 -0.009833

H 1.607531 -1.419588 1.054332


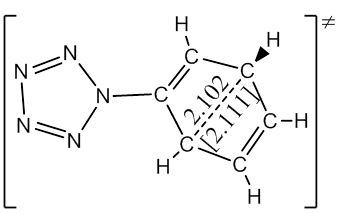


B3LYP/6-311++G**

Imaginary frequency = 422.2i cm^-1^

Total electronic energy = -505.274810 Ha

Zero-point correction = 0.109556 Ha

Thermal correction to enthalpy = 0.118379 Ha

Thermal correction to free energy = 0.075842 Ha

C -0.360731 -0.226613 0.148861

C -1.069604 -1.357211 -0.125747

C -2.333008 -0.889686 0.414749

C -3.145406 -0.020248 -0.445133

C -2.487380 1.142616 -0.223450

C -1.220245 0.890899 0.495898

H -0.866576 -2.151910 -0.830434

H -3.893898 -0.279102 -1.180953

H -2.774914 2.142519 -0.536655

H -2.401888 -0.737780 1.488971

N 1.049124 -0.065185 0.037625

H -0.824593 1.664829 1.150452

N 1.923855 -1.068607 0.041996

N 3.082922 -0.505821 -0.084596

N 2.922160 0.831852 -0.152580

N 1.659097 1.111032 -0.082794

RI-B2KPLYP/ma-def2-TZVP

Imaginary frequency = 360.12i cm^-1^

Total electronic energy = -504.853966 Ha

Zero-point correction = 0.111814 Ha

Thermal correction to enthalpy = 0.120469 Ha

Thermal correction to free energy = 0.079295 Ha

C -0.368857 -0.225263 0.157764

C -1.070442 -1.356340 -0.113377

C -2.327618 -0.893420 0.418849

C -3.130686 -0.036619 -0.440963

C -2.481421 1.129981 -0.220487

C -1.213738 0.899220 0.482530

H -0.857713 -2.153083 -0.806406

H -3.867697 -0.298012 -1.181253

H -2.774164 2.121210 -0.539622

H -2.365948 -0.686367 1.482560

N 1.033090 -0.065195 0.044215

H -0.804269 1.687772 1.103728

N 1.895269 -1.063690 0.037895

N 3.054963 -0.501351 -0.092426

N 2.896064 0.823866 -0.152920

N 1.632824 1.102913 -0.073459


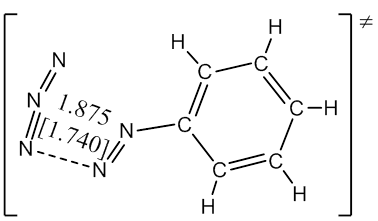


B3LYP/6-311++G**

Imaginary frequency = 126.4i cm^-1^

Total electronic energy = -505.392298 Ha

Zero-point correction = 0.110519 Ha

Thermal correction to enthalpy = 0.120174 Ha

Thermal correction to free energy = 0.075738 Ha

C 2.407195 1.356080 0.000001

C 1.029838 1.156267 -0.000006

C 0.539563 -0.147676 0.000005

C 1.391045 -1.257501 0.000024

C 2.762760 -1.040913 0.000031

C 3.270406 0.261308 0.000019

H 2.804405 2.363896 -0.000009

H 0.328143 1.981041 -0.000020

H 0.976918 -2.258007 0.000032

H 3.439123 -1.887513 0.000046

H 4.342647 0.419600 0.000025

N -0.874520 -0.297450 -0.000003

N -1.536062 -1.261845 0.000002

N -3.335715 -0.735531 -0.000016

N -3.140757 0.453866 -0.000026

N -2.583814 1.471760 -0.000033

RI-B2KPLYP/ma-def2-TZVP

Imaginary frequency = 186.9i cm^-1^

Total electronic energy = -504.963406 Ha

Zero-point correction = 0.112410 Ha

Thermal correction to enthalpy = 0.121750 Ha

Thermal correction to free energy = 0.079494 Ha

C 2.424762 1.343814 0.000005

C 1.048679 1.179832 -0.000013

C 0.527692 -0.103907 -0.000007

C 1.344657 -1.229735 0.000007

C 2.715604 -1.050039 0.000021

C 3.255191 0.232180 0.000024

H 2.846089 2.337124 0.000007

H 0.369946 2.018740 -0.000027

H 0.905334 -2.215065 0.000008

H 3.367584 -1.909895 0.000034

H 4.326766 0.361736 0.000041

N -0.884836 -0.202413 -0.000001

N -1.512067 -1.197755 0.000089

N -3.211823 -0.824980 0.000032

N -3.133401 0.386457 -0.000050

N -2.645309 1.431105 -0.000108

The xyz coordinates of geometries in Fig. 3:

­­­___________________________________________________________


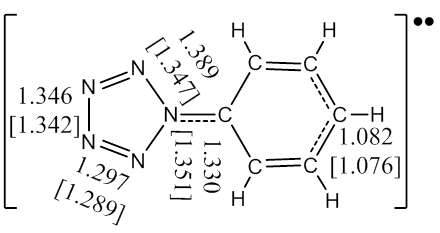


B3LYP/6-311++G**

Total electronic energy = - 505.330909 Ha

Zero-point correction = 0.109480 Ha

Thermal correction to enthalpy = 0.118875 Ha

Thermal correction to free energy = 0.074583 Ha

C 2.426594 1.242491 0.000009

C 1.070630 1.271934 0.000009

C 0.337284 -0.000003 0.000000

C 1.070640 -1.271934 -0.000009

C 2.426604 -1.242480 -0.000009

C 3.142076 0.000008 0.000000

H 2.983943 2.171727 0.000016

H 0.504782 2.192386 0.000016

H 0.504799 -2.192390 -0.000017

H 2.983961 -2.171711 -0.000017

H 4.224276 0.000013 0.000000

N -0.992656 -0.000007 0.000001

N -1.790712 -1.137104 -0.000006

N -3.001846 -0.673175 0.000010

N -3.001874 0.673184 -0.000014

N -1.790731 1.137085 0.000011

RI-B2KPLYP/ma-def2-TZVP

Total electronic energy = -504.892416 Ha

Zero-point correction = 0.112182 Ha

Thermal correction to enthalpy = 0.12159 Ha

Thermal correction to free energy = 0.078302 Ha

C -2.406841 1.251222 -0.000018

C -1.068831 1.267532 -0.000022

C -0.353699 -0.000000 -0.000007

C -1.068825 -1.267536 0.000015

C -2.406835 -1.251232 0.000022

C -3.138272 -0.000007 0.000004

H -2.958135 2.178668 -0.000031

H -0.500984 2.182419 -0.000039

H -0.500973 -2.182419 0.000028

H -2.958123 -2.178681 0.000040

H -4.214561 -0.000009 0.000008

N 0.996796 0.000002 -0.000009

N 1.762628 -1.107625 -0.000012

N 2.975867 -0.670926 -0.000143

N 2.975864 0.670939 0.000191

N 1.762623 1.107632 -0.000027


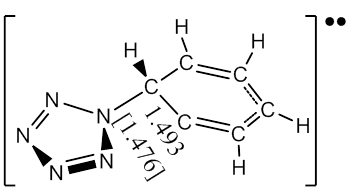


B3LYP/6-311++G**

Total electronic energy = -505.303901 Ha

Zero-point correction = 0.110849 Ha

Thermal correction to enthalpy = 0.120112 Ha

Thermal correction to free energy = 0.074743 Ha

C -1.120365 -1.107747 -0.700716

C -0.344638 0.145120 -0.827417

C -1.088857 1.296606 -0.179170

C -2.297918 1.121043 0.419785

C -2.941019 -0.144002 0.482672

C -2.314333 -1.278073 -0.100389

H -3.902757 -0.246365 0.969636

H -2.795882 -2.249815 -0.063870

H -2.788167 1.978428 0.867802

H -0.605855 2.266757 -0.215766

N 1.024398 0.011677 -0.247318

N 2.133602 0.235537 -0.938448

N 3.100351 0.040123 -0.098354

N 2.578982 -0.301089 1.098577

N 1.285822 -0.317664 1.007382

H -0.126644 0.373227 -1.879270

RI-B2KPLYP/ma-def2-TZVP

Total electronic energy = -504.871717 Ha

Zero-point correction = 0.113627 Ha

Thermal correction to enthalpy = 0.121797 Ha

Thermal correction to free energy = 0.080713 Ha

C 1.120398 -1.073203 0.753545

C 0.347790 0.176120 0.844732

C 1.081506 1.297702 0.160185

C 2.261040 1.105544 -0.453129

C 2.900825 -0.160548 -0.492266

C 2.292412 -1.264111 0.137267

H 3.845941 -0.276974 -0.996570

H 2.768854 -2.233143 0.123216

H 2.741468 1.943789 -0.934950

H 0.603909 2.266367 0.176744

N -0.996471 0.018978 0.255970

N -2.108270 0.283282 0.907314

N -3.057269 0.049615 0.056090

N -2.516510 -0.351306 -1.098282

N -1.225266 -0.369051 -0.977057

H 0.125171 0.435586 1.882574


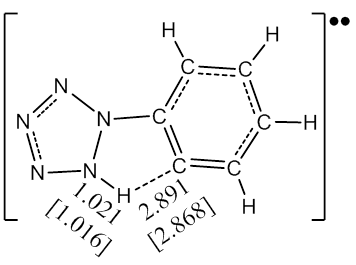


B3LYP/6-311++G**

Total electronic energy = -505.304949 Ha

Zero-point correction = 0.111431 Ha

Thermal correction to enthalpy = 0.120677 Ha

Thermal correction to free energy = 0.076374 Ha

C -2.532123 1.148898 -0.004327

C -1.145153 1.214835 0.037351

C -0.399631 0.017574 0.037847

C -1.111633 -1.169766 0.005738

C -2.475949 -1.273498 -0.040461

C -3.207087 -0.075312 -0.042097

H -3.099035 2.072457 -0.009928

H -2.976283 -2.234831 -0.074505

H 1.628323 -1.840181 0.637775

N 0.986861 0.027498 0.107414

N 1.786243 -1.104598 -0.051793

N 1.780870 1.127725 0.003466

N 3.010085 0.675061 -0.073698

N 3.091497 -0.615801 -0.057683

H -0.622455 2.162652 0.063152

H -4.289986 -0.105676 -0.074733

RI-B2KPLYP/ma-def2-TZVP

Total electronic energy = -504.860916 Ha

Zero-point correction = 0.114735 Ha

Thermal correction to enthalpy = 0.123771 Ha

Thermal correction to free energy = 0.080776 Ha

C -2.513481 1.137196 -0.006296

C -1.138629 1.201552 0.051278

C -0.405299 0.016376 0.062761

C -1.106553 -1.159893 0.022461

C -2.461736 -1.262594 -0.039538

C -3.182322 -0.076283 -0.049663

H -3.078226 2.056631 -0.019342

H -2.958523 -2.220024 -0.079170

H 1.610891 -1.839381 0.637593

N 0.976716 0.027591 0.151468

N 1.761774 -1.078564 -0.017944

N 1.761886 1.130218 -0.013822

N 2.970597 0.673342 -0.113237

N 3.056129 -0.600501 -0.070198

H -0.615910 2.144578 0.082184

H -4.260290 -0.103686 -0.095113


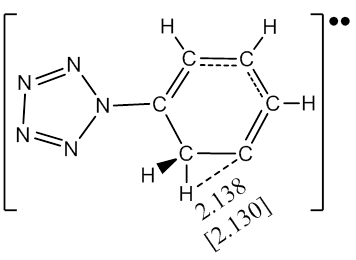


B3LYP/6-311++G**

Total electronic energy = -505.315335 Ha

Zero-point correction = 0.110680 Ha

Thermal correction to enthalpy = 0.119854 Ha

Thermal correction to free energy = 0.075662 Ha

C 2.514371 1.172845 -0.000191

C 1.109034 1.186170 -0.000186

C 0.389604 0.015477 -0.000016

C 1.030427 -1.356670 0.000176

C 2.496154 -1.198852 0.000130

C 3.222342 -0.070124 -0.000025

H 3.060905 2.107553 -0.000326

H 0.577170 2.129142 -0.000320

H 0.688359 -1.937885 0.870128

H 4.307169 -0.080580 -0.000037

N -1.016450 0.037704 0.000007

N -1.769623 -1.065406 -0.000024

N -2.995895 -0.640123 0.000053

N -2.997507 0.704449 -0.000002

N -1.776744 1.138642 0.000080

H 0.688330 -1.938161 -0.869577

RI-B2KPLYP/ma-def2-TZVP

Total electronic energy = -504.881141 Ha

Zero-point correction = 0.113339 Ha

Thermal correction to enthalpy = 0.122358 Ha

Thermal correction to free energy = 0.079425 Ha

C 2.507131 1.165203 -0.000187

C 1.101446 1.173268 -0.000188

C 0.399523 0.017824 -0.000004

C 1.022931 -1.347523 0.000214

C 2.483445 -1.190277 0.000155

C 3.204819 -0.069233 -0.000017

H 3.048492 2.096872 -0.000331

H 0.567885 2.110649 -0.000338

H 0.681461 -1.921851 0.868043

H 4.284543 -0.083939 -0.000032

N -1.007653 0.040743 0.000020

N -1.745857 -1.055148 0.000021

N -2.971789 -0.637254 -0.000309

N -2.975915 0.698532 0.000395

N -1.756295 1.130098 0.000010

H 0.681418 -1.922161 -0.867390


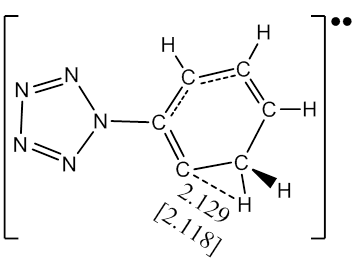


B3LYP/6-311++G**

Total electronic energy = -505.306908 Ha

Zero-point correction = 0.110395 Ha

Thermal correction to enthalpy = 0.119718 Ha

Thermal correction to free energy = 0.074634 Ha

C 2.438887 1.225422 -0.000201

C 1.020859 1.246706 -0.000203

C 0.335902 0.000462 0.000000

C 1.038131 -1.151361 0.000174

C 2.501183 -1.298134 0.000162

C 3.160142 0.068410 -0.000035

H 2.962908 2.174790 -0.000346

H 0.463981 2.173044 -0.000348

H 4.244439 0.091781 -0.000046

N -1.092272 -0.018468 0.000012

N -1.825350 -1.128631 -0.000079

N -3.053559 -0.722637 -0.000682

N -3.074115 0.626958 0.000709

N -1.856940 1.068254 0.000166

H 2.836829 -1.883611 -0.871555

H 2.836861 -1.883374 0.872026

RI-B2KPLYP/ma-def2-TZVP

Total electronic energy = -504.873333 Ha

Zero-point correction = 0.113106 Ha

Thermal correction to enthalpy = 0.122246 Ha

Thermal correction to free energy = 0.078692 Ha

C 2.431799 1.213997 -0.000128

C 1.015200 1.236737 -0.000123

C 0.340517 -0.000086 -0.000010

C 1.034682 -1.143687 0.000092

C 2.492092 -1.288464 0.000088

C 3.141573 0.069515 -0.000032

H 2.954702 2.158452 -0.000213

H 0.459848 2.158520 -0.000205

H 4.221096 0.096808 -0.000037

N -1.079621 -0.018782 0.000000

N -1.801633 -1.121591 -0.000089

N -3.029799 -0.716816 0.000362

N -3.050100 0.621374 -0.000322

N -1.833327 1.061419 0.000122

H 2.824697 -1.867656 -0.869122

H 2.824718 -1.867512 0.869386


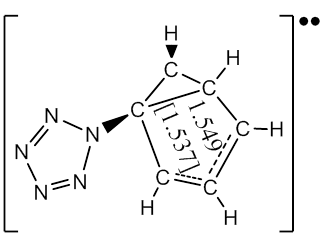


B3LYP/6-311++G**

Total electronic energy = -505.271311 Ha

Zero-point correction = 0.108709 Ha

Thermal correction to enthalpy= 0.117937 Ha

Thermal correction to free energy= 0.073210 Ha

C 1.072511 1.578474 -0.316120

C 1.459205 0.751755 0.852032

C 2.705397 -0.050390 0.709271

C 2.567554 -0.955162 -0.343845

C 1.310687 -0.875248 -0.930632

C 0.532391 0.201381 -0.260953

H 1.624088 2.097966 -1.087442

H 1.065686 1.013099 1.830837

N -0.885496 0.030226 -0.071557

N -1.816622 0.857941 -0.538324

N -2.943328 0.340167 -0.171708

N -2.704695 -0.803500 0.508362

N -1.427523 -0.997662 0.573739

H 3.583225 0.085015 1.325068

H 0.938931 -1.456969 -1.761831

H 3.345240 -1.634169 -0.668737

RI-B2KPLYP/ma-def2-TZVP

Total electronic energy = -504.845639 Ha

Zero-point correction = 0.111614 Ha

Thermal correction to enthalpy= 0.120558 Ha

Thermal correction to free energy= 0.077675 Ha

C 1.072999 1.529549 -0.474132

C 1.441628 0.829812 0.772557

C 2.680102 0.024464 0.721247

C 2.553115 -0.974133 -0.231403

C 1.310054 -0.956242 -0.826193

C 0.534222 0.174258 -0.280592

H 1.650695 1.942150 -1.283449

H 1.035325 1.192343 1.707196

N -0.872826 0.017986 -0.090650

N -1.780880 0.910750 -0.431741

N -2.911609 0.388095 -0.077931

N -2.688153 -0.813448 0.464619

N -1.414414 -1.048254 0.462411

H 3.551405 0.220975 1.321564

H 0.942701 -1.620422 -1.589047

H 3.332674 -1.674287 -0.483434


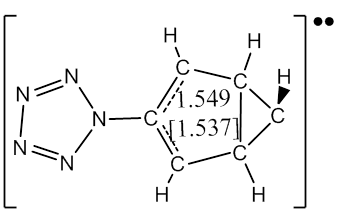


B3LYP/6-311++G**

Total electronic energy = -505.274790 Ha

Zero-point correction = 0.108758 Ha

Thermal correction to enthalpy = 0.117920 Ha

Thermal correction to free energy = 0.073567 Ha

C -0.267095 -2.545854 0.774609

C -0.212462 -1.117019 1.163899

C -0.156467 -0.353784 0.000000

C -0.212462 -1.117019 -1.163899

C -0.267095 -2.545854 -0.774609

C 0.900518 -3.048213 0.000000

H -0.862923 -3.251570 1.345512

H -0.190621 -0.734981 2.173102

H -0.190621 -0.734981 -2.173102

H -0.862923 -3.251570 -1.345512

H 1.942433 -2.754705 0.000000

N -0.035619 1.059227 0.000000

N 0.017926 1.807292 -1.098968

N 0.103815 3.026970 -0.674927

N 0.103815 3.026970 0.674927

N 0.017926 1.807292 1.098968

RI-B2KPLYP/ma-def2-TZVP

Total electronic energy = -504.848545 Ha

Zero-point correction = 0.111632 Ha

Thermal correction to enthalpy = 0.120532 Ha

Thermal correction to free energy = 0.077892 Ha

C -0.270887 -2.534323 0.768413

C -0.213193 -1.114857 1.154774

C -0.154155 -0.360581 -0.000005

C -0.213189 -1.114863 -1.154783

C -0.270880 -2.534330 -0.768416

C 0.896023 -3.025858 0.000006

H -0.858310 -3.241189 1.335903

H -0.187655 -0.731209 2.158812

H -0.187650 -0.731219 -2.158822

H -0.858295 -3.241203 -1.335905

H 1.921979 -2.696615 0.000009

N -0.032748 1.045100 -0.000002

N 0.020118 1.781875 -1.091809

N 0.106266 3.001567 -0.668988

N 0.106294 3.001560 0.669002

N 0.020169 1.781863 1.091811


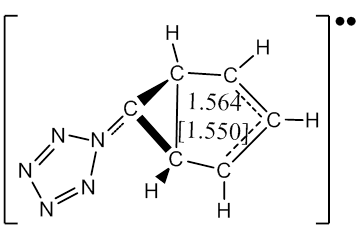


B3LYP/6-311++G**

Total electronic energy = -505.281197 Ha

Zero-point correction = 0.109733 Ha

Thermal correction to enthalpy = 0.118759 Ha

Thermal correction to free energy = 0.074772 Ha

C 1.869363 -1.153166 0.540287

C 2.024103 0.000288 1.304853

C 1.869277 1.153406 0.539797

C 1.562506 0.781582 -0.868059

C 0.326396 -0.000277 -1.086676

C 1.562558 -0.781947 -0.867725

H 1.925443 -2.171217 0.897465

H 2.225476 0.000512 2.368711

H 1.960535 1.350840 -1.702197

H 1.960637 -1.351519 -1.701628

N -0.875559 -0.000117 -0.420106

N -1.565609 -1.107517 -0.091323

N -2.659782 -0.679475 0.434879

N -2.659656 0.679711 0.434793

N -1.565480 1.107463 -0.091636

H 1.925292 2.171615 0.896539

RI-B2KPLYP/ma-def2-TZVP

Total electronic energy = -504.853668 Ha

Zero-point correction = 0.112793 Ha

Thermal correction to enthalpy = 0.121563 Ha

Thermal correction to free energy = 0.079141 Ha

C 1.809298 -1.143310 0.527832

C 1.917680 0.001519 1.294861

C 1.809173 1.144577 0.525197

C 1.574440 0.774228 -0.885179

C 0.349359 -0.001358 -1.143478

C 1.574528 -0.776228 -0.883387

H 1.847574 -2.156570 0.887322

H 2.055186 0.002757 2.364063

H 1.998867 1.339886 -1.701562

H 1.999019 -1.343731 -1.698454

N -0.830655 -0.000538 -0.433308

N -1.488457 -1.097698 -0.072235

N -2.553562 -0.673568 0.512036

N -2.553141 0.674630 0.511290

N -1.488310 1.097442 -0.074436

H 1.847306 2.158660 0.882369


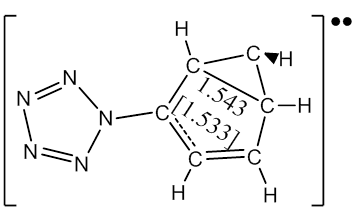


B3LYP/6-311++G**

Total electronic energy = -505.281673 Ha

Zero-point correction = 0.109275 Ha

Thermal correction to enthalpy = 0.118339 Ha

Thermal correction to free energy = 0.074544 Ha

C -2.708857 -0.346328 -0.311874

C -2.476243 1.110968 -0.112720

C -1.127406 1.371851 0.015330

C -0.400142 0.176632 -0.117155

C -1.303891 -0.983349 -0.304389

C -2.265565 -1.219358 0.805486

H -3.484343 -0.698245 -0.985639

H -3.265161 1.847215 -0.044952

H -2.192416 -1.052015 1.871968

N 0.976022 0.061073 -0.046015

N 1.640388 -1.096730 -0.176647

N 2.892245 -0.779925 -0.059548

N 3.002505 0.546753 0.140340

N 1.823266 1.083542 0.150883

H -0.681741 2.337320 0.209106

H -1.024684 -1.799761 -0.961640

RI-B2KPLYP/ma-def2-TZVP

Total electronic energy = -504.853645 Ha

Zero-point correction = 0.112153 Ha

Thermal correction to enthalpy= 0.121023 Ha

Thermal correction to free energy= 0.078563 Ha

C -2.695054 -0.344193 -0.317280

C -2.466737 1.102677 -0.116707

C -1.126638 1.363285 0.017287

C -0.409052 0.180045 -0.120692

C -1.297903 -0.974699 -0.311696

C -2.249211 -1.205855 0.798686

H -3.469070 -0.701785 -0.980785

H -3.253013 1.834868 -0.049386

H -2.159780 -1.005104 1.853181

N 0.967429 0.062287 -0.046480

N 1.614040 -1.086931 -0.177250

N 2.865263 -0.783127 -0.052967

N 2.980153 0.533985 0.151438

N 1.803686 1.070273 0.156728

H -0.684077 2.323732 0.219782

H -1.019897 -1.790183 -0.962089


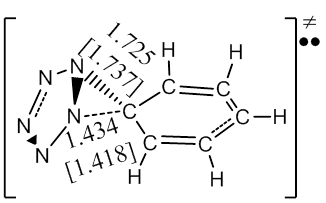


B3LYP/6-311++G**

Imaginary frequency = 334.3i cm^-1^

Total electronic energy = -505.270580 Ha

Zero-point correction = 0.107829 Ha

Thermal correction to enthalpy = 0.116570 Ha

Thermal correction to free energy = 0.073936 Ha

C -2.501434 -0.889193 0.056737

C -1.220295 -1.307571 -0.145091

C -0.138301 -0.328579 -0.209091

C -0.480048 1.091422 -0.186771

C -1.781142 1.460059 0.015618

C -2.804138 0.493452 0.157704

H -3.301609 -1.616713 0.125462

H -0.955880 -2.351781 -0.244939

H 0.303215 1.825274 -0.311156

H -2.035133 2.512794 0.056087

H -3.825452 0.810911 0.327516

N 1.168417 -0.823201 0.801887

N 1.141347 -0.798303 -0.654001

N 2.055456 0.188730 -1.054157

N 2.604460 0.655111 0.048647

N 2.082749 0.163658 1.130823

RI-B2KPLYP/ma-def2-TZVP

Imaginary frequency = 352.9i cm^-1^

Total electronic energy = -504.827811 Ha

Zero-point correction = 0.110753 Ha

Thermal correction to enthalpy= 0.119313 Ha

Thermal correction to free energy= 0.078074 Ha

C -2.481816 -0.884491 0.060386

C -1.217593 -1.301358 -0.137578

C -0.148392 -0.325608 -0.226818

C -0.474692 1.088842 -0.196028

C -1.760221 1.457213 0.008129

C -2.778671 0.497451 0.153839

H -3.281935 -1.603960 0.138378

H -0.950210 -2.341697 -0.226015

H 0.316421 1.809513 -0.313723

H -2.012445 2.505497 0.053251

H -3.793943 0.815953 0.326932

N 1.163017 -0.827410 0.796063

N 1.123375 -0.782352 -0.656473

N 2.013106 0.197670 -1.043105

N 2.554871 0.653662 0.062751

N 2.050665 0.135604 1.115160


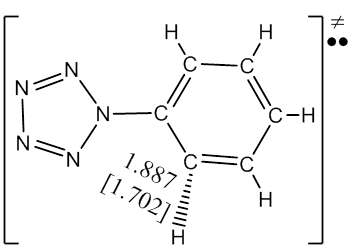


B3LYP/6-311++G**

Imaginary frequency = 900.1i cm^-1^

Total electronic energy = -505.249727 Ha

Zero-point correction = 0.102974 Ha

Thermal correction to enthalpy = 0.112376 Ha

Thermal correction to free energy = 0.067697 Ha

C 2.451240 -1.232696 -0.134068

C 1.077246 -1.167118 0.048100

C 0.357020 0.023084 0.002329

C 1.066409 1.224099 0.049457

C 2.459679 1.185241 0.012252

C 3.151931 -0.027180 -0.093676

H 2.961844 -2.182053 -0.246609

H 3.011046 2.117307 0.032908

H 4.233318 -0.031254 -0.170655

H 0.530454 2.164104 0.091778

N -1.058279 0.003254 -0.012235

N -1.831282 1.087428 0.016440

N -1.786485 -1.112027 -0.059045

N -3.015643 -0.711817 -0.061787

N -3.043628 0.638466 -0.016032

H 1.029415 -1.437822 1.914821

RI-B2KPLYP/ma-def2-TZVP

Imaginary frequency = 1586.8i cm^-1^

Total electronic energy = -504.817203 Ha

Zero-point correction = 0.106979 Ha

Thermal correction to enthalpy= 0.115765 Ha

Thermal correction to free energy= 0.073663 Ha

C 2.436320 -1.231507 -0.138561

C 1.069806 -1.164395 0.071719

C 0.358297 0.021815 0.007277

C 1.061589 1.205615 0.071979

C 2.444519 1.162147 0.049093

C 3.128832 -0.041778 -0.080192

H 2.939501 -2.175969 -0.276876

H 2.997172 2.087718 0.084435

H 4.204744 -0.042381 -0.169701

H 0.530709 2.144004 0.113498

N -1.048478 0.002118 -0.012391

N -1.811909 1.078292 0.004842

N -1.763489 -1.107347 -0.046766

N -2.993843 -0.710436 -0.053668

N -3.023600 0.627844 -0.022460

H 1.052968 -1.363307 1.762437


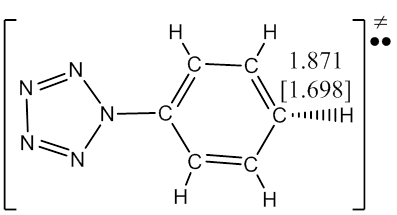


B3LYP/6-311++G**

Imaginary frequency = 944.3i cm^-1^

Total electronic energy = -505.251178 Ha

Zero-point correction = 0.102974 Ha

Thermal correction to enthalpy = 0.112345 Ha

Thermal correction to free energy = 0.067473 Ha

C 2.466715 -1.231113 -0.099471

C 1.076103 -1.227076 -0.062765

C 0.402227 -0.000002 -0.024185

C 1.076101 1.227074 -0.062758

C 2.466712 1.231116 -0.099464

C 3.105205 0.000002 0.014747

H 3.017042 -2.162262 -0.160911

H 3.017036 2.162266 -0.160900

H 3.358333 -0.000003 1.868556

H 0.515212 2.152120 -0.090603

N -1.022873 -0.000001 0.000826

N -1.775201 1.098412 0.013321

N -1.775203 -1.098413 0.013336

N -2.997735 -0.674251 0.031824

N -2.997734 0.674252 0.031815

H 0.515217 -2.152123 -0.090616

RI-B2KPLYP/ma-def2-TZVP

Imaginary frequency = 1620.2i cm^-1^

Total electronic energy = -504.818845 Ha

Zero-point correction = 0.106285 Ha

Thermal correction to enthalpy= 0.114371 Ha

Thermal correction to free energy= 0.074100 Ha

C 2.460133 -1.225068 -0.093064

C 1.084576 -1.217998 -0.058501

C 0.420551 -0.000004 -0.008534

C 1.084547 1.218004 -0.058373

C 2.460109 1.225108 -0.092943

C 3.092596 0.000024 0.054162

H 3.010167 -2.149908 -0.166925

H 3.010129 2.149964 -0.166711

H 3.220616 -0.000046 1.747180

H 0.520796 2.136030 -0.103015

N -0.997518 -0.000020 0.015566

N -1.738701 1.091085 0.027313

N -1.738672 -1.091131 0.027885

N -2.961405 -0.668274 0.045159

N -2.961422 0.668207 0.044812

H 0.520845 -2.136033 -0.103235


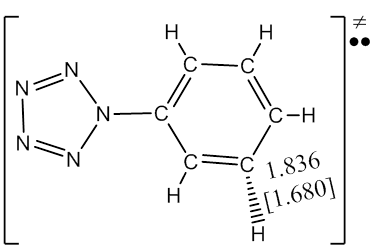


B3LYP/6-311++G**

Imaginary frequency = 968.0i cm^-1^

Total electronic energy = -505.250246 Ha

Zero-point correction = 0.102933 Ha

Thermal correction to enthalpy = 0.112297 Ha

Thermal correction to free energy = 0.067421 Ha

C 2.424879 -1.200108 -0.064542

C 1.039608 -1.252438 -0.161509

C 0.386142 -0.029939 -0.009523

C 1.092226 1.173581 0.096449

C 2.486547 1.163603 0.016435

C 3.185163 -0.034946 -0.127272

H 2.590927 -1.526862 1.734856

H 3.026957 2.103101 0.044520

H 4.264442 -0.054901 -0.218819

H 0.550654 2.103743 0.198763

N -1.041973 -0.001304 -0.002792

N -1.774024 1.109383 -0.048056

N -1.812302 -1.084864 0.047808

N -3.026916 -0.640648 0.031700

N -3.003335 0.707786 -0.024699

H 0.489485 -2.176073 -0.287273

RI-B2KPLYP/ma-def2-TZVP

Imaginary frequency = 1617.6i cm^-1^

Total electronic energy = -504.817863 Ha

Zero-point correction = 0.106707 Ha

Thermal correction to enthalpy= 0.115531 Ha

Thermal correction to free energy= 0.073201 Ha

C 2.414763 -1.199176 -0.026619

C 1.036729 -1.253799 -0.148310

C 0.397816 -0.043289 0.003720

C 1.094888 1.150222 0.114210

C 2.478668 1.143728 0.014462

C 3.171051 -0.040106 -0.123916

H 2.529801 -1.403980 1.637298

H 3.012758 2.082080 0.020542

H 4.244037 -0.062177 -0.230858

H 0.553002 2.075051 0.219246

N -1.023691 -0.009049 0.001652

N -1.739590 1.097842 -0.031733

N -1.787910 -1.082962 0.034227

N -3.000350 -0.634317 0.018537

N -2.970790 0.702976 -0.019497

H 0.488878 -2.172662 -0.280869


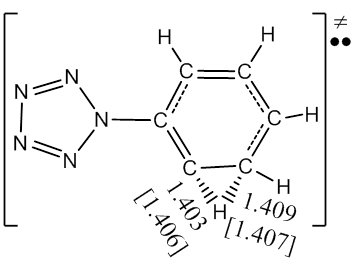


B3LYP/6-311++G**

Imaginary frequency = 1893.1i cm^-1^

Total electronic energy = -505.237558 Ha

Zero-point correction = 0.104291 Ha

Thermal correction to enthalpy = 0.113371 Ha

Thermal correction to free energy = 0.069379 Ha

C 2.468632 -1.232911 -0.048696

C 1.056489 -1.160928 -0.020002

C 0.319775 0.009101 -0.024908

C 1.075915 1.235682 0.062464

C 2.450646 1.197551 0.006988

C 3.188093 -0.009667 -0.087541

H 2.991899 2.136557 0.030223

H 4.268730 -0.022636 -0.096359

H 0.535853 2.170202 0.140831

N -1.074183 -0.007423 -0.011195

N -1.840504 1.084634 -0.014125

N -1.813107 -1.124304 -0.004575

N -3.045760 -0.704607 -0.008717

N -3.065330 0.631043 -0.014374

H 1.766159 -1.477741 1.147990

H 2.952236 -2.194749 -0.181617

RI-B2KPLYP/ma-def2-TZVP

Imaginary frequency = 1877.9i cm^-1^

Total electronic energy = -504.803899 Ha

Zero-point correction = 0.107096 Ha

Thermal correction to enthalpy= 0.115943 Ha

Thermal correction to free energy= 0.073514 Ha

C 2.462665 -1.226634 -0.052691

C 1.056777 -1.159147 -0.031561

C 0.327184 0.006386 -0.051041

C 1.071411 1.222458 0.061314

C 2.436733 1.188316 0.011567

C 3.173322 -0.013423 -0.100639

H 2.973672 2.123985 0.052047

H 4.249350 -0.021986 -0.102923

H 0.532737 2.152089 0.152247

N -1.065914 -0.009562 -0.021664

N -1.817901 1.075465 -0.021531

N -1.793531 -1.114020 -0.004348

N -3.024115 -0.698161 0.003822

N -3.040924 0.630614 -0.006216

H 1.764125 -1.453639 1.146800

H 2.943033 -2.186863 -0.170838


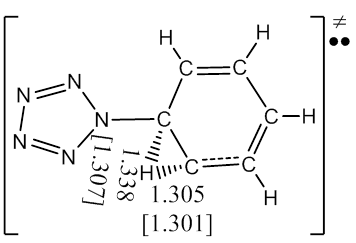


B3LYP/6-311++G**

Imaginary frequency = 2302.7i cm^-1^

Total electronic energy = -505.226115 Ha

Zero-point correction = 0.105245 Ha

Thermal correction to enthalpy = 0.114447 Ha

Thermal correction to free energy = 0.070226 Ha

C 1.101498 -1.204827 0.231671

C 0.321153 0.033471 0.273869

C 1.103922 1.266061 0.085611

C 2.434159 1.186056 -0.155796

C 3.152202 -0.069988 -0.225140

C 2.473942 -1.256965 -0.018576

H 4.218141 -0.074349 -0.410991

H 2.991023 -2.210159 -0.016010

H 2.989934 2.105177 -0.304055

H 0.710774 -0.659055 1.350356

H 0.573081 2.208580 0.119697

N -1.052572 0.006467 0.045934

N -1.855727 1.079586 0.119568

N -3.055963 0.600561 -0.055987

N -2.996484 -0.723950 -0.216360

N -1.754140 -1.118812 -0.162988

RI-B2KPLYP/ma-def2-TZVP

Imaginary frequency = 2617.0i cm^-1^

Total electronic energy = -504.790165 Ha

Zero-point correction = 0.108789 Ha

Thermal correction to enthalpy= 0.117806 Ha

Thermal correction to free energy= 0.074829 Ha

C 1.094150 -1.212636 0.229398

C 0.328559 0.040018 0.290114

C 1.093096 1.248836 0.070076

C 2.416761 1.182917 -0.140534

C 3.139694 -0.081235 -0.196249

C 2.473183 -1.246401 -0.025932

H 4.199732 -0.081397 -0.388956

H 2.982156 -2.197796 -0.070929

H 2.967502 2.099120 -0.287549

H 0.739849 -0.627159 1.336239

H 0.560592 2.187199 0.079751

N -1.050485 0.009906 0.064596

N -1.837459 1.072572 0.114253

N -3.035089 0.601057 -0.070158

N -2.975276 -0.717843 -0.214657

N -1.734692 -1.104448 -0.136439


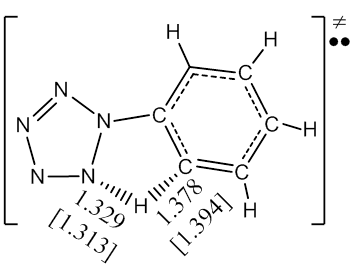


B3LYP/6-311++G**

Imaginary frequency = 2352.9i cm^-1^

Total electronic energy = -505.254571 Ha

Zero-point correction = 0.104389 Ha

Thermal correction to enthalpy = 0.113333 Ha

Thermal correction to free energy = 0.069967 Ha

C 2.566446 1.060669 -0.117265

C 1.221702 1.347956 0.062378

C 0.354926 0.258336 0.206184

C 0.837034 -1.056119 0.039146

C 2.189391 -1.349117 -0.033875

C 3.054973 -0.266291 -0.170953

H 3.271418 1.879769 -0.202478

H 2.558097 -2.367396 -0.073915

H -0.418273 -1.591954 -0.149312

N -1.008288 0.265172 0.450707

N -1.543962 -1.022939 0.268174

N -1.946633 1.181398 -0.010011

N -2.972188 0.450071 -0.362189

N -2.774960 -0.847415 -0.244331

H 0.851743 2.363447 0.128021

H 4.112396 -0.440482 -0.332449

RI-B2KPLYP/ma-def2-TZVP

Imaginary frequency = 411.9i cm^-1^

Total electronic energy = -504.809959 Ha

Zero-point correction = 0.115308 Ha

Thermal correction to enthalpy= 0.123642 Ha

Thermal correction to free energy= 0.082727 Ha

C 2.549242 1.046970 -0.135927

C 1.210901 1.327108 0.039461

C 0.356304 0.239115 0.204435

C 0.844524 -1.077766 0.045495

C 2.204890 -1.352797 -0.007265

C 3.050413 -0.274415 -0.163762

H 3.243977 1.866405 -0.242750

H 2.582774 -2.363771 -0.002623

H -0.419182 -1.575830 -0.268764

N -0.995124 0.236733 0.373103

N -1.526464 -1.000653 0.138959

N -1.949147 1.183865 0.098210

N -2.994682 0.490607 -0.205863

N -2.804630 -0.796428 -0.199780

H 0.831329 2.336621 0.070784

H 4.107725 -0.436777 -0.306817


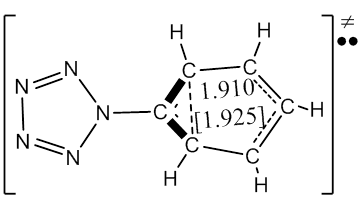


B3LYP/6-311++G**

Imaginary frequency = 377.0i cm^-1^

Total electronic energy = -505.275710 Ha

Zero-point correction = 0.108376 Ha

Thermal correction to enthalpy = 0.117309 Ha

Thermal correction to free energy = 0.073601 Ha

C 2.508265 1.155260 -0.031274

C 1.139537 0.954770 0.414842

C 0.293437 -0.000007 -0.258031

C 1.139539 -0.954771 0.414855

C 2.508271 -1.155257 -0.031254

C 3.213153 0.000002 -0.350764

H 0.699889 -1.602004 1.170108

H 2.931512 -2.152171 -0.060762

H 4.217180 0.000001 -0.752030

N -1.085393 -0.000004 -0.115794

N -1.842176 1.106080 -0.083412

N -3.064633 0.671604 -0.031302

N -3.064638 -0.671597 -0.031319

N -1.842185 -1.106082 -0.083439

H 2.931502 2.152176 -0.060794

H 0.699884 1.602002 1.170094

RI-B2KPLYP/ma-def2-TZVP

Imaginary frequency = 367.7i cm^-1^

Total electronic energy = -504.844616 Ha

Zero-point correction = 0.111757 Ha

Thermal correction to enthalpy= 0.120396 Ha

Thermal correction to free energy= 0.078280 Ha

C 2.497668 1.147139 -0.017985

C 1.121237 0.962493 0.366741

C 0.311983 -0.000033 -0.328061

C 1.121216 -0.962395 0.366980

C 2.497649 -1.147158 -0.017675

C 3.206149 -0.000054 -0.317742

H 0.651645 -1.609173 1.097779

H 2.927894 -2.136689 -0.022036

H 4.220139 -0.000110 -0.678944

N -1.069213 0.000008 -0.138345

N -1.809022 1.094881 -0.075240

N -3.028470 0.667125 0.030892

N -3.028493 -0.667005 0.031018

N -1.809061 -1.094825 -0.075032

H 2.927920 2.136666 -0.022636

H 0.651680 1.609446 1.097394


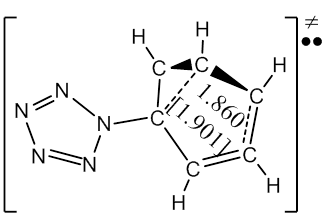


B3LYP/6-311++G**

Imaginary frequency = 376.0i cm^-1^

Total electronic energy = -505.268046 Ha

Zero-point correction = 0.108063 Ha

Thermal correction to enthalpy = 0.116936 Ha

Thermal correction to free energy = 0.073461 Ha

C 2.804288 0.090902 0.596096

C 1.758386 1.104201 0.534439

C 1.060978 1.403833 -0.701701

C 0.485276 0.109806 -0.387186

C 1.233568 -1.134951 -0.566445

C 2.533946 -1.089563 -0.090695

H 0.763058 -1.999354 -1.015635

H 3.240996 -1.899381 -0.204809

H 3.704425 0.266995 1.173004

H 1.585796 1.739637 1.402478

N -0.900294 0.000268 -0.107546

H 0.440438 2.283352 -0.805444

N -1.503910 -1.133028 0.267593

N -2.754689 -0.823438 0.391049

N -2.920801 0.483926 0.108308

N -1.776500 1.001327 -0.210353

RI-B2KPLYP/ma-def2-TZVP

Imaginary frequency = 297.6i cm^-1^

Total electronic energy = -504.839232 Ha

Zero-point correction = 0.111752 Ha

Thermal correction to enthalpy= 0.120310 Ha

Thermal correction to free energy= 0.078413 Ha

C -2.816414 -0.091703 0.573110

C -1.798851 -1.116935 0.540060

C -1.051545 -1.397224 -0.665673

C -0.475024 -0.110192 -0.381435

C -1.215301 1.120341 -0.533629

C -2.524664 1.077690 -0.099363

H -0.729562 1.997669 -0.928922

H -3.216242 1.893812 -0.218023

H -3.727344 -0.251212 1.129651

H -1.644466 -1.742922 1.412158

N 0.908038 0.002734 -0.125431

H -0.419819 -2.267012 -0.733356

N 1.496551 1.119682 0.275310

N 2.750424 0.820030 0.375922

N 2.922754 -0.466393 0.052544

N 1.779378 -0.981705 -0.266715


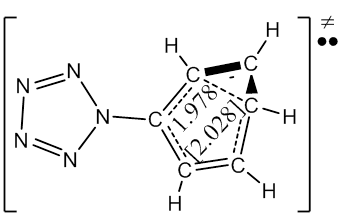


B3LYP/6-311++G**

Imaginary frequency = 435.2i cm^-1^

Total electronic energy = -505.268704 Ha

Zero-point correction = 0.107796 Ha

Thermal correction to enthalpy = 0.116818 Ha

Thermal correction to free energy = 0.073056 Ha

C -2.394490 1.221374 0.107119

C -2.900548 -0.121399 0.297742

C -2.450780 -1.227245 -0.536794

C -1.180458 -1.083130 0.128830

C -0.395118 0.112323 -0.064689

C -1.057010 1.347594 -0.199608

H -0.750416 -1.897189 0.706449

H -0.553437 2.271499 -0.438971

H -3.052982 2.074147 0.230533

H -3.652354 -0.302973 1.064070

H -2.962001 -2.181510 -0.502875

N 1.000341 0.026700 -0.016529

N 1.828930 1.080571 -0.035275

N 3.015628 0.578736 0.014865

N 2.929929 -0.773026 0.069593

N 1.688260 -1.121706 0.045231

RI-B2KPLYP/ma-def2-TZVP

Imaginary frequency = 907.9i cm^-1^

Total electronic energy = -504.836303 Ha

Zero-point correction = 0.110787 Ha

Thermal correction to enthalpy= 0.119651 Ha

Thermal correction to free energy= 0.077200 Ha

C -2.371005 1.209924 0.118898

C -2.902207 -0.096464 0.289268

C -2.453529 -1.220614 -0.495925

C -1.154041 -1.104974 0.085663

C -0.405903 0.083829 -0.101596

C -1.051483 1.321540 -0.251575

H -0.682723 -1.934631 0.595388

H -0.537166 2.237046 -0.476944

H -2.986110 2.071788 0.333331

H -3.675494 -0.249078 1.034573

H -2.964552 -2.164755 -0.390580

N 0.986077 0.018013 -0.021210

N 1.785732 1.079193 -0.013585

N 2.976702 0.603586 0.052806

N 2.914163 -0.745197 0.088483

N 1.683737 -1.110378 0.037784


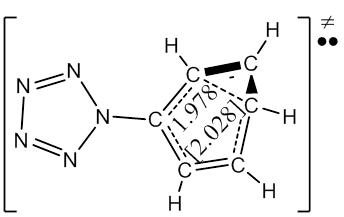


B3LYP/6-311++G**

Imaginary frequency = 412.9i cm^-1^

Total electronic energy = -505.266431 Ha

Zero-point correction = 0.107774 Ha

Thermal correction to enthalpy = 0.116796 Ha

Thermal correction to free energy = 0.072681 Ha

C 1.087053 1.165768 -0.193788

C 2.516407 0.959423 -0.162563

C 3.133925 -0.000016 0.733474

C 2.516404 -0.959415 -0.162610

C 1.087047 -1.165764 -0.193836

C 0.331946 -0.000003 -0.091542

H 0.654774 2.147680 -0.336340

H 3.149375 1.618619 -0.753712

H 4.208402 -0.000019 0.871925

H 3.149371 -1.618560 -0.753814

H 0.654770 -2.147672 -0.336421

N -1.078666 -0.000001 0.001675

N -1.826562 1.099034 0.035808

N -3.052206 0.671183 0.087119

N -3.052205 -0.671182 0.087164

N -1.826560 -1.099034 0.035884

RI-B2KPLYP/ma-def2-TZVP

Imaginary frequency = 355.0i cm^-1^

Total electronic energy = -504.837075 Ha

Zero-point correction = 0.111891 Ha

Thermal correction to enthalpy= 0.120528 Ha

Thermal correction to free energy= 0.078414 Ha

C -1.083547 -1.158384 -0.192711

C -2.504041 -0.969819 -0.161662

C -3.119029 0.000096 0.711797

C -2.504028 0.969795 -0.161891

C -1.083530 1.158343 -0.192956

C -0.338191 -0.000012 -0.081012

H -0.644730 -2.132037 -0.342379

H -3.130564 -1.633241 -0.745797

H -4.190268 0.000118 0.835896

H -3.130544 1.633043 -0.746230

H -0.644701 2.131959 -0.342836

N 1.066566 -0.000004 0.006895

N 1.803138 -1.091689 0.036995

N 3.029342 -0.665434 0.082786

N 3.029323 0.665466 0.082934

N 1.803106 1.091696 0.037245


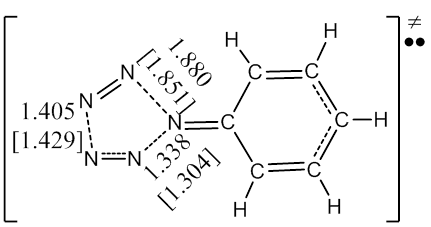


B3LYP/6-311++G**

Imaginary frequency = 377.2i cm^-1^

Total electronic energy = -505.318202 Ha

Zero-point correction = 0.107269 Ha

Thermal correction to enthalpy = 0.116857 Ha

Thermal correction to free energy = 0.071765 Ha

C 2.544631 -1.172130 -0.065373

C 1.177399 -1.258284 -0.017532

C 0.393767 -0.039492 0.046838

C 1.072893 1.239014 0.059333

C 2.440288 1.276745 0.016516

C 3.195693 0.081293 -0.047236

H 3.136175 -2.079047 -0.116484

H 0.654015 -2.204325 -0.028869

H 0.471704 2.137483 0.105495

H 2.952329 2.231695 0.028515

H 4.277097 0.127957 -0.083325

N -0.918111 -0.050559 0.095756

N -2.202064 1.312547 -0.070917

N -3.135438 0.563218 -0.077710

N -2.962658 -0.830785 -0.066106

N -1.701635 -1.133941 0.138892

RI-B2KPLYP/ma-def2-TZVP

Imaginary frequency = 397.4i cm^-1^

Total electronic energy = -504.868001 Ha

Zero-point correction = 0.111670 Ha

Thermal correction to enthalpy= 0.121012 Ha

Thermal correction to free energy= 0.077664 Ha

C -2.511210 -1.175812 0.118016

C -1.169052 -1.266970 0.030597

C -0.382138 -0.042070 -0.099759

C -1.071129 1.243600 -0.120266

C -2.413141 1.274436 -0.035245

C -3.164950 0.077387 0.082699

H -3.103331 -2.073124 0.216220

H -0.643964 -2.207601 0.052187

H -0.470144 2.134299 -0.207675

H -2.931420 2.220963 -0.053079

H -4.239734 0.123327 0.149356

N 0.901726 -0.047591 -0.192642

N 2.124964 1.278147 0.223594

N 3.076199 0.570536 0.207107

N 2.915678 -0.839135 0.035155

N 1.691686 -1.081865 -0.277526


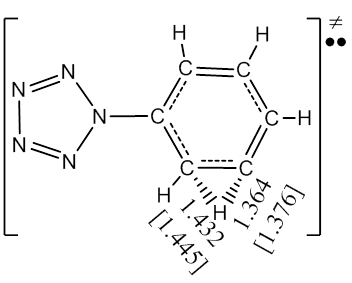


B3LYP/6-311++G**

Imaginary frequency = 1841.2i cm^-1^

Total electronic energy = -505.240489 Ha

Zero-point correction = 0.104419 Ha

Thermal correction to enthalpy = 0.113463 Ha

Thermal correction to free energy = 0.069608 Ha

C 2.499622 1.155833 0.018990

C 1.126207 1.192593 0.005004

C 0.361795 -0.017133 -0.069056

C 1.037076 -1.261702 -0.034752

C 2.450934 -1.209683 -0.013458

C 3.236354 -0.071688 -0.066321

H 3.052255 2.087758 0.063814

H 0.600054 2.136959 0.032659

H 0.505644 -2.202950 -0.113661

H 4.315362 -0.098205 -0.104462

N -1.034321 0.006254 -0.024437

N -1.809112 -1.084973 -0.041458

N -3.030455 -0.626976 -0.004131

N -3.007115 0.704981 0.034383

N -1.768691 1.121907 0.025354

H 1.802610 -1.501229 1.151235

RI-B2KPLYP/ma-def2-TZVP

Imaginary frequency = 1818.5i cm^-1^

Total electronic energy = -504.807687 Ha

Zero-point correction = 0.106718 Ha

Thermal correction to enthalpy= 0.115594 Ha

Thermal correction to free energy= 0.073019 Ha

C 2.489276 1.147062 0.025184

C 1.121188 1.180656 0.015202

C 0.371254 -0.021949 -0.089644

C 1.031972 -1.255975 -0.042642

C 2.439112 -1.203720 -0.021777

C 3.220506 -0.072843 -0.086372

H 3.036080 2.076158 0.086685

H 0.594894 2.119536 0.065437

H 0.504097 -2.195248 -0.108590

H 4.294744 -0.101727 -0.126219

N -1.027333 0.004273 -0.036632

N -1.789885 -1.074963 -0.033151

N -3.008364 -0.622348 0.006884

N -2.982829 0.704170 0.027340

N -1.746076 1.110883 0.001608

H 1.795833 -1.474293 1.163932


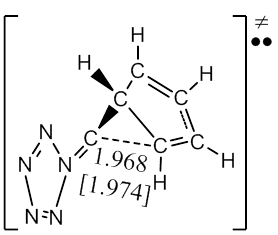


B3LYP/6-311++G**

Imaginary frequency = 429.4i cm^-1^

Total electronic energy = -505.254147 Ha

Zero-point correction = 0.108521 Ha

Thermal correction to enthalpy = 0.117452 Ha

Thermal correction to free energy = 0.073105 Ha

C -1.597803 1.243294 -0.291490

C -1.427084 -0.007841 -1.103942

C -0.185513 -0.737049 -0.788780

C -2.038030 -1.078351 -0.219311

C -2.443086 -0.488014 0.966452

C -2.184356 0.924358 0.895990

H -2.195216 -2.097080 -0.537731

H -2.857000 -1.008197 1.818596

H -2.402914 1.626718 1.690002

N 0.996645 -0.313479 -0.288543

N 1.519034 0.941802 -0.412469

N 2.670815 0.878786 0.157825

N 2.892784 -0.375243 0.616832

N 1.869977 -1.118620 0.360306

H -1.231032 2.210042 -0.601729

H -1.703385 0.037414 -2.160303

RI-B2KPLYP/ma-def2-TZVP

Imaginary frequency = 585.0i cm^-1^

Total electronic energy = -504.820380 Ha

Zero-point correction = 0.112119 Ha

Thermal correction to enthalpy= 0.120864 Ha

Thermal correction to free energy= 0.077612 Ha

C -1.586749 1.228439 -0.392153

C -1.516231 -0.023894 -1.186829

C -0.212820 -0.706598 -1.002538

C -1.990199 -1.076501 -0.227348

C -2.209540 -0.490301 0.984057

C -1.976248 0.928509 0.857500

H -2.144475 -2.103394 -0.505438

H -2.472954 -1.001295 1.893647

H -2.075217 1.636482 1.664232

N 0.893416 -0.290976 -0.325454

N 1.505581 0.890968 -0.487730

N 2.520447 0.854280 0.295402

N 2.557350 -0.337417 0.928909

N 1.552657 -1.046021 0.557192

H -1.295383 2.192248 -0.770277

H -1.884313 -0.016351 -2.207223


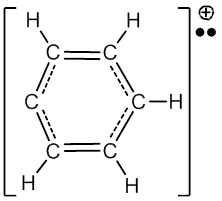


B3LYP/6-311++G**

Total electronic energy = -231.284900 Ha

Zero-point correction = 0.085337 Ha

Thermal correction to enthalpy = 0.091070 Ha

Thermal correction to free energy = 0.056605 Ha

C -1.254192 0.617164 -0.000142

C -1.264851 -0.757050 0.000055

C 0.000311 -1.376962 0.000123

C 1.265396 -0.756715 0.000024

C 1.253757 0.617751 -0.000400

C -0.000370 1.307416 0.000161

H -2.177656 1.184793 -0.001072

H -2.180732 -1.337122 0.000307

H 2.181546 -1.336339 0.000128

H 2.176636 1.186364 0.000107

H -0.000096 2.392677 0.001607

RI-B2KPLYP/ma-def2-TZVP

Total electronic energy = -231.053800 Ha

Zero-point correction = 0.087938 Ha

Thermal correction to enthalpy= 0.093441 Ha

Thermal correction to free energy= 0.060395 Ha

C -1.247264 0.615068 -0.000019

C -1.259645 -0.752385 -0.000079

C 0.000356 -1.368832 -0.000004

C 1.260075 -0.751825 0.000082

C 1.246941 0.615617 0.000173

C -0.000330 1.298959 0.000209

H -2.165627 1.182181 -0.000103

H -2.170958 -1.330374 -0.000168

H 2.171685 -1.329341 0.000092

H 2.164949 1.183307 0.000261

H -0.000433 2.379601 0.000456


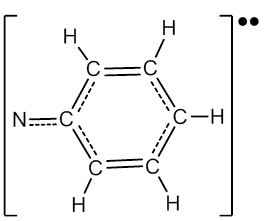


B3LYP/6-311++G**

Total electronic energy = -286.3822119 Ha

Zero-point correction = 0.090726 Ha

Thermal correction to enthalpy = 0.096996 Ha

Thermal correction to free energy = 0.060865 Ha

C -1.044263 -1.215034 -0.000084

C 0.336928 -1.232018 0.000024

C 1.071017 0.000036 -0.000229

C 0.336501 1.232384 -0.000178

C -1.044215 1.215043 0.000094

C -1.744066 -0.000289 0.000006

H -1.591143 -2.151213 0.000089

H 0.890015 -2.163106 0.000096

H 0.889934 2.163260 0.000049

H -1.591733 2.150848 0.000229

H -2.827593 -0.000167 0.000356

N 2.394158 -0.000052 0.000197

RI-B2KPLYP/ma-def2-TZVP

Total electronic energy = -286.101531 Ha

Zero-point correction = 0.092918 Ha

Thermal correction to enthalpy= 0.099036 Ha

Thermal correction to free energy= 0.064225 Ha

C -2.552384 -0.751049 0.263344

C -1.319348 -1.310122 0.047309

C -0.208064 -0.481251 -0.225562

C -0.391038 0.919138 -0.270710

C -1.634105 1.454015 -0.051120

C -2.718818 0.627524 0.215752

H -3.398799 -1.387149 0.473090

H -1.173763 -2.378562 0.081341

H 0.461390 1.546246 -0.480346

H -1.770363 2.524240 -0.086068

H -3.693189 1.057672 0.388514

N 0.994057 -1.012517 -0.435736


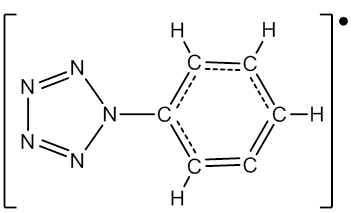


B3LYP/6-311++G**

Total electronic energy = -504.757113 Ha

Zero-point correction = 0.101490 Ha

Thermal correction to enthalpy = 0.110186 Ha

Thermal correction to free energy = 0.066665 Ha

C 2.435867 -1.245025 -0.001183

C 1.065604 -1.292682 -0.001015

C 0.421730 -0.047478 0.000205

C 1.140253 1.148743 0.001165

C 2.532279 1.110268 0.000999

C 3.215713 -0.113982 -0.000253

H 3.091192 2.039262 0.001798

H 0.611872 2.092090 0.002131

N -1.006411 -0.005016 -0.000053

N -1.727583 1.113528 -0.002097

N -1.787671 -1.081670 0.002027

N -2.998413 -0.625073 0.001209

N -2.961334 0.723471 -0.001265

H 0.499507 -2.215316 -0.001661

H 4.298649 -0.151785 -0.000523

RI-B2KPLYP/ma-def2-TZVP

Total electronic energy = -504.336652 Ha

Zero-point correction = 0.104566 Ha

Thermal correction to enthalpy= 0.113015 Ha

Thermal correction to free energy= 0.071287 Ha

C 2.331525 -0.996621 -0.513374

C 0.975461 -1.086308 -0.435489

C 0.311638 0.126483 -0.310033

C 0.986593 1.331361 -0.269667

C 2.365894 1.334960 -0.356464

C 3.074452 0.145782 -0.481017

H 2.897130 2.274471 -0.324553

H 0.434731 2.251049 -0.169538

N -1.107054 0.124944 -0.217980

N -1.847448 1.213193 -0.135260

N -1.844081 -0.967919 -0.201062

N -3.064844 -0.550140 -0.106223

N -3.066718 0.786275 -0.065595

H 0.436112 -2.019070 -0.468008

H 4.151007 0.139469 -0.548671


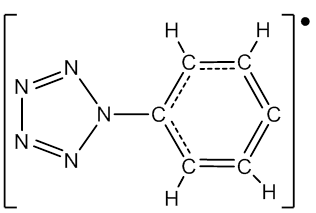


B3LYP/6-311++G**

Total electronic energy = -504.756990 Ha

Zero-point correction = 0.101540 Ha

Thermal correction to enthalpy = 0.110245 Ha

Thermal correction to free energy = 0.066257 Ha

C -2.520064 -1.223799 0.002818

C -1.121528 -1.222821 0.003058

C -0.448212 -0.000040 0.000043

C -1.121442 1.222803 -0.002831

C -2.519966 1.223842 -0.002946

C -3.145425 0.000041 -0.000158

H -3.067900 2.158683 -0.005093

H -0.564202 2.150389 -0.004925

N 0.981871 -0.000037 0.000195

N 1.732795 1.097400 0.006179

N 1.732852 -1.097431 -0.006069

N 2.956563 -0.673726 -0.003844

N 2.956524 0.673765 0.003539

H -0.564288 -2.150406 0.005194

H -3.068018 -2.158623 0.004920

RI-B2KPLYP/ma-def2-TZVP

Total electronic energy = -504.336548 Ha

Zero-point correction = 0.104592 Ha

Thermal correction to enthalpy= 0.112171 Ha

Thermal correction to free energy= 0.073037 Ha

C -2.507610 1.226480 0.137617

C -1.120804 1.234706 0.089583

C -0.454258 0.042990 -0.124714

C -1.114765 -1.158866 -0.297275

C -2.501786 -1.171636 -0.258306

C -3.123041 0.022777 -0.040784

H -3.046676 -2.092803 -0.392731

H -0.554410 -2.065482 -0.461541

N 0.967456 0.054045 -0.169065

N 1.700000 -0.926147 -0.658313

N 1.713366 1.046181 0.273624

N 2.934025 0.674172 0.053247

N 2.925986 -0.534302 -0.514697

H -0.564739 2.149581 0.218938

H -3.057094 2.139222 0.306308


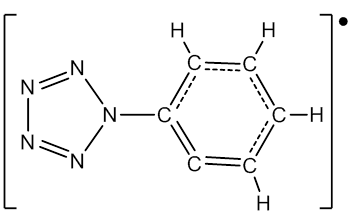


B3LYP/6-311++G**

Total electronic energy = -504.755031 Ha

Zero-point correction = 0.101540 Ha

Thermal correction to enthalpy = 0.110252 Ha

Thermal correction to free energy = 0.066896 Ha

C 2.454449 -1.269432 -0.000060

C 1.087385 -1.184782 -0.000196

C 0.376341 -0.006789 0.000007

C 1.088357 1.198501 0.000212

C 2.480229 1.155846 0.000131

C 3.165482 -0.059830 -0.000036

H 3.034956 2.086218 0.000046

H 0.552286 2.139238 0.000254

N -1.046479 -0.015132 -0.000022

N -1.806557 1.075915 -0.000298

N -1.785063 -1.122596 0.000209

N -3.011044 -0.710423 0.000188

N -3.025030 0.639754 -0.000130

H 4.249523 -0.075656 -0.000065

H 2.968987 -2.223512 -0.000206

RI-B2KPLYP/ma-def2-TZVP

Total electronic energy = -504.333811 Ha

Zero-point correction = 0.104598 Ha

Thermal correction to enthalpy= 0.113061Ha

Thermal correction to free energy= 0.071453 Ha

C 2.449173 -0.968135 -0.623305

C 1.092649 -0.894682 -0.574365

C 0.381357 0.266022 -0.496271

C 1.076075 1.465264 -0.462475

C 2.456754 1.435475 -0.510106

C 3.143294 0.236836 -0.589185

H 3.003257 2.365139 -0.483392

H 0.533932 2.395813 -0.398977

N -1.033314 0.246261 -0.449281

N -1.788830 1.324617 -0.397702

N -1.752535 -0.858832 -0.450386

N -2.981055 -0.456921 -0.397960

N -3.003836 0.880191 -0.366060

H 4.222405 0.231527 -0.625655

H 2.966980 -1.912658 -0.687612


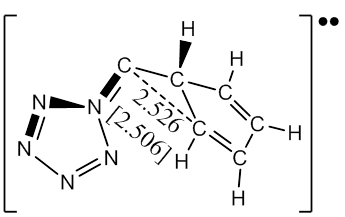


B3LYP/6-311++G**

Total electronic energy = -505.265936 Ha

Zero-point correction = 0.109313 Ha

Thermal correction to enthalpy = 0.118880 Ha

Thermal correction to free energy = 0.072679 Ha

C -1.855021 0.254340 1.191618

C -1.330791 1.047327 -0.000180

C 0.121146 1.281527 -0.000185

C -1.854961 0.253962 -1.191753

C -2.589983 -0.772584 -0.733906

C -2.590019 -0.772352 0.734061

H -1.652741 0.525871 -2.217370

H -3.112925 -1.496934 -1.344639

H -3.112992 -1.496509 1.344996

N 1.174668 0.443583 -0.000048

N 1.117455 -0.917639 0.000163

N 2.355447 -1.291918 0.000138

N 3.166753 -0.220561 0.000154

N 2.463256 0.865150 -0.000083

H -1.652851 0.526572 2.217159

H -1.813775 2.037378 -0.000349

RI-B2KPLYP/ma-def2-TZVP

Total electronic energy = -504.837232 Ha

Zero-point correction = 0.112179 Ha

Thermal correction to enthalpy= 0.120664 Ha

Thermal correction to free energy= 0.078649 Ha

C -1.828724 0.268688 1.182575

C -1.337040 1.071338 -0.000180

C 0.105710 1.335821 -0.000185

C -1.828665 0.268324 -1.182714

C -2.522168 -0.783963 -0.730271

C -2.522204 -0.783739 0.730420

H -1.639218 0.546420 -2.205056

H -3.011547 -1.524444 -1.341315

H -3.011612 -1.524032 1.341668

N 1.142999 0.454235 -0.000038

N 1.020103 -0.877047 0.000140

N 2.232812 -1.316589 0.000260

N 3.086233 -0.283512 0.000077

N 2.422665 0.822002 -0.000101

H -1.639330 0.547098 2.204841

H -1.837352 2.046614 -0.000344


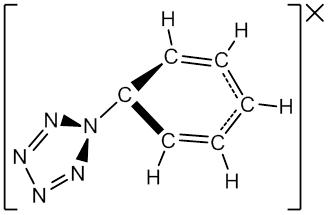


B3LYP/6-311++G**

Total electronic energy = -505.310915 Ha

Zero-point correction = 0.109161 Ha

Thermal correction to enthalpy = 0.118718 Ha

Thermal correction to free energy = 0.073511 Ha

C -2.182276 1.247688 0.066192

C -1.282732 1.202445 -0.956229

C -0.493468 -0.065819 -0.981485

C -1.274348 -1.325423 -0.789720

C -2.174323 -1.242225 0.229270

C -2.538068 0.041626 0.755481

H -2.728428 2.159131 0.288457

H -1.120087 2.005068 -1.663971

H -1.104884 -2.212995 -1.385288

H -2.715078 -2.120104 0.568651

H -3.251523 0.092953 1.569812

N 0.678220 -0.009648 -0.182557

N 1.358022 -1.087471 0.239908

N 2.425124 -0.619499 0.794934

N 2.420928 0.733383 0.706156

N 1.351272 1.118215 0.095093

RI-B2KPLYP/ma-def2-TZVP

Total electronic energy = -504.873685 Ha

Zero-point correction = 0.110912 Ha

Thermal correction to enthalpy= 0.118708 Ha

Thermal correction to free energy= 0.079808 Ha

C -2.133275 1.242365 0.055180

C -1.290770 1.189581 -0.988818

C -0.506404 -0.068511 -1.029539

C -1.281613 -1.316303 -0.823700

C -2.124973 -1.238018 0.217521

C -2.443049 0.042781 0.769547

H -2.676858 2.145014 0.293319

H -1.159975 1.981360 -1.707113

H -1.142620 -2.194904 -1.430974

H -2.663120 -2.105464 0.571006

H -3.100555 0.096191 1.622378

N 0.640943 -0.009205 -0.183533

N 1.291267 -1.075100 0.258902

N 2.336348 -0.611118 0.854214

N 2.334683 0.730519 0.764129

N 1.288321 1.108136 0.112184


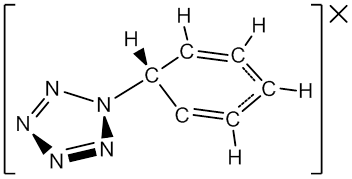


B3LYP/6-311++G**

Total electronic energy = -505.293617 Ha

Zero-point correction = 0.109044 Ha

Thermal correction to enthalpy = 0.118890 Ha

Thermal correction to free energy = 0.072854 Ha

C 1.098512 -1.182193 0.584799

C 0.396608 0.110095 0.674331

C 1.120855 1.294961 0.082284

C 2.369154 1.127285 -0.413964

C 3.029931 -0.137873 -0.347192

C 2.446614 -1.227207 0.315860

H 3.987533 -0.257579 -0.844220

H 3.008926 -2.149653 0.431589

H 2.900739 1.972981 -0.836112

H 0.663276 2.274695 0.174738

N -1.000597 0.026771 0.187834

N -2.063452 0.188644 0.962946

N -3.081530 0.036937 0.175096

N -2.636154 -0.215090 -1.073205

N -1.339145 -0.220936 -1.066513

H 0.265855 0.316661 1.753782

RI-B2KPLYP/ma-def2-TZVP

Total electronic energy = -504.862527 Ha

Zero-point correction = 0.110313 Ha

Thermal correction to enthalpy= 0.119601 Ha

Thermal correction to free energy= 0.076195 Ha

C 1.092470 -1.172714 0.598011

C 0.397376 0.116328 0.694780

C 1.111191 1.284848 0.084364

C 2.349956 1.122962 -0.407150

C 3.013691 -0.137333 -0.339095

C 2.425057 -1.229312 0.291350

H 3.977695 -0.246539 -0.814623

H 2.975722 -2.155373 0.379237

H 2.873175 1.964306 -0.835525

H 0.637483 2.254681 0.137294

N -0.980045 0.029673 0.203610

N -2.042117 0.200609 0.958892

N -3.049225 0.042969 0.157348

N -2.591081 -0.218783 -1.069746

N -1.293910 -0.226167 -1.044442

H 0.269687 0.328347 1.767745


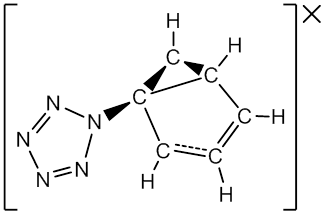


B3LYP/6-311++G**

Total electronic energy = -505.259838 Ha

Zero-point correction = 0.107392 Ha

Thermal correction to enthalpy = 0.117417 Ha

Thermal correction to free energy = 0.071018 Ha

C 2.806523 -0.199642 -0.420230

C 1.521239 -0.966343 -0.585485

C 1.094309 -1.390068 0.721123

C 0.554435 -0.039860 0.266436

C 1.252381 1.225703 0.458433

C 2.593373 1.054824 0.126734

H 0.767928 2.129338 0.797457

H 3.372491 1.776536 0.333713

H 3.767195 -0.666768 -0.594751

H 1.231422 -1.423111 -1.531384

N -0.863655 0.013165 0.024311

H 0.478157 -2.247988 0.958339

N -1.527088 1.130550 -0.262545

N -2.764154 0.765252 -0.392541

N -2.857186 -0.562669 -0.194904

N -1.678519 -1.035554 0.070003

RI-B2KPLYP/ma-def2-TZVP

Total electronic energy = -504.837023 Ha

Zero-point correction = 0.109507 Ha

Thermal correction to enthalpy = 0.118473 Ha

Thermal correction to free energy = 0.076624 Ha

C 2.786273 -0.193070 -0.432518

C 1.509938 -0.958447 -0.572284

C 1.079570 -1.377525 0.733720

C 0.555995 -0.044895 0.273181

C 1.250499 1.216664 0.472691

C 2.579714 1.047832 0.126169

H 0.771118 2.108880 0.833923

H 3.357049 1.768006 0.320187

H 3.741304 -0.643563 -0.647280

H 1.212973 -1.429871 -1.502019

N -0.850382 0.010361 0.027385

H 0.472064 -2.231074 0.985343

N -1.498942 1.122166 -0.261427

N -2.733954 0.758632 -0.412904

N -2.829390 -0.558218 -0.224210

N -1.654978 -1.032508 0.054750


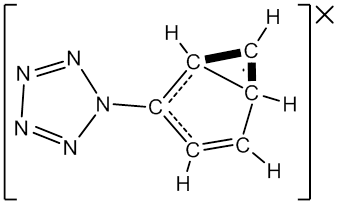


B3LYP/6-311++G**

Total electronic energy = -505.271309 Ha

Zero-point correction = 0.107446 Ha

Thermal correction to enthalpy = 0.117562 Ha

Thermal correction to free energy = 0.070750 Ha

C 2.776650 0.027394 -0.174815

C 1.503254 -0.754607 -0.358676

C 1.064084 -1.254075 0.915256

C 0.493340 0.096774 0.519684

C 1.193211 1.342456 0.750952

C 2.557732 1.244308 0.415957

H 3.307662 1.979900 0.669570

H 3.745028 -0.397533 -0.404130

H 1.242162 -1.175320 -1.328787

N 0.568609 2.481183 1.235659

H 0.470793 -2.142918 1.096696

N 1.161283 3.678636 1.362838

N 0.249506 4.452834 1.858653

N -0.891624 3.754221 2.026611

N -0.706074 2.528780 1.649637

H -0.579486 0.111751 0.361589

RI-B2KPLYP/ma-def2-TZVP

Total electronic energy = -504.846003 Ha

Zero-point correction = 0.109851 Ha

Thermal correction to enthalpy = 0.118835 Ha

Thermal correction to free energy = 0.076879 Ha

C 2.770637 0.034176 -0.175596

C 1.508311 -0.746379 -0.341489

C 1.038293 -1.216827 0.936099

C 0.491033 0.107323 0.487363

C 1.196766 1.345597 0.733015

C 2.551616 1.245760 0.411770

H 3.297745 1.981572 0.657874

H 3.735276 -0.375606 -0.425281

H 1.263301 -1.211480 -1.288204

N 0.574099 2.473826 1.235649

H 0.453116 -2.099862 1.136402

N 1.160803 3.654314 1.372414

N 0.249662 4.422511 1.877755

N -0.877858 3.724120 2.039481

N -0.685682 2.505439 1.646680

H -0.570985 0.129298 0.292765

The xyz coordinates of geometries in Fig. 4:

­­­___________________________________________________________


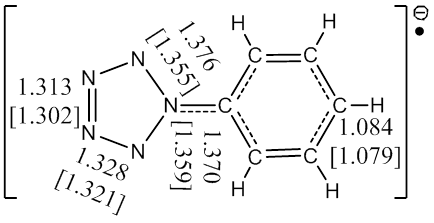


B3LYP/6-311++G**

Total electronic energy = -505.480153 Ha

Total electronic energy solvent (THF) = -505.549040 Ha

Total electronic energy CCSD(T)/cc-pVTZ = -504.476663 Ha

Total electronic energy HF/cc-pVTZ = -502.418837 Ha

Total electronic energy CCSD(T)/cc-pVDZ = -503.990189 Ha

Total electronic energy HF/cc-pVTZ = -502.291516 Ha

Total electronic energy CCSD(T)/CBS= -504.737717 Ha

Zero-point correction = 0.111045 Ha

Thermal correction to enthalpy = 0.120031 Ha

Thermal correction to free energy = 0.076991 Ha

C -2.460665 1.206307 -0.000023

C -1.075939 1.224142 -0.000025

C -0.351924 -0.000002 0.000000

C -1.075944 -1.224143 0.000025

C -2.460670 -1.206302 0.000024

C -3.181899 0.000004 0.000001

H -2.995365 2.152490 -0.000041

H -0.527131 2.156313 -0.000045

H -0.527140 -2.156316 0.000045

H -2.995374 -2.152483 0.000041

H -4.266228 0.000006 0.000001

N 1.018063 -0.000004 0.000000

N 1.801981 -1.130979 -0.000004

N 3.042800 -0.656461 -0.000226

N 3.042810 0.656467 0.000227

N 1.801987 1.130970 0.000002

RI-B2KPLYP/ma-def2-TZVP

Total electronic energy = -505.039975 Ha

Zero-point correction = 0.112860 Ha

Thermal correction to enthalpy= 0.121780 Ha

Thermal correction to free energy= 0.080060 Ha

C 2.444759 1.197602 0.000032

C 1.069284 1.218906 0.000032

C 0.353319 -0.000006 -0.000006

C 1.069287 -1.218916 -0.000044

C 2.444763 -1.197606 -0.000032

C 3.162630 -0.000001 0.000004

H 2.977649 2.139719 0.000058

H 0.521426 2.146925 0.000062

H 0.521433 -2.146937 -0.000075

H 2.977656 -2.139722 -0.000054

H 4.242031 0.000001 0.000007

N -1.006100 -0.000003 -0.000002

N -1.772805 -1.117470 0.000019

N -3.008809 -0.650968 0.000380

N -3.008796 0.650993 -0.000366

N -1.772788 1.117475 -0.000017


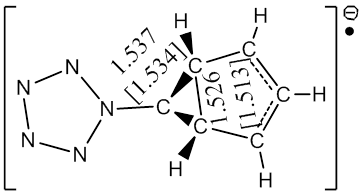


B3LYP/6-311++G**

Total electronic energy = -505.363949 Ha

Zero-point correction = 0.107718 Ha

Thermal correction to enthalpy = 0.117020 Ha

Thermal correction to free energy = 0.072745 Ha

C -2.504663 -1.148972 0.003650

C -1.155570 -0.763013 0.469173

C -0.291664 -0.000153 -0.548102

C -1.155520 0.763147 0.468852

C -2.504597 1.148984 0.003161

C -3.244482 -0.000029 -0.279868

H -0.652354 1.329719 1.248825

H -2.834307 2.168139 -0.160691

H -4.243632 -0.000096 -0.703393

N 1.082328 -0.000081 -0.205872

N 1.838508 -1.099839 -0.101557

N 3.074493 -0.659880 0.048704

N 3.074382 0.659993 0.048904

N 1.838301 1.099793 -0.100956

H -2.834395 -2.168170 -0.159897

H -0.652414 -1.329275 1.249381

RI-B2KPLYP/ma-def2-TZVP

Total electronic energy = -504.933517 Ha

Zero-point correction = 0.110508 Ha

Thermal correction to enthalpy= 0.119495 Ha

Thermal correction to free energy= 0.077042 Ha

C -2.490993 -1.140111 0.015987

C -1.146058 -0.756574 0.459453

C -0.314512 -0.000109 -0.584975

C -1.146058 0.756884 0.459044

C -2.490988 1.140167 0.015322

C -3.227595 -0.000051 -0.261169

H -0.617524 1.325069 1.212943

H -2.820714 2.154442 -0.146228

H -4.224874 -0.000165 -0.675992

N 1.057669 -0.000041 -0.217180

N 1.802340 -1.090785 -0.097421

N 3.030278 -0.658359 0.083010

N 3.030132 0.658396 0.083746

N 1.802270 1.090743 -0.097435

H -2.820744 -2.154484 -0.144893

H -0.617500 -1.324353 1.213639


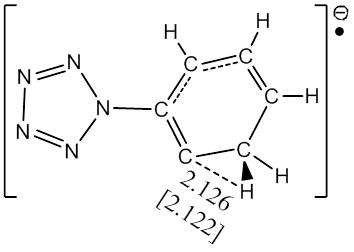


B3LYP/6-311++G**

Total electronic energy = -505.385245 Ha

Zero-point correction = 0.108025 Ha

Thermal correction to enthalpy = 0.117610 Ha

Thermal correction to free energy = 0.072423 Ha

C -2.512923 -1.210636 0.219895

C -0.996001 -1.295207 0.268949

C -0.389200 -0.102525 -0.002429

C -0.994807 1.167792 -0.272173

C -2.402305 1.247059 -0.239106

C -3.165245 0.135457 0.007987

H -2.878522 2.210034 -0.408840

H -4.251348 0.214902 0.046222

H -0.395405 2.048889 -0.472098

N 1.079878 -0.062624 0.005882

N 1.789266 0.911206 0.562847

N 1.869424 -0.966919 -0.550658

N 3.087442 -0.555660 -0.321378

N 3.039048 0.598411 0.356192

H -2.917527 -1.682345 1.133583

H -2.849723 -1.914010 -0.567798

RI-B2KPLYP/ma-def2-TZVP

Total electronic energy = -504.949697 Ha

Zero-point correction = 0.110772 Ha

Thermal correction to enthalpy= 0.120149 Ha

Thermal correction to free energy= 0.076529 Ha

C 2.495927 -1.226364 -0.122326

C 0.986979 -1.303793 -0.156598

C 0.396115 -0.093262 -0.013393

C 1.004122 1.187849 0.132516

C 2.405964 1.244462 0.151255

C 3.149665 0.115119 0.035925

H 2.890485 2.205891 0.265479

H 4.232088 0.177451 0.054333

H 0.411054 2.085533 0.220575

N -1.060807 -0.040763 -0.025662

N -1.753191 0.824253 -0.737078

N -1.849448 -0.821773 0.675950

N -3.061987 -0.443607 0.383484

N -3.003756 0.567674 -0.478887

H 2.876813 -1.716866 -1.030532

H 2.846201 -1.904042 0.671226


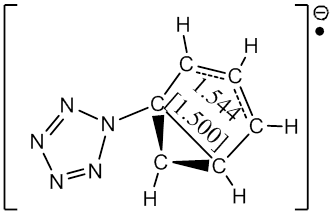


B3LYP/6-311++G**

Total electronic energy = -505.339728 Ha

Zero-point correction = 0.106670 Ha

Thermal correction to enthalpy = 0.116053 Ha

Thermal correction to free energy = 0.071400 Ha

C 2.596461 0.225250 0.830015

C 1.492229 1.059132 0.358849

C 1.151101 1.257767 -1.164107

C 0.574590 0.107317 -0.439149

C 1.349001 -1.167216 -0.523038

C 2.508710 -1.036641 0.232816

H 1.100382 -1.978869 -1.192876

H 3.270170 -1.807182 0.306617

H 3.338082 0.537602 1.555308

H 1.043710 1.787952 1.031170

N -0.845410 -0.007323 -0.085207

H 0.427643 2.067642 -1.293140

N -1.514719 -1.152990 -0.066916

N -2.755823 -0.825936 0.204126

N -2.834871 0.496961 0.342064

N -1.650968 1.020601 0.151734

RI-B2KPLYP/ma-def2-TZVP

Total electronic energy = -504.908791 Ha

Zero-point correction = 0.109600 Ha

Thermal correction to enthalpy= 0.118712 Ha

Thermal correction to free energy= 0.075986 Ha

C 2.638272 0.192811 0.727153

C 1.458756 1.000576 0.427950

C 1.127558 1.305314 -1.084033

C 0.565904 0.093647 -0.366006

C 1.309154 -1.171329 -0.532480

C 2.528432 -1.042739 0.105610

H 0.990109 -1.990057 -1.154639

H 3.308334 -1.789695 0.078657

H 3.488752 0.531148 1.298695

H 1.028043 1.650209 1.178558

N -0.832099 -0.018131 -0.027532

H 0.341982 2.069265 -1.070220

N -1.571906 -1.055499 -0.354783

N -2.781095 -0.758938 0.050121

N -2.762791 0.441626 0.606720

N -1.546880 0.916864 0.561512


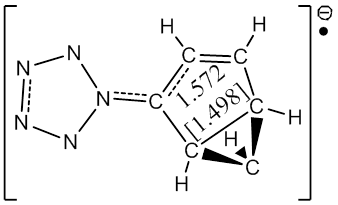


B3LYP/6-311++G**

Total electronic energy = - 505.351010 Ha

Zero-point correction = 0.107459 Ha

Thermal correction to enthalpy = 0.116747 Ha

Thermal correction to free energy = 0.073015 Ha

C 2.482268 1.145503 -0.132650

C 2.713337 -0.326856 -0.297940

C 2.278114 -1.275818 0.757667

C 1.291443 -0.996631 -0.320399

C 0.400920 0.150446 -0.040899

C 1.148148 1.366843 0.057071

H 0.993853 -1.760581 -1.037432

H 0.687489 2.326243 0.256910

H 3.258708 1.897876 -0.163204

H 3.462499 -0.679491 -1.007330

H 2.174907 -1.046783 1.814500

N -0.960637 0.054162 -0.011875

N -1.811403 1.094246 0.137142

N -3.018232 0.546934 0.091266

N -2.920191 -0.750637 -0.076414

N -1.641370 -1.104446 -0.140197

RI-B2KPLYP/ma-def2-TZVP

Total electronic energy = -504.911159 Ha

Zero-point correction = 0.110405 Ha

Thermal correction to enthalpy= 0.119399 Ha

Thermal correction to free energy= 0.077251 Ha

C 2.455497 1.089718 -0.155078

C 2.677667 -0.333599 -0.402727

C 2.353656 -1.335884 0.747259

C 1.320627 -0.967169 -0.391052

C 0.429975 0.140719 -0.126663

C 1.132991 1.354358 0.047361

H 1.014590 -1.771019 -1.043775

H 0.696118 2.280750 0.381050

H 3.247135 1.818390 -0.057666

H 3.446797 -0.648747 -1.096645

H 2.090976 -0.726653 1.619899

N -0.938795 0.024439 -0.009403

N -1.760554 1.030190 0.277651

N -2.952015 0.503729 0.272892

N -2.862532 -0.787397 -0.007976

N -1.609856 -1.106347 -0.186594


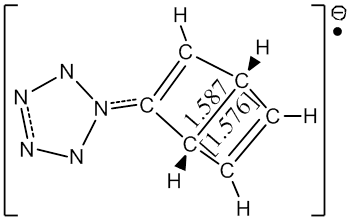


B3LYP/6-311++G**

Total electronic energy = -505.349735 Ha

Zero-point correction = 0.108166 Ha

Thermal correction to enthalpy = 0.117357 Ha

Thermal correction to free energy = 0.073747 Ha

C 0.311418 0.377763 0.258282

C 1.154016 1.457536 0.097449

C 2.389988 0.633047 0.444568

C 2.993821 -0.226883 -0.674374

C 2.155942 -1.259390 -0.483589

C 1.403612 -0.590590 0.663965

H 1.035659 2.431241 -0.356878

H 3.743151 -0.010165 -1.431294

H 1.965042 -2.181058 -1.022887

H 3.059496 0.969840 1.242870

N -1.001940 0.126950 0.056094

H 1.314935 -1.109436 1.620845

N -1.924642 1.060775 -0.351244

N -3.076684 0.393601 -0.335148

N -2.892645 -0.849086 0.048917

N -1.614241 -1.082142 0.311316

RI-B2KPLYP/ma-def2-TZVP

Total electronic energy = -504.911838 Ha

Zero-point correction = 0.110874 Ha

Thermal correction to enthalpy= 0.119850 Ha

Thermal correction to free energy= 0.077733 Ha

C 0.316776 0.383318 0.256968

C 1.155843 1.458384 0.087412

C 2.380127 0.631926 0.431751

C 2.966445 -0.234551 -0.678186

C 2.128057 -1.257298 -0.469785

C 1.397191 -0.576222 0.669238

H 1.037482 2.422193 -0.377536

H 3.709309 -0.034164 -1.439133

H 1.934497 -2.183562 -0.990801

H 3.055698 0.965386 1.218885

N -0.985603 0.129840 0.061196

H 1.311667 -1.079571 1.628979

N -1.891831 1.043204 -0.358966

N -3.038256 0.375358 -0.348089

N -2.850473 -0.849712 0.046368

N -1.578886 -1.066555 0.319995


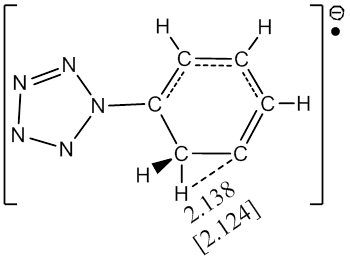


B3LYP/6-311++G**

Total electronic energy = -505.388733 Ha

Zero-point correction = 0.108683 Ha

Thermal correction to enthalpy = 0.118079 Ha

Thermal correction to free energy = 0.073969 Ha

C -2.480786 1.183759 0.069951

C -1.100484 1.194153 0.221715

C -0.378866 0.003865 0.157064

C -1.082322 -1.327243 0.186520

C -2.556319 -1.250442 -0.156470

C -3.152362 -0.033861 -0.299291

H -3.042849 2.102733 0.219642

H -0.567804 2.129961 0.349525

H -0.979801 -1.809155 1.174921

H -4.185266 0.036513 -0.643848

N 1.002916 0.025666 0.023863

N 1.773978 -1.076547 0.064529

N 3.009767 -0.631884 -0.058886

N 3.000976 0.686941 -0.153600

N 1.763308 1.131565 -0.117148

H -0.574049 -2.031631 -0.488476

RI-B2KPLYP/ma-def2-TZVP

Total electronic energy = -504.946602 Ha

Zero-point correction = 0.111314 Ha

Thermal correction to enthalpy= 0.120594 Ha

Thermal correction to free energy= 0.077457 Ha

C -2.614004 1.248592 -0.025387

C -1.105149 1.251162 0.044172

C -0.395454 -0.063508 0.011268

C -1.073750 -1.250769 -0.059258

C -2.461092 -1.223956 -0.111588

C -3.183560 -0.006799 -0.097450

H -0.723132 1.899583 -0.756470

H -4.266201 -0.092414 -0.149542

N 0.999114 -0.060807 0.062220

N 1.777487 -1.137922 0.045747

N 2.996412 -0.675430 0.111188

N 2.966042 0.643535 0.165946

N 1.720753 1.048111 0.136513

H -2.989707 -2.168785 -0.168380

H -0.808850 1.807831 0.945097

H -0.533168 -2.184858 -0.076635


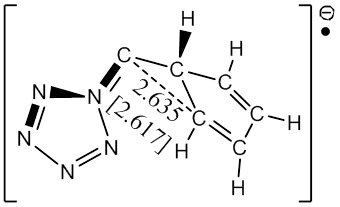


B3LYP/6-311++G**

Total electronic energy = - 505.365344 Ha

Zero-point correction = 0.108025 Ha

Thermal correction to enthalpy = 0.117532 Ha

Thermal correction to free energy = 0.072553 Ha

C -1.736400 0.246664 1.174685

C -1.232097 1.056621 -0.000153

C 0.219439 1.564511 -0.000152

C -1.736274 0.246254 -1.174762

C -2.474163 -0.792653 -0.731526

C -2.474244 -0.792395 0.731733

H -1.530841 0.508422 -2.204333

H -2.971688 -1.533983 -1.347463

H -2.971836 -1.533509 1.347876

N 1.124449 0.537075 -0.000046

N 0.908263 -0.819631 0.000073

N 2.092236 -1.368486 -0.000047

N 3.033548 -0.413058 0.000281

N 2.476761 0.763487 -0.000098

H -1.531076 0.509194 2.204185

H -1.838919 1.980155 -0.000345

RI-B2KPLYP/ma-def2-TZVP

Total electronic energy = -504.930324 Ha

Zero-point correction = 0.110863 Ha

Thermal correction to enthalpy= 0.120121 Ha

Thermal correction to free energy= 0.076887 Ha

C -1.718568 -0.249714 -1.168978

C -1.233426 -1.063288 -0.001943

C 0.195994 -1.598438 -0.011976

C -1.710652 -0.245486 1.164780

C -2.422372 0.808474 0.726326

C -2.426054 0.806638 -0.729494

H -1.512013 -0.511199 2.190642

H -2.898296 1.556492 1.342947

H -2.902762 1.555315 -1.344731

N 1.104468 -0.540422 -0.000237

N 0.858440 0.777029 0.002453

N 2.016538 1.361139 0.007508

N 2.972344 0.428474 0.010969

N 2.427144 -0.744118 0.002772

H -1.521199 -0.517914 -2.194337

H -1.856404 -1.969798 0.003200


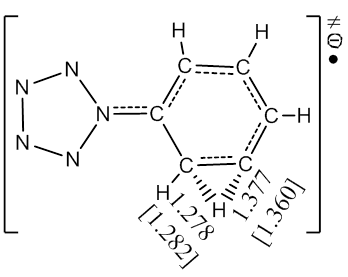


B3LYP/6-311++G**

Imaginary frequency = 1307.0i cm^-1^

Total electronic energy = -505.358583 Ha

Zero-point correction = 0.105303 Ha

Thermal correction to enthalpy = 0.114318 Ha

Thermal correction to free energy = 0.071040 Ha

C 2.474626 1.180708 -0.000869

C 1.085366 1.227487 -0.019854

C 0.358009 0.019920 -0.033163

C 1.102409 -1.231418 -0.051732

C 2.545671 -1.294982 -0.088630

C 3.187308 -0.032691 0.031109

H 3.016974 2.125206 0.001592

H 0.554504 2.170420 -0.030820

H 0.534540 -2.134721 -0.258416

H 4.272325 0.005689 0.102520

N -1.016816 0.012869 -0.008196

N -1.787086 -1.109437 -0.006417

N -3.028326 -0.654425 0.008779

N -3.034335 0.662782 0.020562

N -1.796355 1.128958 0.013594

H 1.741747 -1.665967 0.965712

RI-B2KPLYP/ma-def2-TZVP

Imaginary frequency = 1335.3i cm^-1^

Total electronic energy = -504.917827 Ha

Zero-point correction = 0.107852 Ha

Thermal correction to enthalpy = 0.116689 Ha

Thermal correction to free energy = 0.074771 Ha

C 2.455403 1.176794 0.007783

C 1.078741 1.222947 -0.009043

C 0.364329 0.012966 -0.048614

C 1.095097 -1.228870 -0.048132

C 2.533021 -1.289354 -0.088400

C 3.169799 -0.034532 0.011437

H 2.994970 2.117303 0.029014

H 0.546184 2.160638 -0.006674

H 0.532676 -2.135786 -0.229377

H 4.250102 0.008273 0.071253

N -1.016799 0.011534 -0.022258

N -1.769059 -1.087234 -0.013207

N -3.004302 -0.645880 -0.001233

N -3.005604 0.666231 0.002573

N -1.769742 1.109409 -0.007582

H 1.754970 -1.652175 0.965925


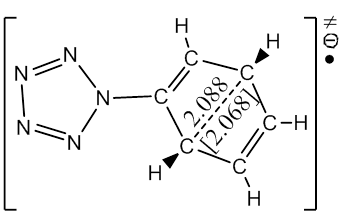


B3LYP/6-311++G**

Imaginary frequency = 635.2i cm^-1^

Total electronic energy = -505.320394 Ha

Zero-point correction = 0.104968 Ha

Thermal correction to enthalpy = 0.114352 Ha

Thermal correction to free energy = 0.070181 Ha

C -0.355431 0.221368 -0.248634

C -1.088267 1.366048 -0.123279

C -2.444413 0.846039 -0.444333

C -3.048463 -0.088063 0.573546

C -2.319357 -1.209415 0.434603

C -1.281431 -0.882602 -0.586057

H -0.794878 2.335592 0.266554

H -3.754642 0.172286 1.363452

H -2.373002 -2.127515 1.023935

H -3.054806 1.286494 -1.234495

N 1.022730 0.061633 -0.030332

H -0.952141 -1.639384 -1.298926

N 1.862555 1.063721 0.279343

N 3.056661 0.514927 0.287993

N 2.956129 -0.775153 -0.009691

N 1.695303 -1.086232 -0.206683

RI-B2KPLYP/ma-def2-TZVP

Imaginary frequency = 832.2i cm^-1^

Total electronic energy = -504.885114 Ha

Zero-point correction = 0.108745 Ha

Thermal correction to enthalpy = 0.11772 Ha

Thermal correction to free energy = 0.075186 Ha

C -0.372458 -0.214914 0.294304

C -1.086245 -1.350084 0.142251

C -2.439999 -0.838462 0.435436

C -3.010314 0.097418 -0.585689

C -2.285056 1.213555 -0.422690

C -1.297933 0.874942 0.631392

H -0.766936 -2.315380 -0.231186

H -3.697846 -0.150531 -1.390493

H -2.318583 2.127298 -1.012939

H -3.081965 -1.335583 1.157799

N 1.016887 -0.061394 0.036945

H -0.963254 1.634715 1.334362

N 1.833764 -1.049753 -0.287573

N 3.020678 -0.510837 -0.341833

N 2.925146 0.779298 -0.050665

N 1.674725 1.072275 0.185918


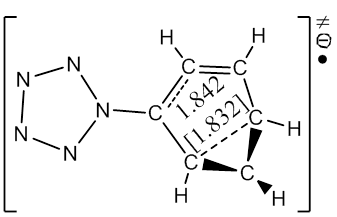


B3LYP/6-311++G**

Imaginary frequency = 477.8i cm^-1^

Total electronic energy = -505.345545 Ha

Zero-point correction = 0.105553 Ha

Thermal correction to enthalpy = 0.114956 Ha

Thermal correction to free energy = 0.070770 Ha

C 2.409755 1.172508 -0.192660

C 2.815722 -0.201555 -0.469140

C 2.490015 -1.206405 0.539608

C 1.219725 -1.063855 -0.151304

C 0.387510 0.072699 0.203536

C 1.106872 1.297180 0.250148

H 0.823136 -1.863555 -0.774019

H 0.642548 2.237252 0.519628

H 3.049619 2.020552 -0.410694

H 3.520242 -0.429705 -1.271293

H 2.712279 -1.226869 1.608027

N -0.990939 0.018935 0.068901

N -1.808478 1.085314 0.063147

N -3.024710 0.580394 -0.054379

N -2.956615 -0.731615 -0.138211

N -1.694318 -1.118899 -0.046998

RI-B2KPLYP/ma-def2-TZVP

Imaginary frequency = 455.1i cm^-1^

Total electronic energy = -504.916011 Ha

Zero-point correction = 0.108485 Ha

Thermal correction to enthalpy = 0.117479 Ha

Thermal correction to free energy = 0.075299 Ha

C -2.403751 1.154388 0.171680

C -2.800599 -0.206120 0.471623

C -2.486686 -1.212512 -0.531031

C -1.217735 -1.069045 0.148565

C -0.400145 0.051319 -0.243648

C -1.113854 1.269921 -0.299401

H -0.821642 -1.852346 0.784257

H -0.655842 2.202027 -0.591532

H -3.030468 2.005147 0.395438

H -3.482782 -0.432594 1.285870

H -2.687216 -1.183658 -1.597294

N 0.971310 0.011641 -0.079060

N 1.769703 1.077105 -0.062614

N 2.983433 0.590254 0.088136

N 2.924602 -0.717762 0.181711

N 1.672276 -1.110541 0.063174


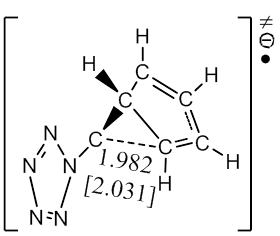


B3LYP/6-311++G**

Imaginary frequency = 386.2i cm^-1^

Total electronic energy = -505.348368 Ha

Zero-point correction = 0.107162 Ha

Thermal correction to enthalpy = 0.116027 Ha

Thermal correction to free energy = 0.072664 Ha

C -1.561401 1.150761 -0.569787

C -1.483179 -0.263436 -1.060143

C -0.209594 -1.103530 -0.910046

C -1.884895 -1.069678 0.147921

C -2.043315 -0.200723 1.224704

C -1.851095 1.149129 0.764504

H -2.119524 -2.120930 0.127170

H -2.241242 -0.491677 2.248765

H -1.881336 2.027293 1.400839

N 0.867707 -0.421133 -0.312409

N 1.295698 0.833478 -0.586701

N 2.448375 0.954641 0.014313

N 2.727278 -0.174707 0.670553

N 1.766767 -1.039802 0.473612

H -1.308618 2.014732 -1.167478

H -1.989183 -0.471887 -2.007796

RI-B2KPLYP/ma-def2-TZVP

Imaginary frequency = 437.0i cm^-1^

Total electronic energy = -504.914002 Ha

Zero-point correction = 0.110619 Ha

Thermal correction to enthalpy = 0.119292 Ha

Thermal correction to free energy = 0.077238 Ha

C -1.538263 1.128730 -0.578627

C -1.545096 -0.262208 -1.083966

C -0.225539 -1.079037 -1.061834

C -1.876815 -1.067787 0.121364

C -1.903394 -0.241376 1.211833

C -1.706183 1.114486 0.757449

H -2.065308 -2.124807 0.102830

H -1.988757 -0.549952 2.240981

H -1.653643 1.978085 1.404181

N 0.798331 -0.385384 -0.351440

N 1.353792 0.759528 -0.736881

N 2.380063 0.936826 0.046830

N 2.444994 -0.068752 0.910023

N 1.466917 -0.900767 0.667920

H -1.327523 1.993637 -1.184608

H -2.081131 -0.458692 -2.008033


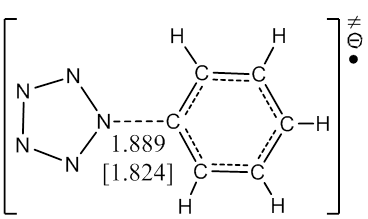


B3LYP/6-311++G**

Imaginary frequency = 326.4i cm^-1^

Total electronic energy = -505.436236 Ha

Total electronic energy solvent (THF) = -505.505552 Ha

Total electronic energy CCSD(T)/cc-pVTZ = -504.438251 Ha

Total electronic energy HF/cc-pVTZ = -502.367510 Ha

Total electronic energy CCSD(T)/cc-pVDZ = -503.956425 Ha

Total electronic energy HF/cc-pVDZ = -502.242460 Ha

Total electronic energy CCSD(T)/CBS = -504.697095 Ha

Zero-point correction = 0.108403 Ha

Thermal correction to enthalpy = 0.117632 Ha

Thermal correction to free energy = 0.072635 Ha

C -2.594398 1.207884 0.016834

C -1.211287 1.213177 -0.166171

C -0.531448 0.000004 -0.281462

C -1.211281 -1.213174 -0.166178

C -2.594393 -1.207888 0.016827

C -3.296786 -0.000004 0.108040

H -3.133355 2.151302 0.077831

H -0.663148 2.149160 -0.229858

H -0.663139 -2.149154 -0.229871

H -3.133345 -2.151308 0.077819

H -4.372236 -0.000006 0.253513

N 1.230448 0.000005 0.402619

N 1.971613 -1.087843 0.180778

N 3.170500 -0.664120 -0.176130

N 3.170503 0.664113 -0.176140

N 1.971618 1.087847 0.180762

RI-B2KPLYP/ma-def2-TZVP

Imaginary frequency = 369.4i cm^-1^

Total electronic energy = -504.999941 Ha

Zero-point correction = 0.110976 Ha

Thermal correction to enthalpy = 0.119967 Ha

Thermal correction to free energy = 0.076853 Ha

C 2.567693 -1.199726 0.022006

C 1.204086 -1.207772 -0.226542

C 0.522695 0.000065 -0.367042

C 1.204092 1.207864 -0.226228

C 2.567690 1.199754 0.022336

C 3.264097 -0.000003 0.147667

H 3.099759 -2.139802 0.108084

H 0.668120 -2.143133 -0.322345

H 0.668120 2.143248 -0.321784

H 3.099749 2.139808 0.108693

H 4.325588 -0.000033 0.348200

N -1.168069 0.000032 0.318382

N -1.915553 1.080169 0.175376

N -3.140666 0.661162 -0.050551

N -3.140580 -0.661440 -0.050399

N -1.915410 -1.080232 0.175632


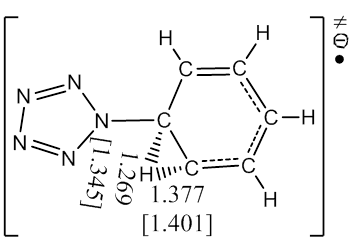


B3LYP/6-311++G**

Imaginary frequency = 1519.9i cm^-1^

Total electronic energy = -505.334905 Ha

Zero-point correction = 0.103917 Ha

Thermal correction to enthalpy = 0.113070 Ha

Thermal correction to free energy = 0.069272 Ha

C 1.092587 -1.339953 0.157022

C 0.388200 -0.065976 0.196355

C 1.056654 1.232947 0.028071

C 2.422022 1.210644 -0.169008

C 3.155334 0.011071 -0.186177

C 2.483286 -1.213685 0.071671

H 4.228627 0.035677 -0.353911

H 3.089404 -2.113572 0.179296

H 2.943780 2.155578 -0.306129

H 0.496236 -0.758242 1.253820

H 0.496521 2.155386 0.098053

N -1.037885 -0.054335 0.028282

N -1.774303 1.059432 0.201708

N -3.017707 0.668330 0.043431

N -3.042683 -0.633908 -0.223429

N -1.819290 -1.108821 -0.258383

RI-B2KPLYP/ma-def2-TZVP

Imaginary frequency = 1460.3i cm^-1^

Total electronic energy = -504.898972 Ha

Zero-point correction = 0.105622 Ha

Thermal correction to enthalpy = 0.114573 Ha

Thermal correction to free energy = 0.072056 Ha

C 1.063772 -1.320312 0.200147

C 0.420892 -0.052059 0.151200

C 1.020681 1.194631 -0.196471

C 2.429638 1.189593 -0.236435

C 3.135126 0.017106 -0.097933

C 2.460948 -1.225875 0.093319

H 4.217258 0.043558 -0.147774

H 3.059206 -2.118403 0.228653

H 2.958229 2.118394 -0.408204

H 0.528139 -0.689366 1.330798

H 0.442857 2.104556 -0.232995

N -1.034652 -0.049339 0.090034

N -1.792764 0.753116 0.806347

N -3.011073 0.499454 0.434412

N -2.984522 -0.446938 -0.505915

N -1.748834 -0.787483 -0.727986


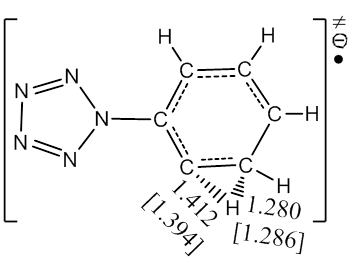


B3LYP/6-311++G**

Imaginary frequency = 1385.0i cm^-1^

Total electronic energy = -505.354023 Ha

Zero-point correction = 0.104552 Ha

Thermal correction to enthalpy = 0.113614 Ha

Thermal correction to free energy = 0.069994 Ha

C 2.468135 -1.187851 -0.032379

C 1.035371 -1.311879 -0.152267

C 0.362229 -0.069869 -0.044342

C 1.026586 1.198473 0.012764

C 2.416339 1.237338 0.036936

C 3.183898 0.072021 0.002028

H 2.907853 2.206429 0.078160

H 4.267663 0.100019 0.047424

H 0.452279 2.115530 0.035377

N -1.048576 -0.055357 -0.018106

N -1.770813 1.081180 -0.045473

N -1.857005 -1.129067 0.044932

N -3.074489 -0.634117 0.044891

N -3.028241 0.688851 -0.006909

H 1.818294 -1.620172 0.981708

H 3.052422 -2.091617 -0.214457

RI-B2KPLYP/ma-def2-TZVP

Imaginary frequency = 1427.1i cm^-1^

Total electronic energy = -504.916672 Ha

Zero-point correction = 0.107476 Ha

Thermal correction to enthalpy = 0.116267 Ha

Thermal correction to free energy = 0.074416 Ha

C 2.457877 -1.185091 -0.040767

C 1.030627 -1.310657 -0.148519

C 0.374084 -0.073131 -0.052724

C 1.026658 1.190457 0.042186

C 2.405025 1.227759 0.047615

C 3.168593 0.065503 -0.027749

H 2.896014 2.191155 0.105698

H 4.247580 0.094223 0.008372

H 0.453009 2.101784 0.099186

N -1.041447 -0.052547 -0.029312

N -1.748001 1.063591 -0.138183

N -1.833722 -1.104281 0.110387

N -3.047671 -0.623347 0.073768

N -3.000103 0.688905 -0.072176

H 1.796435 -1.603856 0.978979

H 3.035371 -2.090544 -0.201027


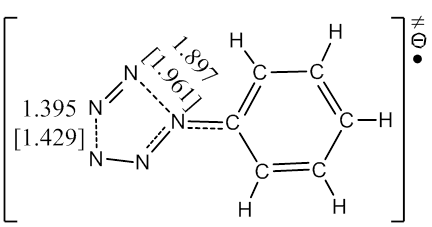


B3LYP/6-311++G**

Imaginary frequency = 442.6i cm^-1^

Total electronic energy = -505.446107 H

Total electronic energy solvent (THF) = -505.515183 Ha

Total electronic energy CCSD(T)/cc-pVTZ = -504.435740 Ha

Total electronic energy HF/cc-pVTZ = -502.364864 Ha

Total electronic energy CCSD(T)/cc-pVDZ = -503.952540 Ha

Total electronic energy HF/cc-pVDZ = -502.23903 Ha

Total electronic energy CCSD(T)/CBS = -504.695207 Ha

Zero-point correction = 0.107604 Ha

Thermal correction to enthalpy = 0.116975 Ha

Thermal correction to free energy = 0.072475 Ha

C -2.599421 -1.141178 0.044938

C -1.213094 -1.217534 0.066618

C -0.427571 -0.033047 0.027622

C -1.105773 1.209542 -0.052693

C -2.490719 1.261169 -0.074031

C -3.262396 0.089933 -0.021694

H -3.177440 -2.061529 0.077857

H -0.705701 -2.172704 0.115216

H -0.510335 2.114122 -0.096386

H -2.982536 2.228228 -0.136648

H -4.345916 0.137356 -0.036886

N 0.935575 -0.032954 0.128869

N 2.386364 1.324007 0.149598

N 3.237340 0.487764 -0.048453

N 2.967284 -0.880680 -0.076795

N 1.661404 -1.077962 -0.134320

RI-B2KPLYP/ma-def2-TZVP

Imaginary frequency = 502.9i cm^-1^

Total electronic energy = -504.998704 Ha

Zero-point correction = 0.109503 Ha

Thermal correction to enthalpy = 0.118737 Ha

Thermal correction to free energy = 0.075765 Ha

C -2.579568 -1.139103 0.016057

C -1.198183 -1.201790 0.095941

C -0.431387 -0.023398 0.100189

C -1.105084 1.204184 0.004981

C -2.485015 1.250286 -0.075438

C -3.240966 0.081048 -0.068118

H -3.150259 -2.058765 0.018014

H -0.685719 -2.148739 0.159792

H -0.511992 2.106867 -0.003884

H -2.978586 2.209995 -0.151574

H -4.318803 0.120630 -0.129749

N 0.932505 -0.002851 0.272054

N 2.378872 1.310859 0.108340

N 3.194053 0.491154 -0.232429

N 2.898242 -0.903306 -0.130463

N 1.619407 -1.070197 -0.058589


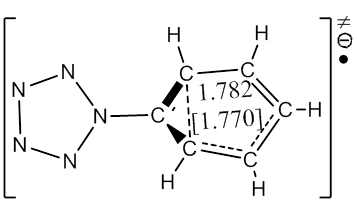


B3LYP/6-311++G**

Imaginary frequency = 691.1i cm^-1^

Total electronic energy = -505.354056 Ha

Zero-point correction = 0.106889 Ha

Thermal correction to enthalpy = 0.115838 Ha

Thermal correction to free energy = 0.072592 Ha

C 2.469661 1.153928 -0.142464

C 1.119541 0.882419 0.422589

C 0.290858 0.030645 -0.380035

C 1.179861 -0.898173 0.412951

C 2.544605 -1.154692 0.110448

C 3.218500 0.015131 -0.326748

H 0.650297 -1.509516 1.141460

H 3.039832 -2.077222 0.390970

H 4.222064 0.017069 -0.741231

N -1.076023 0.000478 -0.160339

N -1.832516 1.088478 0.081122

N -3.073067 0.642772 0.102323

N -3.079187 -0.666731 -0.084815

N -1.846175 -1.099012 -0.254170

H 2.781692 2.157835 -0.409660

H 0.716725 1.474392 1.249178

RI-B2KPLYP/ma-def2-TZVP

Imaginary frequency = 940.9i cm^-1^

Total electronic energy = -504.918451 Ha

Zero-point correction = 0.110291 Ha

Thermal correction to enthalpy = 0.118926 Ha

Thermal correction to free energy = 0.077451 Ha

C -2.461316 -1.146611 -0.129613

C -1.108065 -0.875803 0.392401

C -0.311609 -0.046357 -0.448888

C -1.172169 0.893054 0.367442

C -2.527907 1.145114 0.120458

C -3.206834 -0.022226 -0.300216

H -0.599219 1.516012 1.044399

H -3.013333 2.061075 0.419731

H -4.214725 -0.027692 -0.691010

N 1.056403 -0.006825 -0.173491

N 1.803777 -1.084272 0.045007

N 3.031843 -0.638492 0.137192

N 3.029773 0.676432 0.004759

N 1.803330 1.090620 -0.192574

H -2.779141 -2.149224 -0.374846

H -0.675853 -1.453578 1.209049


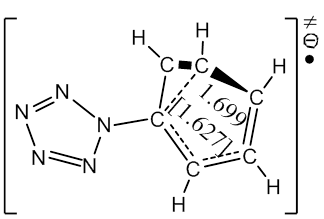


B3LYP/6-311++G**

Imaginary frequency = 593.8i cm^-1^

Total electronic energy = -505.331024 Ha

Zero-point correction = 0.105966 Ha

Thermal correction to enthalpy = 0.114921 Ha

Thermal correction to free energy = 0.071791 Ha

C 2.796375 0.165952 0.552967

C 1.585408 1.042411 0.563100

C 1.089550 1.439583 -0.740130

C 0.541849 0.065928 -0.355023

C 1.240241 -1.190064 -0.501890

C 2.568704 -1.045291 -0.076830

H 0.746689 -2.115822 -0.759098

H 3.329472 -1.807636 -0.212006

H 3.746020 0.500885 0.955430

H 1.292515 1.536992 1.499392

N -0.866747 -0.014524 -0.085088

H 0.351556 2.237590 -0.719453

N -1.512208 -1.142897 0.234674

N -2.771312 -0.804915 0.327058

N -2.899501 0.498787 0.084834

N -1.721519 1.003102 -0.192539

RI-B2KPLYP/ma-def2-TZVP

Imaginary frequency = 1242.8i cm^-1^

Total electronic energy = -504.897069 Ha

Zero-point correction = 0.108326 Ha

Thermal correction to enthalpy = 0.117317 Ha

Thermal correction to free energy = 0.075021 Ha

C 2.781260 0.236610 0.485056

C 1.509880 1.016189 0.557308

C 1.058588 1.337047 -0.763572

C 0.571861 -0.023530 -0.270470

C 1.249657 -1.278878 -0.329169

C 2.576469 -1.032557 0.002484

H 0.772668 -2.220258 -0.528514

H 3.372015 -1.755174 -0.121579

H 3.732948 0.681306 0.728936

H 1.196400 1.534092 1.466560

N -0.853771 -0.037578 -0.070056

H 0.320819 2.121518 -0.887596

N -1.635274 -1.013567 -0.483189

N -2.843921 -0.651699 -0.151647

N -2.790674 0.531700 0.448479

N -1.549841 0.924808 0.502952


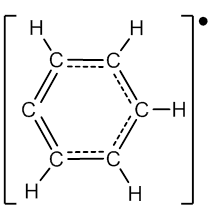


B3LYP/6-311++G**

Total electronic energy = -231.622376 Ha

Zero-point correction = 0.086918 Ha

Thermal correction to enthalpy = 0.092255 Ha

Thermal correction to free energy = 0.058869 Ha

C -1.212659 -0.631598 -0.000024

C -1.224736 0.771198 -0.000024

C 0.000000 1.395207 0.000000

C 1.224736 0.771198 0.000024

C 1.212659 -0.631598 0.000024

C 0.000000 -1.322209 0.000000

H -2.151117 -1.176276 -0.000043

H -2.158348 1.322783 -0.000043

H 2.158348 1.322783 0.000043

H 2.151117 -1.176276 0.000043

H 0.000000 -2.406208 0.000000

RI-B2KPLYP/ma-def2-TZVP

Total electronic energy = -231.391118 Ha

Zero-point correction = 0.089269 Ha

Thermal correction to enthalpy = 0.094488 Ha

Thermal correction to free energy = 0.062333 Ha

C -1.202475 -0.627264 -0.000024

C -1.215680 0.764289 -0.000024

C -0.000000 1.383451 0.000000

C 1.215680 0.764289 0.000024

C 1.202475 -0.627264 0.000024

C 0.000000 -1.313063 -0.000000

H -2.136653 -1.170122 -0.000042

H -2.144647 1.313646 -0.000043

H 2.144647 1.313645 0.000043

H 2.136653 -1.170121 0.000042

H -0.000000 -2.392482 -0.000000


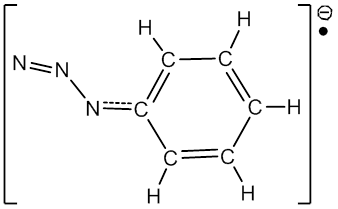


B3LYP/6-311++G**

Total electronic energy = -395.975391 Ha

Zero-point correction = 0.098989 Ha

Thermal correction to enthalpy = 0.107303 Ha

Thermal correction to free energy = 0.066371 Ha

C -1.608807 1.363697 0.000030

C -0.255490 1.048187 0.000229

C 0.188896 -0.306348 0.000252

C -0.832398 -1.298726 0.000015

C -2.178494 -0.965698 -0.000101

C -2.596056 0.372062 -0.000199

H -1.902362 2.411514 0.000038

H 0.487891 1.835574 0.000600

H -0.518930 -2.337756 0.000092

H -2.919756 -1.761687 -0.000234

H -3.649929 0.630318 -0.000182

N 1.483594 -0.753262 0.000285

N 3.581305 0.188652 0.000039

N 2.391841 0.270752 -0.000563

RI-B2KPLYP/ma-def2-TZVP

Total electronic energy = -395.614547 Ha

Zero-point correction = 0.100269 Ha

Thermal correction to enthalpy = 0.108748 Ha

Thermal correction to free energy = 0.068619 Ha

C -1.596956 1.354346 0.000059

C -0.250101 1.034934 0.000050

C 0.185562 -0.313027 0.000020

C -0.833350 -1.295133 -0.000065

C -2.172959 -0.959891 -0.000052

C -2.583146 0.372370 -0.000043

H -1.883942 2.399048 0.000078

H 0.490693 1.819137 0.000121

H -0.525026 -2.331440 -0.000085

H -2.913164 -1.750112 -0.000125

H -3.631262 0.634505 0.000006

N 1.471708 -0.763356 0.000066

N 3.544930 0.228308 0.000160

N 2.368318 0.267591 0.000113

The xyz coordinates of geometries in Fig. 5:

­­­___________________________________________________________


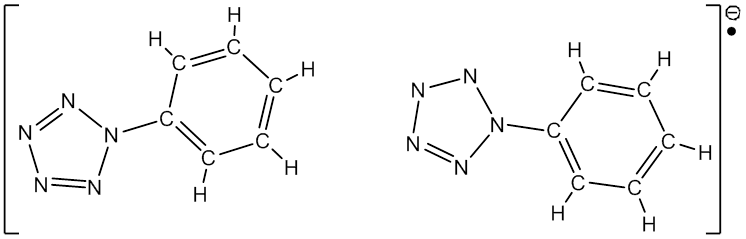


B3LYP/6-311++G**

Total electronic energy = -1010.951088 Ha

Zero-point correction = 0.226227 Ha

Thermal correction to enthalpy = 0.245354 Ha

Thermal correction to free energy = 0.170901 Ha

C 7.289901 0.892840 -0.000479

C 5.911009 1.040450 0.000063

C 5.088246 -0.108085 -0.000011

C 5.678070 -1.391849 -0.000575

C 7.059879 -1.508579 -0.001085

C 7.884945 -0.375817 -0.001065

H 7.913130 1.781645 -0.000445

H 5.451460 2.019438 0.000551

H 5.040954 -2.265778 -0.000580

H 7.502974 -2.499516 -0.001512

H 8.963925 -0.479139 -0.001488

N 3.708451 0.024564 0.000481

N 2.830189 -1.017880 0.000360

N 1.646569 -0.442735 0.000909

N 1.772842 0.874365 0.001264

N 3.045450 1.215207 0.001049

C -2.023441 -0.364612 0.000819

C -3.349347 -0.781962 0.000632

C -4.368542 0.181586 -0.000008

C -4.062914 1.548984 -0.000505

C -2.728971 1.940830 -0.000276

C -1.699851 0.995351 0.000438

H -1.221755 -1.093872 0.001198

H -3.606161 -1.832707 0.000918

H -4.862786 2.277124 -0.001078

H -2.489969 2.998893 -0.000631

H -0.659370 1.298398 0.000618

N -5.721031 -0.228995 -0.000201

N -6.770731 0.609617 -0.001548

N -7.821242 -0.169095 -0.001251

N -7.434570 -1.444604 0.000189

N -6.128257 -1.508916 0.000856


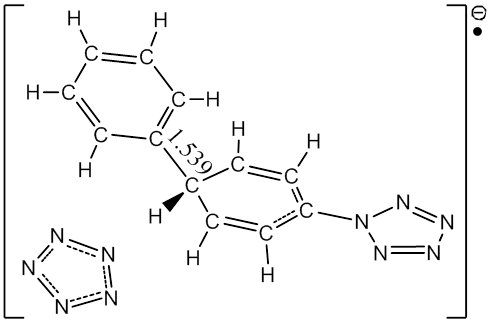


B3LYP/6-311++G**

Total electronic energy = -1010.953486 Ha

Zero-point correction = 0.226470 Ha

Thermal correction to enthalpy = 0.245695 Ha

Thermal correction to free energy = 0.171354 Ha

C -3.345851 2.754119 -0.007996

C -2.638695 1.549870 -0.005973

C -1.240621 1.561498 0.002320

C -0.567915 2.789771 0.008490

C -1.276411 3.988893 0.006567

C -2.671813 3.973512 -0.001712

H -4.430377 2.730064 -0.014295

H -3.179165 0.607280 -0.010733

H 0.517467 2.805395 0.014834

H -0.740988 4.932882 0.011523

H -3.227066 4.905851 -0.003200

N -2.759485 -2.029929 0.008201

N -2.806737 -3.352357 0.020457

N -4.081120 -3.708838 0.005945

N -4.820211 -2.607498 -0.015293

N -4.002759 -1.568890 -0.013996

C 0.376662 0.095150 -1.242458

C 1.714481 -0.136284 -1.237978

C 2.409419 -0.243947 -0.000155

C 1.718170 -0.133255 1.239477

C 0.380408 0.098389 1.247407

C -0.441315 0.246020 0.003557

H -0.152374 0.165871 -2.186272

H 2.266269 -0.246823 -2.162190

H 2.272684 -0.241793 2.162294

H -0.145765 0.171354 2.192653

H -1.196824 -0.572650 0.005895

N 3.792248 -0.457179 -0.001935

N 4.546547 -0.566736 1.100736

N 5.760707 -0.748048 0.664082

N 5.758656 -0.750199 -0.673044

N 4.543158 -0.570304 -1.106551


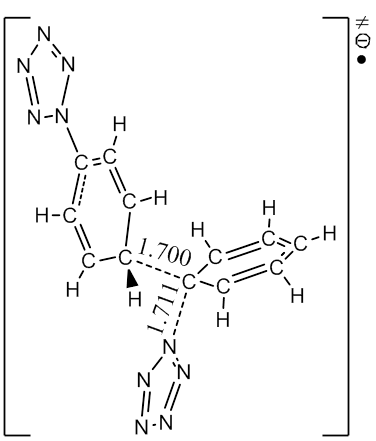


B3LYP/6-311++G**

Imaginary frequency = 333.8i cm^-1^

Total electronic energy = -1010.880719 Ha

Zero-point correction = 0.225101 Ha

Thermal correction to enthalpy = 0.242924 Ha

Thermal correction to free energy = 0.176863 Ha

C -2.710771 2.753756 -0.388987

C -2.459063 1.495545 -0.894107

C -1.680633 0.515103 -0.154028

C -1.208227 0.964999 1.140675

C -1.482288 2.231435 1.608651

C -2.238044 3.155382 0.869219

H -3.288793 3.448414 -0.992986

H -2.836320 1.213552 -1.871591

H -0.593673 0.290846 1.724611

H -1.081348 2.520906 2.576158

H -2.439850 4.148117 1.252574

N -2.690473 -0.847759 0.067460

N -3.119302 -1.277226 1.234578

N -3.970286 -2.238798 0.969118

N -4.058004 -2.379295 -0.357416

N -3.258796 -1.508360 -0.921511

C 0.473637 0.746803 -1.609495

C 1.778581 0.753065 -1.235421

C 2.286881 -0.271424 -0.387569

C 1.452837 -1.353060 -0.014654

C 0.144778 -1.379161 -0.402861

C -0.503977 -0.253649 -1.111172

H 0.098051 1.545553 -2.238962

H 2.449489 1.528667 -1.582150

H 1.875468 -2.173474 0.551695

H -0.462051 -2.237540 -0.140056

H -1.148077 -0.613464 -1.921245

N 3.627632 -0.247535 0.009696

N 4.205414 -1.167445 0.793909

N 5.450664 -0.778629 0.912505

N 5.633464 0.344850 0.226534

N 4.508948 0.696721 -0.346240

The xyz coordinates of geometries in Fig. 7:

­­­___________________________________________________________


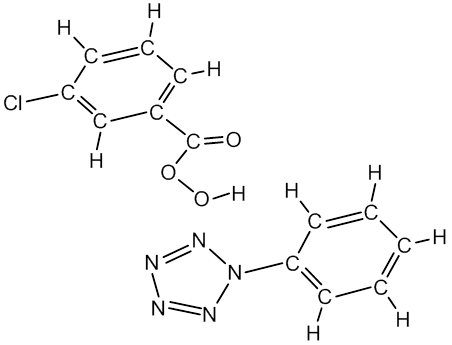


B3LYP/6-311++G**

Total electronic energy = -1461.166580 Ha

Zero-point correction = 0.223809 Ha

Thermal correction to enthalpy = 0.244450 Ha

Thermal correction to free energy = 0.165974 Ha

C 3.927049 1.584546 0.922642

C 2.904889 0.796567 0.400972

C 3.165573 -0.549570 0.149491

C 4.413130 -1.117793 0.404033

C 5.420882 -0.311228 0.923144

C 5.182249 1.037521 1.184131

H 3.735911 2.632461 1.120823

H 1.930611 1.218868 0.192917

H 4.584036 -2.165673 0.198290

H 6.394344 -0.742136 1.124675

H 5.971934 1.659443 1.589054

N 2.124592 -1.374626 -0.383076

N 2.293897 -2.631782 -0.781743

N 1.127596 -3.023560 -1.186812

N 0.249057 -2.013318 -1.040191

N 0.862523 -0.987622 -0.540699

C -3.649303 -0.198676 2.013739

C -3.808653 -0.374256 0.641006

C -2.974022 0.258261 -0.270453

C -1.953711 1.085429 0.213185

C -1.779331 1.270059 1.588309

C -2.629226 0.627820 2.481050

H -4.313842 -0.704085 2.702894

H -3.105811 0.102673 -1.331913

H -0.982723 1.913756 1.937918

H -2.501824 0.767424 3.547959

Cl -5.089004 -1.418972 0.051320

C -1.025623 1.791096 -0.699442

O -0.053548 2.444973 -0.374339

O -1.376217 1.655854 -1.998972

O -0.459787 2.338111 -2.867548

H 0.149768 2.746003 -2.211393


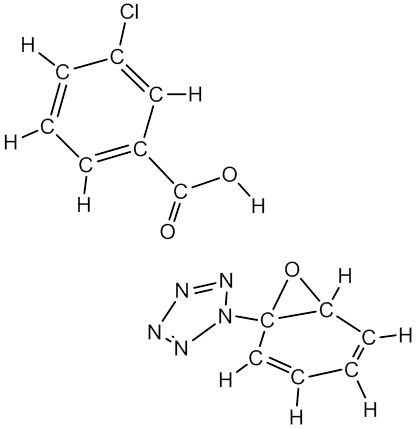


B3LYP/6-311++G**

Total electronic energy = -1461.193331 Ha

Zero-point correction = 0.224343 Ha

Thermal correction to enthalpy = 0.244353 Ha

Thermal correction to free energy = 0.170208 Ha

C -3.997635 -1.505398 1.799580

C -3.309388 -0.406561 1.434164

C -2.997530 -0.210938 0.010197

C -3.607423 -1.141916 -1.006576

C -4.459815 -2.220522 -0.497011

C -4.576994 -2.419747 0.832186

H -4.192342 -1.682910 2.850904

H -2.975171 0.338138 2.142668

H -3.753323 -0.770975 -2.015590

H -5.010986 -2.821424 -1.210581

H -5.189059 -3.233827 1.203650

N -2.694836 1.152866 -0.388360

N -2.263722 1.495513 -1.593543

N -2.116768 2.781302 -1.543819

N -2.462053 3.217478 -0.315780

N -2.823408 2.205129 0.406395

C 5.113667 0.618733 0.838815

C 4.706651 -0.368743 -0.054300

C 3.361668 -0.615960 -0.297182

C 2.401018 0.146584 0.375934

C 2.794831 1.141532 1.275764

C 4.146826 1.373151 1.502207

H 6.168513 0.792656 1.009534

H 3.059698 -1.384445 -0.994575

H 2.033029 1.720028 1.782329

H 4.457062 2.144764 2.196968

Cl 5.921539 -1.323665 -0.894091

C 0.942510 -0.075377 0.160329

O 0.071946 0.538646 0.741129

O 0.679744 -1.031470 -0.747356

O -2.196574 -1.174736 -0.635761

H -0.289319 -1.114264 -0.828751


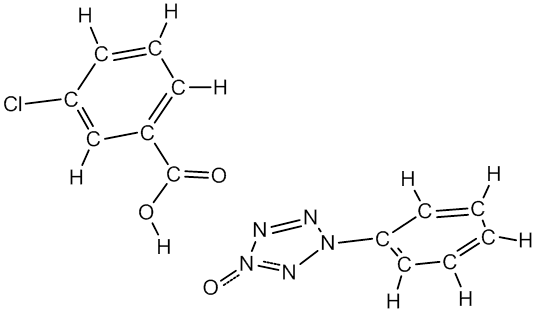


B3LYP/6-311++G**

Total electronic energy = -1461.211044 Ha

Zero-point correction = 0.225549 Ha

Thermal correction to enthalpy = 0.245334 Ha

Thermal correction to free energy = 0.171669 Ha

C 5.871842 -1.606922 0.628223

C 4.993005 -0.557370 0.873661

C 4.014616 -0.271876 -0.077114

C 3.897819 -1.001594 -1.258170

C 4.792459 -2.042604 -1.487412

C 5.776795 -2.348535 -0.549117

H 6.636665 -1.840185 1.359167

H 5.059127 0.029047 1.780099

H 3.122674 -0.759897 -1.972639

H 4.712453 -2.618362 -2.401585

H 6.467679 -3.162454 -0.734098

N 3.104309 0.802131 0.169204

N 2.324093 1.323438 -0.768560

N 1.661277 2.245609 -0.100877

N 2.051453 2.292031 1.216596

N 2.945136 1.377730 1.353194

O 0.782516 2.978969 -0.584803

C -4.994858 -1.864525 0.522542

C -5.079182 -0.599292 -0.052097

C -3.954360 0.199273 -0.214901

C -2.712102 -0.285405 0.207938

C -2.612391 -1.554131 0.786308

C -3.751471 -2.336287 0.941453

H -5.886785 -2.466964 0.639262

H -4.033363 1.180721 -0.660598

H -1.640549 -1.906715 1.107002

H -3.678954 -3.319935 1.390702

Cl -6.645127 0.001512 -0.582782

C -1.468539 0.529273 0.057186

O -0.367930 0.136194 0.392515

O -1.689825 1.731363 -0.483812

H -0.835553 2.220244 -0.549100


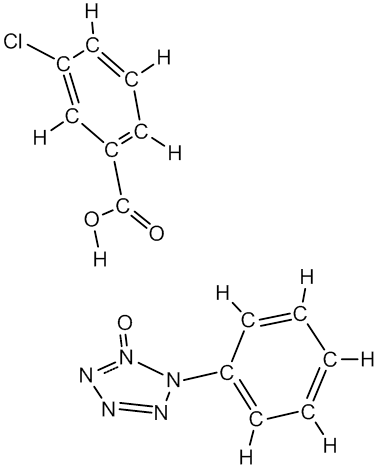


B3LYP/6-311++G**

Total electronic energy = -1461.211044 Ha

Zero-point correction = 0.225549 Ha

Thermal correction to enthalpy = 0.245334 Ha

Thermal correction to free energy = 0.171669 Ha

C 5.546871 -1.721165 0.435457

C 5.037266 -0.455612 0.163334

C 3.661039 -0.314381 0.000855

C 2.781674 -1.388697 0.102811

C 3.310537 -2.644526 0.387331

C 4.685145 -2.811507 0.552350

H 6.614803 -1.852473 0.561244

H 5.687359 0.405354 0.072103

H 1.720988 -1.235794 -0.053134

H 2.644951 -3.495268 0.470305

H 5.087012 -3.794312 0.769760

N 3.144952 0.985268 -0.302081

N 2.206952 1.655435 0.425623

N 2.030364 2.830430 -0.147161

N 2.861226 2.874262 -1.187369

N 3.540837 1.776706 -1.296496

C -5.218318 -0.899991 -1.074355

C -4.958838 -0.224039 0.114930

C -3.664119 0.111569 0.489412

C -2.602845 -0.241885 -0.351036

C -2.848576 -0.920378 -1.548462

C -4.152666 -1.245615 -1.904374

H -6.236784 -1.149113 -1.344118

H -3.476007 0.638336 1.414260

H -2.011474 -1.180196 -2.183548

H -4.348440 -1.770092 -2.832278

Cl -6.298924 0.214386 1.165387

C -1.192018 0.090512 0.002596

O -0.238344 -0.205741 -0.693583

O -1.075834 0.744777 1.162567

H -0.125145 0.940939 1.324141

O 1.654580 1.209416 1.445638


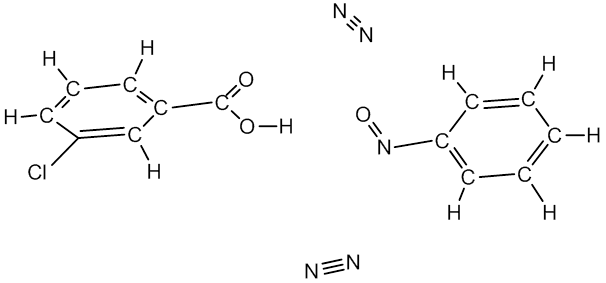


B3LYP/6-311++G**

Total electronic energy = -1461.340216 Ha

Zero-point correction = 0.215390 Ha

Thermal correction to enthalpy = 0.241670 Ha

Thermal correction to free energy = 0.141758 Ha

C 5.846508 0.343109 -1.807126

C 4.464034 0.394296 -1.746217

C 3.850232 0.663995 -0.513095

C 4.606594 0.876543 0.644743

C 5.994613 0.823038 0.571869

C 6.609804 0.557747 -0.650965

H 6.341797 0.136100 -2.748620

H 3.846258 0.230454 -2.620068

H 4.088657 1.075716 1.575458

H 6.594069 0.985153 1.459546

H 7.691679 0.514864 -0.709825

N 2.441832 0.738726 -0.329153

N 2.325703 -3.001044 0.022936

N 1.774529 -3.916573 0.263383

N 1.094613 1.180124 4.074426

N 2.114024 1.194093 3.673669

O 1.768732 0.559033 -1.334075

C -5.698535 -0.594396 0.271872

C -5.078336 0.524984 -0.275736

C -3.706235 0.563057 -0.488935

C -2.935436 -0.552011 -0.143525

C -3.544560 -1.683179 0.407147

C -4.919816 -1.699975 0.611961

H -6.769880 -0.598544 0.427849

H -3.237360 1.438511 -0.915348

H -2.926728 -2.533660 0.665638

H -5.395447 -2.575360 1.038758

Cl -6.055205 1.924207 -0.708300

C -1.453808 -0.571042 -0.350220

O -0.751931 -1.518419 -0.069472

O -0.985499 0.568177 -0.878959

H -0.010244 0.500637 -0.999723


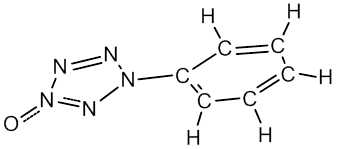


B3LYP/6-311++G**

Total electronic energy = -580.627181 Ha

Zero-point correction = 0.118692 Ha

Thermal correction to enthalpy = 0.128314 Ha

Thermal correction to free energy = 0.082748 Ha

C -2.945826 -1.054028 0.000236

C -1.562697 -1.196201 0.000202

C -0.772842 -0.046633 -0.000034

C -1.333636 1.229450 -0.000214

C -2.720506 1.348570 -0.000163

C -3.528550 0.213349 0.000054

H -3.569155 -1.940205 0.000420

H -1.100571 -2.173975 0.000353

H -0.696420 2.103216 -0.000393

H -3.166742 2.335807 -0.000302

H -4.607295 0.314522 0.000090

N 0.648857 -0.181350 -0.000087

N 1.479939 0.862667 0.000218

N 2.672135 0.278128 0.000102

N 2.540762 -1.111115 -0.000251

N 1.277695 -1.346173 -0.000371

O 3.748601 0.859811 0.000259


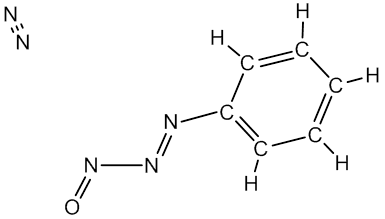


B3LYP/6-311++G**

Total electronic energy = -580.674971 Ha

Zero-point correction = 0.110949 Ha

Thermal correction to enthalpy = 0.124952 Ha

Thermal correction to free energy = 0.062386 Ha

C 2.923689 1.490478 -0.447495

C 1.589838 1.159812 -0.665223

C 1.087564 -0.056338 -0.189698

C 1.919212 -0.947847 0.507183

C 3.247071 -0.609069 0.719455

C 3.750795 0.607183 0.244022

H 3.316307 2.431326 -0.814519

H 0.919753 1.822950 -1.198947

H 1.512605 -1.884051 0.867390

H 3.897717 -1.289737 1.256273

H 4.790494 0.862356 0.415378

N -0.282168 -0.292276 -0.466442

N -0.751706 -1.361683 -0.061819

N -2.231281 -1.359690 -0.490907

N -4.199376 2.351086 0.078842

N -3.896605 1.662994 0.875722

O -2.752241 -2.351397 -0.135350


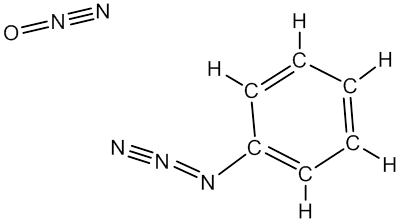


B3LYP/6-311++G**

Total electronic energy = -580.662401 Ha

Zero-point correction = 0.114476 Ha

Thermal correction to enthalpy = 0.127192 Ha

Thermal correction to free energy = 0.069577 Ha

C -3.769597 -0.190258 0.000212

C -2.891899 0.888103 0.000209

C -1.513241 0.658462 -0.000018

C -1.016448 -0.649535 -0.000260

C -1.907328 -1.719941 -0.000253

C -3.283424 -1.498019 -0.000021

H -4.838129 -0.007682 0.000389

H -3.254843 1.908572 0.000385

H 0.051963 -0.835439 -0.000448

H -1.519289 -2.732160 -0.000432

H -3.970493 -2.335876 -0.000020

N -0.686836 1.815592 0.000019

N 3.028575 -1.392557 0.000331

N 3.992789 -0.811775 0.000131

N 1.668441 1.674335 -0.000170

N 0.534303 1.670928 -0.000113

O 5.007687 -0.203243 -0.000059


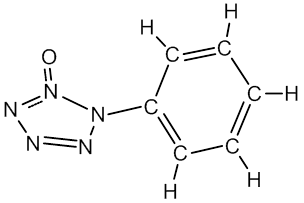


Total electronic energy = -580.618050 Ha

Zero-point correction = 0.118522 Ha

Thermal correction to enthalpy = 0.128110 Ha

Thermal correction to free energy = 0.083508 Ha

C -2.682979 -1.163072 -0.340695

C -1.296239 -1.252629 -0.309018

C -0.557330 -0.126170 0.057840

C -1.174288 1.080565 0.387751

C -2.564005 1.150515 0.339520

C -3.318804 0.036314 -0.020600

H -3.265608 -2.031924 -0.622461

H -0.787577 -2.175219 -0.554947

H -0.584565 1.942977 0.661168

H -3.054804 2.083391 0.589620

H -4.399954 0.101689 -0.053333

N 0.861416 -0.254794 0.117538

N 1.785635 0.714147 -0.195063

N 2.982781 0.150027 -0.090768

N 2.779588 -1.119296 0.253262

N 1.521036 -1.386463 0.384577

O 1.517648 1.875073 -0.499457


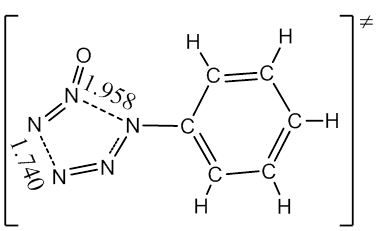


B3LYP/6-311++G**

Imaginary frequency = 496.1i cm^-1^

Total electronic energy = -580.582533 Ha

Zero-point correction = 0.114878 Ha

Thermal correction to enthalpy = 0.125100 Ha

Thermal correction to free energy = 0.078962 Ha

C -2.679553 -1.064632 -0.602527

C -1.322029 -1.219094 -0.352155

C -0.621245 -0.187943 0.289967

C -1.273556 0.994589 0.656403

C -2.627635 1.143982 0.375044

C -3.334626 0.116497 -0.247502

H -3.227860 -1.866215 -1.083811

H -0.805512 -2.129343 -0.632336

H -0.712666 1.778402 1.148995

H -3.132859 2.061579 0.652534

H -4.391937 0.232431 -0.454706

N 0.756789 -0.254153 0.548652

N 2.114220 0.847736 -0.332852

N 3.055188 0.119496 -0.367971

N 2.580618 -1.453993 0.205888

N 1.427747 -1.337244 0.530676

O 1.735095 1.971232 -0.554602


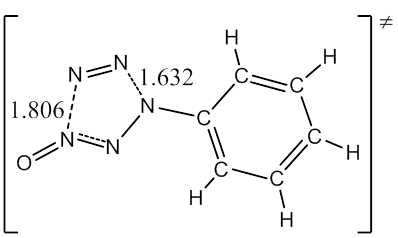


B3LYP/6-311++G**

Imaginary frequency = 489.0i cm^-1^

Total electronic energy = -580.602562 Ha

Zero-point correction = 0.115138 Ha

Thermal correction to enthalpy = 0.125151 Ha

Thermal correction to free energy = 0.079751 Ha

C 3.006228 0.901508 -0.202044

C 1.656845 1.128898 -0.448281

C 0.739910 0.096719 -0.242581

C 1.162473 -1.161250 0.191170

C 2.519125 -1.381517 0.413572

C 3.441510 -0.353624 0.224606

H 3.720274 1.702097 -0.355350

H 1.305060 2.092808 -0.794621

H 0.440724 -1.955330 0.334074

H 2.853460 -2.359548 0.739646

H 4.495487 -0.530563 0.404079

N -0.634816 0.391217 -0.504482

N -1.485221 -0.612698 -0.628176

N -2.594921 -0.431681 -0.051623

N -2.396371 1.159467 0.778654

N -1.285705 1.451126 0.552952

O -3.649039 -1.004488 -0.122219


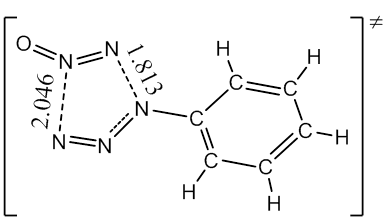


B3LYP/6-311++G**

Imaginary frequency = 579.3i cm^-1^

Total electronic energy = -580.575862 Ha

Zero-point correction = 0.114370 Ha

Thermal correction to enthalpy = 0.124733 Ha

Thermal correction to free energy = 0.078249 Ha

C -2.868583 -1.163187 -0.220082

C -1.543889 -1.098077 -0.638926

C -0.789325 0.040342 -0.348755

C -1.351981 1.115346 0.346845

C -2.685080 1.048366 0.736026

C -3.443068 -0.090378 0.460744

H -3.455783 -2.047730 -0.436519

H -1.083997 -1.915002 -1.180478

H -0.757111 1.994729 0.563086

H -3.130723 1.884754 1.261593

H -4.479429 -0.139033 0.773724

N 0.570125 0.021542 -0.757028

N 1.601291 -1.076242 0.253402

N 2.636584 -0.500279 0.390088

N 2.370873 1.319743 -0.506477

N 1.260897 1.111485 -0.820961

O 3.740026 -0.628241 0.886288


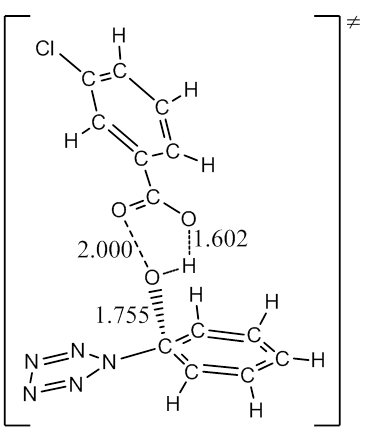


B3LYP/6-311++G**

Imaginary frequency = 471.2i cm^-1^

Total electronic energy = -1461.112932 Ha

Zero-point correction = 0.220954 Ha

Thermal correction to enthalpy = 0.240648 Ha

Thermal correction to free energy = 0.168945 Ha

C 3.404593 -2.419474 -0.733962

C 3.110078 -1.106304 -0.999115

C 3.040629 -0.159677 0.086145

C 3.445132 -0.587625 1.397319

C 3.744790 -1.919865 1.626068

C 3.731858 -2.830844 0.573460

H 3.401898 -3.140620 -1.541590

H 2.887789 -0.759894 -1.999344

H 3.457740 0.147544 2.189524

H 3.995333 -2.243299 2.628384

H 3.976614 -3.870173 0.758736

N 3.259655 1.220165 -0.253237

N 3.609132 2.157060 0.620856

N 3.646689 3.254980 -0.059393

N 3.318192 2.990289 -1.343947

N 3.074737 1.727326 -1.466458

C -5.346873 -0.707945 -0.501681

C -4.727487 0.368206 0.128375

C -3.350348 0.411796 0.302100

C -2.575430 -0.652017 -0.169469

C -3.180448 -1.739952 -0.803558

C -4.562219 -1.761961 -0.966711

H -6.422368 -0.717738 -0.625227

H -2.877344 1.251991 0.791658

H -2.558321 -2.550125 -1.160429

H -5.037305 -2.602937 -1.458701

Cl -5.712568 1.702144 0.717242

C -1.077661 -0.642623 -0.001590

O -0.388190 -1.581845 -0.451578

O -0.588395 0.363679 0.619089

O 1.357643 -0.094879 0.580631

H 0.942651 -0.905876 0.129704


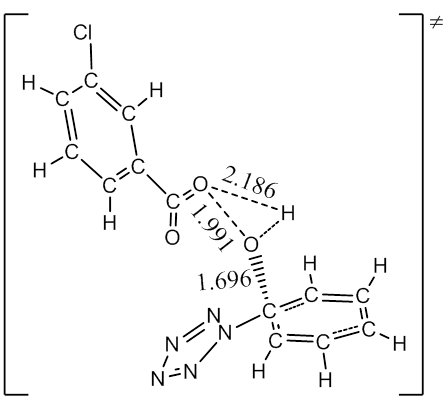


B3LYP/6-311++G**

Imaginary frequency = 465.9i cm^-1^

Total electronic energy = -1461.102993 Ha

Zero-point correction = 0.220687 Ha

Thermal correction to enthalpy = 0.240616 Ha

Thermal correction to free energy = 0.168990 Ha

C -3.169496 -2.356487 0.970506

C -2.764967 -1.047923 1.003785

C -2.911562 -0.236451 -0.191614

C -3.708574 -0.757909 -1.280464

C -4.086895 -2.084497 -1.275209

C -3.816972 -2.884320 -0.161868

H -3.004097 -2.985203 1.836549

H -2.283676 -0.611430 1.865123

H -3.945854 -0.096024 -2.104236

H -4.619613 -2.493066 -2.124880

H -4.134868 -3.920448 -0.158970

N -3.011807 1.190330 0.035155

N -3.009619 2.086731 -0.943689

N -3.110541 3.232678 -0.353254

N -3.174201 3.035646 0.981168

N -3.112884 1.766725 1.226326

C 5.131166 -0.072620 1.041370

C 4.660862 0.028527 -0.264395

C 3.304908 -0.048272 -0.557332

C 2.395651 -0.235188 0.487742

C 2.850485 -0.339744 1.804134

C 4.213292 -0.256725 2.073958

H 6.192769 -0.008066 1.243237

H 2.953126 0.034455 -1.575888

H 2.127464 -0.485077 2.595577

H 4.568867 -0.336011 3.095007

Cl 5.811558 0.260258 -1.577845

C 0.892847 -0.332644 0.225479

O 0.136682 -0.566773 1.167729

O 0.590863 -0.144304 -1.008391

O -1.379882 -0.404250 -0.899500

H -1.443010 -0.040149 -1.803365

B3LYP/6-311++G**

Imaginary frequency = 526.0i cm^-1^

Total electronic energy = -1461.084609 Ha

Zero-point correction = 0.220761 Ha

Thermal correction to enthalpy = 0.240407 Ha

Thermal correction to free energy = 0.169194 Ha

C -3.632123 2.854531 -0.180660

C -3.007805 1.665674 -0.544858

C -3.480658 0.478679 0.001479

C -4.542522 0.442512 0.899213

C -5.143516 1.644827 1.266523

C -4.693520 2.846912 0.725040

H -3.278764 3.790350 -0.596946

H -2.160643 1.637367 -1.215678

H -4.885738 -0.497847 1.311285

H -5.964653 1.636365 1.973208

H -5.167361 3.778819 1.010889

N -2.864790 -0.771915 -0.420874

N -2.879310 -1.869247 0.444291

N -3.060837 -2.892508 -0.238714

N -3.257306 -2.499218 -1.622523

N -3.176581 -1.265332 -1.706540

C 5.479086 -0.098458 0.981484

C 4.907971 0.431669 -0.173818

C 3.547993 0.317233 -0.423088

C 2.742435 -0.344542 0.509833

C 3.299170 -0.882789 1.674219

C 4.665815 -0.755828 1.902429

H 6.543176 0.002608 1.153514

H 3.106996 0.727666 -1.321526

H 2.663055 -1.393207 2.384904

H 5.105988 -1.170322 2.801660

Cl 5.930967 1.260365 -1.336683

C 1.288002 -0.463683 0.236685

O 0.570163 -1.096874 1.133785

O 0.769557 0.005770 -0.782968

O -1.132477 -0.486954 -0.592692

H -0.356645 -1.046407 0.720932

B3LYP/6-311++G**

Imaginary frequency = 670.1i cm^-1^

Total electronic energy = -1461.065327 Ha

Zero-point correction = 0.217391 Ha

Thermal correction to enthalpy = 0.237743 Ha

Thermal correction to free energy = 0.165581 Ha

C -3.280903 2.662648 -0.016434

C -2.650922 1.424409 0.118972

C -3.431202 0.288290 -0.047722

C -4.795288 0.337827 -0.336077

C -5.398188 1.582732 -0.465844

C -4.640229 2.744205 -0.304866

H -2.691482 3.563232 0.105003

H -1.587726 1.357358 0.336545

H -5.376059 -0.568607 -0.460556

H -6.455485 1.644135 -0.692928

H -5.114479 3.713348 -0.407737

N -2.831787 -1.044445 0.103236

N -3.219979 -1.797150 1.281134

N -3.465226 -2.945486 0.947091

N -3.310730 -3.058659 -0.561367

N -2.993385 -1.964282 -1.002936

C 5.515522 1.079472 0.064445

C 4.973564 -0.192244 -0.095795

C 3.600605 -0.403916 -0.090461

C 2.746289 0.688871 0.084473

C 3.274694 1.972146 0.246002

C 4.653076 2.161751 0.235362

H 6.589310 1.217549 0.056509

H 3.189547 -1.395928 -0.215041

H 2.592527 2.800918 0.383817

H 5.065765 3.156166 0.362815

Cl 6.056068 -1.566531 -0.310574

C 1.238794 0.510788 0.104893

O 0.510481 1.465415 0.370230

O 0.856193 -0.692569 -0.191907

O -1.180577 -0.962742 0.323419

H -0.740965 -0.809708 -0.554398

B3LYP/6-311++G**

Imaginary frequency = 513.8i cm^-1^

Total electronic energy = -1461.113323 Ha

Zero-point correction = 0.221598 Ha

Thermal correction to enthalpy = 0.240824 Ha

Thermal correction to free energy = 0.170589 Ha

C -4.102016 2.904154 -0.722900

C -4.275186 1.527025 -0.793406

C -3.434355 0.712945 -0.035394

C -2.434522 1.230063 0.785359

C -2.274445 2.611071 0.833555

C -3.102647 3.446088 0.085400

H -4.742835 3.551935 -1.308434

H -5.038019 1.087742 -1.422329

H -1.791019 0.584516 1.367939

H -1.498233 3.030051 1.461794

H -2.968669 4.520370 0.130593

N -3.637468 -0.703968 -0.106427

N -2.704178 -1.641306 0.048015

N -3.279003 -2.795257 -0.090636

N -4.572365 -2.572955 -0.304353

N -4.804953 -1.285778 -0.312813

C 5.330109 0.328324 0.884725

C 4.796347 0.155090 -0.389727

C 3.463856 -0.186706 -0.577487

C 2.643267 -0.355079 0.541901

C 3.162925 -0.184850 1.827605

C 4.502602 0.154263 1.992931

H 6.373199 0.592538 1.004395

H 3.055513 -0.325439 -1.569398

H 2.509495 -0.324987 2.678828

H 4.911774 0.284143 2.988333

Cl 5.837103 0.373640 -1.793323

C 1.198784 -0.724257 0.364221

O 0.448179 -0.814970 1.377546

O 0.779460 -0.935393 -0.815092

O -1.129389 -1.354633 -0.411415

H -0.690490 -1.226709 0.529244

B3LYP/6-311++G**

Imaginary frequency = 562.5i cm^-1^

Total electronic energy = -1461.099695 Ha

Zero-point correction = 0.221017 Ha

Thermal correction to enthalpy = 0.241025 Ha

Thermal correction to free energy = 0.169057 Ha

C -5.104471 2.204413 -0.720633

C -4.938683 0.845689 -0.492255

C -3.677191 0.388962 -0.098959

C -2.583795 1.235024 0.075630

C -2.781550 2.593465 -0.167074

C -4.026579 3.078088 -0.559382

H -6.072334 2.578625 -1.031101

H -5.760011 0.152259 -0.615482

H -1.598240 0.886941 0.377967

H -1.941674 3.265108 -0.039500

H -4.161511 4.137788 -0.742419

N -3.560272 -1.023183 0.162901

N -2.533895 -1.824167 -0.107385

N -2.841582 -3.033839 0.242031

N -4.094381 -2.997140 0.683211

N -4.541624 -1.771018 0.648649

C 5.468212 1.112326 0.402230

C 5.047148 -0.096565 -0.143253

C 3.701381 -0.430755 -0.222348

C 2.745608 0.469548 0.257421

C 3.153378 1.687448 0.807070

C 4.507123 2.003287 0.878261

H 6.523500 1.348699 0.452820

H 3.384622 -1.372727 -0.647751

H 2.395854 2.368107 1.173487

H 4.823705 2.947839 1.306505

Cl 6.254573 -1.231918 -0.749812

C 1.260820 0.141947 0.190994

O 0.446048 0.968999 0.614496

O 0.996534 -1.006480 -0.321078

O -1.071617 -1.237343 -0.256492

H -0.515497 -2.023279 -0.435192

B3LYP/6-311++G**

Imaginary frequency = 494.2i cm^-1^

Total electronic energy = -1461.119647 Ha

Zero-point correction = 0.221857 Ha

Thermal correction to enthalpy = 0.241188 Ha

Thermal correction to free energy = 0.168742 Ha

C 7.470607 -0.056962 -0.073334

C 6.214426 -0.560960 -0.393981

C 5.090505 0.153561 0.015704

C 5.185372 1.345717 0.731046

C 6.451442 1.828215 1.045219

C 7.591579 1.132084 0.644878

H 8.355232 -0.599679 -0.383252

H 6.104563 -1.484443 -0.946283

H 4.292330 1.878206 1.029987

H 6.543787 2.753485 1.600611

H 8.573887 1.516350 0.892405

N 3.792176 -0.356063 -0.313004

N 2.672545 0.055518 0.249603

N 1.766330 -0.662513 -0.330269

N 2.289805 -1.487723 -1.240075

N 3.568739 -1.307668 -1.226187

C -6.520399 -0.466897 0.588987

C -5.861708 0.589557 -0.034103

C -4.482945 0.591407 -0.196629

C -3.742939 -0.495266 0.278519

C -4.387955 -1.563401 0.906482

C -5.771324 -1.544880 1.058537

H -7.596825 -0.444077 0.702736

H -3.976297 1.414495 -0.681914

H -3.792790 -2.393016 1.265419

H -6.276130 -2.372009 1.544718

Cl -6.803078 1.955924 -0.629968

C -2.247910 -0.519575 0.115386

O -1.590441 -1.501669 0.546587

O -1.703425 0.474614 -0.462788

O 0.189637 -0.088435 -0.394178

H -0.301259 -0.882545 0.067330

The xyz coordinates of geometries in Fig. 8:

­­­___________________________________________________________

B3LYP/6-311++G**

Total electronic energy = -1535.987656 Ha

Zero-point correction = 0.213359 Ha

Thermal correction to enthalpy = 0.235897 Ha

Thermal correction to free energy = 0.154129 Ha

C -4.755557 -1.489809 0.942833

C -3.884698 -0.604809 1.444560

C -2.491694 -0.542894 0.942888

C -2.177669 -1.295521 -0.294118

C -3.055900 -2.175357 -0.793967

C -4.394571 -2.366403 -0.193052

H -5.767147 -1.588150 1.320707

H -4.126838 0.067829 2.257990

H -1.185024 -1.158174 -0.706248

H -2.832432 -2.783681 -1.663394

N -3.311397 1.441541 -0.476411

N -2.865343 1.909659 -1.630122

N -3.423337 3.092786 -1.816900

N -4.210730 3.349769 -0.777278

N -4.141011 2.326611 0.054336

C 5.838681 0.894427 0.382923

C 5.357023 -0.199017 -0.331761

C 4.020013 -0.564922 -0.285972

C 3.137488 0.179878 0.501810

C 3.603670 1.280278 1.226269

C 4.949318 1.632469 1.161869

H 6.886564 1.161172 0.327485

H 3.643217 -1.410361 -0.846181

H 2.907733 1.850216 1.826903

H 5.313187 2.488486 1.718918

Cl 6.478937 -1.138716 -1.319557

C 1.693967 -0.239927 0.532769

O 0.966088 0.497057 1.359966

O 1.270178 -1.162564 -0.135129

O -1.605248 -0.049922 1.642930

H 0.003570 0.218781 1.359211

O -5.176324 -3.207179 -0.625342

B3LYP/6-311++G**

Total electronic energy = -1535.940513 Ha

Zero-point correction = 0.215633 Ha

Thermal correction to enthalpy = 0.236328 Ha

Thermal correction to free energy = 0.159760 Ha

C 6.211870 -0.818307 0.761901

C 5.134758 0.003999 0.972967

C 4.137899 0.134678 -0.018433

C 4.249541 -0.586357 -1.226780

C 5.325760 -1.409755 -1.441280

C 6.387980 -1.584356 -0.462781

H 6.980152 -0.921845 1.520804

H 5.037630 0.559773 1.898011

H 3.477089 -0.482184 -1.979680

H 5.414529 -1.966225 -2.368183

N 3.045062 0.985211 0.197863

N 2.072761 1.178271 -0.691484

N 1.288836 2.052529 -0.061795

N 1.749799 2.378374 1.142392

N 2.861497 1.706221 1.312337

O 0.233695 2.535235 -0.578504

C -5.912502 -1.718115 0.520422

C -5.757426 -0.545148 -0.211555

C -4.531846 0.101840 -0.303672

C -3.427473 -0.442690 0.359235

C -3.566723 -1.619865 1.098452

C -4.803370 -2.251874 1.176196

H -6.879801 -2.201553 0.574322

H -4.422421 1.012419 -0.875894

H -2.694747 -2.019397 1.600774

H -4.912142 -3.166082 1.748858

Cl -7.158373 0.136270 -1.044546

C -2.073526 0.212358 0.300491

O -1.109898 -0.243938 0.880436

O -2.064526 1.313486 -0.438685

H -1.143438 1.727566 -0.453317

O 7.373248 -2.332501 -0.657572

B3LYP/6-311++G**

Total electronic energy = -1535.928058 Ha

Zero-point correction = 0.215307 Ha

Thermal correction to enthalpy = 0.236200 Ha

Thermal correction to free energy = 0.159815 Ha

C -6.358397 -0.460287 -0.416918

C -5.398117 0.516741 -0.431946

C -4.051666 0.202068 -0.128923

C -3.702315 -1.127839 0.205713

C -4.666528 -2.104244 0.224811

C -6.062767 -1.845794 -0.083082

H -7.387070 -0.222219 -0.666549

H -5.655306 1.539654 -0.680314

H -2.675215 -1.367589 0.440552

H -4.400595 -3.124609 0.479702

N -3.125881 1.256249 -0.091788

N -1.777883 1.186392 -0.239478

N -1.289239 2.427661 -0.203059

N -2.320233 3.228084 -0.066202

N -3.442993 2.558076 0.019047

C 6.407426 -0.473620 0.625720

C 5.639379 -0.426055 -0.533451

C 4.259163 -0.276264 -0.489268

C 3.629174 -0.170192 0.754800

C 4.386600 -0.215993 1.927749

C 5.767669 -0.367248 1.860345

H 7.481800 -0.591082 0.561343

H 3.672284 -0.240315 -1.396264

H 3.872634 -0.130816 2.876860

H 6.356492 -0.403132 2.770085

Cl 6.443871 -0.559728 -2.099701

C 2.136951 -0.005759 0.869575

O 1.577945 0.095200 1.940681

O 1.535101 0.010969 -0.314261

H 0.545009 0.121638 -0.230321

O -1.119766 0.115327 -0.395047

O -6.940991 -2.738306 -0.068946

B3LYP/6-311++G**

Total electronic energy = -1535.913786 Ha

Total electronic energy solvent (water) = -1535.983970 Ha

Total electronic energy solvent (methanol) = -1535.982409 Ha

Total electronic energy solvent (acetonitrile) = -1535.982638 Ha

Total electronic energy RI-B2KPLYP/ma-def2-TZVP = -1534.862574 Ha

Zero-point correction = 0.214068 Ha

Thermal correction to enthalpy = 0.235019 Ha

Thermal correction to free energy = 0.156391 Ha

C 2.120805 1.039416 0.041224

C 3.311696 0.649376 0.619931

C 4.264094 -0.051381 -0.134061

C 4.006358 -0.356293 -1.478992

C 2.816565 0.033897 -2.057614

C 1.812848 0.751688 -1.329408

H 1.387485 1.583992 0.625473

H 3.518134 0.881954 1.657338

H 4.746817 -0.897458 -2.055010

H 2.610015 -0.197192 -3.096581

N 5.489211 -0.451391 0.461989

N 6.452541 -1.117372 -0.170563

N 7.395779 -1.286884 0.720755

N 7.016297 -0.736513 1.871351

N 5.826897 -0.210039 1.726464

C -6.037778 -1.240007 0.816446

C -4.881146 -1.517162 0.093646

C -3.904517 -0.553650 -0.123775

C -4.094182 0.731106 0.399187

C -5.250815 1.024707 1.125897

C -6.214163 0.042664 1.332535

H -6.780713 -2.012417 0.970172

H -3.011739 -0.788340 -0.686609

H -5.372373 2.026779 1.517229

H -7.110679 0.271133 1.898158

Cl -4.653735 -3.139266 -0.563679

C -3.092943 1.842743 0.209887

O -3.260480 2.965132 0.621095

O -2.033128 1.387505 -0.463793

O -1.043144 2.404469 -0.692477

O 0.714415 1.105942 -1.892818

H -0.300762 1.827440 -1.183055

B3LYP/6-311++G**

Total electronic energy = -655.417878 Ha

Zero-point correction = 0.103175 Ha

Thermal correction to enthalpy = 0.117453 Ha

Thermal correction to free energy = 0.055951 Ha

C 2.419662 1.426739 0.009295

C 1.077582 1.199128 0.012297

C 0.531483 -0.127168 0.004440

C 1.457427 -1.230100 -0.006901

C 2.799581 -1.016038 -0.009944

C 3.382292 0.329098 -0.002117

H 2.825543 2.432959 0.015270

H 0.365805 2.018868 0.020818

H 1.045801 -2.232163 -0.012827

H 3.502537 -1.843333 -0.018421

N -0.804917 -0.224871 0.008465

N -1.311614 -1.429549 0.001247

N -2.669676 -1.304495 0.007233

N -3.936389 2.270443 -0.557289

N -3.950284 2.278590 0.537916

O -3.243142 -2.390496 0.001252

O 4.613430 0.515858 -0.005034

B3LYP/6-311++G**

Total electronic energy = -655.359882 Ha

Zero-point correction = 0.104529 Ha

Thermal correction to enthalpy = 0.118216 Ha

Thermal correction to free energy = 0.059030 Ha

C 3.588008 0.839836 0.000131

C 2.344641 1.443795 0.000328

C 1.169440 0.677456 0.000200

C 1.275817 -0.723311 -0.000136

C 2.515337 -1.337778 -0.000341

C 3.761140 -0.597936 -0.000223

H 4.489791 1.445100 0.000234

H 2.256901 2.527186 0.000583

H 0.374284 -1.332663 -0.000242

H 2.585979 -2.421572 -0.000601

N -0.074125 1.381755 0.000384

N -4.809735 -1.629383 0.001324

N -5.103699 -0.544040 -0.000070

N -2.201701 0.352940 0.000312

N -1.139587 0.794349 0.000336

O -5.435568 0.593390 -0.001553

O 4.894152 -1.158361 -0.000412

B3LYP/6-311++G**

Total electronic energy = -655.350603 Ha

Zero-point correction = 0.109330 Ha

Thermal correction to enthalpy =0.119863 Ha

Thermal correction to free energy = 0.072651 Ha

C 2.543504 1.145115 -0.000015

C 1.173367 1.245883 0.000031

C 0.376616 0.082932 0.000109

C 0.991174 -1.185248 0.000235

C 2.361850 -1.290008 0.000177

C 3.238656 -0.131672 -0.000047

H 3.158067 2.039466 -0.000168

H 0.692553 2.217122 -0.000047

H 0.371295 -2.074241 0.000396

H 2.835658 -2.266138 0.000231

N -1.027702 0.192231 0.000051

N -1.844784 -0.864200 -0.000212

N -3.055703 -0.288863 -0.000143

N -2.968038 1.055974 0.000104

N -1.687480 1.346982 0.000110

O -4.125753 -0.927602 -0.000099

O 4.490425 -0.224036 -0.000242

B3LYP/6-311++G**

Total electronic energy = -655.339855 Ha

Zero-point correction = 0.109065 Ha

Thermal correction to enthalpy = 0.119616 Ha

Thermal correction to free energy = 0.072844 Ha

C -2.315167 -1.217682 0.262861

C -0.943525 -1.277804 0.256912

C -0.177324 -0.121262 -0.008893

C -0.832519 1.100539 -0.278145

C -2.206400 1.159449 -0.273734

C -3.048510 0.007862 -0.007531

H -2.900874 -2.104970 0.480885

H -0.431810 -2.212453 0.456002

H -0.246970 1.987596 -0.475510

H -2.709394 2.099688 -0.475340

N 1.223813 -0.254179 -0.078572

N 2.160650 0.717643 0.148869

N 3.365849 0.143757 0.039238

N 3.155787 -1.132732 -0.208989

N 1.879910 -1.403351 -0.301919

O -4.302838 0.062462 -0.004866

O 1.918795 1.915733 0.394212

B3LYP/6-311++G**

Total electronic energy = -580.166639 Ha

Total electronic energy solvent (water) = -580.242023 Ha

Total electronic energy solvent (methanol) = -580.240502 Ha

Total electronic energy solvent (acetonitrile) = -580.240725 Ha

Total electronic energy RI-B2KPLYP/ma-def2-TZVP = -579.693248 Ha

Zero-point correction = 0.105278 Ha

Thermal correction to enthalpy = 0.114918 Ha

Thermal correction to free energy = 0.069993 Ha

C -2.067634 -1.219401 0.000110

C -0.692019 -1.216899 0.000311

C 0.017357 0.000000 0.000176

C -0.692019 1.216899 0.000312

C -2.067634 1.219401 0.000111

C -2.856508 0.000000 -0.000552

H -2.612541 -2.158054 0.000293

H -0.140216 -2.149732 0.000578

H -0.140216 2.149732 0.000579

H -2.612541 2.158054 0.000294

N 1.428833 0.000000 -0.000025

N 3.425406 -0.662618 -0.000116

N 2.186134 -1.098821 -0.000089

N 3.425406 0.662618 -0.000118

N 2.186134 1.098821 -0.000089

O -4.113393 0.000000 -0.000186

B3LYP/6-311++G**

Total electronic energy = -580.199197 Ha

Zero-point correction = 0.098895 Ha

Thermal correction to enthalpy = 0.112383 Ha

Thermal correction to free energy = 0.052824 Ha

C 2.504830 0.878034 -0.000533

C 1.165533 1.226955 -0.000249

C 0.166088 0.240138 0.000379

C 0.551195 -1.108771 0.000718

C 1.886421 -1.468170 0.000435

C 2.960236 -0.496936 -0.000215

H 3.268646 1.650244 -0.001016

H 0.885229 2.278412 -0.000517

H -0.224368 -1.870321 0.001221

H 2.169897 -2.516658 0.000707

N -1.236288 0.524607 0.000693

N -2.147030 2.706955 0.000293

N -1.646147 1.672663 0.000467

N -4.471809 -1.131188 -0.000407

N -4.066415 -2.148869 -0.001020

O 4.183573 -0.817294 -0.000472

B3LYP/6-311++G**

Imaginary frequency = 429.6i cm^-1^

Total electronic energy = -655.304663 Ha

Zero-point correction = 0.104801 Ha

Thermal correction to enthalpy = 0.116177 Ha

Thermal correction to free energy = 0.067350 Ha

C -2.393876 0.912591 -0.756959

C -1.054681 0.810317 -1.026166

C -0.247805 -0.175105 -0.397859

C -0.863632 -1.048264 0.542024

C -2.203808 -0.968863 0.806154

C -3.067900 0.022297 0.174397

H -3.003616 1.668898 -1.240452

H -0.573572 1.485152 -1.727364

H -0.249755 -1.796881 1.036048

H -2.675499 -1.650635 1.506831

N 1.090067 -0.192895 -0.672629

N 2.494810 0.830308 0.457773

N 3.451814 0.120219 0.485852

N 2.945344 -1.426505 -0.310094

N 1.822878 -1.260763 -0.699644

O 2.152022 1.968001 0.775858

O -4.294740 0.092385 0.417466

B3LYP/6-311++G**

Imaginary frequency = 606.3i cm^-1^

Total electronic energy = -655.318093 Ha

Zero-point correction = 0.105379 Ha

Thermal correction to enthalpy = 0.116362 Ha

Thermal correction to free energy = 0.068648 Ha

C 2.618020 1.038322 -0.401806

C 1.260744 1.209241 -0.541968

C 0.362046 0.173690 -0.214601

C 0.877129 -1.049808 0.266946

C 2.232428 -1.238894 0.396410

C 3.201835 -0.203705 0.075282

H 3.305709 1.837789 -0.658772

H 0.856627 2.148680 -0.908886

H 0.184454 -1.847493 0.520274

H 2.625954 -2.185326 0.753985

N -1.012384 0.415612 -0.334619

N -1.856496 -0.544266 -0.680700

N -2.978621 -0.463374 -0.084469

N -2.893569 0.960398 0.867176

N -1.760313 1.300294 0.794455

O -4.038009 -1.029300 -0.338354

O 4.440975 -0.371620 0.198220

B3LYP/6-311++G**

Imaginary frequency = 548.7i cm^-1^

Total electronic energy = -580.129809 Ha

Total electronic energy solvent (water) = -580.205408 Ha

Total electronic energy solvent (methanol) = -580.203863 Ha

Total electronic energy solvent (acetonitrile) = -580.204091 Ha

Total electronic energy RI-B2KPLYP/ma-def2-TZVP = -579.651327 Ha

Zero-point correction = 0.100883 Ha

Thermal correction to enthalpy = 0.111238 Ha

Thermal correction to free energy = 0.064289 Ha

C 2.054780 -1.204865 0.260002

C 0.682742 -1.154628 0.191525

C 0.012461 0.050818 -0.116636

C 0.782326 1.216377 -0.319030

C 2.155363 1.183723 -0.242797

C 2.891333 -0.034572 0.048086

H 2.563638 -2.137543 0.483537

H 0.090636 -2.049802 0.364437

H 0.264180 2.145715 -0.540137

H 2.740110 2.083940 -0.404911

N -1.379538 0.117376 -0.148160

N -3.402216 -0.477675 -0.756990

N -2.208399 -0.417735 -0.995525

N -3.498753 0.262165 0.796804

N -2.411239 0.547057 1.142041

O 4.146052 -0.075217 0.112873

B3LYP/6-311++G**

Imaginary frequency = 358.3i cm^-1^

Total electronic energy = -1535.864401 Ha

Zero-point correction = 0.213093 Ha

Thermal correction to enthalpy = 0.233660 Ha

Thermal correction to free energy = 0.158738 Ha

C 7.095383 0.111110 -0.501338

C 5.866721 -0.385188 -0.848032

C 4.716504 -0.009087 -0.115845

C 4.836878 0.877187 0.979361

C 6.063984 1.374408 1.329874

C 7.284633 1.027837 0.614051

H 7.981557 -0.175642 -1.057094

H 5.762252 -1.069558 -1.681375

H 3.947791 1.158715 1.531396

H 6.162193 2.058798 2.165486

N 3.467391 -0.520087 -0.468459

N 2.353571 -0.373100 0.242128

N 1.457661 -1.043585 -0.451744

N 1.970237 -1.550020 -1.552181

N 3.237951 -1.258291 -1.583468

C -6.918242 -0.051933 0.737656

C -6.155850 0.764808 -0.091861

C -4.791066 0.569303 -0.256974

C -4.168790 -0.477911 0.429075

C -4.918970 -1.307495 1.265373

C -6.285867 -1.092691 1.416987

H -7.980855 0.124342 0.847999

H -4.204878 1.208964 -0.902688

H -4.410212 -2.110103 1.783982

H -6.869180 -1.735983 2.066758

Cl -6.951453 2.086016 -0.956365

C -2.682220 -0.730972 0.279681

O -2.152980 -1.674317 0.884469

O -2.067634 0.087831 -0.494918

O -0.272716 -0.462525 -0.492222

H -0.574410 -1.174161 0.124541

O 8.405105 1.480473 0.933573

B3LYP/6-311++G**

Imaginary frequency = 338.1i cm^-1^

Total electronic energy = -1535.861278 Ha

Zero-point correction = 0.212843 Ha

Thermal correction to enthalpy = 0.233367 Ha

Thermal correction to free energy = 0.159901 Ha

C -4.745483 2.134642 -0.715303

C -4.507913 0.804224 -0.904538

C -3.545218 0.129565 -0.106865

C -2.826745 0.842070 0.888034

C -3.064571 2.172261 1.083481

C -4.042961 2.915659 0.298198

H -5.474100 2.660671 -1.322084

H -5.041907 0.244163 -1.661813

H -2.090294 0.320472 1.484135

H -2.525349 2.723653 1.845266

N -3.339501 -1.221408 -0.295665

N -2.428504 -1.976211 0.373171

N -2.614692 -3.225752 -0.074951

N -3.582104 -3.218144 -0.944089

N -4.060584 -1.996306 -1.114005

C 5.777365 0.492460 0.615630

C 5.008599 0.525326 -0.543701

C 3.682466 0.114083 -0.554836

C 3.106107 -0.346511 0.632596

C 3.863194 -0.386568 1.805305

C 5.190928 0.031437 1.793871

H 6.808954 0.820679 0.594535

H 3.090173 0.143803 -1.459313

H 3.389452 -0.747507 2.709328

H 5.779093 0.001501 2.704746

Cl 5.747320 1.107904 -2.041929

C 1.659747 -0.809332 0.667979

O 1.175211 -1.210415 1.738233

O 1.043570 -0.745940 -0.450905

O -0.750484 -1.386574 -0.067087

H -0.384796 -1.528657 0.839050

O -4.269681 4.127391 0.480833

B3LYP/6-311++G**

Imaginary frequency = 360.7i cm^-1^

Total electronic energy = -1535.878333 Ha

Total electronic energy solvent (water) = -1535.952711 Ha

Total electronic energy solvent (methanol) = -1535.951092 Ha

Total electronic energy solvent (acetonitrile) = -1535.951330 Ha

Total electronic energy RI-B2KPLYP/ma-def2-TZVP = -1534.822270 Ha

Zero-point correction = 0.212728 Ha

Thermal correction to enthalpy = 0.233760 Ha

Thermal correction to free energy = 0.157632 Ha

C -3.817653 -1.518052 1.547850

C -3.454722 -0.214946 1.348379

C -3.202664 0.267895 0.037741

C -3.398792 -0.597657 -1.070818

C -3.757169 -1.904005 -0.874273

C -4.000942 -2.459066 0.450630

H -3.981287 -1.896624 2.551051

H -3.336987 0.464359 2.183431

H -3.247363 -0.206816 -2.069723

H -3.875814 -2.575263 -1.717794

N -3.094466 1.665840 -0.180894

N -2.998846 2.233370 -1.378882

N -2.889943 3.515450 -1.142941

N -2.920233 3.723363 0.171857

N -3.048928 2.575604 0.786637

C 5.649542 -0.872987 -0.235758

C 4.955099 0.254415 0.192734

C 3.567081 0.300466 0.194305

C 2.854462 -0.818050 -0.249718

C 3.535528 -1.957794 -0.683821

C 4.926540 -1.981117 -0.674996

H 6.732178 -0.880603 -0.224953

H 3.039700 1.182469 0.530787

H 2.957020 -2.808012 -1.022000

H 5.457258 -2.865045 -1.010645

Cl 5.865856 1.660283 0.748625

C 1.346447 -0.841262 -0.275880

O 0.715714 -1.794895 -0.699955

O 0.816087 0.261066 0.204978

O -0.827712 0.198147 0.141414

H -0.897736 -0.719315 -0.197189

O -4.343608 -3.647117 0.631279

B3LYP/6-311++G**

Total electronic energy = -580.0656128 Ha

Zero-point correction = 0.10038819 Ha

Thermal correction to enthalpy = 0.11012342 Ha

Thermal correction to free energy = 0.06198178 Ha

C 8.526203 -0.765705 -0.831781

C 8.138293 -0.000838 0.248545

C 6.818273 0.446073 0.263631

C 5.840006 0.208803 -0.693405

C 6.228972 -0.557817 -1.777588

C 7.582634 -1.079428 -1.898287

H 9.533302 -1.157662 -0.929153

H 8.830104 0.241404 1.049470

H 4.817495 0.595659 -0.608038

H 5.530828 -0.792753 -2.574262

N 1.907960 1.105727 0.365738

N 2.787616 1.249488 -0.615374

N 2.210416 1.925961 -1.598784

N 0.971285 2.201177 -1.224779

N 0.784607 1.694831 -0.011565

O 7.935294 -1.775542 -2.880871

B3LYP/6-311++G**

Total electronic energy = -580.062170 Ha

Zero-point correction = 0.098959 Ha

Thermal correction to enthalpy = 0.109607 Ha

Thermal correction to free energy = 0.061532 Ha

C -2.273373 1.230320 -0.018744

C -0.912067 1.189389 0.191813

C -0.296375 -0.058236 0.325914

C -0.978537 -1.273714 0.222304

C -2.340265 -1.246399 0.011994

C -3.062432 0.010987 -0.114227

H -2.808187 2.169733 -0.124827

H -0.319536 2.097498 0.248348

H -0.435842 -2.210838 0.302165

H -2.924903 -2.157988 -0.071146

N 1.824607 -0.121873 -0.523049

N 2.564635 -1.214888 -0.439682

N 3.823452 -0.826005 -0.344429

N 3.850460 0.504404 -0.358304

N 2.608434 0.941884 -0.462114

O -4.305283 0.042043 -0.301007

The xyz coordinates of geometries in Fig. 9:

­­­___________________________________________________________

B3LYP/6-311++G**

Total electronic energy = -1820.469903 Ha

Total electronic energy solvent (water) = -1820.540343 Ha

Total electronic energy solvent (methanol) = -1820.538745 Ha

Total electronic energy solvent (acetonitrile) = -1820.538979 Ha

Total electronic energy RI-B2KPLYP/ma-def2-TZVP = -1819.186623 Ha

Zero-point correction = 0.296388 Ha

Thermal correction to enthalpy = 0.324177 Ha

Thermal correction to free energy = 0.229002 Ha

C 2.735736 0.925851 0.050943

C 3.952634 0.469759 0.533594

C 4.657456 -0.515615 -0.162955

C 4.149339 -1.050920 -1.349147

C 2.935390 -0.594481 -1.832727

C 2.208133 0.397243 -1.144791

H 2.182898 1.699965 0.578027

H 4.360184 0.879765 1.448716

H 4.702615 -1.815332 -1.878825

H 2.519057 -0.996722 -2.748429

N 5.910338 -0.982218 0.337266

N 6.761168 -1.732919 -0.355036

N 7.765862 -1.943039 0.448168

N 7.535030 -1.323343 1.610420

N 6.378345 -0.724968 1.553650

C -5.027164 -3.028782 1.339638

C -4.316975 -2.925369 0.147238

C -3.892581 -1.698226 -0.345873

C -4.196525 -0.538959 0.376877

C -4.908597 -0.626304 1.575873

C -5.319185 -1.866817 2.051977

H -5.341968 -3.999502 1.701283

H -3.334866 -1.636675 -1.269585

H -5.127722 0.285860 2.116019

H -5.868898 -1.936422 2.983761

Cl -3.946597 -4.392483 -0.758127

C -3.789171 0.830128 -0.090249

O -4.094177 1.850548 0.474634

O -3.045481 0.736753 -1.210901

O -2.706052 2.015844 -1.767311

H -1.845076 2.253331 -1.261792

O 1.049668 0.788120 -1.671415

N -2.395255 4.684199 -0.052707

H -3.023406 3.885252 -0.050926

H -2.095718 4.811784 -1.013860

C -1.243066 4.433397 0.805420

H -0.646799 5.347359 0.892859

H -1.600223 4.218498 1.817676

C -0.239254 3.310331 0.452722

O 0.782847 3.236774 1.142302

O -0.519280 2.525115 -0.535055

H 0.546431 1.478424 -1.128363

B3LYP/6-311++G**

Total electronic energy = -1536.459090 Ha

Zero-point correction = 0.229632 Ha

Thermal correction to enthalpy = 0.250615 Ha

Thermal correction to free energy = 0.175019 Ha

C 5.706625 -0.936779 0.750576

C 4.759578 0.061194 0.913493

C 3.701045 0.151613 0.008631

C 3.580198 -0.738287 -1.054911

C 4.539171 -1.730228 -1.217822

C 5.602091 -1.834591 -0.316636

H 6.538605 -1.029554 1.436878

H 4.835819 0.763869 1.732543

H 2.746865 -0.658516 -1.739992

H 4.452265 -2.428628 -2.043694

N 2.722164 1.174456 0.180297

N 1.916674 1.577660 -0.792527

N 1.182548 2.491091 -0.186441

N 1.557398 2.651971 1.121735

N 2.513151 1.811522 1.323672

O 0.255549 3.124622 -0.722793

C -5.186363 -2.037674 0.586557

C -5.348346 -0.802854 -0.035634

C -4.275118 0.057007 -0.231367

C -3.005361 -0.333486 0.206973

C -2.827844 -1.570508 0.833286

C -3.916254 -2.415328 1.020498

H -6.039520 -2.689001 0.728062

H -4.414285 1.013930 -0.714239

H -1.836212 -1.849205 1.165955

H -3.783085 -3.374446 1.507350

Cl -6.948502 -0.320696 -0.585548

C -1.814453 0.550707 0.022268

O -0.692238 0.239942 0.372723

O -2.109588 1.712904 -0.566585

H -1.287592 2.253469 -0.653371

O 6.568016 -2.786777 -0.423135

H 6.404403 -3.347229 -1.189477

B3LYP/6-311++G**

Total electronic energy = -1820.533317 Ha

Zero-point correction = 0.294678 Ha

Thermal correction to enthalpy = 0.324438 Ha

Thermal correction to free energy = 0.223240 Ha

C -3.171609 -0.146086 0.892019

C -2.120458 0.419729 1.501353

C -0.737489 0.198523 1.018244

C -0.571380 -0.522338 -0.266567

C -1.625924 -1.084668 -0.873411

C -2.986342 -0.943428 -0.332313

H -4.187886 -0.042846 1.256261

H -2.217860 1.018033 2.399529

H 0.442980 -0.621518 -0.635568

H -1.533006 -1.648266 -1.794510

N -1.073494 2.276714 -0.388768

N -2.005975 2.409490 -1.310797

N -1.897369 3.628478 -1.807521

N -0.893804 4.239303 -1.184181

N -0.381953 3.399501 -0.301893

C 7.704970 -0.070383 0.445351

C 7.001329 -0.921330 -0.402673

C 5.617087 -1.000809 -0.366709

C 4.913862 -0.211277 0.547807

C 5.603970 0.647779 1.407360

C 6.993584 0.715196 1.350457

H 8.785499 -0.024097 0.394698

H 5.069133 -1.656379 -1.030459

H 5.045766 1.257243 2.105120

H 7.531451 1.384782 2.012083

Cl 7.896429 -1.919218 -1.551306

C 3.414474 -0.314455 0.562120

O 2.861101 0.422219 1.514755

O 2.802301 -1.010296 -0.224933

O 0.220123 0.430737 1.758103

H 1.861888 0.368762 1.494868

O -3.940181 -1.491411 -0.899229

N -9.355564 -1.298630 -0.815279

H -8.979451 -2.098424 -1.314444

H -9.167114 -0.493394 -1.404147

C -8.672777 -1.150139 0.464288

H -9.029522 -0.250180 0.970387

H -8.946759 -1.987117 1.116516

C -7.143057 -1.086165 0.478290

O -6.522275 -0.726626 1.457413

O -6.593877 -1.487631 -0.663882

H -5.596248 -1.440718 -0.630562

B3LYP/6-311++G**

Total electronic energy = -939.943999 Ha

Zero-point correction = 0.187890 Ha

Thermal correction to enthalpy = 0.207584 Ha

Thermal correction to free energy = 0.133407 Ha

C 0.109972 0.829251 0.671192

C -1.047224 1.084295 1.299700

C -2.192938 1.703066 0.582205

C -2.108482 1.727855 -0.902473

C -0.950847 1.473630 -1.529093

C 0.236382 1.039361 -0.780400

H 0.988990 0.456711 1.186230

H -1.177497 0.932259 2.364710

H -3.009927 2.018398 -1.427727

H -0.844194 1.532685 -2.606269

N -3.114731 -0.743956 0.113421

N -3.179465 -1.766531 0.950465

N -4.011139 -2.651968 0.429896

N -4.458608 -2.171975 -0.725776

N -3.904258 -0.989452 -0.921817

O 1.305518 0.835740 -1.375085

N 6.175988 -1.359750 -0.531833

H 6.145051 -0.725638 -1.323844

H 5.656930 -2.183440 -0.819440

C 5.529565 -0.741834 0.619846

H 5.505660 -1.449140 1.452205

H 6.138814 0.101362 0.963980

C 4.106114 -0.199269 0.462698

O 3.433731 0.126832 1.418789

O 3.719619 -0.105758 -0.805900

H 2.791041 0.259138 -0.884918

O -3.064446 2.311305 1.181263

B3LYP/6-311++G**

Total electronic energy = -1536.592093 Ha

Zero-point correction = 0.219613 Ha

Thermal correction to enthalpy = 0.247015 Ha

Thermal correction to free energy = 0.144287 Ha

C -5.488103 -0.280636 -1.661946

C -4.111505 -0.354990 -1.624824

C -3.465465 -0.625791 -0.405589

C -4.212487 -0.815565 0.767754

C -5.592787 -0.742193 0.733804

C -6.232613 -0.474640 -0.482458

H -6.003107 -0.071642 -2.594819

H -3.514230 -0.206875 -2.515649

H -3.682530 -1.015758 1.691413

H -6.193335 -0.883915 1.623208

N -2.069152 -0.717805 -0.256723

N -1.930177 3.090194 0.050495

N -1.311498 3.966072 0.273838

N -0.613188 -1.098692 4.069280

N -1.646943 -1.112699 3.707152

O -1.410146 -0.557176 -1.281855

C 6.056056 0.565798 0.203933

C 5.418305 -0.560691 -0.307639

C 4.043574 -0.590239 -0.504543

C 3.287702 0.541029 -0.179679

C 3.914471 1.679517 0.334769

C 5.292109 1.687585 0.523945

H 7.129100 0.562955 0.347807

H 3.560765 -1.471400 -0.902704

H 3.307879 2.542530 0.577969

H 5.781171 2.568766 0.922803

Cl 6.376355 -1.980945 -0.714731

C 1.803113 0.569378 -0.370475

O 1.116219 1.532846 -0.106175

O 1.318533 -0.577304 -0.862653

H 0.339490 -0.508191 -0.975230

O -7.584088 -0.411256 -0.467612

H -7.924897 -0.218130 -1.348612

B3LYP/6-311++G**

Total electronic energy = -1536.450090 Ha

Zero-point correction = 0.229495 Ha

Thermal correction to enthalpy = 0.250504 Ha

Thermal correction to free energy = 0.175155 Ha

C -5.401595 -1.168077 -0.297350

C -4.810855 0.071190 -0.110389

C -3.430401 0.147490 0.076599

C -2.627942 -0.989937 0.077648

C -3.225256 -2.227562 -0.122250

C -4.608092 -2.319875 -0.309092

H -6.470202 -1.261821 -0.442112

H -5.410786 0.972534 -0.102734

H -1.561113 -0.902221 0.244120

H -2.613976 -3.124248 -0.122698

N -2.837389 1.427273 0.291876

N -1.851541 1.981675 -0.470754

N -1.604861 3.182805 0.015131

N -2.443138 3.358556 1.035195

N -3.190857 2.315996 1.217985

C 5.433109 -1.065422 1.073271

C 5.196141 -0.407185 -0.130578

C 3.918317 -0.003204 -0.495589

C 2.851119 -0.268743 0.369345

C 3.074328 -0.928954 1.581321

C 4.361862 -1.323132 1.927696

H 6.438820 -1.368743 1.335303

H 3.747514 0.509492 -1.431639

H 2.233201 -1.119497 2.235350

H 4.540466 -1.833166 2.867055

Cl 6.543255 -0.080115 -1.212120

C 1.457459 0.140648 0.027187

O 0.498926 -0.081803 0.744209

O 1.363882 0.774412 -1.145204

H 0.424989 1.027896 -1.299966

O -1.317652 1.423902 -1.445662

O -5.243848 -3.505867 -0.504615

H -4.609370 -4.230987 -0.499403

B3LYP/6-311++G**

Total electronic energy = -1536.441475 Ha

Zero-point correction = 0.228450 Ha

Thermal correction to enthalpy = 0.249709 Ha

Thermal correction to free energy = 0.172667 Ha

C -2.278028 -1.589017 1.656592

C -2.206546 -0.292113 1.313568

C -2.761137 0.131638 0.014822

C -3.507755 -0.872318 -0.803104

C -3.627078 -2.231702 -0.288895

C -2.983735 -2.575474 0.850328

H -1.837673 -1.914203 2.594841

H -1.747175 0.449758 1.950230

H -4.255828 -0.505888 -1.495678

N -3.125140 1.534685 -0.073681

N -3.894821 2.041365 -1.029685

N -3.955033 3.307806 -0.779795

N -3.229001 3.574039 0.329267

N -2.709728 2.474852 0.768191

C 5.475381 -0.521260 -0.637082

C 4.709827 -1.288995 -1.512414

C 3.320235 -1.211173 -1.491558

C 2.688550 -0.354646 -0.584470

C 3.447607 0.422008 0.297091

C 4.831234 0.327915 0.260084

H 6.556487 -0.577056 -0.648670

H 2.723487 -1.804526 -2.170896

H 2.947226 1.084322 0.990876

C 1.202767 -0.229861 -0.515975

O 0.621636 0.505006 0.255327

O 0.565960 -1.019665 -1.395421

O -2.165593 -0.367634 -1.170842

H -0.398344 -0.865256 -1.308884

Cl 5.792390 1.298970 1.366499

H 5.205480 -1.950027 -2.213741

H -4.247201 -2.948053 -0.811260

O -3.061286 -3.856840 1.299049

H -2.574694 -3.957746 2.123792

B3LYP/6-311++G**

Total electronic energy = -1536.421408 Ha

Zero-point correction = 0.228658 Ha

Thermal correction to enthalpy = 0.250151 Ha

Thermal correction to free energy = 0.170642 Ha

C 2.685390 1.011073 -1.742324

C 3.943699 1.053095 -1.162460

C 4.239060 0.194189 -0.102645

C 3.288314 -0.703582 0.377960

C 2.028931 -0.740681 -0.206835

C 1.718894 0.115766 -1.270013

H 2.435671 1.665307 -2.568173

H 4.694399 1.742344 -1.524814

H 3.532505 -1.363161 1.199720

H 1.283292 -1.435278 0.162579

N 5.533681 0.235778 0.497617

N 5.930028 -0.564546 1.481870

N 7.151472 -0.209423 1.739694

N 7.499281 0.797427 0.920767

N 6.497392 1.080341 0.145515

C -4.695569 2.205831 0.126034

C -5.277158 1.007551 0.536857

C -4.610498 -0.201338 0.396439

C -3.327044 -0.205329 -0.168223

C -2.730069 0.990553 -0.584673

C -3.420724 2.188070 -0.434875

H -5.236128 3.136234 0.245199

H -5.076770 -1.121087 0.720760

H -1.740421 0.983055 -1.022290

H -2.965209 3.116715 -0.756646

Cl -6.880723 1.026134 1.243984

C -2.572985 -1.466142 -0.334598

O -1.434656 -1.590507 -0.760938

O -3.282413 -2.547136 0.041823

O -2.547193 -3.768084 -0.115896

H -1.709820 -3.441597 -0.513708

O 0.506427 0.123754 -1.875729

H -0.086978 -0.542397 -1.486537

B3LYP/6-311++G**

Total electronic energy = -939.900410 Ha

Zero-point correction = 0.189089 Ha

Thermal correction to enthalpy = 0.206686 Ha

Thermal correction to free energy = 0.140656 Ha

C 0.347614 -0.034593 0.138126

C -0.863432 -0.595719 0.304221

C -2.062032 0.260763 0.344956

C -1.911913 1.706674 0.038421

C -0.579311 2.213569 -0.231184

C 0.517603 1.403159 -0.118325

H 1.242219 -0.659402 0.128398

H -0.980920 -1.667537 0.406053

H -2.763629 2.232173 -0.377763

H -0.458735 3.244790 -0.538290

N -3.310206 -0.416194 -0.022487

N -3.695020 -1.572447 0.497134

N -4.862000 -1.808321 -0.017055

N -5.188751 -0.794675 -0.842905

N -4.224256 0.072125 -0.848517

O 1.734041 1.886471 -0.314068

N 6.484686 -0.259525 0.018218

H 6.487350 0.060328 0.983307

H 6.173535 0.554711 -0.508213

C 5.438113 -1.279882 -0.126661

H 5.564893 -1.784014 -1.092367

H 5.567776 -2.052070 0.635887

C 3.970684 -0.791216 -0.076205

O 3.085592 -1.656795 0.049371

O 3.805986 0.466514 -0.201798

H 2.513134 1.201364 -0.246632

O -2.240714 1.182108 1.401345

B3LYP/6-311++G**

Total electronic energy = -1611.590738 Ha

Zero-point correction = 0.232063 Ha

Thermal correction to enthalpy = 0.254342 Ha

Thermal correction to free energy = 0.173879 Ha

C -3.171609 -0.146086 0.892019

C -2.120458 0.419729 1.501353

C -0.737489 0.198523 1.018244

C -0.571380 -0.522338 -0.266567

C -1.625924 -1.084668 -0.873411

C -2.986342 -0.943428 -0.332313

H -4.187886 -0.042846 1.256261

H -2.217860 1.018033 2.399529

H 0.442980 -0.621518 -0.635568

H -1.533006 -1.648266 -1.794510

N -1.073494 2.276714 -0.388768

N -2.005975 2.409490 -1.310797

N -1.897369 3.628478 -1.807521

N -0.893804 4.239303 -1.184181

N -0.381953 3.399501 -0.301893

C 7.704970 -0.070383 0.445351

C 7.001329 -0.921330 -0.402673

C 5.617087 -1.000809 -0.366709

C 4.913862 -0.211277 0.547807

C 5.603970 0.647779 1.407360

C 6.993584 0.715196 1.350457

H 8.785499 -0.024097 0.394698

H 5.069133 -1.656379 -1.030459

H 5.045766 1.257243 2.105120

H 7.531451 1.384782 2.012083

Cl 7.896429 -1.919218 -1.551306

C 3.414474 -0.314455 0.562120

O 2.861101 0.422219 1.514755

O 2.802301 -1.010296 -0.224933

O 0.220123 0.430737 1.758103

H 1.861888 0.368762 1.494868

O -3.940181 -1.491411 -0.899229

N -9.355564 -1.298630 -0.815279

H -8.979451 -2.098424 -1.314444

H -9.167114 -0.493394 -1.404147

C -8.672777 -1.150139 0.464288

H -9.029522 -0.250180 0.970387

H -8.946759 -1.987117 1.116516

C -7.143057 -1.086165 0.478290

O -6.522275 -0.726626 1.457413

O -6.593877 -1.487631 -0.663882

H -5.596248 -1.440718 -0.630562

B3LYP/6-311++G**

Total electronic energy = -1611.584724 Ha

Zero-point correction = 0.229844 Ha

Thermal correction to enthalpy = 0.253024 Ha

Thermal correction to free energy = 0.172720 Ha

C -2.219653 1.565405 -0.656087

C -3.486658 1.208545 -0.389900

C -3.838721 -0.221482 -0.325925

C -2.746591 -1.237693 -0.383232

C -1.371878 -0.755438 -0.548693

C -1.172044 0.560803 -0.755847

H -1.915683 2.605097 -0.700373

H -4.255825 1.945528 -0.201023

H -2.901411 -2.190406 0.109595

H -0.555149 -1.459033 -0.493788

N -5.039976 -0.525408 0.431214

N -5.180074 -1.562840 1.248844

N -6.404888 -1.507966 1.658212

N -7.011763 -0.439721 1.094507

N -6.167236 0.169128 0.328061

O -0.103235 3.895381 0.203991

O -3.681696 -1.019067 -1.482808

O 1.110849 0.223608 -0.938817

C 2.182281 0.795874 -0.288949

O 2.149488 1.878803 0.230367

C 3.351311 -0.122825 -0.327076

C 3.391883 -1.270180 -1.128277

C 4.443059 0.218408 0.479523

C 4.527494 -2.073538 -1.114413

H 2.553749 -1.523634 -1.763043

C 5.562778 -0.601003 0.479868

H 4.403411 1.111451 1.088538

C 5.617524 -1.747553 -0.310428

H 4.569548 -2.960220 -1.735545

H 6.502270 -2.371265 -0.296645

Cl 6.933244 -0.184726 1.490931

H 0.208415 4.650359 0.710650

O 0.034043 1.173651 -1.008989

H 0.632786 3.265179 0.196587

B3LYP/6-311++G**

Total electronic energy = -1536.414847 Ha

Zero-point correction = 0.226613 Ha

Thermal correction to enthalpy = 0.248808 Ha

Thermal correction to free energy = 0.169770 Ha

C -2.194299 1.570230 -0.296332

C -3.461187 1.160496 0.101112

C -3.821640 -0.179024 -0.038265

C -2.941652 -1.118056 -0.575170

C -1.683566 -0.706705 -0.996055

C -1.326730 0.632287 -0.852867

H -1.859761 2.594026 -0.171104

H -4.154826 1.867701 0.535155

H -3.244527 -2.151643 -0.671817

H -0.991467 -1.416343 -1.427221

N -5.117277 -0.603472 0.385480

N -5.511555 -1.872779 0.436754

N -6.733140 -1.833402 0.865641

N -7.084670 -0.550400 1.078354

N -6.085452 0.218182 0.780144

O -0.102200 3.982569 0.062317

O 0.934845 0.181088 -1.136183

C 1.985404 0.759793 -0.469533

O 1.970912 1.874127 -0.017669

C 3.128380 -0.192453 -0.386767

C 3.159262 -1.408696 -1.078791

C 4.203837 0.188639 0.423649

C 4.267703 -2.239940 -0.953662

H 2.332718 -1.695995 -1.713965

C 5.297006 -0.658605 0.535819

H 4.172168 1.133631 0.948873

C 5.341567 -1.873324 -0.145447

H 4.300899 -3.180842 -1.489775

H 6.205340 -2.517996 -0.044475

Cl 6.647352 -0.191019 1.552557

H 0.278347 4.675799 0.608681

O -0.133481 1.150785 -1.329168

H 0.595648 3.315676 -0.032958

B3LYP/6-311++G**

Total electronic energy = -1535.115959 Ha

Zero-point correction = 0.205962 Ha

Thermal correction to enthalpy = 0.225462 Ha

Thermal correction to free energy = 0.154181 Ha

C -2.257237 1.668678 1.138291

C -3.478371 1.144396 0.947633

C -3.709008 0.262203 -0.211207

C -2.542718 -0.152371 -1.044812

C -1.220619 0.368477 -0.683026

C -1.130550 1.279221 0.305406

H -2.061016 2.342482 1.963383

H -4.298701 1.347918 1.622918

H -2.594778 -1.100366 -1.567439

H -0.350384 0.006439 -1.209384

N -4.841216 -0.636814 -0.083222

N -4.868819 -1.890122 -0.522201

N -6.068808 -2.306063 -0.282558

N -6.772869 -1.311387 0.302732

N -6.014021 -0.271848 0.422654

O -3.550003 0.796687 -1.510386

O 1.154849 1.493400 -0.021039

C 1.999598 0.713060 0.765841

O 1.713574 0.319114 1.856568

C 3.265038 0.451982 0.024976

C 3.601571 1.103262 -1.167632

C 4.134296 -0.489294 0.588744

C 4.809220 0.806079 -1.791763

H 2.933364 1.838128 -1.595357

C 5.331156 -0.771111 -0.053957

H 3.866127 -0.980900 1.514353

C 5.680005 -0.132283 -1.242428

H 5.079488 1.309113 -2.712523

H 6.620024 -0.366419 -1.725685

Cl 6.425358 -1.951732 0.642733

O 0.011661 1.913661 0.732987

B3LYP/6-311++G**

Total electronic energy = -1459.946104 Ha

Zero-point correction = 0.202573 Ha

Thermal correction to enthalpy = 0.221301 Ha

Thermal correction to free energy = 0.150701 Ha

C -2.405358 1.973194 0.491448

C -3.633777 1.333596 0.511263

C -3.741773 0.043275 -0.011465

C -2.637264 -0.607855 -0.550831

C -1.402463 0.033810 -0.574650

C -1.297141 1.318493 -0.052355

H -2.292454 2.971521 0.894310

H -4.501561 1.824688 0.929819

H -2.739642 -1.607179 -0.951363

H -0.541789 -0.461754 -0.998043

N -5.006854 -0.617132 0.007705

N -5.226268 -1.832822 -0.483694

N -6.484607 -2.063146 -0.274216

N -7.031546 -0.997717 0.339587

N -6.118036 -0.095332 0.517919

O 0.967835 1.349987 -0.580217

C 1.863366 0.982501 0.421689

O 1.635473 1.093502 1.588799

C 3.098708 0.427206 -0.198505

C 3.376092 0.522825 -1.567312

C 4.002773 -0.201149 0.665888

C 4.559043 -0.015100 -2.064827

H 2.681627 1.019795 -2.230779

C 5.174108 -0.732544 0.145713

H 3.781138 -0.262068 1.723023

C 5.463763 -0.647081 -1.214788

H 4.783556 0.059010 -3.122179

H 6.384874 -1.067102 -1.598321

Cl 6.311625 -1.525217 1.220251

O -0.141140 2.069987 -0.027263

B3LYP/6-311++G**

Total electronic energy = -1535.107403 Ha

Zero-point correction = 0.201021 Ha

Thermal correction to enthalpy = 0.222234 Ha

Thermal correction to free energy = 0.144010 Ha

C 2.935079 -2.272355 0.024625

C 4.073108 -1.573166 -0.056075

C 4.030250 -0.112333 -0.320039

C 2.727640 0.601753 -0.320300

C 1.527953 -0.195115 -0.089698

C 1.596787 -1.633972 -0.030060

H 2.941284 -3.345260 0.175044

H 5.044865 -2.042932 0.034047

H 2.709984 1.657356 -0.078290

H 0.558699 0.270335 0.065915

N 5.212209 0.616752 0.095344

N 5.209670 1.845106 0.603871

N 6.454758 2.147566 0.761622

N 7.218764 1.108285 0.351573

N 6.449998 0.159084 -0.067024

O 3.485496 0.313116 -1.549351

O -1.832460 -1.642662 -0.404380

C -2.249491 -0.506650 0.081043

O -1.529968 0.422268 0.410991

C -3.751796 -0.471125 0.137205

C -4.502870 -1.624435 0.393242

C -4.379420 0.761429 -0.071749

C -5.890905 -1.535399 0.456365

H -4.001924 -2.570104 0.554014

C -5.763409 0.823056 -0.008111

H -3.785550 1.645935 -0.259730

C -6.529199 -0.314817 0.253620

H -6.482332 -2.418700 0.666093

H -7.608084 -0.238588 0.299154

Cl -6.570955 2.360653 -0.255371

O 0.573075 -2.331213 0.038608

B3LYP/6-311++G**

Total electronic energy = -655.908535 Ha

Zero-point correction = 0.118438 Ha

Thermal correction to enthalpy = 0.132407 Ha

Thermal correction to free energy = 0.072923 Ha

C 3.348321 0.442432 -0.000370

C 2.349987 1.410134 -0.000131

C 1.006737 1.032408 0.000072

C 0.670419 -0.326399 0.000040

C 1.669089 -1.292106 -0.000183

C 3.011895 -0.912613 -0.000381

H 4.390894 0.745777 -0.000544

H 2.602825 2.463191 -0.000122

H -0.367851 -0.639402 0.000260

H 1.419113 -2.345895 -0.000184

N 0.052704 2.088231 0.000164

N -3.125824 -1.555670 0.004618

N -4.172377 -1.141315 0.000374

N -2.271171 1.678650 0.000793

N -1.143037 1.806961 0.000470

O -5.273678 -0.708718 -0.004091

O 3.948798 -1.910580 -0.000667

H 4.833310 -1.530426 -0.000567

B3LYP/6-311++G**

Total electronic energy = - 655.874905 Ha

Zero-point correction = 0.122713 Ha

Thermal correction to enthalpy = 0.133579 Ha

Thermal correction to free energy = 0.085132 Ha

C 2.487564 1.152080 -0.046033

C 1.105877 1.251707 -0.044629

C 0.335516 0.087938 0.003290

C 0.933196 -1.168975 0.046093

C 2.320225 -1.260655 0.040797

C 3.101457 -0.103847 -0.004087

H 3.106506 2.039487 -0.083009

H 0.624912 2.219878 -0.079736

H 0.323266 -2.061335 0.082705

H 2.791229 -2.237675 0.073875

N -1.086790 0.188921 0.007711

N -1.890690 -0.873005 -0.055612

N -3.098685 -0.317502 -0.018535

N -3.002665 1.066899 0.061604

N -1.742952 1.335042 0.078391

O -4.159692 -0.928246 -0.052406

O 4.462609 -0.130820 -0.010255

H 4.780215 -1.039801 0.019948

B3LYP/6-311++G**

Total electronic energy = -655.866367 Ha

Zero-point correction = 0.122589 Ha

Thermal correction to enthalpy = 0.133400 Ha

Thermal correction to free energy = 0.086103 Ha

C 2.253726 -1.208036 0.337219

C 0.870802 -1.272200 0.306300

C 0.136484 -0.143046 -0.067824

C 0.774355 1.049330 -0.406202

C 2.162002 1.108908 -0.360965

C 2.904922 -0.015414 0.006601

H 2.843690 -2.069620 0.622728

H 0.356472 -2.189412 0.561026

H 0.200206 1.920842 -0.684492

H 2.664776 2.035853 -0.616837

N -1.281095 -0.252515 -0.125575

N -2.186398 0.725975 0.206314

N -3.393667 0.182723 0.105545

N -3.213908 -1.085880 -0.251544

N -1.959660 -1.370552 -0.399398

O 4.264042 -0.014257 0.065225

H 4.610009 0.851084 -0.179188

O -1.894765 1.881224 0.517099

B3LYP/6-311++G**

Total electronic energy = -655.925570 Ha

Zero-point correction = 0.115232 Ha

Thermal correction to enthalpy = 0.130332 Ha

Thermal correction to free energy = 0.064825 Ha

C 2.332987 1.466785 0.000018

C 0.986410 1.147047 0.000025

C 0.568179 -0.192634 0.000000

C 1.527652 -1.221257 -0.000027

C 2.873238 -0.906131 -0.000031

C 3.280769 0.438452 -0.000013

H 2.671899 2.494965 0.000031

H 0.229607 1.922044 0.000049

H 1.199878 -2.252855 -0.000038

H 3.618561 -1.695840 -0.000048

N -0.820193 -0.396767 0.000009

N -1.210874 -1.577441 0.000009

N -2.719618 -1.518310 0.000035

N -4.313553 2.432342 -0.547806

N -4.313051 2.433083 0.547714

O -3.169640 -2.610103 0.000083

O 4.588677 0.798853 -0.000007

H 5.153378 0.017756 -0.000156

B3LYP/6-311++G**

Total electronic energy = -580.695587 Ha

Total electronic energy solvent (water) = -580.709011 Ha

Total electronic energy solvent (methanol) = -580.708612 Ha

Total electronic energy solvent (acetonitrile) = -580.708670 Ha

Total electronic energy RI-B2KPLYP/ma-def2-TZVP = -580.225631 Ha

Zero-point correction = 0.118696 Ha

Thermal correction to enthalpy = 0.128663 Ha

Thermal correction to free energy = 0.082609 Ha

C 2.014226 1.220639 0.000081

C 0.628024 1.218950 0.000081

C -0.056595 0.002523 0.000015

C 0.631878 -1.208017 -0.000057

C 2.021369 -1.199295 -0.000052

C 2.717005 0.012482 0.000014

H 2.567003 2.151442 0.000124

H 0.076939 2.149565 0.000136

H 0.087390 -2.142447 -0.000119

H 2.562314 -2.140042 -0.000121

N -1.483963 -0.001467 -0.000021

N -2.230925 -1.100935 0.000139

N -3.457736 -0.679733 0.000092

N -2.237342 1.093488 -0.000160

N -3.461753 0.664949 -0.000086

O 4.076831 0.083138 -0.000017

H 4.458296 -0.801425 -0.000114

B3LYP/6-311++G**

Total electronic energy = -2415.694940 Ha

Zero-point correction = 0.312517 Ha

Thermal correction to enthalpy = 0.342473 Ha

Thermal correction to free energy = 0.242316 Ha

C 0.360626 1.704847 1.777708

C 1.008667 2.836753 1.465549

C 0.895207 3.373953 0.096513

C -0.047944 2.725303 -0.854481

C -0.805896 1.562164 -0.391233

C -0.547876 1.068245 0.836083

H 0.455779 1.257546 2.759037

H 1.620440 3.364494 2.184584

H -0.462246 3.324933 -1.655637

H -1.556218 1.132683 -1.037295

N 1.169656 4.789659 -0.036417

N 0.529060 5.612182 -0.860539

N 1.116470 6.753958 -0.722211

N 2.113767 6.631803 0.184355

N 2.151335 5.412218 0.607972

C 6.086653 -3.652877 -0.755858

C 5.077208 -3.758297 0.198566

C 4.114131 -2.770158 0.341889

C 4.161285 -1.647351 -0.490870

C 5.167861 -1.527128 -1.454101

C 6.124497 -2.530337 -1.580413

H 6.827961 -4.436411 -0.848659

H 3.330503 -2.851191 1.083319

H 5.195941 -0.654457 -2.092303

H 6.907911 -2.442102 -2.324036

Cl 5.024529 -5.172876 1.241956

C 3.110682 -0.602018 -0.307136

O 3.243082 0.426697 -1.160551

O 2.229485 -0.668783 0.522926

O 1.417886 2.602469 -0.982847

O -2.057131 -0.656682 0.522313

C -3.364973 -0.462225 0.960018

O -3.666234 0.288421 1.839137

C -4.285841 -1.308116 0.151097

C -3.838006 -2.329679 -0.694804

C -5.654385 -1.046882 0.286149

C -4.766455 -3.085321 -1.404353

H -2.780615 -2.537149 -0.786563

C -6.560225 -1.811281 -0.434864

H -5.988333 -0.260366 0.949638

C -6.130618 -2.831340 -1.281779

H -4.429106 -3.882265 -2.056231

H -6.855481 -3.417525 -1.832227

Cl -8.277342 -1.489175 -0.276260

O -1.129381 -0.026901 1.413435

H 2.540012 1.086640 -0.989968

B3LYP/6-311++G**

Total electronic energy = -2415.665536 Ha

Zero-point correction = 0.311711 Ha

Thermal correction to enthalpy = 0.342335 Ha

Thermal correction to free energy = 0.238710 Ha

C 1.849120 1.529990 -0.398151

C 0.480437 1.514978 -0.649638

C -0.378955 2.231772 0.175086

C 0.105361 2.961966 1.261318

C 1.466473 2.973306 1.519041

C 2.329492 2.257969 0.685093

H 2.524104 0.981109 -1.037486

H 0.084380 0.945592 -1.479193

H -0.577728 3.511133 1.894930

H 1.869320 3.529779 2.355572

N -1.779099 2.224346 -0.094503

N -2.708403 2.635588 0.761213

N -3.836232 2.469398 0.144465

N -3.598051 1.956359 -1.076130

N -2.320109 1.805163 -1.232151

C -8.021316 -1.926028 -0.339228

C -7.106337 -1.066139 -0.942991

C -5.804827 -0.945599 -0.472342

C -5.419364 -1.715288 0.632974

C -6.327851 -2.580080 1.253041

C -7.624137 -2.681012 0.762640

H -9.029385 -1.998726 -0.727279

H -5.116143 -0.259094 -0.946722

H -6.004923 -3.159189 2.108415

H -8.333287 -3.349140 1.236641

Cl -7.611115 -0.114960 -2.327697

C -4.049846 -1.654176 1.194330

O -3.654000 -2.214533 2.193943

O -3.216295 -0.882306 0.448143

O -1.899361 -0.833401 1.021219

H -2.017102 -1.411032 1.810853

O 4.503623 1.649588 0.130529

C 5.073323 0.542502 0.752132

O 4.758368 0.152145 1.836175

C 6.095873 -0.059436 -0.149735

C 6.544153 0.563256 -1.320547

C 6.613253 -1.301706 0.235167

C 7.509120 -0.063823 -2.102846

H 6.150333 1.528238 -1.608844

C 7.572569 -1.908493 -0.562696

H 6.263820 -1.769934 1.145646

C 8.028114 -1.302206 -1.731792

H 7.864674 0.413642 -3.008123

H 8.778735 -1.793274 -2.337924

Cl 8.224307 -3.467536 -0.090790

O 3.658200 2.361861 1.042793

B3LYP/6-311++G**

Total electronic energy = -580.748960 Ha

Zero-point correction = 0.112755 Ha

Thermal correction to enthalpy = 0.126477 Ha

Thermal correction to free energy = 0.066230 Ha

C -2.449366 -1.382012 0.000005

C -1.063674 -1.328826 0.000004

C -0.401960 -0.096385 -0.000002

C -1.147570 1.084380 -0.000008

C -2.539530 1.030297 -0.000007

C -3.194886 -0.200835 -0.000001

H -2.969284 -2.332061 0.000010

H -0.477279 -2.239407 0.000009

H -0.654015 2.049834 -0.000013

H -3.111349 1.953352 -0.000012

N 1.019667 -0.141597 -0.000003

N 1.643273 0.918324 0.000006

N 2.328444 1.822962 0.000014

N 5.066136 -0.644377 -0.000005

N 6.146656 -0.825244 -0.000004

O -4.558975 -0.317278 0.000000

H -4.963592 0.556318 0.000001

B3LYP/6-311++G**

Imaginary frequency = 596.5i cm^-1^

Total electronic energy = -1611.477483 Ha

Zero-point correction = 0.228212 Ha

Thermal correction to enthalpy = 0.249614 Ha

Thermal correction to free energy = 0.174769 Ha

C -2.592843 -1.723457 -1.054210

C -3.134848 -0.499928 -0.975385

C -2.991607 0.278543 0.274234

C -2.148240 -0.260418 1.371553

C -1.506244 -1.567111 1.174937

C -1.762074 -2.214850 0.021410

H -2.688176 -2.327151 -1.947099

H -3.667482 -0.060269 -1.807885

H -1.674978 0.440205 2.049839

H -0.788112 -1.923456 1.901405

N -3.104482 1.716931 0.108447

N -2.418156 2.614671 0.805412

N -2.869391 3.762028 0.417065

N -3.828316 3.567093 -0.515304

N -3.979927 2.297180 -0.704399

O -1.916778 -3.901445 0.135516

O -3.612772 -0.207914 1.451412

O 0.941987 -2.350546 -0.301068

C 1.303820 -1.119312 -0.793250

O 0.656192 -0.488741 -1.581789

C 2.605905 -0.688566 -0.198369

C 3.394246 -1.521169 0.604118

C 3.032397 0.610233 -0.495510

C 4.602418 -1.048754 1.107964

H 3.068176 -2.528518 0.823619

C 4.239789 1.061078 0.019350

H 2.419186 1.243253 -1.122837

C 5.033992 0.244594 0.821127

H 5.218730 -1.690694 1.726468

H 5.973337 0.616361 1.210240

Cl 4.777792 2.691658 -0.348345

H -1.994868 -4.118900 1.077085

O -0.217358 -2.853324 -0.932209

H -0.956820 -4.003870 -0.210219

B3LYP/6-311++G**

Imaginary frequency = 414.7i cm^-1^

Total electronic energy = -1611.494656 Ha

Zero-point correction = 0.229287 Ha

Thermal correction to enthalpy = 0.249968 Ha

Thermal correction to free energy = 0.177748 Ha

C -2.370728 1.907093 0.840746

C -3.524547 1.260214 0.638787

C -3.609745 0.239902 -0.432943

C -2.390488 -0.094045 -1.199123

C -1.154979 0.624653 -0.898946

C -1.158262 1.549775 0.105505

H -2.280314 2.669344 1.604958

H -4.402251 1.463711 1.238159

H -2.319397 -1.081362 -1.639780

H -0.250676 0.357381 -1.430030

N -4.612239 -0.789986 -0.196713

N -4.468432 -2.072740 -0.505283

N -5.601561 -2.622086 -0.209391

N -6.435206 -1.678368 0.279505

N -5.823858 -0.538318 0.283799

O -0.288798 2.860976 -0.203278

O -3.535879 0.664938 -1.777985

O 0.910141 0.322888 1.302334

C 1.952784 0.782196 0.621764

O 1.985832 1.896139 0.090291

C 3.074142 -0.181967 0.551125

C 3.036162 -1.407621 1.227761

C 4.190165 0.171602 -0.216518

C 4.117315 -2.275983 1.128659

H 2.171819 -1.670695 1.822164

C 5.257036 -0.711827 -0.299452

H 4.212397 1.122370 -0.731347

C 5.232956 -1.936131 0.365314

H 4.096712 -3.226085 1.648772

H 6.076483 -2.610107 0.286116

Cl 6.659988 -0.278924 -1.259227

H -0.421755 3.042102 -1.147923

O -0.088390 1.310741 1.541131

H 0.712145 2.572928 -0.023666

B3LYP/6-311++G**

Imaginary frequency = 398.1i cm^-1^

Total electronic energy = -1536.323508 Ha

Zero-point correction = 0.225340 Ha

Thermal correction to enthalpy = 0.245261 Ha

Thermal correction to free energy = 0.173756 Ha

C -2.508860 1.972092 0.558167

C -3.687792 1.254089 0.506684

C -3.738096 0.030154 -0.168815

C -2.607447 -0.460747 -0.821878

C -1.416597 0.246739 -0.780028

C -1.334965 1.428940 -0.011281

H -2.455775 2.924468 1.068696

H -4.580372 1.648408 0.974153

H -2.664321 -1.388244 -1.376224

H -0.539924 -0.137097 -1.287737

N -4.960326 -0.698035 -0.226208

N -6.093233 -0.307983 0.347583

N -6.957263 -1.239430 0.064241

N -6.359453 -2.184482 -0.671788

N -5.113764 -1.856503 -0.858639

O -0.441698 2.566420 -0.678912

O 0.881968 0.406859 1.201409

C 1.890894 0.794235 0.439410

O 1.861689 1.795403 -0.286961

C 3.065386 -0.103424 0.520029

C 3.116077 -1.169505 1.427025

C 4.141695 0.151663 -0.338368

C 4.246076 -1.978334 1.466800

H 2.282427 -1.354606 2.090698

C 5.258319 -0.669805 -0.279484

H 4.096270 0.980702 -1.031218

C 5.322850 -1.735856 0.615568

H 4.294780 -2.804536 2.165817

H 6.204520 -2.363455 0.644910

Cl 6.611713 -0.359930 -1.351199

H -0.658732 2.599238 -1.623046

O -0.184625 1.372375 1.322069

H 0.586366 2.329047 -0.505791

B3LYP/6-311++G**

Imaginary frequency = 614.3i cm^-1^

Total electronic energy = -1536.304515 Ha

Zero-point correction = 0.224572 Ha

Thermal correction to enthalpy = 0.245187 Ha

Thermal correction to free energy = 0.171251 Ha

C -2.612165 -1.917701 -0.965318

C -3.262075 -0.695968 -0.919279

C -3.025916 0.179215 0.146296

C -2.143532 -0.156252 1.168270

C -1.475046 -1.378140 1.130679

C -1.700275 -2.210146 0.042619

H -2.774417 -2.609035 -1.780716

H -3.953229 -0.419869 -1.704148

H -1.973890 0.528378 1.988454

H -0.759686 -1.637093 1.900535

N -3.708991 1.430124 0.191432

N -3.545811 2.338818 1.147548

N -4.339850 3.315008 0.828739

N -4.981565 3.006702 -0.310206

N -4.594584 1.834595 -0.712843

O -1.707407 -3.898442 0.421700

O 1.044880 -2.231683 -0.093362

C 1.427687 -1.081502 -0.744249

O 0.832487 -0.593100 -1.662547

C 2.676906 -0.535295 -0.130058

C 3.432915 -1.233943 0.817900

C 3.088260 0.729764 -0.563266

C 4.593579 -0.662149 1.330588

H 3.120239 -2.217279 1.141250

C 4.247180 1.281962 -0.036076

H 2.501273 1.257717 -1.302914

C 5.008758 0.599462 0.909732

H 5.185408 -1.201107 2.061153

H 5.910673 1.048892 1.305377

Cl 4.764938 2.872267 -0.572254

H -1.778463 -3.998385 1.382324

O -0.056912 -2.857048 -0.728449

H -0.728283 -3.931152 0.098546

B3LYP/6-311++G**

Imaginary frequency = 561.3i cm^-1^

Total electronic energy = -580.663357 Ha

Total electronic energy solvent (water) = -580.673959 Ha

Total electronic energy solvent (methanol) = -580.673637 Ha

Total electronic energy solvent (acetonitrile) = -580.673684 Ha

Total electronic energy RI-B2KPLYP/ma-def2-TZVP = -580.188387 Ha

Zero-point correction = 0.114701 Ha

Thermal correction to enthalpy = 0.125301 Ha

Thermal correction to free energy = 0.078346 Ha

C 2.070375 1.184865 -0.273045

C 0.689132 1.182612 -0.395487

C -0.033250 0.007661 -0.165479

C 0.635782 -1.168507 0.182576

C 2.022095 -1.171630 0.275898

C 2.742849 0.005376 0.056072

H 2.643616 2.087664 -0.442283

H 0.160788 2.088397 -0.665360

H 0.074098 -2.076225 0.364924

H 2.541525 -2.090024 0.531219

N -1.440453 0.064443 -0.253665

N -2.219439 -0.909629 -0.558618

N -3.402128 -0.880526 -0.277886

N -2.475194 1.117958 0.621517

N -3.528862 0.610789 0.551935

O 4.102629 0.065611 0.145871

H 4.459567 -0.798205 0.378326

B3LYP/6-311++G**

Imaginary frequency = 495.2i cm^-1^

Total electronic energy = -655.850186 Ha

Zero-point correction = 0.119169 Ha

Thermal correction to enthalpy = 0.130421 Ha

Thermal correction to free energy = 0.082264 Ha

C 2.552247 1.058251 -0.330866

C 1.191921 1.240923 -0.523970

C 0.302140 0.192364 -0.278348

C 0.778666 -1.046904 0.149144

C 2.145018 -1.238027 0.320398

C 3.033895 -0.185332 0.088225

H 3.255696 1.861059 -0.512277

H 0.807456 2.194853 -0.863290

H 0.088439 -1.861711 0.328041

H 2.517971 -2.206540 0.638746

N -1.087943 0.453370 -0.477569

N -1.899045 -0.573356 -0.678602

N -2.997908 -0.495023 -0.057717

N -2.848208 1.014320 0.906330

N -1.755529 1.375201 0.685527

O -4.029242 -1.106053 -0.160490

O 4.382423 -0.310816 0.245243

H 4.602106 -1.201935 0.537466

B3LYP/6-311++G**

Imaginary frequency = 460.0i cm^-1^

Total electronic energy = -1536.369258 Ha

Zero-point correction = 0.225903 Ha

Thermal correction to enthalpy = 0.246775 Ha

Thermal correction to free energy = 0.171568 Ha

C -3.276960 -1.918010 1.139467

C -3.091676 -0.560318 1.192611

C -2.919121 0.189792 -0.017844

C -3.150931 -0.473247 -1.271664

C -3.342851 -1.827203 -1.310600

C -3.399116 -2.561970 -0.108276

H -3.328341 -2.495637 2.055913

H -3.012407 -0.037795 2.136003

H -3.110706 0.117189 -2.176752

H -3.447651 -2.354353 -2.249779

N -3.219971 1.592242 0.039223

N -3.350285 2.369796 -1.028425

N -3.555598 3.556787 -0.557273

N -3.546318 3.504194 0.791745

N -3.335228 2.284333 1.166078

C 5.577472 -0.877533 0.073654

C 4.971784 0.371377 -0.035244

C 3.589884 0.509300 -0.045645

C 2.794753 -0.636112 0.055708

C 3.386174 -1.896950 0.165454

C 4.773013 -2.011498 0.174026

H 6.657126 -0.957897 0.079400

H 3.128285 1.483605 -0.130343

H 2.748269 -2.767689 0.242348

H 5.236975 -2.987579 0.258935

Cl 5.981995 1.807456 -0.162551

C 1.289621 -0.532065 0.048205

O 0.586070 -1.555643 0.144083

O 0.817502 0.655912 -0.061982

O -1.125271 0.333079 -0.035763

H -0.790168 -0.609299 0.039937

O -3.574867 -3.884517 -0.218552

H -3.563238 -4.312782 0.646874

B3LYP/6-311++G**

Imaginary frequency = 485.2i cm^-1^

Total electronic energy = -1536.368469 Ha

Zero-point correction = 0.226002 Ha

Thermal correction to enthalpy = 0.246554 Ha

Thermal correction to free energy = 0.171748 Ha

C 7.044131 -0.291195 -0.193827

C 5.785312 -0.784958 -0.491147

C 4.660052 -0.052328 -0.108433

C 4.775781 1.161583 0.565337

C 6.040923 1.646912 0.864184

C 7.178162 0.925104 0.485538

H 7.935176 -0.836885 -0.476286

H 5.672296 -1.726247 -1.012024

H 3.892212 1.717144 0.850222

H 6.141848 2.591554 1.387862

N 3.360866 -0.559283 -0.415475

N 2.244608 -0.102380 0.117166

N 1.330366 -0.841381 -0.428458

N 1.845303 -1.719989 -1.285962

N 3.128589 -1.556818 -1.276786

C -6.900887 -0.238932 0.808384

C -6.253054 0.657256 -0.037376

C -4.885024 0.584639 -0.262535

C -4.144728 -0.413480 0.377687

C -4.779111 -1.321252 1.229100

C -6.151778 -1.230305 1.440569

H -7.969022 -0.160731 0.966827

H -4.387104 1.282673 -0.921710

H -4.183854 -2.085616 1.711530

H -6.648234 -1.932784 2.100437

Cl -7.194764 1.913040 -0.840521

C -2.660456 -0.515638 0.152738

O -2.004662 -1.420030 0.729943

O -2.125813 0.336152 -0.627244

O -0.243075 -0.256634 -0.544984

H -0.720086 -0.937259 0.076284

O 8.439422 1.349121 0.746915

H 8.426707 2.192125 1.213960

B3LYP/6-311++G**

Imaginary frequency = 368.1i cm^-1^

Total electronic energy = -1820.434409 Ha

Total electronic energy solvent (water) = -1820.505357 Ha

Total electronic energy solvent (methanol) = -1820.503744 Ha

Total electronic energy solvent (acetonitrile) = -1820.503980 Ha

Total electronic energy RI-B2KPLYP/ma-def2-TZVP = -1819.143025 Ha

Zero-point correction = 0.293353 Ha

Thermal correction to enthalpy = 0.321481 Ha

Thermal correction to free energy = 0.226008 Ha

C 2.883078 0.786418 -0.288852

C 1.932930 1.687423 -0.686761

C 1.138058 2.369792 0.273000

C 1.396192 2.174045 1.655696

C 2.343662 1.271629 2.055895

C 3.143516 0.533184 1.108614

H 3.476864 0.245915 -1.017277

H 1.760842 1.882663 -1.737408

H 0.817640 2.734872 2.378311

H 2.522112 1.085082 3.108420

N 0.399215 3.518190 -0.132961

N -0.215478 4.345918 0.703032

N -0.797195 5.230803 -0.057989

N -0.539598 4.944966 -1.337927

N 0.206150 3.876601 -1.395829

C -5.894710 -3.026532 0.149460

C -5.833531 -1.709620 -0.296312

C -4.661820 -0.967580 -0.224109

C -3.516883 -1.564297 0.313042

C -3.560598 -2.884971 0.765712

C -4.745618 -3.609340 0.682117

H -6.821937 -3.581221 0.080093

H -4.626486 0.054636 -0.575071

H -2.658372 -3.319975 1.176380

H -4.781362 -4.635026 1.032057

Cl -7.285929 -0.966343 -0.971011

C -2.208748 -0.814839 0.421262

O -1.212230 -1.330514 0.910061

O -2.273046 0.400134 -0.052710

O -0.742221 1.169501 0.125049

H -0.319694 0.375540 0.517216

O 4.025692 -0.286664 1.508894

N 7.320301 -3.957105 -0.333481

H 6.528495 -4.514891 -0.027775

H 7.638850 -3.473513 0.500932

C 6.869668 -2.972096 -1.313655

H 7.736078 -2.413507 -1.684233

H 6.453930 -3.485700 -2.184279

C 5.835872 -1.922927 -0.880122

O 5.307884 -1.193273 -1.700301

O 5.625848 -1.903264 0.419492

H 4.938238 -1.191748 0.732100

B3LYP/6-311++G**

Imaginary frequency = 268.2i cm^-1^

Total electronic energy = -939.896653 Ha

Zero-point correction = 0.186858 Ha

Thermal correction to enthalpy = 0.204284 Ha

Thermal correction to free energy = 0.138102 Ha

C 0.240455 -0.190043 0.147061

C -0.991319 -0.662378 0.386590

C -2.137443 0.284532 0.413566

C -1.889308 1.666888 -0.013102

C -0.561108 2.104864 -0.274355

C 0.522039 1.236938 -0.164452

H 1.095991 -0.858866 0.138271

H -1.177620 -1.715187 0.563558

H -2.737776 2.241875 -0.355449

H -0.383501 3.139764 -0.539441

N -3.408690 -0.364407 -0.016841

N -3.823797 -1.531510 0.446859

N -4.999949 -1.712774 -0.074920

N -5.299105 -0.654409 -0.849328

N -4.309573 0.187041 -0.812970

O 1.738267 1.597433 -0.357662

N 6.612665 -0.112051 0.104121

H 6.461792 0.344332 0.999157

H 6.444190 0.614815 -0.585612

C 5.610808 -1.163082 -0.070115

H 5.811098 -1.695828 -1.006218

H 5.719553 -1.906635 0.723800

C 4.127079 -0.760129 -0.111058

O 3.260854 -1.623573 -0.087012

O 3.934962 0.529420 -0.190784

H 2.906100 0.865685 -0.246370

O -2.317570 1.144379 1.491087

B3LYP/6-311++G**

Imaginary frequency = 496.1i cm^-1^

Total electronic energy = -1536.362949 Ha

Zero-point correction = 0.226069 Ha

Thermal correction to enthalpy = 0.246478 Ha

Thermal correction to free energy = 0.173667 Ha

C -4.076532 2.438229 -0.806210

C -4.189972 1.059932 -0.821809

C -3.301473 0.293019 -0.062696

C -2.306175 0.884803 0.713281

C -2.196371 2.267429 0.717675

C -3.075884 3.047884 -0.039498

H -4.744613 3.058005 -1.390144

H -4.952086 0.576796 -1.418646

H -1.618004 0.294390 1.303157

H -1.422096 2.736476 1.315252

N -3.447642 -1.126012 -0.088067

N -2.483564 -2.021291 0.124684

N -3.012431 -3.203003 0.008712

N -4.302525 -3.038491 -0.248274

N -4.585930 -1.760097 -0.305112

C 5.410366 0.516236 0.854185

C 4.907982 0.176508 -0.399343

C 3.604119 -0.272600 -0.560919

C 2.780356 -0.380539 0.563612

C 3.269103 -0.044208 1.828547

C 4.580409 0.400807 1.968230

H 6.431737 0.861277 0.953603

H 3.220505 -0.539468 -1.536352

H 2.614176 -0.141677 2.684589

H 4.965663 0.659139 2.948017

Cl 5.952141 0.319983 -1.810022

C 1.364327 -0.860689 0.415043

O 0.608974 -0.898360 1.426564

O 0.978496 -1.213580 -0.742384

O -0.910968 -1.702604 -0.336989

H -0.506845 -1.479741 0.589769

O -3.013120 4.401489 -0.073018

H -2.278776 4.720919 0.463561

B3LYP/6-311++G**

Imaginary frequency = 523.9i cm^-1^

Total electronic energy = -1536.333057 Ha

Zero-point correction = 0.224861 Ha

Thermal correction to enthalpy = 0.245685 Ha

Thermal correction to free energy = 0.172327 Ha

C -3.515929 2.396643 -0.309498

C -2.767331 1.250002 -0.545754

C -3.258733 0.026574 -0.111384

C -4.478780 -0.074339 0.554870

C -5.216210 1.074270 0.802106

C -4.737036 2.312542 0.365767

H -1.808479 1.282731 -1.043551

H -4.846938 -1.036950 0.888366

H -6.161660 1.028314 1.327244

N -2.511968 -1.187316 -0.402803

N -2.441437 -2.190864 0.570667

N -2.509421 -3.289909 -0.006510

N -2.707394 -3.058158 -1.427209

N -2.742374 -1.836691 -1.634636

C 5.743809 0.218357 0.994387

C 5.140194 0.643521 -0.187538

C 3.794974 0.410182 -0.433955

C 3.037998 -0.265234 0.529430

C 3.627814 -0.698990 1.720862

C 4.978875 -0.453954 1.945469

H 6.795611 0.411374 1.163484

H 3.329126 0.738819 -1.353257

H 3.028905 -1.221253 2.454896

H 5.444498 -0.787026 2.865459

Cl 6.102166 1.491331 -1.388006

C 1.599104 -0.511330 0.260968

O 0.928062 -1.156475 1.183768

O 1.050074 -0.130049 -0.780500

O -0.796518 -0.762387 -0.577338

H 0.002095 -1.197933 0.765237

H -3.143076 3.359699 -0.643457

O -5.506118 3.403969 0.632806

H -5.085239 4.200208 0.290848

B3LYP/6-311++G**

Imaginary frequency = 492.5i cm^-1^

Total electronic energy = -655.830580 Ha

Zero-point correction = 0.118894 Ha

Thermal correction to enthalpy = 0.130357 Ha

Thermal correction to free energy = 0.081457 Ha

C 2.248415 1.018489 0.574977

C 0.887012 0.903372 0.824869

C 0.182856 -0.214507 0.366752

C 0.856612 -1.220573 -0.344895

C 2.218222 -1.117538 -0.569588

C 2.918659 0.007000 -0.116763

H 2.790420 1.890794 0.926208

H 0.358441 1.676440 1.367660

H 0.310027 -2.084816 -0.703337

H 2.756977 -1.892102 -1.101100

N -1.199705 -0.231648 0.588993

N -2.473146 0.877125 -0.404534

N -3.440276 0.185989 -0.466915

N -3.042253 -1.391083 0.189000

N -1.906593 -1.294577 0.570195

O -2.040266 1.979853 -0.643922

O 4.254366 0.053083 -0.381408

H 4.634493 0.868107 -0.036072

B3LYP/6-311++G**

Imaginary frequency = 465.1i cm^-1^

Total electronic energy = -2415.617948 Ha

Zero-point correction = 0.309296 Ha

Thermal correction to enthalpy = 0.339059 Ha

Thermal correction to free energy = 0.240620 Ha

C -0.469766 1.651136 2.094059

C 0.486666 2.482227 1.586397

C 0.689120 2.566378 0.164318

C -0.249818 1.911925 -0.696805

C -1.209295 1.070309 -0.180126

C -1.317282 0.943933 1.208954

H -0.598805 1.525002 3.160941

H 1.138713 3.051399 2.234827

H -0.145896 2.052199 -1.763701

H -1.861597 0.513558 -0.835039

N 1.304437 3.765132 -0.329737

N 1.260846 4.158110 -1.597257

N 1.977424 5.233527 -1.640757

N 2.458896 5.493742 -0.405984

N 2.044821 4.580770 0.410683

C 6.577909 -3.629490 0.285812

C 6.758484 -2.429705 -0.397144

C 5.770474 -1.453978 -0.423642

C 4.569525 -1.688514 0.252545

C 4.371830 -2.886922 0.942764

C 5.375982 -3.850308 0.956447

H 7.364086 -4.373778 0.291668

H 5.919985 -0.524809 -0.956375

H 3.433742 -3.043319 1.459093

H 5.227994 -4.781439 1.491492

Cl 8.274133 -2.144194 -1.245888

C 3.469243 -0.656542 0.248584

O 2.401596 -0.871178 0.853803

O 3.717721 0.417377 -0.407486

O 2.047313 1.440961 -0.158559

H 1.827653 0.610097 0.363154

O -3.017393 -0.602450 0.935269

C -4.346196 -0.148803 0.982379

O -4.670466 0.857649 1.534104

C -5.210599 -1.092467 0.229355

C -4.782323 -2.363162 -0.174417

C -6.509839 -0.654844 -0.056431

C -5.660428 -3.190350 -0.867416

H -3.783155 -2.704375 0.059690

C -7.365135 -1.496671 -0.752526

H -6.831978 0.325702 0.267818

C -6.953112 -2.763832 -1.162676

H -5.339998 -4.177156 -1.179052

H -7.638165 -3.405224 -1.702379

Cl -8.991527 -0.958484 -1.120551

O -2.205583 0.149740 1.847383

B3LYP/6-311++G**

Total electronic energy = -655.309139 Ha

Zero-point correction = 0.100301 Ha

Thermal correction to enthalpy = 0.116743 Ha

Thermal correction to free energy = 0.043691 Ha

C 1.432162 -1.853345 0.000031

C 2.740584 -1.565448 0.000242

C 3.222404 -0.160175 0.000140

C 2.194857 0.912090 -0.000231

C 0.886142 0.624576 -0.000442

C 0.405847 -0.780171 -0.000334

H 1.056465 -2.870640 0.000108

H 3.508863 -2.330823 0.000503

H 2.569972 1.929586 -0.000318

H 0.120182 1.391990 -0.000720

N -3.747306 -1.636232 1.176988

N -3.741878 -1.635764 0.000260

N -3.748041 -1.637204 -1.176468

N -2.008625 4.738542 0.000553

N -1.264196 3.934900 -0.000543

O 4.413748 0.101495 0.000346

O -0.785889 -1.043366 -0.000537

B3LYP/6-311++G**

Imaginary frequency = 404.0i cm^-1^

Total electronic energy = -655.186304 Ha

Zero-point correction = 0.102504 Ha

Thermal correction to enthalpy = 0.114451 Ha

Thermal correction to free energy = 0.061833 Ha

C 1.829342 0.019343 -1.327486

C 0.817748 -0.839960 -1.189261

C 0.347473 -1.245394 0.165133

C 0.971267 -0.571755 1.360168

C 1.987669 0.271032 1.187651

C 2.513569 0.627863 -0.156680

H 2.195465 0.322058 -2.302072

H 0.309464 -1.312166 -2.020851

H 0.566020 -0.857097 2.321318

H 2.475815 0.748206 2.030506

N -1.381757 0.056547 0.154764

N -1.671052 1.284648 0.534625

N -2.891812 1.506633 0.160902

N -3.376046 0.378988 -0.472933

N -2.448584 -0.493472 -0.473477

O 3.451666 1.388746 -0.292270

O -0.197219 -2.338895 0.343119

B3LYP/6-311++G**

Imaginary frequency = 442.1i cm^-1^

Total electronic energy = -655.210874 Ha

Zero-point correction = 0.107567 Ha

Thermal correction to enthalpy = 0.118038 Ha

Thermal correction to free energy = 0.070508 Ha

C -1.973531 1.325387 -0.160228

C -0.670075 1.304151 0.130375

C 0.023841 -0.001392 0.404292

C -0.696988 -1.251116 0.045980

C -2.054180 -1.194426 -0.228303

C -2.778174 0.086840 -0.295713

H -2.508787 2.255716 -0.309773

H -0.085767 2.209462 0.240767

H -0.122361 -2.163559 0.000963

H -2.619589 -2.093501 -0.443230

N 1.439454 0.035291 -0.009215

N 2.115964 -1.007177 -0.475935

N 3.331784 -0.589770 -0.609791

N 3.400100 0.702530 -0.218866

N 2.226412 1.091206 0.158581

O -0.185709 -0.546942 1.643150

O -3.984898 0.115774 -0.490720

B3LYP/6-311++G**

Total electronic energy = - 655.219036 Ha

Zero-point correction = 0.108069 Ha

Thermal correction to enthalpy = 0.119227 Ha

Thermal correction to free energy = 0.069815 Ha

C 1.958957 -1.259157 -0.180687

C 0.717098 -1.280496 0.307449

C -0.016058 -0.000448 0.684840

C 0.717128 1.280167 0.308788

C 1.958961 1.259084 -0.179596

C 2.702731 0.000026 -0.424209

H 2.477093 -2.176739 -0.436659

H 0.177293 -2.196810 0.506886

H 0.177326 2.196262 0.509170

H 2.477210 2.176868 -0.434657

N -1.363662 -0.000001 0.012942

N -2.063544 1.096884 -0.246979

N -3.204747 0.675788 -0.686802

N -3.205390 -0.675143 -0.686059

N -2.064497 -1.096588 -0.245811

O -0.133600 -0.000440 2.030145

O 3.854981 0.000289 -0.814556

B3LYP/6-311++G**

Total electronic energy = -655.220022 Ha

Zero-point correction = 0.108737 Ha

Thermal correction to enthalpy = 0.119350 Ha

Thermal correction to free energy = 0.071662 Ha

C -1.980589 1.335801 -0.124853

C -0.656990 1.320356 0.070034

C 0.047654 0.029957 0.284919

C -0.683339 -1.251615 0.121029

C -2.099693 -1.167378 -0.217640

C -2.795200 0.100126 -0.245658

H -2.521198 2.267483 -0.243495

H -0.068321 2.228602 0.109044

H -0.127000 -2.140128 -0.152045

H -2.659830 -2.060759 -0.466183

N 1.464690 0.051154 -0.020272

N 2.143711 -0.962276 -0.549204

N 3.371223 -0.564230 -0.584251

N 3.445310 0.688958 -0.078390

N 2.264161 1.071031 0.276806

O -0.287918 -0.716474 1.434986

O -4.016878 0.155081 -0.420877

B3LYP/6-311++G**

Total electronic energy = -1535.091051 Ha

Zero-point correction = 0.201388 Ha

Thermal correction to enthalpy = 0.221198 Ha

Thermal correction to free energy = 0.149556 Ha

C -1.521496 -0.673911 0.238842

C -2.666616 -0.722088 -0.457627

C -3.895264 -0.104653 0.096279

C -3.814547 0.736699 1.329836

C -2.490394 0.890219 1.922072

C -1.411494 0.093299 1.486892

H -0.614569 -1.166974 -0.093685

H -2.740688 -1.234428 -1.408680

H -4.552146 1.514538 1.489454

H -2.321607 1.604053 2.719606

N -4.922719 0.213408 -0.873793

N -5.717388 1.279049 -0.817968

N -6.527669 1.147665 -1.814191

N -6.231585 0.005743 -2.479108

N -5.239321 -0.578319 -1.894736

O -4.389715 -0.607120 1.320153

O 1.220058 0.763899 1.202007

C 1.934572 -0.194704 0.671603

O 1.575051 -1.313847 0.359405

C 3.340661 0.313189 0.411581

C 3.941758 1.272418 1.233636

C 4.029651 -0.224827 -0.680065

C 5.249163 1.675675 0.972509

H 3.391345 1.682729 2.069777

C 5.327294 0.201793 -0.926094

H 3.557630 -0.976202 -1.299274

C 5.948827 1.148342 -0.110049

H 5.728885 2.407627 1.612331

H 6.962807 1.462134 -0.323215

Cl 6.211329 -0.469316 -2.287546

O -0.365279 0.064444 2.208689

The xyz coordinates of geometries in Fig. 13:

­­­___________________________________________________________

B3LYP/6-311++G**(HNO) + LanL2DZ(Co)

Total electronic energy = -998.553448 Ha

Zero-point correction = 0.149932 Ha

Thermal correction to enthalpy = 0.169956 Ha

Thermal correction to free energy = 0.100914 Ha

H 1.253336 -2.186751 0.805824

H -1.276365 0.734257 2.128958

H 1.252676 2.186814 -0.805919

H -1.276002 -0.734488 -2.128840

H -0.170049 2.455495 -1.405134

H 0.023740 -1.596897 -2.178875

H -0.169566 -2.456321 1.404380

H 0.023438 1.596537 2.179966

Co 0.022340 0.000124 0.000073

O 0.293889 -2.108622 0.635011

O -0.300307 0.700341 2.029061

O 0.293230 2.108575 -0.635301

O -0.299948 -0.700526 -2.028863

N 2.069385 0.000291 0.000267

N -2.074358 -0.000221 0.000070

N 2.838801 -1.004243 0.388943

N 4.080404 -0.622493 0.240023

N -2.840080 0.333927 1.020976

N -4.088241 0.205568 0.630108

N 2.838883 1.004511 -0.388747

N 4.080493 0.622397 -0.240409

N -2.839910 -0.334198 -1.021019

N -4.088134 -0.205560 -0.630438

B3LYP/6-311++G**(HNO) + LanL2DZ(Co)

Total electronic energy = -998.545840 Ha

Zero-point correction = 0.151119 Ha

Thermal correction to enthalpy = 0.170775 Ha

Thermal correction to free energy = 0.102313 Ha

H -1.113851 2.170177 0.776585

H 1.112624 -0.776835 2.167692

H -1.113851 -2.170199 -0.776594

H 1.112596 0.776822 -2.167692

H 0.297497 -2.277286 -1.439560

H -0.297644 1.443058 -2.268063

H 0.297484 2.277235 1.439589

H -0.297619 -1.443064 2.268090

Co 0.000350 -0.000010 0.000003

O -0.155722 2.054478 0.618549

O 0.154461 -0.620062 2.050148

O -0.155728 -2.054499 -0.618538

O 0.154433 0.620050 -2.050141

N -1.930734 -0.000013 0.000010

N 1.931168 -0.000002 -0.000006

N -2.692013 1.017611 0.352462

N -3.937737 0.627842 0.216734

N 2.692411 -0.352850 1.017468

N 3.938144 -0.216967 0.627715

N -2.692046 -1.017609 -0.352458

N -3.937759 -0.627787 -0.216745

N 2.692398 0.352859 -1.017484

N 3.938136 0.217005 -0.627736

B3LYP/6-311++G**(HNO) + LanL2DZ(Co)

Total electronic energy = -998.547929 Ha

Zero-point correction = 0.148648 Ha

Thermal correction to enthalpy = 0.169161 Ha

Thermal correction to free energy = 0.097173 Ha

H -0.485473 -2.576733 -0.649999

H 1.066131 2.209692 -0.574242

H -2.581179 2.255757 -0.090584

H -0.685153 0.098308 2.660061

H -2.531414 3.664649 -0.777459

H 0.888822 0.157439 2.648001

H 1.141468 -2.480343 -0.626756

H -0.561561 2.429483 -0.558751

Co 0.153406 -0.110575 0.039232

O 0.299699 -2.007247 -0.709835

O 0.196073 1.786249 -0.643715

O -2.036902 3.068437 -0.207038

O 0.104431 -0.054602 2.128395

N -1.920596 -0.405483 -0.037545

N 2.215196 -0.061058 0.017236

N -2.393881 -1.631497 -0.202513

N -3.702763 -1.548207 -0.228796

N 2.949270 1.037958 -0.054100

N 4.195408 0.656805 -0.207152

N -2.941866 0.431302 0.037570

N -4.042344 -0.274474 -0.080604

N 3.000495 -1.119998 -0.099446

N 4.226794 -0.673263 -0.234934

B3LYP/6-311++G**(HNO) + LanL2DZ(Co)

Total electronic energy = -998.546086 Ha

Zero-point correction = 0.151441 Ha

Thermal correction to enthalpy = 0.170623 Ha

Thermal correction to free energy = 0.103669 Ha

H 0.844822 -1.740674 1.793573

H -1.011884 2.018627 0.837425

H 2.294150 2.402886 -0.604898

H -1.274941 -1.979673 -1.186273

H 2.210370 3.879673 -0.084550

H 0.209563 -2.483490 -0.930639

H -0.322108 -1.079254 2.590787

H 0.520911 2.334808 0.450447

Co -0.136233 -0.101507 -0.050951

O -0.075861 -1.438646 1.731425

O -0.105620 1.659946 0.835374

O 1.681135 3.140279 -0.398415

O -0.327985 -1.727209 -1.199204

N 1.806425 -0.274231 -0.106712

N -2.056366 0.035268 -0.107025

N 2.375254 -1.400100 0.294026

N 3.669079 -1.258768 0.126306

N -2.777805 1.051979 0.322963

N -4.033022 0.744604 0.103163

N 2.741619 0.557259 -0.521013

N 3.895477 -0.052941 -0.375443

N -2.851741 -0.899363 -0.593304

N -4.078341 -0.459019 -0.463479

B3LYP/6-311++G**(HNO) + LanL2DZ(Co)

Total electronic energy = -998.555784 Ha

Zero-point correction = 0.149354 Ha

Thermal correction to enthalpy = 0.169312 Ha

Thermal correction to free energy = 0.098439 Ha

H -1.986917 2.839720 0.044934

H 1.342023 -2.218233 0.811630

H -1.987667 -2.839526 0.044597

H 1.342580 2.217294 0.810709

H -2.038954 -4.040937 1.055153

H -0.201561 2.576978 1.042197

H -2.039413 4.039816 1.056660

H -0.202525 -2.577208 1.041188

Co 0.224792 -0.000307 0.395257

O -1.445134 3.491988 0.535364

O 0.499407 -1.873882 1.152257

O -1.445356 -3.491631 0.534655

O 0.500086 1.873425 1.152137

N -1.685834 -0.000093 -0.300227

N 2.078686 -0.000301 -0.348914

N -2.419288 1.074923 -0.559298

N -3.588579 0.665866 -0.970778

N 2.831202 -1.083354 -0.499321

N 4.040823 -0.668930 -0.758211

N -2.420004 -1.074665 -0.559134

N -3.589006 -0.664855 -0.970732

N 2.830373 1.083298 -0.499861

N 4.040343 0.669709 -0.758425

B3LYP/6-311++G**(HNO) + LanL2DZ(Co)

Total electronic energy = -998.546167 Ha

Zero-point correction = 0.151936 Ha

Thermal correction to enthalpy = 0.170575 Ha

Thermal correction to free energy = 0.104323 Ha

H 2.005855 -2.860788 0.453793

H -1.268143 2.074967 0.744234

H 1.947172 2.842788 -0.549771

H -1.263050 -2.080310 -0.763555

H 1.657412 4.309079 -0.075555

H 0.233179 -2.548040 -0.366126

H 1.288908 -4.156746 0.977384

H 0.224874 2.542480 0.343680

Co -0.225655 -0.001009 -0.000003

O 1.356138 -3.557223 0.228198

O -0.330346 1.802018 0.726460

O 1.247725 3.520492 -0.444629

O -0.328271 -1.801720 -0.724173

N 1.733983 0.002274 0.003817

N -2.152791 -0.004368 0.004980

N 2.513041 -1.018065 0.326687

N 3.753639 -0.615141 0.223492

N -2.915220 1.020598 0.341433

N -4.155816 0.626884 0.219260

N 2.503616 1.034784 -0.299563

N 3.747894 0.653281 -0.164167

N -2.914927 -1.031463 -0.325919

N -4.155630 -0.640888 -0.194428

B3LYP/6-311++G**(HNO) + LanL2DZ(Co)

Total electronic energy = -724.760300 Ha

Zero-point correction = 0.123867 Ha

Thermal correction to enthalpy = 0.140376 Ha

Thermal correction to free energy = 0.079235 Ha

Co 1.006646 -0.455213 -0.214581

N -1.005231 -0.197905 0.039689

N -1.796995 -1.261040 0.068792

N -3.036073 -0.841350 0.192608

N -1.768454 0.876825 0.142827

N -3.019415 0.480775 0.240242

O 0.520556 -2.576631 -0.480408

H 0.906435 -3.318785 -0.006313

O -0.257872 3.160010 -0.079399

H -0.952923 2.461244 0.033006

H -0.411486 3.827064 0.595403

H -0.440456 -2.558989 -0.279723

O 2.787538 -0.379560 1.004380

H 2.951358 0.574306 0.954373

O 1.827458 1.603191 -0.509517

H 2.163145 1.821497 -1.385201

H 2.850856 -0.645835 1.926746

H 1.115361 2.273024 -0.314138

B3LYP/6-311++G**(HNO) + LanL2DZ(Co)

Total electronic energy = -889.036316 Ha

Zero-point correction = 0.137471 Ha

Thermal correction to enthalpy = 0.157556 Ha

Thermal correction to free energy = 0.087711 Ha

H -1.045980 -1.574956 2.189999

H 0.937286 -1.880247 -1.258837

H -2.049845 1.003908 -1.835406

H 0.773283 2.044953 0.978543

H -0.783707 1.848971 -1.596672

H -0.640350 2.190204 1.659308

H 0.452853 -1.997567 2.079902

H -0.464189 -1.990481 -1.957960

Co -0.467612 -0.155995 0.141241

O -0.147968 -1.245453 2.037908

O -0.030414 -1.910162 -1.101941

O -1.090614 0.940723 -1.712464

O -0.193655 1.945955 0.841424

N -2.375360 -0.440451 0.679690

N 1.628909 0.067965 -0.054020

N 2.431255 -0.787104 -0.659627

N 3.653252 -0.310471 -0.571317

N -3.277919 0.033254 0.039868

N -4.132895 0.485455 -0.586063

N 2.348779 1.072338 0.407864

N 3.602176 0.838809 0.087633

B3LYP/6-311++G**(HNO) + LanL2DZ(Co)

Total electronic energy = -889.0451518 Ha

Zero-point correction = 0.137276 Ha

Thermal correction to enthalpy = 0.157031 Ha

Thermal correction to free energy = 0.086188 Ha

H -2.371229 -2.148804 -0.182332

H 1.902836 0.932575 2.360606

H -0.404975 2.959055 0.381012

H 0.938989 -2.853825 0.918315

H 0.746342 4.010378 0.551808

H -0.631853 -2.663527 0.901876

H -2.463523 -3.715717 -0.217286

H 0.891616 1.943730 1.638970

Co 0.636550 -0.373992 0.377761

O -2.149338 -2.972253 0.304980

O 1.088326 0.986023 1.851512

O 0.265036 3.313482 1.007895

O 0.231942 -2.250735 1.177019

N -1.157747 0.359177 -0.286814

N 2.150528 -0.923754 -0.637638

N -2.204563 -0.348428 -0.681136

N -3.121695 0.491525 -1.090679

N 2.991875 -0.353476 -1.293146

N 3.816294 0.126941 -1.925311

N -1.438716 1.642433 -0.458154

N -2.647803 1.722980 -0.953373

B3LYP/6-311++G**(HNO) + LanL2DZ(Co)

Total electronic energy = -889.040559 Ha

Zero-point correction = 0.137734 Ha

Thermal correction to enthalpy = 0.157424 Ha

Thermal correction to free energy = 0.087453 Ha

H -1.927616 2.730006 -0.297022

H 0.429535 -2.200089 0.674353

H -3.232448 -2.457399 -0.087776

H 1.385471 2.317185 0.708145

H -3.225736 -3.411920 1.141545

H -0.135944 2.823956 0.742166

H -2.168755 4.098405 0.420687

H -1.168276 -2.192815 0.849906

Co -0.079133 0.253217 0.225566

O -1.492819 3.548393 0.013723

O -0.338630 -1.663203 0.934479

O -2.655965 -2.955675 0.514977

O 0.485405 2.092588 0.987770

N -1.834237 0.763399 -0.496909

N 1.812157 -0.210122 -0.239281

N 2.282971 -1.451396 -0.257726

N 3.576383 -1.364989 -0.407719

N -2.700732 0.001120 -0.845513

N -3.549196 -0.698561 -1.172698

N 2.818721 0.642704 -0.367967

N 3.907710 -0.070871 -0.476457

B3LYP/6-311++G**(HNO) + LanL2DZ(Co)

Imaginary frequency = 604.9i cm^-1^

Total electronic energy = -998.518746 Ha

Zero-point correction = 0.145192 Ha

Thermal correction to enthalpy = 0.166489 Ha

Thermal correction to free energy = 0.093842 Ha

H 1.124992 -2.175267 -0.772764

H -1.310594 -0.769366 2.144744

H 1.171597 2.349138 0.904575

H -1.332112 1.075241 -1.993678

H -0.207745 2.773503 0.355165

H 0.139851 1.098289 -2.552820

H -0.374669 -2.611921 -0.593495

H -0.062091 -0.105298 2.827821

Co 0.035697 0.101345 0.065739

O 0.199042 -1.881678 -0.847895

O -0.340707 -0.643348 2.079013

O 0.227208 2.137084 0.936647

O -0.397564 1.279239 -1.773883

N 2.043878 0.069175 -0.039338

N -2.068168 -0.071291 -0.028322

N 2.947958 -1.338641 -0.407516

N 4.083773 -1.074689 -0.316674

N -2.833097 -0.573639 0.923079

N -4.075556 -0.516456 0.498648

N 2.979438 0.883214 0.206083

N 4.169373 0.658860 0.166777

N -2.832112 0.297751 -1.039048

N -4.075042 0.022712 -0.713054

B3LYP/6-311++G**(HNO) + LanL2DZ(Co)

Imaginary frequency = 604.5i cm^-1^

Total electronic energy = -998.515800 Ha

Zero-point correction = 0.144986 Ha

Thermal correction to enthalpy = 0.166314 Ha

Thermal correction to free energy = 0.092231 Ha

H -2.183762 2.947267 0.177276

H 1.432374 -2.189067 0.743448

H -2.017454 -3.181094 0.104779

H 1.289662 2.282994 0.706974

H -1.842141 -4.257909 1.210444

H -0.256138 2.588860 0.998396

H -2.084266 4.064361 1.264178

H -0.091912 -2.587498 1.020709

Co 0.236936 0.006351 0.303435

O -1.566971 3.536639 0.648341

O 0.581810 -1.868091 1.088847

O -1.361962 -3.649242 0.640441

O 0.463781 1.905946 1.051416

N -1.641196 -0.063116 -0.282576

N 2.134383 0.046098 -0.348035

N -2.783227 1.214028 -0.546745

N -3.779958 0.721534 -0.908473

N 2.913224 -1.024067 -0.441213

N 4.124748 -0.592728 -0.667366

N -2.359337 -1.053016 -0.603015

N -3.515104 -1.022532 -0.972949

N 2.869257 1.138775 -0.502764

N 4.097655 0.743392 -0.705621

B3LYP/6-311++G**(HNO) + LanL2DZ(Co)

Imaginary frequency = 613.6i cm^-1^

Total electronic energy = -998.523704 Ha

Zero-point correction = 0.145495 Ha

Thermal correction to enthalpy = 0.166196 Ha

Thermal correction to free energy = 0.093503 Ha

H -1.462779 2.959473 0.053216

H 0.639839 -2.310354 1.931093

H -2.335129 -2.542492 -0.059784

H 1.651835 1.796270 1.171580

H -2.732869 -3.692530 0.929989

H 0.173318 2.382420 1.242712

H -1.420777 4.133304 1.091948

H -0.833790 -2.440191 1.334845

Co 0.224326 -0.165251 0.453534

O -0.872660 3.501836 0.616999

O -0.126235 -1.799903 1.646854

O -1.981240 -3.240748 0.534319

O 0.736699 1.581002 1.419888

N -1.614432 0.158114 -0.373562

N 1.984190 -0.336483 -0.342626

N -2.148217 1.333287 -0.671913

N -3.322940 1.111537 -1.202115

N 2.654366 -1.167949 -1.033544

N 3.809050 -1.009159 -1.365621

N -2.471642 -0.788550 -0.728350

N -3.523252 -0.201185 -1.237139

N 3.120102 0.886751 -0.091142

N 4.114351 0.561979 -0.623348

B3LYP/6-311++G**(HNO) + LanL2DZ(Fe)

Total electronic energy = -976.928146 Ha

Zero-point correction =0.148297 Ha

Thermal correction to enthalpy = 0.169102 Ha

Thermal correction to free energy = 0.095728 Ha

H 1.340296 -2.041934 1.113346

H -1.336243 1.134675 1.961804

H 1.295285 1.996099 -1.211624

H -1.353809 -1.140788 -1.926221

H -0.130949 2.213734 -1.846810

H -0.021822 -1.968347 -1.945718

H -0.103085 -2.410289 1.629567

H 0.069740 1.831612 2.134904

Fe 0.019917 -0.000682 -0.001091

O 0.386377 -2.023875 0.896125

O -0.364093 1.003242 1.903148

O 0.332575 1.927598 -1.053036

O -0.374644 -1.073432 -1.880506

N 2.123841 0.015361 0.006166

N -2.127640 0.006934 0.023280

N 2.901428 -0.891668 0.575372

N 4.142476 -0.527930 0.369449

N -2.890935 0.570072 0.940290

N -4.142032 0.372985 0.584824

N 2.887539 0.940732 -0.552197

N 4.133857 0.607581 -0.326959

N -2.898725 -0.536907 -0.899005

N -4.146805 -0.309345 -0.550753

B3LYP/6-311++G**(HNO) + LanL2DZ(Fe)

Total electronic energy = -976.908320 Ha

Zero-point correction = 0.150395 Ha

Thermal correction to enthalpy = 0.170199 Ha

Thermal correction to free energy = 0.101941 Ha

H 1.150627 1.421394 1.832954

H -1.149380 1.832339 -1.423273

H 1.149402 -1.424245 -1.830676

H -1.150436 -1.831946 1.422436

H -0.309604 -2.014928 -1.879371

H 0.308550 -1.883339 2.012765

H -0.308751 2.011146 1.885048

H 0.311031 1.886724 -2.010497

Fe 0.000063 0.000276 -0.000191

O 0.199320 1.198649 1.786810

O -0.198444 1.786292 -1.199163

O 0.198052 -1.201758 -1.784619

O -0.199090 -1.786112 1.199863

N 1.966738 0.000436 -0.000384

N -1.966765 0.000513 -0.000049

N 2.730889 0.674533 0.838436

N 3.977956 0.415102 0.516929

N -2.730597 0.838887 -0.675023

N -3.977787 0.517553 -0.416044

N 2.730356 -0.674047 -0.839374

N 3.977595 -0.415262 -0.518056

N -2.730787 -0.838078 0.674608

N -3.977852 -0.516908 0.415022

B3LYP/6-311++G**(HNO) + LanL2DZ(Fe)

Total electronic energy = -976.922319 Ha

Zero-point correction = 0.147094 Ha

Thermal correction to enthalpy = 0.168412 Ha

Thermal correction to free energy = 0.093381 Ha

H -0.405322 -2.715027 -0.549720

H 1.076869 2.271580 -0.633001

H -2.575153 2.239540 -0.066287

H -0.692722 0.162028 2.698673

H -2.551476 3.668449 -0.711154

H 0.874101 0.233223 2.674682

H 1.209361 -2.534223 -0.517995

H -0.545238 2.471368 -0.605320

Fe 0.140185 -0.083949 -0.014655

O 0.343293 -2.101008 -0.564684

O 0.215716 1.842242 -0.751307

O -2.031922 3.052520 -0.185701

O 0.087968 0.068767 2.141521

N -1.946676 -0.431696 -0.067832

N 2.238172 -0.072211 0.000244

N -2.430885 -1.654004 -0.229455

N -3.739203 -1.561374 -0.209593

N 2.987883 1.019370 -0.013625

N 4.232758 0.626189 -0.147100

N -2.960793 0.411868 0.050272

N -4.067279 -0.286402 -0.041683

N 3.014627 -1.137293 -0.129937

N 4.249158 -0.702222 -0.215216

B3LYP/6-311++G**(HNO) + LanL2DZ(Fe)

Total electronic energy = -976.907887 Ha

Zero-point correction = 0.149989 Ha

Thermal correction to enthalpy = 0.169807 Ha

Thermal correction to free energy = 0.099716 Ha

H 0.856269 -2.081577 1.492795

H -0.977020 2.105747 0.816042

H 2.360188 2.366585 -0.595076

H -1.384901 -1.611952 -1.624647

H 2.388226 3.814629 -0.000614

H 0.116917 -2.102096 -1.809555

H -0.608473 -2.023545 2.126194

H 0.575269 2.394032 0.398714

Fe -0.144073 -0.136005 0.020942

O -0.068911 -1.801955 1.361432

O -0.089585 1.721817 0.718579

O 1.806965 3.148100 -0.379313

O -0.443763 -1.340521 -1.625817

N 1.834128 -0.314275 -0.037161

N -2.096140 0.016644 0.036412

N 2.435877 -1.392217 0.442165

N 3.725945 -1.229113 0.272205

N -2.800262 0.998793 0.565726

N -4.059046 0.763763 0.276030

N 2.752931 0.511500 -0.502302

N 3.922498 -0.054988 -0.309818

N -2.907800 -0.820873 -0.586500

N -4.125021 -0.357121 -0.434957

B3LYP/6-311++G**(HNO) + LanL2DZ(Fe)

Total electronic energy = -976.918937 Ha

Zero-point correction = 0.148389 Ha

Thermal correction to enthalpy = 0.168774 Ha

Thermal correction to free energy = 0.096324 Ha

H -2.406766 -2.473545 0.035261

H 1.647153 1.696516 -1.286094

H -1.433381 2.938757 -0.172604

H 0.646211 -2.467414 -1.845796

H -1.455882 4.059001 -1.270254

H -0.878050 -2.455438 -1.356756

H -2.888313 -3.518855 -1.033077

H 0.166588 2.280960 -1.452829

Fe 0.234901 -0.209992 -0.460823

O -2.105855 -3.146235 -0.615204

O 0.736484 1.474508 -1.560638

O -0.891583 3.414572 -0.833651

O -0.087856 -1.886654 -1.616440

N -1.576690 0.166496 0.453656

N 2.065829 -0.249750 0.390728

N -2.458627 -0.757647 0.817248

N -3.493285 -0.142117 1.320961

N 2.915783 0.726674 0.056093

N 4.048321 0.460278 0.632877

N -2.084036 1.357999 0.742910

N -3.260747 1.168279 1.275466

N 2.689780 -1.116147 1.176821

N 3.908745 -0.678313 1.329100

B3LYP/6-311++G**(HNO) + LanL2DZ(Fe)

Total electronic energy = -976.907031 Ha

Zero-point correction = 0.151260 Ha

Thermal correction to enthalpy = 0.170084 Ha

Thermal correction to free energy = 0.101438 Ha

H -2.139885 2.926128 -0.099231

H 1.338413 -2.232063 0.024244

H -2.139818 -2.926080 -0.099317

H 1.338373 2.232036 0.024142

H -1.749065 -4.329842 0.480997

H -0.251962 2.676606 0.010660

H -1.749213 4.329938 0.481013

H -0.251930 -2.676643 0.010783

Fe 0.258612 -0.000025 0.017726

O -1.489688 3.657585 -0.156575

O 0.412784 -1.926498 0.068385

O -1.489589 -3.657517 -0.156645

O 0.412736 1.926470 0.068196

N -1.744902 -0.000045 0.006732

N 2.218990 0.000006 0.001862

N -2.525735 1.071370 0.011622

N -3.768566 0.662672 0.020738

N 2.982862 -1.077791 -0.006952

N 4.225271 -0.665752 -0.021305

N -2.525778 -1.071429 0.011622

N -3.768593 -0.662687 0.020740

N 2.982815 1.077838 -0.006833

N 4.225241 0.665854 -0.021234

B3LYP/6-311++G**(HNO) + LanL2DZ(Fe)

Total electronic energy = -703.114077 Ha

Zero-point correction = 0.123354 Ha

Thermal correction to enthalpy = 0.140211 Ha

Thermal correction to free energy = 0.078044 Ha

H -0.976914 2.451719 0.089189

H 0.868615 -3.412118 -0.106039

H 2.026260 1.806454 -1.442469

H 1.116056 2.312830 -0.279079

H -0.452575 3.803259 0.696284

H -0.479026 -2.593928 -0.248335

Fe 1.016574 -0.439740 -0.053775

O -0.287269 3.157419 0.003819

O 0.493697 -2.566994 -0.366749

O 1.826808 1.655120 -0.511933

N -1.050196 -0.226630 0.057407

N -1.809171 0.854337 0.139483

N -3.064755 0.470305 0.150339

N -1.851982 -1.281428 0.014073

N -3.090612 -0.852886 0.070662

O 3.067473 -0.418129 0.586950

H 3.271676 0.528895 0.603315

H 3.456314 -0.829075 1.364841

B3LYP/6-311++G**(HNO) + LanL2DZ(Fe)

Total electronic energy = -867.406113 Ha

Zero-point correction = 0.136567 Ha

Thermal correction to enthalpy = 0.157146 Ha

Thermal correction to free energy = 0.085424 Ha

H 0.845057 -1.947985 1.202217

H -1.321708 1.638248 2.018097

H 1.025179 1.935955 -1.271773

H -1.710770 -1.352981 -2.016884

H -0.386788 2.306690 -1.857244

H -0.814940 -2.191152 -1.073165

H -0.499340 -2.038578 2.010285

H 0.016237 2.366193 1.750711

Fe -0.480036 0.159069 0.109784

O -0.133056 -1.892201 1.131727

O -0.363950 1.502669 1.951044

O 0.073479 2.035282 -1.056849

O -0.870206 -1.347793 -1.543291

N 1.647245 -0.060781 -0.065604

N -2.424199 0.424066 0.444240

N 2.366780 -1.028186 0.474386

N 3.619125 -0.819389 0.145932

N -3.377519 -0.061562 -0.109851

N -4.293392 -0.515750 -0.632870

N 2.455763 0.747370 -0.727768

N 3.674465 0.280540 -0.596704

B3LYP/6-311++G**(HNO) + LanL2DZ(Fe)

Total electronic energy = -867.413848 Ha

Zero-point correction = 0.137204 Ha

Thermal correction to enthalpy = 0.157133 Ha

Thermal correction to free energy = 0.084740 Ha

H 1.767186 -2.577106 -0.145749

H -1.456310 1.847504 2.206845

H 1.236934 2.816140 0.115776

H -1.155918 -2.445962 1.817964

H 1.556873 3.882207 1.218590

H 0.347056 -2.476892 1.326571

H 2.290678 -3.647142 0.873551

H -0.058954 2.262669 1.592878

Fe -0.771229 -0.137287 0.517128

O 1.489572 -3.269939 0.497567

O -0.630808 1.487408 1.866202

O 0.841431 3.373901 0.824674

O -0.394499 -1.882077 1.644606

N 1.112850 0.097687 -0.429380

N -2.349104 -0.191833 -0.494442

N 1.889993 -0.880982 -0.869981

N 2.926408 -0.340315 -1.459779

N -3.236803 -0.064538 -1.287149

N -4.097062 0.044936 -2.035377

N 1.682192 1.247008 -0.761336

N 2.797073 0.978565 -1.393434

B3LYP/6-311++G**(HNO) + LanL2DZ(Fe)

Total electronic energy = -867.407454 Ha

Zero-point correction = 0.137190 Ha

Thermal correction to enthalpy = 0.157134 Ha

Thermal correction to free energy = 0.086024 Ha

H 1.820300 2.843122 0.303968

H -0.415388 -2.293375 -0.287954

H 3.394032 -2.430697 0.126166

H -1.625331 2.222491 -0.480797

H 3.190123 -3.614742 -0.857254

H -0.155737 2.907433 -0.470584

H 1.834810 4.316492 -0.207477

H 1.191396 -2.333854 -0.472088

Fe 0.104973 0.270395 -0.214539

O 1.273658 3.642307 0.187152

O 0.373484 -1.788470 -0.553617

O 2.734057 -3.050582 -0.225181

O -0.696357 2.131828 -0.754072

N 1.945162 0.804644 0.240244

N -1.869669 -0.264677 0.138982

N -2.314962 -1.507614 0.248256

N -3.615042 -1.440173 0.372936

N 2.928486 0.149364 0.466927

N 3.877373 -0.459448 0.677676

N -2.897012 0.570356 0.189239

N -3.974657 -0.156424 0.335708

B3LYP/6-311++G**(HNO) + LanL2DZ(Fe)

Imaginary frequency = 603.2i cm^-1^

Total electronic energy = -976.888110 Ha

Zero-point correction = 0.144266 Ha

Thermal correction to enthalpy = 0.165949 Ha

Thermal correction to free energy = 0.092025 Ha

H -1.334089 -0.015328 -2.259594

H 0.942757 2.636693 0.135210

H -1.362107 -0.004379 2.256351

H 1.341336 -2.290802 -0.016376

H 0.056398 -0.471050 2.761325

H -0.106184 -2.788407 -0.368748

H 0.050309 0.544414 -2.771768

H -0.591640 2.851572 0.306106

Fe 0.048959 0.054844 0.009234

O -0.359533 -0.070286 -2.154114

O 0.073399 2.296816 -0.116917

O -0.394486 0.138699 2.167163

O 0.390924 -2.142439 0.143641

N -2.116815 -0.044906 -0.004908

N 2.076178 0.134419 0.012144

N -2.879474 -0.055369 -1.081851

N -4.130723 -0.114855 -0.678546

N 2.955616 1.045764 0.061645

N 4.156759 0.897929 0.043024

N -2.890220 -0.096396 1.063226

N -4.137450 -0.138937 0.645631

N 3.069977 -1.248274 -0.079606

N 4.188703 -0.903802 -0.066583

B3LYP/6-311++G**(HNO) + LanL2DZ(Fe)

Imaginary frequency = 621.4i cm^-1^

Total electronic energy = -976.888416 Ha

Zero-point correction = 0.145036 Ha

Thermal correction to enthalpy = 0.165933 Ha

Thermal correction to free energy = 0.092662 Ha

H -1.364160 2.945259 0.150541

H 0.469171 -2.451580 1.994955

H -2.484716 -2.409815 -0.055028

H 1.660534 1.673697 1.340311

H -3.036103 -3.446974 0.984842

H 0.184516 2.260096 1.440008

H -1.398547 4.051061 1.261893

H -1.009852 -2.423382 1.389294

Fe 0.243482 -0.250929 0.456562

O -0.823958 3.424386 0.812873

O -0.224742 -1.864877 1.675621

O -2.227171 -3.103171 0.593588

O 0.741931 1.445891 1.562674

N -1.574098 0.196892 -0.453655

N 2.003824 -0.371145 -0.388130

N -2.045458 1.400191 -0.750312

N -3.223766 1.243445 -1.293949

N 2.684781 -1.129138 -1.152641

N 3.841791 -0.935454 -1.455625

N -2.477798 -0.701512 -0.824898

N -3.491668 -0.058279 -1.340421

N 3.134557 0.818323 -0.004007

N 4.136428 0.552101 -0.555715

B3LYP/6-311++G**(HNO) + LanL2DZ(Fe)

Imaginary frequency = 615.6i cm^-1^

Total electronic energy = -976.879756 Ha

Zero-point correction = 0.143882 Ha

Thermal correction to enthalpy = 0.165767 Ha

Thermal correction to free energy = 0.089251 Ha

H 2.518162 -2.587013 0.168466

H -1.684856 1.727369 1.188172

H 1.508430 3.200996 0.247917

H -0.860952 -2.513850 1.502067

H 1.398440 4.212491 1.422060

H 0.724621 -2.485869 1.334549

H 2.787255 -3.571482 1.351207

H -0.218204 2.298699 1.464847

Fe -0.222888 -0.172688 0.386891

O 2.091789 -3.193501 0.804268

O -0.790054 1.499760 1.509577

O 0.935276 3.504063 0.965243

O -0.093641 -1.930744 1.479742

N 1.541174 0.197492 -0.447711

N -2.102129 -0.243645 -0.400887

N 2.822474 -0.914141 -0.784549

N 3.708299 -0.300940 -1.240693

N -2.934634 0.759868 -0.112935

N -4.088811 0.464000 -0.634525

N 2.086646 1.269864 -0.838845

N 3.200205 1.387113 -1.307171

N -2.757278 -1.158601 -1.100732

N -3.979485 -0.722166 -1.247536

B3LYP/6-311++G**(HNO) + LanL2DZ(Mn)

Total electronic energy = -957.443583 Ha

Zero-point correction = 0.148060 Ha

Thermal correction to enthalpy = 0.168962 Ha

Thermal correction to free energy = 0.096173 Ha

H 1.440254 1.713279 1.534463

H -1.439743 1.535219 -1.713548

H 1.439768 -1.715001 -1.534281

H -1.440143 -1.534325 1.714077

H 0.060939 -2.473707 -1.566269

H -0.062154 -1.563800 2.474167

H 0.061878 2.472794 1.566219

H -0.061042 1.567514 -2.472432

Mn 0.000020 -0.000243 -0.000023

O 0.466092 1.599228 1.543819

O -0.465662 1.543986 -1.599080

O 0.465718 -1.600432 -1.542886

O -0.465970 -1.543066 1.600363

N 2.195006 0.000021 -0.000369

N -2.195055 -0.000221 -0.000398

N 2.966080 0.814578 0.699740

N 4.212198 0.503564 0.433497

N -2.966136 0.700174 -0.814705

N -4.212258 0.434073 -0.503529

N 2.966327 -0.814413 -0.700338

N 4.212350 -0.503236 -0.433809

N -2.966360 -0.700214 0.814036

N -4.212392 -0.433417 0.503083

B3LYP/6-311++G**(HNO) + LanL2DZ(Mn)

Total electronic energy = -957.392317 Ha

Zero-point correction = 0.150138 Ha

Thermal correction to enthalpy = 0.170060 Ha

Thermal correction to free energy = 0.101461 Ha

H 1.227852 -1.727410 1.587129

H -1.217209 1.691065 1.622722

H 1.227866 1.727460 -1.587143

H -1.217224 -1.691132 -1.622685

H -0.200989 1.780277 -2.245290

H 0.214634 -2.329639 -1.575191

H -0.200980 -1.780159 2.245312

H 0.214640 2.329591 1.575259

Mn -0.003187 0.000006 -0.000029

O 0.266905 -1.695270 1.407780

O -0.259657 1.492773 1.626540

O 0.266921 1.695299 -1.407780

O -0.259675 -1.492829 -1.626503

N 2.013911 0.000000 -0.000020

N -2.016504 0.000011 -0.000019

N 2.781292 -0.797353 0.723120

N 4.026398 -0.490369 0.448394

N -2.783912 0.805520 0.714792

N -4.028512 0.494634 0.444169

N 2.781289 0.797361 -0.723135

N 4.026375 0.490342 -0.448344

N -2.783935 -0.805507 -0.714794

N -4.028528 -0.494639 -0.444121

B3LYP/6-311++G**(HNO) + LanL2DZ(Mn)

Total electronic energy = -957.435680 Ha

Zero-point correction = 0.146190 Ha

Thermal correction to enthalpy = 0.168149 Ha

Thermal correction to free energy = 0.090854 Ha

H -0.259473 -2.797850 -0.609039

H 1.015286 2.443635 -0.547286

H -2.681673 2.224816 -0.164848

H -0.631879 0.048651 2.855744

H -2.700571 3.650060 -0.811749

H 0.923860 0.186788 2.795302

H 1.322108 -2.482843 -0.609860

H -0.606222 2.568880 -0.542050

Mn 0.139915 -0.069867 0.037399

O 0.423953 -2.116917 -0.656195

O 0.178798 1.964305 -0.611795

O -2.181636 3.068153 -0.248538

O 0.131951 0.029093 2.268474

N -1.976700 -0.458996 -0.057627

N 2.281626 -0.045080 -0.020961

N -2.437753 -1.699188 -0.151169

N -3.744420 -1.625957 -0.199339

N 3.048056 1.036414 -0.029944

N 4.282130 0.630217 -0.197660

N -3.008281 0.375052 -0.049312

N -4.097537 -0.344758 -0.136969

N 3.044518 -1.118535 -0.190632

N 4.279242 -0.699533 -0.297354

B3LYP/6-311++G**(HNO) + LanL2DZ(Mn)

Total electronic energy = -957.398962 Ha

Zero-point correction = 0.148864 Ha

Thermal correction to enthalpy = 0.169408 Ha

Thermal correction to free energy = 0.097869 Ha

H 0.589968 -0.661020 2.744025

H -1.025961 2.302755 0.021410

H 2.613344 2.328233 -0.253586

H -1.168029 -2.467886 -0.601711

H 2.543212 3.825710 0.211815

H 0.452130 -2.599344 -0.562735

H -0.947337 -0.446908 2.744340

H 0.599720 2.513893 -0.014332

Mn -0.158610 -0.111791 -0.055945

O -0.195578 -0.675651 2.186133

O -0.157660 1.882304 0.121177

O 2.083380 3.154689 -0.300882

O -0.314579 -2.019751 -0.699214

N 1.860934 -0.370691 -0.143844

N -2.150325 0.005921 -0.154667

N 2.345796 -1.606984 -0.183909

N 3.648810 -1.519445 -0.203756

N -2.871504 1.116943 -0.095830

N -4.125184 0.765505 -0.195613

N 2.880821 0.475484 -0.141475

N 3.981030 -0.229465 -0.178915

N -2.962623 -1.033786 -0.292419

N -4.181509 -0.565392 -0.316471

B3LYP/6-311++G**(HNO) + LanL2DZ(Mn)

Total electronic energy = -957.433732 Ha

Zero-point correction = 0.148736 Ha

Thermal correction to enthalpy = 0.169056 Ha

Thermal correction to free energy = 0.096795 Ha

H -2.025304 2.858546 0.008923

H 1.450290 -2.278380 0.675045

H -2.025140 -2.858330 0.009019

H 1.450267 2.278145 0.675216

H -1.949008 -4.194384 0.829145

H -0.088891 2.700257 0.870873

H -1.948784 4.194609 0.828924

H -0.088806 -2.700659 0.870678

Mn 0.208752 -0.000103 0.331822

O -1.419162 3.542724 0.360784

O 0.585196 -1.980370 1.005632

O -1.419233 -3.542691 0.360906

O 0.585211 1.980080 1.005878

N -1.839729 0.000022 -0.239690

N 2.205133 0.000029 -0.337569

N -2.595799 1.074832 -0.429027

N -3.798338 0.665106 -0.732085

N 2.968836 -1.081109 -0.435747

N 4.196222 -0.667408 -0.609339

N -2.595769 -1.074747 -0.429388

N -3.798315 -0.664895 -0.732277

N 2.968786 1.081227 -0.435500

N 4.196185 0.667627 -0.609234

B3LYP/6-311++G**(HNO) + LanL2DZ(Mn)

Total electronic energy = -957.400606 Ha

Zero-point correction = 0.150847 Ha

Thermal correction to enthalpy = 0.170030 Ha

Thermal correction to free energy = 0.100963 Ha

H -2.157115 2.932424 -0.098098

H 1.351090 -2.287431 0.032840

H -2.157065 -2.932979 -0.097909

H 1.351021 2.287624 0.032989

H -1.807430 -4.349056 0.474264

H -0.231298 2.718177 0.026047

H -1.807445 4.348323 0.474552

H -0.231122 -2.718196 0.026251

Mn 0.266684 0.000054 0.028146

O -1.519461 3.674312 -0.148657

O 0.431563 -1.972752 0.083604

O -1.519229 -3.674694 -0.148466

O 0.431534 1.972821 0.083535

N -1.780785 0.000075 0.000236

N 2.262992 0.000015 -0.001349

N -2.566012 1.072468 0.002734

N -3.804797 0.665619 0.007663

N 3.032984 -1.079508 -0.014086

N 4.269983 -0.669446 -0.034377

N -2.565915 -1.072370 0.002722

N -3.804741 -0.665611 0.007635

N 3.032996 1.079542 -0.013538

N 4.270010 0.669540 -0.034028

B3LYP/6-311++G**(HNO) + LanL2DZ(Mn)

Total electronic energy = -683.597662 Ha

Zero-point correction = 0.122584 Ha

Thermal correction to enthalpy = 0.139645 Ha

Thermal correction to free energy = 0.076525 Ha

H 1.134589 2.449837 -0.161378

H -0.924430 -3.286006 -0.458663

H -1.812312 1.928501 1.387546

H -0.975781 2.385655 0.147160

H 0.651605 3.735606 -0.921798

H 0.377477 -2.598728 0.099597

Mn -1.091761 -0.401097 0.029957

O 0.457628 3.169050 -0.169528

O -0.605950 -2.590340 0.125729

O -1.687814 1.753413 0.445441

N 1.077330 -0.247120 0.013881

N 1.895512 0.788006 -0.051033

N 3.126219 0.328880 -0.061705

N 1.811120 -1.348693 0.044298

N 3.073166 -0.993975 -0.002227

O -3.283048 -0.347201 -0.277944

H -3.441484 0.606710 -0.362681

H -3.645591 -0.763223 -1.070786

B3LYP/6-311++G**(HNO) + LanL2DZ(Mn)

Total electronic energy = -847.919240 Ha

Zero-point correction = 0.135910 Ha

Thermal correction to enthalpy = 0.156741 Ha

Thermal correction to free energy = 0.084595 Ha

H 0.992852 1.369226 -1.851158

H -1.923520 -1.871922 -1.262984

H 0.941359 -1.260939 1.947635

H -1.577506 2.185136 1.395378

H -0.366299 -1.018945 2.794757

H -0.213945 2.915861 1.211734

H -0.446202 1.592594 -2.453809

H -0.471777 -2.421275 -1.192135

Mn -0.488098 0.211336 0.139764

O 0.055728 1.557415 -1.633339

O -0.991094 -1.610307 -1.212909

O -0.031493 -1.155530 1.902884

O -0.615776 2.082337 1.480411

N 1.691723 -0.105807 -0.057885

N -2.544115 0.519977 0.292761

N 2.460106 0.386718 -1.014661

N 3.691470 0.004768 -0.769763

N -3.348048 -0.249760 -0.159271

N -4.097689 -1.003492 -0.606696

N 2.450806 -0.792696 0.779092

N 3.685541 -0.726057 0.339295

B3LYP/6-311++G**(HNO) + LanL2DZ(Mn)

Total electronic energy = -847.925760 Ha

Zero-point correction = 0.136976 Ha

Thermal correction to enthalpy = 0.157092 Ha

Thermal correction to free energy = 0.084609 Ha

H -1.614162 2.748249 0.036674

H 1.529252 -2.255406 1.649660

H -1.610582 -2.749324 0.037482

H 1.528028 2.258862 1.646516

H -1.882832 -3.865192 1.102164

H -0.008775 2.474491 1.345375

H -1.886411 3.865093 1.100365

H -0.007127 -2.473197 1.346540

Mn 0.756051 0.000723 0.343313

O -1.185001 3.388602 0.646253

O 0.690006 -1.790999 1.558671

O -1.181385 -3.388924 0.647847

O 0.689210 1.793295 1.557279

N -1.270172 -0.000448 -0.430308

N 2.513391 0.001213 -0.454238

N -1.994565 1.071059 -0.719086

N -3.151312 0.661148 -1.176946

N 3.529562 0.000210 -1.087816

N 4.509254 -0.000660 -1.682799

N -1.993088 -1.073025 -0.718935

N -3.150401 -0.664846 -1.176728

B3LYP/6-311++G**(HNO) + LanL2DZ(Mn)

Total electronic energy = -847.918569 Ha

Zero-point correction = 0.136896 Ha

Thermal correction to enthalpy = 0.157060 Ha

Thermal correction to free energy = 0.085214 Ha

H -1.898850 2.790079 -0.286928

H 0.498114 -2.295012 0.545100

H -3.285566 -2.438182 -0.119721

H 1.536890 2.316721 0.619952

H -3.151716 -3.629838 0.868691

H 0.045867 2.918744 0.641115

H -2.015332 4.243806 0.272783

H -1.102647 -2.348518 0.686762

Mn -0.120484 0.257870 0.214670

O -1.393071 3.597989 -0.074241

O -0.293306 -1.800325 0.819876

O -2.646115 -3.028998 0.313071

O 0.621484 2.160849 0.901003

N -1.983106 0.782572 -0.386474

N 1.881628 -0.228763 -0.240421

N 2.362241 -1.465374 -0.279853

N 3.661705 -1.368494 -0.383817

N -2.935491 0.086446 -0.622607

N -3.852955 -0.568376 -0.842177

N 2.889251 0.630595 -0.309480

N 3.987215 -0.074415 -0.402627

B3LYP/6-311++G**(HNO) + LanL2DZ(Mn)

Imaginary frequency = 621.4i cm^-1^

Total electronic energy = -957.401349 Ha

Zero-point correction = 0.143732 Ha

Thermal correction to enthalpy = 0.165739 Ha

Thermal correction to free energy = 0.090673 Ha

H 1.437960 0.661767 -2.214904

H -1.076669 -2.527826 -0.604860

H 1.435634 -0.585757 2.231301

H -1.490715 2.188585 0.588433

H 0.035021 -0.326899 2.902737

H -0.116468 2.866689 0.262606

H 0.048384 0.417173 -2.919637

H 0.402424 -2.919114 -0.317448

Mn -0.023702 -0.024529 -0.007342

O 0.465579 0.722277 -2.107823

O -0.160433 -2.225150 -0.678948

O 0.466924 -0.688310 2.121973

O -0.521145 2.089209 0.661550

N 2.184405 0.030015 0.006139

N -2.138524 -0.147873 -0.050058

N 2.957138 0.316927 -1.027765

N 4.202692 0.253235 -0.620095

N -3.006153 -1.040241 -0.242035

N -4.213287 -0.913785 -0.203398

N 2.954815 -0.211452 1.053454

N 4.201332 -0.074997 0.667085

N -3.166292 1.196917 0.265133

N -4.274756 0.827596 0.191728

B3LYP/6-311++G**(HNO) + LanL2DZ(Mn)

Imaginary frequency = 634.8i cm^-1^

Total electronic energy = -957.400432 Ha

Zero-point correction = 0.144613 Ha

Thermal correction to enthalpy = 0.165827 Ha

Thermal correction to free energy = 0.090932 Ha

H -1.393992 2.976085 0.096571

H 0.570298 -2.614053 1.695588

H -2.595380 -2.455579 -0.022012

H 1.747308 1.712572 1.251045

H -3.028781 -3.573031 0.989465

H 0.287077 2.360142 1.285162

H -1.275259 4.171350 1.103168

H -0.957984 -2.551776 1.251719

Mn 0.242336 -0.222419 0.372420

O -0.770515 3.489116 0.651088

O -0.148585 -2.008358 1.485457

O -2.267471 -3.189855 0.543291

O 0.810189 1.530285 1.443428

N -1.718374 0.185322 -0.395328

N 2.117010 -0.383610 -0.395976

N -2.198858 1.395824 -0.644566

N -3.421040 1.254646 -1.089579

N 2.843405 -1.111254 -1.132829

N 4.023165 -0.918738 -1.355750

N -2.659316 -0.700571 -0.693137

N -3.705932 -0.041847 -1.119510

N 3.247302 0.793222 0.098236

N 4.272549 0.522041 -0.406891

B3LYP/6-311++G**(HNO) + LanL2DZ(Mn)

Imaginary frequency = 620.3i cm^-1^

Total electronic energy = -957.393379 Ha

Zero-point correction = 0.144427 Ha

Thermal correction to enthalpy = 0.166040 Ha

Thermal correction to free energy = 0.090732 Ha

H 2.159757 -2.969471 0.106326

H -1.562111 2.232620 0.655306

H 1.982698 3.144951 0.031588

H -1.416124 -2.311625 0.613679

H 1.736297 4.400463 0.912888

H 0.122151 -2.705518 0.858596

H 1.959801 -4.242048 0.988499

H -0.052173 2.716570 0.887516

Mn -0.205604 0.002830 0.292720

O 1.494285 -3.588423 0.458334

O -0.689586 1.972687 1.000593

O 1.293630 3.690008 0.438603

O -0.562836 -1.995970 0.957539

N 1.778574 0.086338 -0.241203

N -2.226257 -0.040288 -0.344988

N 2.917558 -1.220437 -0.433240

N 3.947749 -0.739562 -0.706216

N -3.017245 1.022465 -0.415581

N -4.237129 0.582634 -0.585661

N 2.548659 1.055756 -0.478631

N 3.731483 1.002587 -0.756425

N -2.963987 -1.136643 -0.457045

N -4.204283 -0.750435 -0.611431

The xyz coordinates of geometries in Fig. 14:

­­­___________________________________________________________

B3LYP/6-311++G**

Total electronic energy = -1229.567837 Ha

Zero-point correction = 0.130718 Ha

Thermal correction to enthalpy = 0.146140 Ha

Thermal correction to free energy = 0.081872 Ha

N 5.009421 1.318011 -0.665441

N 4.123169 0.435263 -1.078231

N 3.579329 -0.107283 0.000095

N 4.124199 0.434184 1.078491

N 5.010041 1.317329 0.665749

C -4.086293 0.561835 0.000396

C -2.824104 1.148675 0.000031

C -1.662263 0.387114 -0.000167

C -1.770079 -1.008750 -0.000010

C -3.030172 -1.612295 0.000340

C -4.179530 -0.828768 0.000553

H -4.973556 1.182393 0.000550

H -0.690540 0.861287 -0.000441

H -3.083447 -2.693601 0.000430

H -5.157226 -1.297798 0.000839

Cl -2.701619 2.908474 -0.000180

C -0.567436 -1.917326 -0.000247

O -0.640386 -3.121561 -0.000527

O 0.551067 -1.185098 -0.000084

O 1.737101 -1.995553 -0.000395

H 2.446201 -1.244100 -0.000287

RI-B2KPLYP/ma-def2-TZVP

Total electronic energy = -1228.782807 Ha

Zero-point correction = 0.133035 Ha

Thermal correction to enthalpy = 0.145523 Ha

Thermal correction to free energy = 0.093972Ha

N 4.911125 1.346208 -0.661976

N 4.056033 0.437941 -1.072733

N 3.532801 -0.119575 -0.000094

N 4.057141 0.437048 1.072462

N 4.911817 1.345651 0.661576

C -4.039933 0.568601 0.000220

C -2.780324 1.147138 0.000275

C -1.629455 0.378719 0.000247

C -1.748578 -1.008066 0.000166

C -3.004477 -1.602788 0.000113

C -4.143402 -0.814543 0.000139

H -4.918375 1.194731 0.000238

H -0.658237 0.845812 0.000282

H -3.062332 -2.679761 0.000046

H -5.120650 -1.274248 0.000093

Cl -2.647568 2.877175 0.000375

C -0.557762 -1.916527 0.000113

O -0.636391 -3.115805 -0.000056

O 0.555921 -1.193995 0.000134

O 1.713063 -2.024140 -0.000053

H 2.423462 -1.287143 -0.000104

B3LYP/6-311++G**

Imaginary frequency = 306.5i cm^-1^

Total electronic energy = -1229.538974 Ha

Zero-point correction = 0.129157 Ha

Thermal correction to enthalpy = 0.144206 Ha

Thermal correction to free energy = 0.083336 Ha

N -6.056851 -0.417495 -0.667108

N -4.818418 -0.243517 -1.081922

N -4.073267 -0.127111 -0.000083

N -4.818313 -0.242918 1.081897

N -6.056787 -0.417119 0.667301

C 4.484610 0.765989 -0.000065

C 3.798029 -0.444334 0.000023

C 2.410544 -0.498917 0.000042

C 1.686152 0.697269 -0.000021

C 2.359293 1.921004 -0.000104

C 3.750672 1.951615 -0.000131

H 5.567294 0.777660 -0.000082

H 1.886595 -1.444822 0.000101

H 1.771956 2.830485 -0.000142

H 4.273942 2.901574 -0.000202

Cl 4.722727 -1.951340 0.000113

C 0.172574 0.703122 0.000005

O -0.455773 1.757155 0.000118

O -0.346956 -0.487634 -0.000107

O -2.061983 -0.384930 -0.000096

H -2.074232 0.593791 0.000008

RI-B2KPLYP/ma-def2-TZVP

Imaginary frequency = 413.8i cm^-1^

Total electronic energy = -1228.745767Ha

Zero-point correction = 0.131972 Ha

Thermal correction to enthalpy = 0.145610 Ha

Thermal correction to free energy = 0.091270 Ha

N -5.960903 -0.354555 -0.662809

N -4.721450 -0.221055 -1.077769

N -3.977724 -0.128335 -0.000393

N -4.720985 -0.217125 1.077635

N -5.960618 -0.352128 0.663710

C 4.435504 0.775940 0.000059

C 3.768099 -0.438493 -0.000162

C 2.386747 -0.506804 -0.000233

C 1.654217 0.674496 -0.000069

C 2.307272 1.900936 0.000152

C 3.692076 1.947697 0.000218

H 5.514020 0.798584 0.000107

H 1.875511 -1.455611 -0.000415

H 1.709394 2.798574 0.000274

H 4.202259 2.899773 0.000402

Cl 4.691762 -1.910714 -0.000367

C 0.151355 0.658135 -0.000116

O -0.482639 1.705625 -0.000256

O -0.353042 -0.530235 0.000013

O -2.067212 -0.390481 -0.000272

H -2.021836 0.585304 -0.000165

B3LYP/6-311++G**

Total electronic energy = -1229.617798 Ha

Zero-point correction = 0.131097 Ha

Thermal correction to enthalpy = 0.146040 Ha

Thermal correction to free energy = 0.083191 Ha

N -5.297975 1.230009 -0.514814

N -4.086419 0.737278 -0.681026

N -4.079951 -0.395499 0.017270

N -5.251889 -0.614646 0.604892

N -6.001161 0.412840 0.256732

C 3.912782 1.512725 0.122731

C 3.927344 0.126442 -0.000483

C 2.755851 -0.614765 -0.044385

C 1.527398 0.046872 0.039081

C 1.493415 1.438360 0.163230

C 2.682261 2.163085 0.203636

H 4.842312 2.067177 0.153927

H 2.765308 -1.692451 -0.141741

H 0.533585 1.933783 0.224463

H 2.656645 3.243216 0.298228

Cl 5.486065 -0.706701 -0.103645

C 0.262254 -0.783302 -0.011736

O -0.818753 -0.047325 0.108476

O 0.306332 -1.991642 -0.149353

O -3.073833 -1.197821 0.108801

H -1.716989 -0.569889 0.069869

The xyz coordinates of geometries in Fig. 15:

­­­___________________________________________________________

B3LYP/6-311++G**

Total electronic energy = -349.016464 Ha

Zero-point correction = 0.025767 Ha

Thermal correction to enthalpy = 0.030559 Ha

Thermal correction to free energy = -0.000820 Ha

N 0.000000 1.102880 -0.096086

N 0.000000 0.000000 0.667721

N 0.000000 -1.102880 -0.096086

N 0.000000 -0.661014 -1.342988

N 0.000000 0.661014 -1.342988

O 0.000000 0.000000 1.934124

RI-B2KPLYP/ma-def2-TZVP

Total electronic energy = -348.758142 Ha

Zero-point correction = 0.026470 Ha

Thermal correction to enthalpy = 0.031199 Ha

Thermal correction to free energy = 0.000312 Ha

N 0.093546 1.095536 0.000080

N -0.662791 -0.000002 -0.000081

N 0.093551 -1.095536 -0.000023

N 1.337061 -0.657947 0.000091

N 1.337058 0.657953 -0.000082

O -1.922120 -0.000004 0.000022

B3LYP/6-311++G**

Imaginary frequency = 483.3i cm^-1^

Total electronic energy = -348.971742 Ha

Zero-point correction = 0.020819 Ha

Thermal correction to enthalpy = 0.026595 Ha

Thermal correction to free energy = -0.007542 Ha

N -0.135133 1.055210 0.000000

N -0.805871 -0.039260 0.000000

N 0.614691 -1.267468 0.000000

N 1.556929 -0.579663 0.000002

N 1.111308 1.106092 0.000001

O -2.049183 -0.240547 -0.000003

RI-B2KPLYP/ma-def2-TZVP

Imaginary frequency = 558.6i cm^-1^

Total electronic energy = -348.708469 Ha

Zero-point correction = 0.021343 Ha

Thermal correction to enthalpy = 0.026943 Ha

Thermal correction to free energy = -0.005716 Ha

N 0.126407 1.055424 0.000002

N 0.802521 -0.044363 0.000004

N -0.594137 -1.246424 -0.000000

N -1.541593 -0.565134 -0.000002

N -1.118314 1.087498 -0.000001

O 2.032370 -0.252673 0.000001

B3LYP/6-311++G**

Total electronic energy = -349.011492 Ha

Zero-point correction = 0.018539 Ha

Thermal correction to enthalpy = 0.027200 Ha

Thermal correction to free energy = -0.015281 Ha

N -0.679569 0.747094 0.076424

N -1.156508 -0.330971 0.444750

N 1.887044 -0.981445 0.133820

N 2.389777 -0.059115 -0.191682

N -0.105835 1.751997 -0.090192

O -2.043045 -0.986615 -0.326480

The xyz coordinates of geometries in Fig. 16:

­­­___________________________________________________________

B3LYP/6-311++G**

Total electronic energy = -1304.760413 Ha

Zero-point correction = 0.135071 Ha

Thermal correction to enthalpy = 0.151242 Ha

Thermal correction to free energy = 0.086076 Ha

N -4.547386 -1.721069 0.379130

N -3.244925 -1.889042 0.551928

N -2.611100 -0.792945 0.181791

N -3.571367 0.041132 -0.214568

N -4.774710 -0.513196 -0.100351

C 3.942327 -0.983901 -0.034802

C 2.591020 -1.317919 -0.068666

C 1.594528 -0.350010 -0.024599

C 1.972493 0.996676 0.056302

C 3.324126 1.347836 0.096851

C 4.302094 0.359954 0.048456

H 4.693809 -1.762661 -0.070989

H 0.547941 -0.627904 -0.046177

H 3.583536 2.396610 0.168014

H 5.351721 0.631127 0.077114

Cl 2.139069 -3.018612 -0.168856

C 0.964397 2.114105 0.116136

O 1.245268 3.267400 0.334724

O -0.254736 1.617897 -0.114307

O -1.288341 2.610080 -0.019182

H -2.084147 2.014348 -0.246394

O -3.371756 1.228409 -0.668059

RI-B2KPLYP/ma-def2-TZVP

Total electronic energy = -1303.925340 Ha

Zero-point correction = 0.137687Ha

Thermal correction to enthalpy = 0.150896 Ha

Thermal correction to free energy = 0.097544 Ha

N -3.927118 -1.722129 0.801128

N -2.628299 -1.814176 0.602364

N -2.204747 -0.725829 -0.002355

N -3.285980 0.022287 -0.155550

N -4.359031 -0.571820 0.332295

C 3.713393 -1.041423 -0.022476

C 2.366319 -1.313128 -0.208090

C 1.410332 -0.311245 -0.177535

C 1.831414 0.997855 0.045975

C 3.175955 1.286795 0.244085

C 4.112872 0.267433 0.204420

H 4.431409 -1.845976 -0.051902

H 0.361127 -0.538792 -0.304964

H 3.463498 2.309677 0.430570

H 5.159919 0.486240 0.353688

Cl 1.873001 -2.952943 -0.480548

C 0.859449 2.133026 0.107682

O 1.092604 3.199226 0.611730

O -0.264542 1.762664 -0.497009

O -1.294368 2.739712 -0.375477

O -3.304825 1.168768 -0.720770

H -2.078381 2.110379 -0.504261

B3LYP/6-311++G**

Total electronic energy = -1304.794036 Ha

Zero-point correction = 0.134921 Ha

Thermal correction to enthalpy = 0.150844 Ha

Thermal correction to free energy = 0.086233 Ha

N -5.303454 0.437084 -1.312160

N -4.334973 -0.447197 -1.388476

N -3.783509 -0.508977 -0.180164

N -4.451316 0.364735 0.643086

N -5.397939 0.949165 -0.092440

C 4.142014 1.548199 -0.129741

C 4.190735 0.159259 -0.053790

C 3.037718 -0.607634 0.023386

C 1.793979 0.030392 0.024211

C 1.725799 1.424018 -0.051601

C 2.896635 2.174588 -0.127413

H 5.057537 2.123251 -0.188700

H 3.074024 -1.687422 0.083424

H 0.755460 1.902100 -0.047837

H 2.844918 3.256296 -0.184783

Cl 5.767785 -0.643091 -0.056022

C 0.553252 -0.828674 0.110772

O -0.551649 -0.111030 0.084138

O 0.622113 -2.039039 0.195251

O -2.831082 -1.268969 0.209380

H -1.416231 -0.660736 0.150733

O -4.178194 0.548351 1.845689

B3LYP/6-311++G**

Total electronic energy = -1304.804268 Ha

Zero-point correction = 0.135670 Ha

Thermal correction to enthalpy = 0.151423 Ha

Thermal correction to free energy = 0.087238 Ha

N 4.875126 1.044394 0.802277

N 3.673782 0.492391 0.900891

N 3.651699 -0.515076 0.049640

N 4.796793 -0.665927 -0.618121

N 5.543201 0.332026 -0.114823

C -4.322983 1.552457 -0.134257

C -4.364110 0.166409 -0.013321

C -3.206478 -0.596248 0.030213

C -1.966050 0.042996 -0.051130

C -1.905560 1.433799 -0.173015

C -3.080813 2.180225 -0.213189

H -5.241917 2.124247 -0.165216

H -3.236741 -1.673669 0.125691

H -0.937490 1.912884 -0.232709

H -3.035118 3.259743 -0.306018

Cl -5.936873 -0.637592 0.087144

C -0.720023 -0.811170 -0.000213

O 0.379315 -0.094263 -0.116573

O -0.780748 -2.017772 0.133171

O 2.647280 -1.294938 -0.127409

H 1.247093 -0.639084 -0.075930

O 6.722017 0.560656 -0.468795

B3LYP/6-311++G**

Total electronic energy = -1304.959515 Ha

Zero-point correction = 0.123890 Ha

Thermal correction to enthalpy = 0.146148 Ha

Thermal correction to free energy = 0.058397 Ha

N 7.255866 -1.070934 0.134973

N 6.936561 -2.100579 -0.061797

N 3.941643 -0.218560 -0.027086

N 2.290911 3.914143 0.540069

N 2.292397 3.912866 -0.555119

C -4.193293 -1.717242 0.005307

C -4.113632 -0.327945 0.005543

C -2.895384 0.335349 0.003256

C -1.713086 -0.410180 0.000785

C -1.773604 -1.806175 0.000517

C -3.007973 -2.452023 0.002719

H -5.158138 -2.208920 0.007074

H -2.831395 1.415759 0.003260

H -0.845869 -2.363382 -0.001564

H -3.054573 -3.535883 0.002388

Cl -5.616360 0.613488 0.008857

C -0.385204 0.331823 -0.001780

O 0.635341 -0.475960 -0.001356

O -0.366078 1.553970 -0.003949

O 2.900515 0.541152 -0.004327

O 3.730299 -1.430467 -0.049386

H 1.638893 -0.026624 -0.004926

B3LYP/6-311++G**

Imaginary frequency = 341.2i cm^-1^

Total electronic energy = -1304.723404 Ha

Zero-point correction = 0.133456 Ha

Thermal correction to enthalpy = 0.149120 Ha

Thermal correction to free energy = 0.087120 Ha

N -5.517544 -1.048294 -0.801792

N -4.436660 -0.547948 -1.339263

N -3.796107 0.151649 -0.414990

N -4.502496 0.003112 0.737806

N -5.592159 -0.721410 0.490110

C 4.737121 0.680473 0.046773

C 4.005807 -0.502955 0.072963

C 2.619448 -0.509324 -0.004481

C 1.941521 0.708715 -0.112634

C 2.659795 1.906031 -0.140113

C 4.049342 1.888858 -0.060508

H 5.817780 0.654587 0.109041

H 2.058534 -1.433678 0.017713

H 2.107278 2.833343 -0.223655

H 4.607390 2.818728 -0.081503

Cl 4.872341 -2.039551 0.209043

C 0.427679 0.759120 -0.201955

O -0.150858 1.843348 -0.304214

O -0.140719 -0.396787 -0.160425

O -1.938794 -0.145354 -0.287176

H -1.789205 0.825695 -0.292493

O -4.189764 0.514846 1.828539

B3LYP/6-311++G**

Imaginary frequency = 444.9i cm^-1^

Total electronic energy = -1304.697560 Ha

Zero-point correction = 0.129989 Ha

Thermal correction to enthalpy = 0.146490 Ha

Thermal correction to free energy = 0.083123 Ha

N -4.553005 -0.296242 -1.547435

N -4.275214 0.636337 -0.786562

N -4.174820 0.098444 0.566952

N -4.498305 -1.314851 0.431129

N -4.695515 -1.545750 -0.763959

C 4.607704 0.797511 -0.175748

C 3.950548 -0.416804 -0.005211

C 2.566795 -0.498529 0.073854

C 1.810952 0.673839 -0.020083

C 2.454110 1.901561 -0.191087

C 3.843110 1.960480 -0.268637

H 5.688530 0.830056 -0.234009

H 2.061530 -1.445856 0.205770

H 1.841816 2.791924 -0.260326

H 4.341535 2.914954 -0.402169

Cl 4.915236 -1.899230 0.114080

C 0.285327 0.633630 0.061319

O -0.340057 1.701112 -0.013914

O -0.206237 -0.533434 0.202334

O -2.209960 -0.272370 0.286179

H -1.971304 0.676503 0.188159

O -4.400546 0.750146 1.577390

B3LYP/6-311++G**

Imaginary frequency = 307.6i cm^-1^

Total electronic energy = -1304.728205 Ha

Zero-point correction = 0.133593 Ha

Thermal correction to enthalpy = 0.149315 Ha

Thermal correction to free energy = 0.086748 Ha

N 5.551840 -0.371396 0.991374

N 4.303682 -0.098944 1.302547

N 3.637355 0.100230 0.192710

N 4.392503 -0.072317 -0.874614

N 5.595866 -0.361350 -0.363617

C -4.922314 0.687094 -0.101220

C -4.200341 -0.500865 -0.040147

C -2.812544 -0.512489 0.004491

C -2.124401 0.704645 -0.012863

C -2.833378 1.906325 -0.073974

C -4.224414 1.894190 -0.117858

H -6.004315 0.665444 -0.134865

H -2.260031 -1.440863 0.052080

H -2.273625 2.832951 -0.086292

H -4.775533 2.827061 -0.165608

Cl -5.079251 -2.034936 -0.018661

C -0.611264 0.752949 0.033921

O -0.016972 1.830144 0.015802

O -0.053472 -0.413707 0.091737

O 1.699611 -0.211159 0.137990

H 1.614672 0.764999 0.099431

O 6.626998 -0.582822 -1.038079

RI-B2KPLYP/ma-def2-TZVP

Imaginary frequency = 417.7i cm^-1^

Total electronic energy = -1303.881944 Ha

Zero-point correction = 0.136625 Ha

Thermal correction to enthalpy = 0.150899 Ha

Thermal correction to free energy = 0.095227 Ha

N 5.460440 -0.172070 1.011984

N 4.193393 0.031482 1.284355

N 3.536286 0.046463 0.158211

N 4.317889 -0.174300 -0.872878

N 5.525638 -0.302164 -0.323917

C -4.871087 0.716260 -0.072499

C -4.179811 -0.484330 -0.038505

C -2.797530 -0.524583 -0.014535

C -2.088891 0.671065 -0.025638

C -2.766121 1.883883 -0.060705

C -4.151428 1.902577 -0.083894

H -5.949703 0.717022 -0.090068

H -2.266838 -1.462292 0.013388

H -2.186656 2.793431 -0.069062

H -4.680676 2.843754 -0.111450

Cl -5.074622 -1.973952 -0.025098

C -0.586121 0.682713 0.002515

O 0.025466 1.746279 -0.005369

O -0.053160 -0.489599 0.035414

O 1.684034 -0.274287 0.086206

H 1.564565 0.696809 0.055397

O 6.575603 -0.508978 -0.959566

The xyz coordinates of geometries in Fig. 17:

­­­___________________________________________________________

B3LYP/6-311++G**

Total electronic energy = -424.206692 Ha

Zero-point correction = 0.030019 Ha

Thermal correction to enthalpy = 0.035575 Ha

Thermal correction to free energy = 0.001500 Ha

N 0.666182 1.161972 -0.000056

N -0.666223 1.161948 0.000068

N -1.046540 -0.112459 0.000103

N 0.000098 -0.957406 0.000132

N 1.046510 -0.112667 0.000001

O -2.246444 -0.499421 -0.000145

O 2.246420 -0.499294 -0.000072

RI-B2KPLYP/ma-def2-TZVP

Total electronic energy = -423.896590 Ha

Zero-point correction = 0.031024 Ha

Thermal correction to enthalpy = 0.036475 Ha

Thermal correction to free energy = 0.003626 Ha

N 0.664266 1.155908 0.000031

N -0.664288 1.155878 0.000037

N -1.039598 -0.110787 -0.000148

N 0.000021 -0.948801 0.000024

N 1.039587 -0.110804 -0.000113

O -2.230830 -0.499395 0.000113

O 2.230845 -0.499326 0.000087

B3LYP/6-311++G**

Imaginary frequency = 671.4i cm^-1^

Total electronic energy = -424.165260 Ha

Zero-point correction = 0.025424 Ha

Thermal correction to enthalpy = 0.031650 Ha

Thermal correction to free energy = -0.003775 Ha

N 0.597360 1.357435 -0.012817

N -0.597936 1.356801 0.012732

N -1.073064 -0.221972 0.204722

N -0.000106 -0.952477 -0.000579

N 1.073432 -0.221887 -0.204851

O -2.238542 -0.576674 -0.088542

O 2.238817 -0.576489 0.089236

RI-B2KPLYP/ma-def2-TZVP

Imaginary frequency = 653.3i cm^-1^

Total electronic energy = -423.847107 Ha

Zero-point correction = 0.026145 Ha

Thermal correction to enthalpy = 0.032220 Ha

Thermal correction to free energy = -0.001882 Ha

N 0.593718 1.351780 -0.023063

N -0.593988 1.351663 0.023074

N -1.072738 -0.225304 0.212821

N 0.000090 -0.949881 -0.000028

N 1.072776 -0.225087 -0.212857

O -2.229361 -0.569434 -0.095220

O 2.229465 -0.568999 0.095174

B3LYP/6-311++G**

Total electronic energy = -424.266475 Ha

Zero-point correction = 0.023033 Ha

Thermal correction to enthalpy = 0.032822 Ha

Thermal correction to free energy = -0.016636 Ha

N -2.957680 -0.143211 0.548422

N -2.964578 -0.154287 -0.546764

N 0.814196 -1.050736 -0.001501

N 1.513059 0.095572 0.001322

N 0.601154 1.080548 -0.002544

O 1.523716 -2.062176 0.001460

O 1.095902 2.212774 -0.000527
