# Supplementary material for: Roads to pentazolate anion: a theoretical insight
Source: R Soc Open Sci. 2018 May 23;5(5):172269. doi: 10.1098/rsos.172269 (PMC5990749; doi:10.1098/rsos.172269)
Supplement: Computational test [file rsos172269supp2.docx]

**Electronic supplementary material of “Roads to pentazolate anion: A theoretical insight”**

**Table S1.** The selected activation dissociation energy barriers in kcal mol^-1^ excluding ZPCs with and without the empirical dispersion corrections.

| Transition States | B3LYP | B3LYP-D^α^ | RI-B2KPLYP | CCSD(T) |
| --- | --- | --- | --- | --- |
| 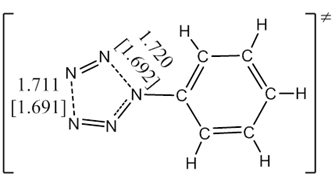 | 19.8 | 20.0 | 23.0 | 22.1 |
| 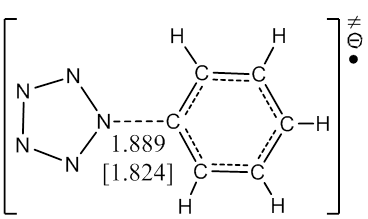 | 27.6 | 28.4 | 25.1 | 25.5 |
| 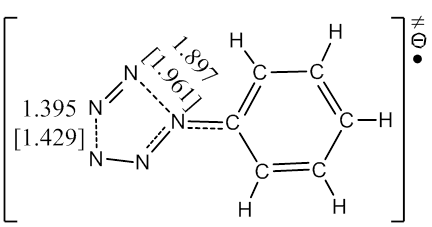 | 21.4 | 21.7 | 25.9 | 26.7 |
| 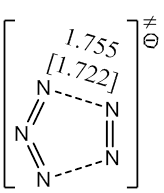 | 26.3 | 26.2 | 28.6 | 28.7 |
| 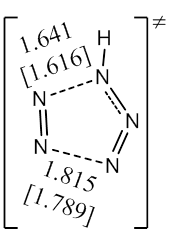 | 19.1 | 19.1 | 22.6 | 21.2 |
| 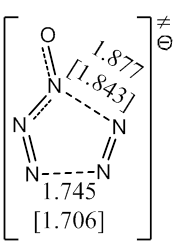 | 28.1 | 28.1 | 31.2 | 33.3 |
| 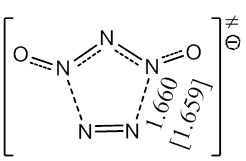 | 26.0 | 26.1 | 31.1 | 32.7 |

^α^ D3 version of Grimme’s dispersion [*J. Chem. Phys.*, 2010, **132**, 154104.] with Becke-Johnson damping [*J. Comp. Chem.*, 2011, **32**, 1456.].
