# Supplementary material for: Roads to pentazolate anion: a theoretical insight
Source: R Soc Open Sci. 2018 May 23;5(5):172269. doi: 10.1098/rsos.172269 (PMC5990749; doi:10.1098/rsos.172269)
Supplement: Supplementary solvent effect [file rsos172269supp3.docx]

**Electronic supplementary material of “Roads to pentazolate anion: A theoretical insight”**

**Table S2.** The correlative activation dissociation energy barriers for radical anion mechanism in kcal mol^-1^ excluding ZPCs.

| Transition States | ΔE^≠^(B3LYP) | | ΔE^≠^(RI-B2KPLYP) | ΔE^≠^[CCSD(T)] |
| --- | --- | --- | --- | --- |
|  | Gas | THF | Gas | Gas |
| 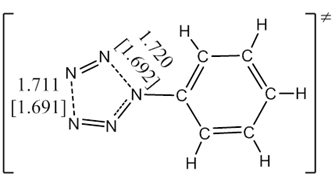 | 19.8 | 21.4 | 23.0 | 22.1 |
| 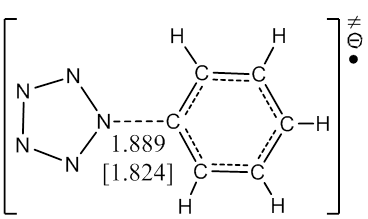 | 27.6 | 27.3 | 25.1 | 25.5 |
| 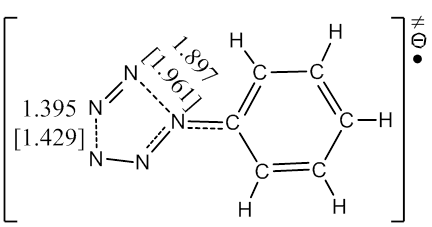 | 21.4 | 21.2 | 25.9 | 26.7 |
| 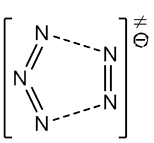 | 26.3 | 27.1 | 28.6 | 28.7 |
