# Supplementary material for: Roads to pentazolate anion: a theoretical insight
Source: R Soc Open Sci. 2018 May 23;5(5):172269. doi: 10.1098/rsos.172269 (PMC5990749; doi:10.1098/rsos.172269)
Supplement: Supplementary potential energy surfaces [file rsos172269supp4.docx]

**Electronic supplementary material of “Roads to pentazolate anion: A theoretical insight”**





**Fig. S1.** The PES scanning result for *m*-CPBA calculated at the B3LYP/6-311++G** level. The activation energy barriers (sum of total electronic energy and ZPC at front, enthalpy of 298 K at middle and free energy of 298 K at back) are in kcal mol^-1^. The crucial bond lengths are labeled in Å.





**Fig. S2.** The PES scanning result for oxo-PZA calculated at the B3LYP/6-311++G** level. The activation energy barriers (sum of total electronic energy and ZPC at front, enthalpy of 298 K at middle and free energy of 298 K at back) are in kcal mol^-1^. The crucial bond lengths are labeled in Å.
