# Supplementary material for: Roads to pentazolate anion: a theoretical insight
Source: R Soc Open Sci. 2018 May 23;5(5):172269. doi: 10.1098/rsos.172269 (PMC5990749; doi:10.1098/rsos.172269)
Supplement: Crystallographic information file [file rsos172269supp5.docx]

**Electronic supplementary material of “Roads to pentazolate anion: A theoretical insight”**

The crystallographic information file for the transition state of the solid (N_5_)_6_(H_3_O)_3_(NH_4_)_4_Cl dissociation:

data_ts

_symmetry_space_group_name_H-M 'P1'

_symmetry_Int_Tables_number 1

_symmetry_cell_setting triclinic

loop_

_symmetry_equiv_pos_as_xyz

x,y,z

_cell_length_a 12.7054

_cell_length_b 12.7054

_cell_length_c 12.7054

_cell_angle_alpha 60.0000

_cell_angle_beta 60.0000

_cell_angle_gamma 60.0000

loop_

_atom_site_label

_atom_site_type_symbol

_atom_site_fract_x

_atom_site_fract_y

_atom_site_fract_z

_atom_site_U_iso_or_equiv

_atom_site_adp_type

_atom_site_occupancy

Cl1 Cl 0.62873 0.62540 0.61078 0.03720 Uani 1.00

Cl2 Cl 0.36386 0.38586 0.38328 0.03720 Uani 1.00

O3 O 0.49767 0.46150 0.04379 0.08200 Uani 1.00

O4 O 0.52067 0.45802 0.53419 0.08200 Uani 1.00

O5 O 0.03007 0.48555 0.50201 0.08200 Uani 1.00

O6 O 0.51839 -0.04320 0.50904 0.08200 Uani 1.00

N7 N 0.60370 0.60920 0.09890 0.03810 Uani 1.00

N8 N 0.66908 0.68380 -0.00318 0.04473 Uani 1.00

H9 H 0.69000 0.70825 0.82866 0.05000 Uiso 1.00

N10 N 0.62153 0.59095 0.20199 0.03810 Uani 1.00

N11 N 0.69798 0.65447 0.16348 0.04473 Uani 1.00

H12 H 0.73281 0.69161 0.26390 0.05000 Uiso 1.00

N13 N 0.09916 0.22556 0.58703 0.03810 Uani 1.00

N14 N 0.00265 0.17867 0.66599 0.04473 Uani 1.00

H15 H 0.83202 0.27500 0.69954 0.05000 Uiso 1.00

N16 N 0.20745 0.12858 0.56742 0.03810 Uani 1.00

N17 N 0.17703 0.02159 0.63487 0.04473 Uani 1.00

H18 H 0.27773 0.84645 0.65979 0.05000 Uiso 1.00

N19 N 0.06578 0.59848 0.58966 0.03810 Uani 1.00

N20 N 0.00473 0.63148 0.68814 0.04473 Uani 1.00

H21 H 0.81649 0.70141 0.69387 0.05000 Uiso 1.00

N22 N 0.21235 0.61457 0.54089 0.03810 Uani 1.00

N23 N 0.20358 0.65230 0.61320 0.04473 Uani 1.00

H24 H 0.28630 0.71145 0.65394 0.05000 Uiso 1.00

N25 N 0.59718 0.11193 0.20506 0.03810 Uani 1.00

N26 N 0.67573 0.00822 0.17122 0.04473 Uani 1.00

H27 H 0.72541 0.82497 0.27088 0.05000 Uiso 1.00

N28 N 0.58579 0.21053 0.09799 0.03810 Uani 1.00

N29 N 0.65696 0.16872 -0.00158 0.04473 Uani 1.00

H30 H 0.69312 0.28065 0.82559 0.05000 Uiso 1.00

N31 N 0.57672 0.11852 0.59140 0.03810 Uani 1.00

N32 N 0.64089 0.01027 0.66305 0.04473 Uani 1.00

H33 H 0.68560 0.83757 0.69350 0.05000 Uiso 1.00

N34 N 0.58648 0.21341 0.59779 0.03810 Uani 1.00

N35 N 0.65601 0.16455 0.67275 0.04473 Uani 1.00

H36 H 0.70269 0.27119 0.69524 0.05000 Uiso 1.00

N37 N 0.24802 0.59830 0.05387 0.03810 Uani 1.00

N38 N 0.19230 0.66912 -0.03554 0.04473 Uani 1.00

H39 H 0.27829 0.71119 0.79080 0.05000 Uiso 1.00

N40 N 0.15722 0.59448 0.16869 0.03810 Uani 1.00

N41 N 0.04494 0.66333 0.14996 0.04473 Uani 1.00

H42 H 0.85892 0.69305 0.27639 0.05000 Uiso 1.00

N43 N 0.40192 0.41786 0.77999 0.03810 Uani 1.00

N44 N 0.32069 0.35542 0.82749 0.04473 Uani 1.00

H45 H 0.27266 0.31017 0.73906 0.05000 Uiso 1.00

N46 N 0.41791 0.41477 0.87647 0.03810 Uani 1.00

N47 N 0.34557 0.35057 0.98420 0.04473 Uani 1.00

H48 H 0.30287 0.31776 0.16666 0.05000 Uiso 1.00

N49 N 0.88674 0.79809 0.40320 0.03810 Uani 1.00

N50 N 0.98937 0.83428 0.34135 0.04473 Uani 1.00

H51 H 0.19088 0.72762 0.27866 0.05000 Uiso 1.00

N52 N 0.78309 0.90326 0.41130 0.03810 Uani 1.00

N53 N 0.82124 1.00413 0.35490 0.04473 Uani 1.00

H54 H 0.70056 0.18403 0.31630 0.05000 Uiso 1.00

N55 N 0.84845 0.43099 0.79983 0.03810 Uani 1.00

N56 N 0.95453 0.35482 0.83163 0.04473 Uani 1.00

H57 H 0.14274 0.30940 0.73326 0.05000 Uiso 1.00

N58 N 0.74902 0.42706 0.90829 0.03810 Uani 1.00

N59 N 0.79412 0.34837 1.00623 0.04473 Uani 1.00

H60 H 0.70370 0.31332 0.17908 0.05000 Uiso 1.00

N61 N 0.42565 0.91380 0.38621 0.03810 Uani 1.00

N62 N 0.33828 1.01153 0.33403 0.04473 Uani 1.00

H63 H 0.29881 0.18975 0.30393 0.05000 Uiso 1.00

N64 N 0.44185 0.80737 0.37537 0.03810 Uani 1.00

N65 N 0.36378 0.83966 0.31643 0.04473 Uani 1.00

H66 H 0.32591 0.72743 0.27434 0.05000 Uiso 1.00

N67 N 0.40939 0.90513 0.75764 0.03810 Uani 1.00

N68 N 0.33220 0.99983 0.80720 0.04473 Uani 1.00

H69 H 0.27864 0.17762 0.73061 0.05000 Uiso 1.00

N70 N 0.42485 0.79387 0.85310 0.03810 Uani 1.00

N71 N 0.35671 0.82083 0.96168 0.04473 Uani 1.00

H72 H 0.32384 0.72253 0.14437 0.05000 Uiso 1.00

N73 N 0.78261 0.41377 0.41476 0.03810 Uani 1.00

N74 N 0.82256 0.33291 0.35557 0.04473 Uani 1.00

H75 H 0.70427 0.31640 0.31000 0.05000 Uiso 1.00

N76 N 0.88494 0.42507 0.40688 0.03810 Uani 1.00

N77 N 0.98837 0.35130 0.34215 0.04473 Uani 1.00

H78 H 0.16848 0.32398 0.29781 0.05000 Uiso 1.00

N79 N 0.72726 0.71165 0.03701 0.04433 Uani 1.00

N80 N 0.05052 0.05216 0.69604 0.04433 Uani 1.00

N81 N 0.03668 0.66862 0.73521 0.04433 Uani 1.00

N82 N 0.71269 0.04341 0.04369 0.04433 Uani 1.00

N83 N 0.68981 0.03827 0.71358 0.04433 Uani 1.00

N84 N 0.06692 0.70944 0.02375 0.04433 Uani 1.00

N85 N 0.28556 0.31385 0.95421 0.04433 Uani 1.00

N86 N 0.94847 0.96125 0.31189 0.04433 Uani 1.00

N87 N 0.92102 0.30363 0.95922 0.04433 Uani 1.00

N88 N 0.30005 0.96565 0.29112 0.04433 Uani 1.00

N89 N 0.29936 0.94787 0.93357 0.04433 Uani 1.00

N90 N 0.94971 0.29446 0.31068 0.04433 Uani 1.00

O91 O 0.86746 0.85383 0.93624 0.07100 Uani 1.00

O92 O 0.09420 0.10207 0.21464 0.07100 Uani 1.00

N93 N 0.71759 0.73622 0.72845 0.03340 Uani 1.00

H94 H 0.68189 0.69861 0.70157 0.05000 Uiso 1.00

N95 N 0.75911 0.72470 0.30521 0.03340 Uani 1.00

H96 H 0.71922 0.69040 0.40590 0.05000 Uiso 1.00

N97 N 0.73099 0.31268 0.72561 0.03340 Uani 1.00

H98 H 0.69831 0.41301 0.68853 0.05000 Uiso 1.00

N99 N 0.31616 0.74530 0.69003 0.03340 Uani 1.00

H100 H 0.41662 0.71144 0.66061 0.05000 Uiso 1.00

N101 N 0.24328 0.27870 0.69941 0.03340 Uani 1.00

H102 H 0.27956 0.31414 0.59889 0.05000 Uiso 1.00

N103 N 0.26875 0.28993 0.26702 0.03340 Uani 1.00

H104 H 0.30108 0.32472 0.30110 0.05000 Uiso 1.00

N105 N 0.29070 0.69190 0.24510 0.03340 Uani 1.00

H106 H 0.32067 0.59080 0.28481 0.05000 Uiso 1.00

N107 N 0.66692 0.28382 0.28019 0.03340 Uani 1.00

H108 H 0.56567 0.31913 0.31400 0.05000 Uiso 1.00

H109 H 0.51917 0.36269 0.07091 0.00000 Uiso 1.00

H110 H 0.57754 0.48063 -0.02978 0.00000 Uiso 1.00

H111 H 0.40950 0.51811 0.03135 0.00000 Uiso 1.00

H112 H 0.54095 0.36086 0.54752 0.00000 Uiso 1.00

H113 H 0.60779 0.46869 0.49224 0.00000 Uiso 1.00

H114 H 0.47644 0.45781 0.62984 0.00000 Uiso 1.00

H115 H 0.06170 0.38773 0.53353 0.00000 Uiso 1.00

H116 H 0.04453 0.54205 0.55461 0.00000 Uiso 1.00

H117 H -0.06075 0.51345 0.51410 0.00000 Uiso 1.00

H118 H 0.48680 -0.06481 0.46474 0.00000 Uiso 1.00

H119 H 0.61588 -0.07060 0.47729 0.00000 Uiso 1.00

H120 H 0.47444 -0.06912 0.61160 0.00000 Uiso 1.00

H121 H 0.81132 0.79606 0.97028 0.00000 Uiso 1.00

H122 H 0.94784 0.80010 0.96468 0.00000 Uiso 1.00

H123 H 0.81268 0.93031 0.96877 0.00000 Uiso 1.00

H124 H 0.03805 0.04568 0.25068 0.00000 Uiso 1.00

H125 H 0.17631 0.04864 0.24080 0.00000 Uiso 1.00

H126 H 0.04004 0.17977 0.24789 0.00000 Uiso 1.00

loop_

_atom_site_aniso_label

_atom_site_aniso_U_11

_atom_site_aniso_U_22

_atom_site_aniso_U_33

_atom_site_aniso_U_12

_atom_site_aniso_U_13

_atom_site_aniso_U_23

Cl1 0.03720 0.03720 0.03720 -0.01240 -0.01240 -0.01240

Cl2 0.03720 0.03720 0.03720 -0.01240 -0.01240 -0.01240

O3 0.05133 0.05133 0.17400 0.00333 -0.05800 -0.05800

O4 0.05133 0.05133 0.05133 0.00333 0.00333 0.00333

O5 0.17400 0.05133 0.05133 -0.05800 -0.05800 0.00333

O6 0.05133 0.17400 0.05133 -0.05800 0.00333 -0.05800

N7 0.04070 0.04070 0.03857 -0.01477 -0.01450 -0.01450

N8 0.05047 0.05047 0.03233 -0.02153 -0.01113 -0.01113

N10 0.04070 0.04070 0.03243 -0.01477 -0.01143 -0.01143

N11 0.05047 0.05047 0.04567 -0.02153 -0.01780 -0.01780

N13 0.03857 0.03243 0.04070 -0.00957 -0.01450 -0.01143

N14 0.03233 0.04567 0.05047 -0.01007 -0.01113 -0.01780

N16 0.03243 0.03857 0.04070 -0.00957 -0.01143 -0.01450

N17 0.04567 0.03233 0.05047 -0.01007 -0.01780 -0.01113

N19 0.03857 0.04070 0.04070 -0.01450 -0.01450 -0.01477

N20 0.03233 0.05047 0.05047 -0.01113 -0.01113 -0.02153

N22 0.03243 0.04070 0.04070 -0.01143 -0.01143 -0.01477

N23 0.04567 0.05047 0.05047 -0.01780 -0.01780 -0.02153

N25 0.04070 0.03857 0.03243 -0.01450 -0.01143 -0.00957

N26 0.05047 0.03233 0.04567 -0.01113 -0.01780 -0.01007

N28 0.04070 0.03243 0.03857 -0.01143 -0.01450 -0.00957

N29 0.05047 0.04567 0.03233 -0.01780 -0.01113 -0.01007

N31 0.04070 0.03857 0.04070 -0.01450 -0.01477 -0.01450

N32 0.05047 0.03233 0.05047 -0.01113 -0.02153 -0.01113

N34 0.04070 0.03243 0.04070 -0.01143 -0.01477 -0.01143

N35 0.05047 0.04567 0.05047 -0.01780 -0.02153 -0.01780

N37 0.03243 0.04070 0.03857 -0.01143 -0.00957 -0.01450

N38 0.04567 0.05047 0.03233 -0.01780 -0.01007 -0.01113

N40 0.03857 0.04070 0.03243 -0.01450 -0.00957 -0.01143

N41 0.03233 0.05047 0.04567 -0.01113 -0.01007 -0.01780

N43 0.04070 0.04070 0.03243 -0.01477 -0.01143 -0.01143

N44 0.05047 0.05047 0.04567 -0.02153 -0.01780 -0.01780

N46 0.04070 0.04070 0.03857 -0.01477 -0.01450 -0.01450

N47 0.05047 0.05047 0.03233 -0.02153 -0.01113 -0.01113

N49 0.03857 0.03243 0.04070 -0.00957 -0.01450 -0.01143

N50 0.03233 0.04567 0.05047 -0.01007 -0.01113 -0.01780

N52 0.03243 0.03857 0.04070 -0.00957 -0.01143 -0.01450

N53 0.04567 0.03233 0.05047 -0.01007 -0.01780 -0.01113

N55 0.03857 0.04070 0.03243 -0.01450 -0.00957 -0.01143

N56 0.03233 0.05047 0.04567 -0.01113 -0.01007 -0.01780

N58 0.03243 0.04070 0.03857 -0.01143 -0.00957 -0.01450

N59 0.04567 0.05047 0.03233 -0.01780 -0.01007 -0.01113

N61 0.04070 0.03857 0.04070 -0.01450 -0.01477 -0.01450

N62 0.05047 0.03233 0.05047 -0.01113 -0.02153 -0.01113

N64 0.04070 0.03243 0.04070 -0.01143 -0.01477 -0.01143

N65 0.05047 0.04567 0.05047 -0.01780 -0.02153 -0.01780

N67 0.04070 0.03857 0.03243 -0.01450 -0.01143 -0.00957

N68 0.05047 0.03233 0.04567 -0.01113 -0.01780 -0.01007

N70 0.04070 0.03243 0.03857 -0.01143 -0.01450 -0.00957

N71 0.05047 0.04567 0.03233 -0.01780 -0.01113 -0.01007

N73 0.03243 0.04070 0.04070 -0.01143 -0.01143 -0.01477

N74 0.04567 0.05047 0.05047 -0.01780 -0.01780 -0.02153

N76 0.03857 0.04070 0.04070 -0.01450 -0.01450 -0.01477

N77 0.03233 0.05047 0.05047 -0.01113 -0.01113 -0.02153

N79 0.04493 0.04493 0.04373 -0.02093 -0.01200 -0.01200

N80 0.04373 0.04373 0.04493 -0.01973 -0.01200 -0.01200

N81 0.04373 0.04493 0.04493 -0.01200 -0.01200 -0.02093

N82 0.04493 0.04373 0.04373 -0.01200 -0.01200 -0.01973

N83 0.04493 0.04373 0.04493 -0.01200 -0.02093 -0.01200

N84 0.04373 0.04493 0.04373 -0.01200 -0.01973 -0.01200

N85 0.04493 0.04493 0.04373 -0.02093 -0.01200 -0.01200

N86 0.04373 0.04373 0.04493 -0.01973 -0.01200 -0.01200

N87 0.04373 0.04493 0.04373 -0.01200 -0.01973 -0.01200

N88 0.04493 0.04373 0.04493 -0.01200 -0.02093 -0.01200

N89 0.04493 0.04373 0.04373 -0.01200 -0.01200 -0.01973

N90 0.04373 0.04493 0.04493 -0.01200 -0.01200 -0.02093

O91 0.07100 0.07100 0.07100 -0.02367 -0.02367 -0.02367

O92 0.07100 0.07100 0.07100 -0.02367 -0.02367 -0.02367

N93 0.03427 0.03427 0.03427 -0.01200 -0.01200 -0.01200

N95 0.03427 0.03427 0.03080 -0.01200 -0.01027 -0.01027

N97 0.03427 0.03080 0.03427 -0.01027 -0.01200 -0.01027

N99 0.03080 0.03427 0.03427 -0.01027 -0.01027 -0.01200

N101 0.03427 0.03427 0.03080 -0.01200 -0.01027 -0.01027

N103 0.03427 0.03427 0.03427 -0.01200 -0.01200 -0.01200

N105 0.03427 0.03080 0.03427 -0.01027 -0.01200 -0.01027

N107 0.03080 0.03427 0.03427 -0.01027 -0.01027 -0.01200

loop_

_geom_bond_atom_site_label_1

_geom_bond_atom_site_label_2

_geom_bond_distance

_geom_bond_site_symmetry_2

_ccdc_geom_bond_type

O3 H109 1.043 . S

O3 H110 1.017 . S

O3 H111 1.027 . S

O4 H112 1.065 . S

O4 H113 1.008 . S

O4 H114 1.053 . S

O5 H115 1.015 . S

O5 H116 1.315 . S

O5 H117 0.974 . S

O6 H118 1.022 . S

O6 H119 1.014 . S

O6 H120 1.070 . S

N7 N8 1.323 . S

N7 N10 1.327 . S

N8 N79 1.324 . S

H9 N93 1.047 . S

N10 N11 1.322 . S

N11 N79 1.324 . S

H12 N95 1.049 . S

N13 N14 1.330 . S

N13 N16 1.320 . S

N14 N80 1.319 . S

H15 N97 1.051 . S

N16 N17 1.327 . S

N17 N80 1.329 . S

H18 N99 1.051 . S

N19 N20 1.254 . S

N19 N22 1.730 . S

N20 N81 1.209 . S

H21 N93 1.027 . S

N22 N23 1.171 . S

N23 N81 1.881 . S

H24 N99 1.036 . S

N25 N26 1.319 . S

N25 N28 1.324 . S

N26 N82 1.331 . S

H27 N95 1.040 . S

N28 N29 1.322 . S

N29 N82 1.316 . S

H30 N97 1.038 . S

N31 N32 1.324 . S

N31 N34 1.316 . S

N32 N83 1.332 . S

H33 N93 1.052 . S

N34 N35 1.332 . S

N35 N83 1.315 . S

H36 N97 1.043 . S

N37 N38 1.321 . S

N37 N40 1.328 . S

N38 N84 1.318 . S

H39 N99 1.047 . S

N40 N41 1.319 . S

N41 N84 1.330 . S

H42 N95 1.038 . S

N43 N44 1.333 . S

N43 N46 1.318 . S

N44 N85 1.318 . S

H45 N101 1.045 . S

N46 N47 1.326 . S

N47 N85 1.330 . S

H48 N103 1.043 . S

N49 N50 1.320 . S

N49 N52 1.331 . S

N50 N86 1.329 . S

H51 N105 1.036 . S

N52 N53 1.322 . S

N53 N86 1.320 . S

H54 N107 1.036 . S

N55 N56 1.329 . S

N55 N58 1.323 . S

N56 N87 1.328 . S

H57 N101 1.044 . S

N58 N59 1.331 . S

N59 N87 1.319 . S

H60 N107 1.050 . S

N61 N62 1.322 . S

N61 N64 1.330 . S

N62 N88 1.319 . S

H63 N103 1.040 . S

N64 N65 1.320 . S

N65 N88 1.331 . S

H66 N105 1.038 . S

N67 N68 1.329 . S

N67 N70 1.324 . S

N68 N89 1.317 . S

H69 N101 1.049 . S

N70 N71 1.323 . S

N71 N89 1.331 . S

H72 N105 1.046 . S

N73 N74 1.322 . S

N73 N76 1.328 . S

N74 N90 1.320 . S

H75 N107 1.036 . S

N76 N77 1.319 . S

N77 N90 1.328 . S

H78 N103 1.041 . S

O91 H121 1.064 . S

O91 H122 1.032 . S

O91 H123 1.018 . S

O92 H124 1.043 . S

O92 H125 1.036 . S

O92 H126 1.036 . S

N93 H94 1.042 . S

N95 H96 1.048 . S

N97 H98 1.042 . S

N99 H100 1.043 . S

N101 H102 1.044 . S

N103 H104 1.050 . S

N105 H106 1.051 . S

N107 H108 1.051 . S
